# Limited movement of an avian hybrid zone in relation to regional variation in magnitude of climate change

**DOI:** 10.1111/mec.16727

**Published:** 2022-10-21

**Authors:** Alana Alexander, Mark B. Robbins, Jesse Holmes, Robert G. Moyle, A. Townsend Peterson

**Affiliations:** ^1^ Biodiversity Institute University of Kansas Lawrence Kansas USA; ^2^ Department of Anatomy University of Otago Dunedin New Zealand; ^3^ Department of Ecology and Evolutionary Biology University of Kansas Lawrence Kansas USA

**Keywords:** climate change, genomic cline, geographic cline, hybridization, Paridae, *Poecile*

## Abstract

Studies of natural hybrid zones can provide documentation of range shifts in response to climate change and identify loci important to reproductive isolation. Using a temporal (36–38 years) comparison of the black‐capped (*Poecile atricapillus*) and Carolina (*P. carolinensis*) chickadee hybrid zone, we investigated movement of the western portion of the zone (western Missouri) and assessed whether loci and pathways underpinning reproductive isolation were similar to those in the eastern portion of the hybrid zone. Using 92 birds sampled along the hybrid zone transect in 2016 and 68 birds sampled between 1978 and 1980, we generated 11,669 SNPs via ddRADseq. These SNPs were used to assess movement of the hybrid zone through time and to evaluate variation in introgression among loci. We demonstrate that the interface has moved ~5 km to the northwest over the last 36–38 years, that is, at only one‐fifth the rate at which the eastern portion (e.g., Pennsylvania, Ohio) of the hybrid zone has moved. Temperature trends over the last 38 years reveal that eastern areas have warmed 50% more than western areas in terms of annual mean temperature, possibly providing an explanation for the slower movement of the hybrid zone in Missouri. Our results suggest hybrid zone movement in broadly distributed species, such as chickadees, will vary between areas in response to local differences in the impacts of climate change.

## 1 INTRODUCTION

Hybrid zones are fundamental for understanding the mechanisms underpinning reproductive isolation (Taylor & Larson, 2019) and speciation (Gompert, Parchman, et al., 2012). In addition, they can provide evidence of range shifts in response to anthropogenic impacts, including habitat modification (Thurman et al., 2019) and climate change (Arntzen, 2019; Ryan et al., 2018; Taylor et al., 2015). One of the most tractable ways to document temporal shifts in hybrid zones is via comparisons of spatial positions of hybrid zones between contemporary and historical samples, and museum collections are invaluable in this regard (Thurman et al., 2019; Wang et al., 2019). Birds have been a frequent subject of hybrid zone studies because their ease of observation facilitates broad characterization of hybrid zones at continental scales.

Many avian hybrid zones studied in North America are oriented roughly longitudinally: for example, meadowlarks (Rohwer, 1972), buntings (Carling et al., 2010; Carling & Brumfield, 2008; Emlen et al., 1975), orioles (Carling et al., 2011; Rising, 1970; Sibley & Short, 1964; Walsh et al., 2020), phoebes (Schukman et al., 2011), and pewees (Manthey & Robbins, 2016). In contrast, the largely latitudinal orientation of the black‐capped (*Poecile atricapillus*)/Carolina (*P. carolinensis*) chickadee hybrid zone (except for extreme western Missouri/southeastern Kansas), makes it particularly relevant in a climate change context as it aligns more consistently with latitudinal temperature patterns. Indeed, this contact zone has been sampled and analysed extensively (Braun & Robbins, 1986; Brewer, 1963; Bronson et al., 2005; Bronson, Grubb, & Braun, 2003; Bronson, Grubb, Sattler, et al., 2003; Curry, 2005; Johnston, 1971; Merritt, 1978; Reudink et al., 2007; Rising, 1968; Robbins et al., 1986; Tanner, 1952; Taylor, Curry, et al., 2014; Taylor, White, et al., 2014; Wagner et al., 2020; Ward & Ward, 1974).

Although the black‐capped/Carolina chickadee hybrid zone ranges from southeastern Kansas to New Jersey (AOU, 1998, https://ebird.org/species/bkcchi/, https://ebird.org/species/carchi/), most research has focused on the eastern portion (Bronson, Grubb, & Braun, 2003; Bronson, Grubb, Sattler, et al., 2003; Curry, 2005; Reudink et al., 2007; Taylor, Curry, et al., 2014; Taylor, White, et al., 2014; Wagner et al., 2020). It has been proposed that the hybrid zone location may be determined by winter temperatures, which may limit the northward range of Carolina chickadees (Taylor, White, et al., 2014). This limitation is potentially mediated by differences in metabolism and competitive ability between the two species (McQuillan & Rice, 2015; Olson et al., 2010). In addition, the hybrid zone is relatively narrow (Taylor, White, et al., 2014), probably caused by reduced reproductive success of hybrids (Bronson et al., 2005; Bronson, Grubb, & Braun, 2003). Learning and memory impairment (e.g., recall ability for location of stored food caches) in hybrid chickadees may contribute to this reduced reproductive success (McQuillan et al., 2018).

Morphological studies in Pennsylvania and Ohio have demonstrated that the hybrid zone has moved northward at >1 km/year for over 100 years (Brewer, 1963; Bronson, Grubb, Sattler, & Braun, 2003; Harr & Price, 2014) and this northward movement of the hybrid zone has been confirmed genetically and associated with climate change (Reudink et al., 2007; Taylor, White, et al., 2014). However, movement of the zone has been predicted to differ geographically, with ecological niche models indicating a retraction of suitable habitat in the western portion of the Carolina chickadee distribution (McQuillan & Rice, 2015). Analysis of song data in Illinois supports these models, with little hybrid zone movement detected (Enstrom & Bollinger, 2009), but song and morphology are less robust indicators of hybridization than genetic markers owing to extreme similarities in plumage morphology, intraspecific song variation, and heterospecific song learning between these species (Bronson, Grubb, Sattler, et al., 2003; Johnston, 1971; Kroodsma et al., 1995; Robbins et al., 1986; Sattler et al., 2007; Sattler & Braun, 2000; Shackleton & Ratcliffe, 1993; Tanner, 1952). In spite of the existence of early analyses (Braun & Robbins, 1986; Robbins et al., 1986), data are lacking on the magnitude of hybrid zone shifts in the farthest western portions of the range (e.g. Missouri and Kansas) (McQuillan & Rice, 2015).

In addition to movement of hybrid zones as a whole, the influence of localized selective pressures on the introgression of genes linked to reproductive isolation is of interest at contact zones (Gompert et al., 2017; Harrison & Larson, 2016; Moran et al., 2020; Taylor & Larson, 2019). Comparisons of transects in different portions of broadly distributed contact zones, such as the chickadees, are therefore of particular interest. Previous genetic analyses of the chickadee hybrid zone in eastern Pennsylvania have identified genes underpinning metabolic and neural signalling pathways as being subject to temporally consistent restriction in introgression across the hybrid zone (Taylor, Curry, et al., 2014; Wagner et al., 2020). In addition, these studies affirmed that SNPs associated with sex chromosome Z are particularly resistant to introgression (Taylor, Curry, et al., 2014; Wagner et al., 2020), a pattern seen in other avian systems (Battey, 2020; Bourgeois et al., 2020) and analogously in systems involving chromosome X (Carneiro et al., 2014; Janoušek et al., 2012; Maroja et al., 2015). These temporally‐consistent specific genes resistant to introgression support observations about differences in metabolic capability between black‐capped and Carolina chickadees, and of memory deficiency in hybrids (McQuillan et al., 2018). However, no information exists regarding whether these specific genes and associated metabolic pathways are spatially consistent. That is, are the same regions of the genome resistant to introgression 1500 km to the west in Missouri, in an area subject to different local selective pressures?

In 2016, we resampled a segment of the hybrid zone in west‐central Missouri that had been sampled intensively by one of us in 1978–1980 (Braun & Robbins, 1986; Robbins et al., 1986). At 36–38 years apart, these samples provide not only the deepest temporal genetic comparison of the chickadee hybrid zone interface, but indeed one of the deepest of any avian contact zone in North America. We demonstrated limited northwest movement of the hybrid zone in Missouri as compared to other areas of the USA. A comparison with climate data for the same time period suggests that eastern areas of the USA have warmed 50% more than Missouri in terms of annual mean temperature, providing the beginnings of an explanation for the slower movement of the hybrid zone in Missouri. Our results suggest that specific impacts of climate change on broadly distributed species will manifest at local scales and provides further illustration of how crucial museum collections are in assessing the impacts of climate change.

## 2 MATERIALS AND METHODS

### 2.1 Fieldwork and selection of historical samples

**FIGURE 1:**
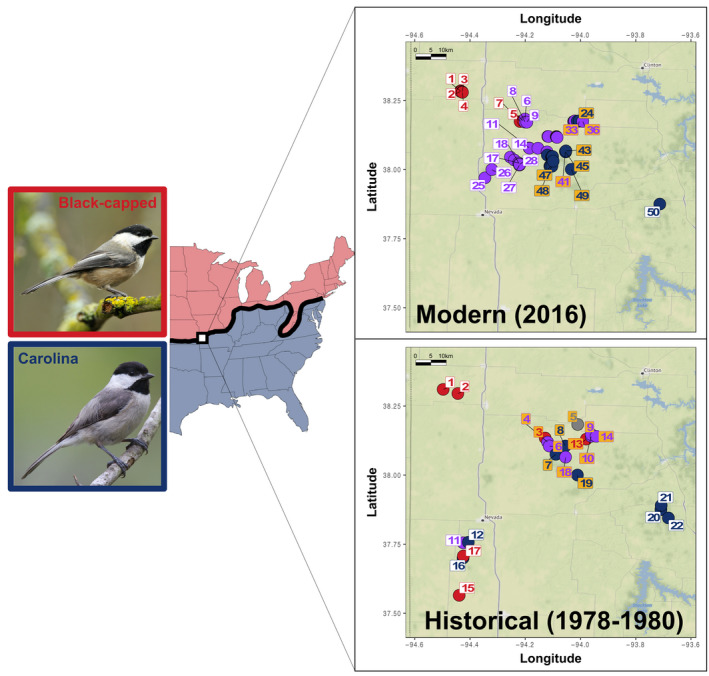
The black‐capped and Carolina chickadee Missouri hybrid zone transect. Overall extent of hybrid zone and images of black‐capped and Carolina chickadees shown on far left. Right panel gives spatial location of sampling sites (shown by circles on map), with dotted line within maps indicating Kansas/Missouri border. Sampling sites are coloured red if only black‐capped birds present (individual assignment of structure for all birds >95% to black‐capped cluster), blue if only Carolina present, and purple if hybrids and/or mix of parental species present. Sampling sites highlighted in yellow used for spatial interpolation of hybrid zone movement (the zoomed in extent shown in left panel of Figure 2). Map tiles provided by Stamen Design, under CC BY 3.0. Map data by OpenStreetMap, under ODbL. Figure generated using code presented at https://github.com/laninsky/chickadees. Images via Wikimedia Commons (black‐capped chickadee: Minette Layne, Carolina chickadee: Dan Pancamo)

The same west‐central Missouri transect that was sampled by Robbins in 1978 and 1980 (Figure 1 in Robbins et al., 1986) was sampled again by Robbins in March–April 2016 (Table S1, Figure 1). Of the 92 chickadees collected in 2016, 17 were obtained from parental populations classified as “pure” (putatively nonadmixed) during sampling in 1978–1980 based on morphological and vocal variation (Robbins et al., 1986). For the Carolina chickadee, these 10 “pure” samples were taken from the Bird Song Conservation Area, St. Clair County (Site 50 in top panel of Figure 1; equivalent to Sites 20–22 in the bottom panel of Figure 1 and Site 4 in Robbins et al., 1986). For the black‐capped chickadee, *n* = 7 “pure” samples were taken from the upper Miami Creek drainage northwest of Butler, Bates County (Sites 1–4 in top panel of Figure 1, equivalent to Sites 1–2 in the bottom panel of Figure 1 and Site 1 in Robbins et al., 1986). We also included a further five reference birds (three black‐capped and two Carolina) sampled from well outside the putative contact zone (locations in Table S1), just in case the hybrid zone was wider than it appeared in Robbins et al. (1986).

The remaining 75 samples from 2016 were taken from within the contact zone, which was more intensively sampled than in 1978–1980, including samples from several additional sites. For both sampling periods, when possible, chickadees were audio‐recorded, then collected, and immediately frozen on dry ice. The protocol and procedures employed during collection were reviewed and approved by the University of Kansas Institutional Animal Care and Use Committee. Samples were archived in either −80°C freezers (1978–1980 samples) or in liquid nitrogen (2016 samples). Voucher study skins (*n* = 92) and genetic material from the 2016 study are deposited at the University of Kansas Biodiversity Institute. Specimen data (including links to audio recordings) for all 2016 samples are accessible via vertnet (vertnet.org). Audio recordings from both 1978–1980 and 2016 are deposited at the Macaulay Library, Cornell Laboratory of Ornithology, Ithaca, New York. The 1978–1980 genetic samples are deposited at the United States National Museum, Smithsonian Institution, whereas associated voucher specimens are deposited at Louisiana State University of Natural Science, Baton Rouge, Louisiana.

In all, 68 genetic samples were included from the 1978–1980 study. We included 10 of 17 and 10 of 21 total birds available from upper Miami Creek (Sites 1–2 in bottom panel of Figure 1) and Collins (Sites 20–22 in the bottom panel of Figure 1), respectively, to reflect more closely the numbers of samples taken from those locations in 2016 (*n =* 7 birds across Sites 1–4, and *n* = 10 at Site 50, respectively, top panel of Figure 1), as based on the results of the 1978–1980 study, these sites were expected to reflect nonadmixed black‐capped and Carolina chickadee populations.

### 2.2 DNA extraction

DNA was extracted from ~15 mg of tissue using a Blood DNA kit and manufacturer protocols on a Maxwell RSC instrument (Promega), with the following modifications: before loading into the cartridge, samples were lysed for 24 h with 32 μl of proteinase K and 180 μl of tissue lysis buffer (Promega) in a 1.5 ml tube on a heat block at 56°C before being spun for 2 min at maximum speed to pellet any remaining tissue at the bottom of the tube. The supernatant was then transferred to well 1 of the cartridge. The volume of elution buffer used was 100 μl. DNA was quantified using the quantifluor dsDNA System.

### 2.3 Laboratory methods for ddRADseq

We used a double‐digest RADseq protocol (Peterson et al., 2012) with the restriction enzymes *SbfI* and *MspI*, pooling sets of 8–16 samples (distinguished using internal barcodes), with pools distinguished by external barcodes (Table S2; additional details on protocol given in Supporting Information Methods). An initial set of eight samples was sequenced on 5% of a HiSeq 3000 paired‐end 150 bp lane at the Oklahoma Medical Research Foundation (OMRF). Following this successful test run, the remaining 157 samples were prepared and combined in pools of 15–16 individuals. After combining the pools at equimolar concentrations, the final library (of 191 individuals, including 34 samples unrelated to this project) was sequenced on a paired‐end 150 bp HiSeq3000 run.

### 2.4 ddRADseq data analysis and identification of genetic clusters

Our SNP data set was generated by mapping reads to the black‐capped chickadee genome (Wagner et al., 2020; BioSample: SAMN13264372; BioProject: PRJNA589043; Assembly accession: GCA_011421415.1) through ipyrad version 0.9.51 (Eaton & Overcast, 2020). To be included in the final data set, loci were required to be found in at least one of the reference black‐capped and one of the reference Carolina samples. Specific code/parameters used for this analysis and all other downstream analyses in this study are detailed at https://github.com/laninsky/chickadees.

From this data set, we selected one variable site per locus, and used custom r code to filter out singletons, as per the recommendations of Linck and Battey (2019) for running structure (Falush et al., 2003; Pritchard et al., 2000). We used this data set as input into the program structure version 2.3.4 run via structure_threader version 1.3.0 (Pina‐Martins et al., 2017). We carried out an initial run at *K* = 1 to infer lambda, using 50,000 burnin steps, followed by 100,000 steps. We fixed lambda at its inferred value and then carried out five replicates for *K* = 1 to *K* = 5 under the ancestry admixture model and allowing for correlated allele frequencies. The Evanno method (Evanno et al., 2005) was used to assess the best‐fitting *K* through structure harvester (Earl & vonHoldt, 2012), and individual structure assignments to each cluster were calculated for the best fitting *K* averaged across the five replicates. To verify these results using an additional method of assessing ancestry, we also ran a PCA on the structure input file using the r package smartsnp version 1.1.0 (Herrando‐Pérez et al., 2021).

### 2.5 Movement of hybrid zone

**FIGURE 2:**
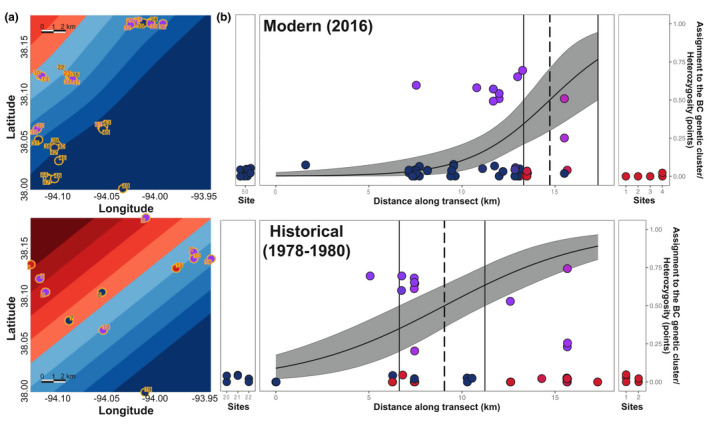
Movement of Missouri hybrid zone through time. (a) Spatial interpolation of 2016 samples shown on top, 1978–1980 samples shown on bottom. Note, dark red contour not observed across 2016 sites so analyses of hybrid zone movement are restricted to the position of the black‐capped/Carolina interface (the red/blue interface), rather than considering width of hybrid zone. Numbered sample sites correspond to those given in Figure 1. (b) Geographic cline analysis of the change in black‐capped (BC) chickadee ancestry with distance along transect, assuming a strict southwest (left) to northeast (right) direction for samples from same sites as in left panel. Ribbon gives the 95% confidence interval of the geographic cline estimated for the 2016 samples (top) and 1978–1980 samples (bottom). The line in the centre of ribbons is mean estimated geographic cline. Solid vertical lines correspond to minimum and maximum 95% confidence intervals of the centre of the genomic cline, with dashed lines giving the estimated centre. Heterozygosity for individual samples shown as points (*y*‐axis), coloured by structure assignment (blue, Carolina; red, black‐capped), including for “allopatric” sites on each side of detailed zone of sampling (additional sites not included in both sampling periods given in Figure S4). Code for generating this figure is given at https://github.com/laninsky/chickadees.

Sampling locations were plotted using program r (R Core Team, 2022), along with the dplyr (Wickham et al., 2018), ggmap (Kahle & Wickham, 2013), ggplot2 (Wickham, 2016), ggrepel (Slowikowski, 2017), and readr (Wickham et al., 2017) packages. The plot function of tess3r (Caye et al., 2016; Caye & Francois, 2016) was used to interpolate structure assignments spatially to assess hybrid zone movement between the modern and historical sampling periods. This analysis was also repeated using PC1 scores as an input. Analyses of the movement of the hybrid zone were restricted to the area of overlap between the two sampling periods (yellow background in labels on Figure 1) to restrict the influence of sampling sites that were not well matched between the temporal samples (e.g., sites 5–9 in 2016 sample; sites 11, 12, 15–17 in 1978–1980 sample, Figure 1). After confirming that the hybrid zone interface ran from the southwest to the northeast with the tess3r analysis, we calculated the distance to each of our samples from a southwest‐northeast line centred on the southeast portion of the study area shown in Figure 2a. We then used the structure assessments of genomic admixture to conduct a geographic cline analysis using hzar version 0.2.5 separately for the 2016 and 1978–1980 samples (Derryberry et al., 2014), also repeating this analysis using the PC1 scores as the input measure of genomic admixture. In addition to these measures of admixture for comparisons between the two temporal sampling periods, we also calculated a Hybrid Index for our samples using gghybrid version 2.0.0 (Bailey, 2022) and characterized heterozygosity of our samples using SNPs that were fixed between putatively unadmixed individuals (individuals that showed ≥99% assignment to the respective genetic cluster based on the previous structure analysis).

### 2.6 Variation in patterns of introgression by locus

We identified loci putatively involved in reproductive isolation between black‐capped and Carolina chickadees by carrying out a genomic cline analysis in BGC version 1.0.3 (Gompert & Buerkle, 2012), following the approach of Taylor, Curry, et al. (2014). Black‐capped and Carolina parental “populations” were defined as individuals that showed ≥99% assignment to the respective genetic cluster based on the previous structure analysis, with the admixed population including all remaining individuals. Given the limited geographic extent of the Missouri hybrid zone that we studied, nested population effects were not included in our model; instead, the hybrid zone was considered as a single population, following Gompert and Buerkle (2011). The analysis was conducted across all samples because the shared ancestry across the temporal sampling periods means they cannot be considered independent (Taylor, Curry, et al., 2014) and we did not limit the samples to just those from the more concentrated overlapping region used in the geographic cline analysis. We restricted loci to those found in ≥90% of our samples to limit the total number of loci owing to computational constraints. We implemented the genotype uncertainty model of Gompert, Lucas, et al. (2012). Parameter estimates were based on the median of the marginal posterior probability distribution across our 50,000 MCMC state chain (sampling every fifth state), which followed a 25,000‐iteration burnin. We confirmed convergence of parameter estimates by running a second shorter chain (25,000 MCMC stats, 12,500 burnin).

Loci for which 95% posterior probability intervals did not overlap 0 and where median α and/or β values were in the top/bottom 1% of all loci were classified as outliers following Galaverni et al. (2017). Positive α outliers have an increase in the probability of black‐capped ancestry in comparison to that predicted by the hybrid index (i.e., more black‐capped than expected); negative α have an increase in the probability of Carolina ancestry; positive β outliers have excess ancestry‐based linkage disequilibrium (i.e., locus‐specific ancestry restricted to matching genomic background, potentially indicating loci that are less free to introgress across the hybrid zone); negative β outliers have ancestry less strongly associated with genomic background than in other loci (i.e., loci are more free to introgress). We investigated significant differences in how these outlier loci were distributed across chromosomes using G‐tests.

Because positive β outliers (less freely introgressing loci) could be associated with reproductive isolation between the species (Gompert, Parchman, & Buerkle, 2012), we focused on such loci for additional comparisons. First, we identified consecutive SNPs that were positive β outliers, potentially indicative of broader regions (e.g., inversions/nonrecombining areas of chromosomes) of reduced introgression. We used a cutoff of three consecutive loci, which would be unlikely to occur by chance if positive β outliers were randomly distributed across our data set. We extracted the sequence from these regions using seqtk version 1.3 (Li, 2020), and used magic‐blast version 1.5.0 (Boratyn et al., 2019) to match these regions to nucleotide sequence from black‐capped chickadee coding sequences (CDS) identified using a different black‐capped reference genome (GCA_013398625.1_ASM1339862v1_cds; Bird 10,000 Genomes [B10K] Project – Family phase). A direct comparison to the reference genome that we used for the rest of our analyses (GCA_011421415.1) was not possible, as annotations are not yet available for this genome (however, GCA_011421415.1 had higher contiguousness than GCA_013398625.1, making it more suitable for the reference‐based steps of our analyses).

We carried out an analysis of biological processes enriched among the genes associated with our outlier SNPs using gene ontology (GO) annotation through http://geneontology.org/ (panther overrepresentation test [released 20,220,712]; GO Ontology database doi: https://doi.org/10.5281/zenodo.6399963 released 2022‐03‐22; *Homo sapiens* reference list. *Homo sapiens* was selected as the reference list was more complete than the avian genomes available), with a Fisher's exact test, and a false discovery rate for multiple comparisons. We then repeated these analyses (extracting sequence, magic‐blast to identify whether SNPs were near/within CDS regions, GO term enrichment) for all significant positive β outlier SNPs, using 25,000 bp of flanking sequence on each side of the SNP. Finally, we compared the positive β outliers (and associated genes) identified in our analyses with those identified in previous genetic investigations of the black‐capped/Carolina chickadee hybrid zone (Taylor, Curry, et al., 2014; Wagner et al., 2020).

### 2.7 Climate analyses

To provide an environmental context for the genetic analyses, annual precipitation and mean annual temperature data were downloaded from PRISM (2017). All data for 1976–1980, 1998–2002, 2008–2012, and 2012–2016 were downloaded in *.bil format. These date ranges were selected to correspond to the 5 years prior to the start and end dates of the studies in Missouri (1980–2016) and Pennsylvania (2002–2012). We derived two estimates of the rate of change of temperature and precipitation: one based on the 1980–2016 interval, and the other on the 2002–2012 interval. We averaged each climate dimension over the appropriate 5‐year range. We calculated the change in temperature as the average of conditions during the end of the interval minus the average of the 5 years preceding the beginning of the interval. We then calculated the rate of change by dividing change by the number of years covered by this period (e.g., for Missouri, 2016–1980 = 36 years).

**FIGURE 3:**
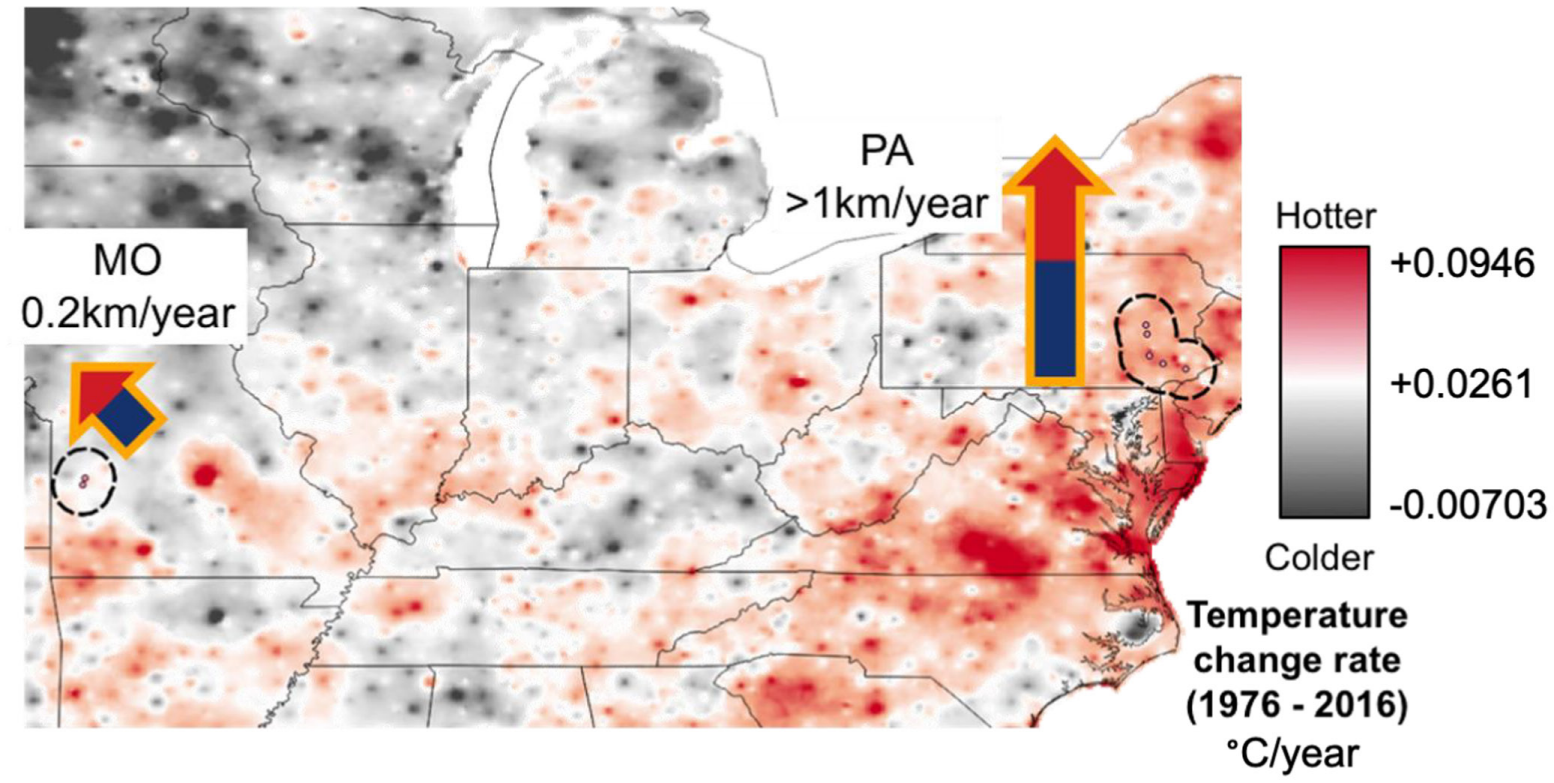
Slower movement of the black‐capped and Carolina chickadee hybrid zone is associated with less temperature change in Missouri (MO), compared with Pennsylvania (PA). Rate of temperature change between 1976–1980 and 2012–2016 is based on five‐year means. Sample sites used to infer climatic trends at each location are listed in Table S3.

To examine consistency in rates of change between 1978–2014 and 2000–2010, we examined correlations in the rates of change between these two time periods. Following this exploratory analysis, we examined longer‐term (38 years, i.e., the duration of our Missouri study) and shorter‐term (10 years, i.e., the duration of the Pennsylvania study Taylor, White, et al., 2014) trends at each of the sites (Table S3). Overall, we conducted two separate contrasts, 1998–2002 versus 2008–2012 (corresponding to the Pennsylvania study time frame), and 1976–1980 versus 2012–2016 (corresponding to our study in Missouri). We generated frequency histograms of rates of realized change in each environmental dimension within the 0.5° (~55 km) buffers shown as dashed lines in Figure 3.

## 3 RESULTS

### 3.1 Summary of ddRADseq data set and initial structure runs

Detail on the number of reads obtained and levels of missingness in our data set are provided in Supporting Information Results and in Figures S1 and S2, Table S4. Based on 8056 SNPs, the Evanno et al. (2005) method selected a *K* of 2 for our structure analyses, consistent with our samples spanning two separate species. Our results suggest that we can distinguish between the unadmixed parental species: four of the five reference samples we collected well away from the hybrid zone were inferred to belong to the “pure” populations they were purported to represent (99.9% assignment to respective genetic clusters, Table S1), and we observed a strong gradient of genomes ranging from “pure” black‐capped (*n =* 34) to admixed individuals (*n* = 79) to “pure” Carolina chickadees (*n =* 51, Table S1) across our transect. The remaining black‐capped chickadee reference sample (catalogue no. 95776), showed an assignment of 93.5% to the black‐capped chickadee cluster, despite being sampled even further away from the hybrid zone than the other black‐capped reference samples. structure assignments were also strongly correlated with the alternative method of assessing ancestry we employed, PCA (Pearson's correlation = 0.979; Figure S3). For this reason, downstream analyses using structure assignments are presented in the main manuscript, with analyses based on PC1 scores presented at Figure S4. The five reference samples were then excluded from downstream analyses, except for the genomic cline analyses and calculation of Hybrid Index values.

### 3.2 Movement of hybrid zone

Spatial interpolation of the structure assignments of birds sampled in 1978–1980 in comparison with samples from 2016 showed that the contact zone has moved ~5 km to the northwest over the last 36–38 years (Figure 2a). To estimate quantitatively the movement of the hybrid zone, we assumed the hybrid zone interface had moved strictly to the northwest. The geographic cline analysis indicated that the hybrid zone had moved 5.66 km (Figure 2b). This pattern of movement was also supported by comparisons of the locations with fine‐scale sampling overlap between both periods: Appleton City and Rockville. Based on the 12 birds sampled in 1978–1980 (Sites 5, 9, 10, 13 and 14 in bottom map of Figure 1, Figure 2a, bottom), and the 10 birds sampled in 2016 (Sites 21, 24, 29, 30, 32, 33 and 36 in the top map of Figure 1, Figure 2a, top), the influence of Carolina genomes increased 27% through time at Appleton City (Hybrid Index where pure Carolina = 1.0, average 1978–1980 value = 0.46, average 2016 = 0.58, *p*‐value = .0315). This same result was also reflected in average structure genomic proportions (average assignment to the Carolina cluster in 1978–1980 sample = 39%; average assignment in 2016 sample = 73%; Mann–Whitney *U* test *p*‐value = .1377). Based on the 28 birds sampled in 1978–1980 (Sites 3, 4, 6, 7, 8, and 18 in bottom map of Figure 1, Figure 2a, bottom), and the 31 birds sampled in 2016 (Sites 10, 11, 13, 14, 16, 19, 20, 22, 23, 31, 34, 35, 37, 41, 43 and 45 in top map of Figure 1, Figure 2a, top), the influence of Carolina genomes increased by 26% through time at Rockville (average 1978–1980 Hybrid Index value = 0.43, average 2016 = 0.54, *p*‐value = .004). This result was again reflected in the average structure assignments (average assignment to the Carolina cluster in 1978–1980 sample = 34%; average assignment in 2016 sample = 68%; *p*‐value = .01062; assuming unequal variance between samples).

### 3.3 Limitations of hybrid zone width assessment

When examining the structure assignment of the 1978–1980 birds characterized with ddRADseq, the contact zone appeared to extend further northwest than originally defined based on vocalizations, plumage morphology, and allozyme data (Robbins et al., 1986). For example, based on those data sets, Site 4 in the 1980 sample (bottom panel of Figure 1, equivalent to Robbins et al., 1986 Site 2) was considered outside the hybrid zone, falling in an area where only black‐capped chickadees were thought to occur. However, structure analyses inferred that five of 12 birds collected at this site were hybrids (defined as having ≤95% of their genome assigning to any given parental species cluster), with the remainder classified as black‐capped chickadees (Table S1). In contrast to these genetic results, only black‐capped vocalizations were heard and recorded at that site in 1980 (Robbins et al., 1986).

In addition to the proposed repositioning of the 1978–1980 hybrid zone based on genetic data, spatial interpolation of structure assignment of birds from the 2016 sample suggested that the current hybrid zone extends to the northwest of our dense spatial sampling regime (e.g., failure to observe dark red contour; Figure 2a, top). For this reason, we focused our hybrid zone movement analyses on the position of the black‐capped/Carolina chickadee interface as inferred through tess3r and geographic cline analyses, and do not comment on changes in the potential extent of hybridization (i.e., hybrid zone width) across this zone through time, including differences in hybrid zone width for putative loci involved in reproductive isolation.

### 3.4 Variation in patterns of introgression by locus

**FIGURE 4:**
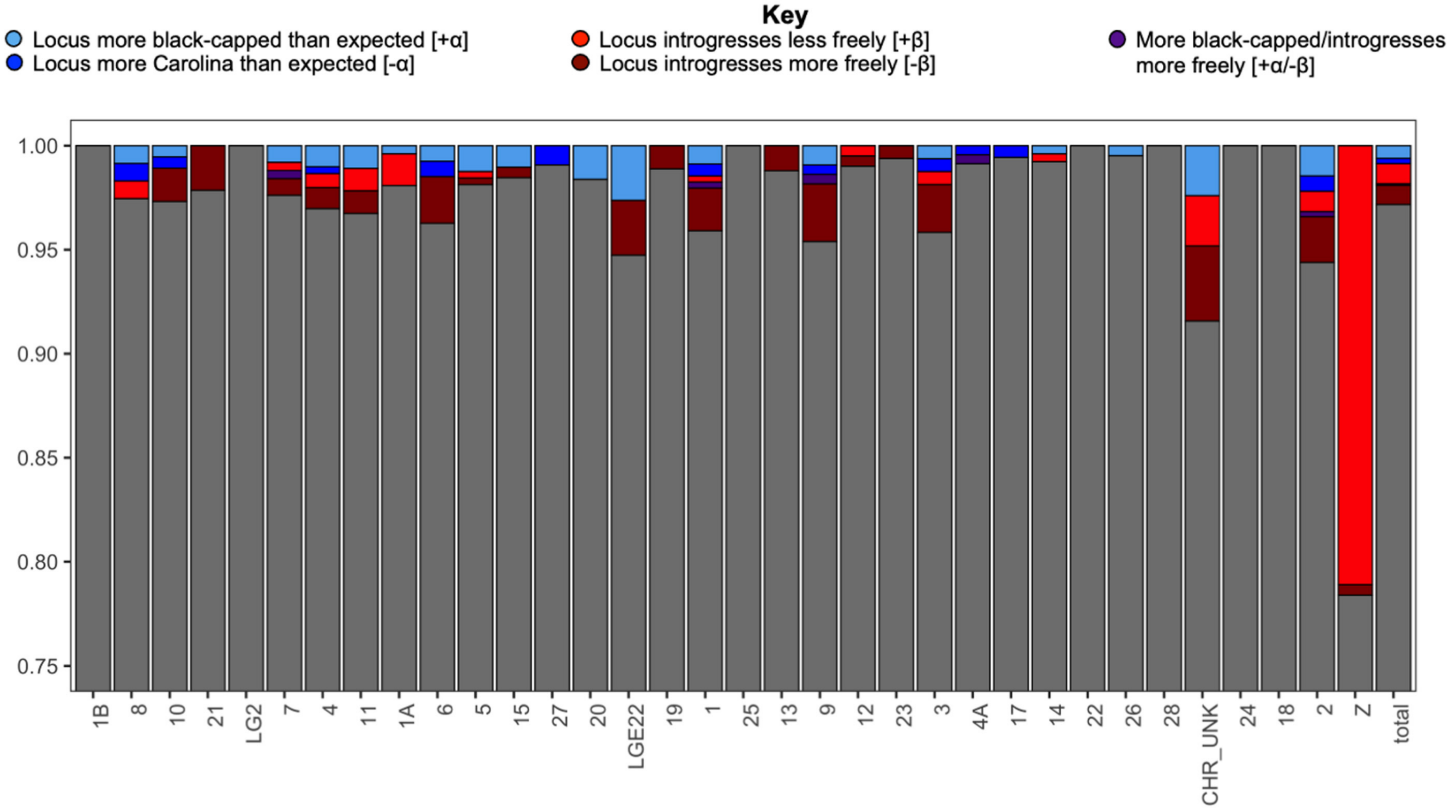
Proportion of outlying loci categories (as identified by bgc) for each chromosome. Chromosomes ordered by G‐test statistic on whether their outlier loci composition differed significantly from the background total genome composition (which is shown on the far right). Ordered from left (not significantly different to background genome composition) to right (chromosome 14 and all chromosome/scaffolds to the right of it were significantly different from the background genome composition at alpha = 0.05). Nonoutlying loci are indicated in grey and comprised the remainder of loci not shown for each chromosome. Specific values for the numbers of loci in each outlier category by chromosome are available at https://github.com/laninsky/chickadees/blob/master/output/outlier_by_chrom.csv.

Although we acknowledge the limitations of using RADseq markers to detect selection given limitations in marker density relative to blocks of linkage disequilibrium (Lowry et al., 2017), we conducted a genomic cline analysis in an attempt to identify loci showing restricted movement across the hybrid interface using bgc. Based on inspection of the bgc chains, we removed an additional 1500 states, as well as the defined burnin, before confirming convergence. Of the 6748 loci included in this analysis, 191 outlier loci (2.8% of total loci) were identified (Table S5A; Figure S5A). Outliers were classified as a locus being “more black‐capped” than expected based on genomic background (+*α*: 0.68% of total loci), “more Carolina” than expected based on genomic background (−*α*: 0.25% of total loci), less capable of introgressing across the hybrid zone (+*β*: 0.98% of total loci), more capable of introgressing across the hybrid zone (−*β*: 0.99% of total loci), and combinations of these categories (Table S5A; Figure S5A). These outlier categories were not distributed evenly across the chromosomes (Figure 4). The five “chromosomes” most distinct from the underlying distribution shown by the total genome (Figure 4) were Chromosome Z, 2, 18, 24, and unplaced scaffolds (“CHR_UNK”). Chromosomes 18 and 24 had significantly fewer outlying loci compared to the genomic background. Chromosomes 2 and the unplaced scaffolds had a larger percentage of loci across multiple outlier categories. Chromosome Z showed a very distinctive pattern, with a large excess of loci that appear to introgress less freely (+*β*), even after accounting for the total number of loci mapping to this chromosome (Figure S5B).

For the remainder of our analyses, we focused on significant positive β outliers as regions of the genome potentially involved in reproductive isolation, including comparing to outliers identified by Wagner et al. (2020), who reanalysed RADseq data from Pennsylvania (Taylor, Curry, et al., 2014; Taylor, White, et al., 2014) using a reference black‐capped chickadee genome. Most of our positive β outliers (36 of 66 loci) were <25 kbp from black‐capped CDS regions (Table S5B). However, this proportion was lower than that of the outlying loci identified by Wagner et al. (2020) (452 of 470, Fisher's exact test, *p* < .0001), potentially owing to the different restriction enzymes used influencing the targeted regions of the genome (*SbfI/MspI* in our study, *PstI* in Taylor, Curry, et al., 2014; Taylor, White, et al., 2014; Wagner, et al., 2020), and/or the ability of Wagner et al. (2020) to use the annotations that they developed for the genome rather than the CDS mapping approach we performed. Among the 49 CDS regions represented across the 36 positive β outliers within 25 kbp of a gene (some SNPs were associated with more than one gene), we found no significant enrichment for GO terms. None of the 13 genes associated with the outlier loci of Taylor, Curry, et al. (2014) were identified in our current analyses and none of our 66 positive β outlier loci was <25 kbp of any of the 1850 loci identified as outlying by Wagner et al. (2020). We then searched for stretches of consecutive significant positive β loci (potentially indicative of inversions/regions of reduced recombination), finding these only for chromosome Z (two total regions) (Table S5B; Figure S5C). No significant enrichment for GO terms was found for either of these regions, or in combination.

### 3.5 Correlation of hybrid zone movement with climate change

Additional detail on quality control of the climate data can be found in the Supporting Information Results and Figure S6. However, over the longer‐term contrast, Pennsylvania has warmed ~50% more than Missouri (Figure 3, Figure S7A), correlating with the different rates of movement of the chickadee hybrid zone in each of these areas. This warming is strongly evident when plotting the rates of change within 50 km of the Missouri and Pennsylvania transects (Figure S8). In terms of precipitation, Missouri has become wetter, whereas Pennsylvania has not changed (Figure S7B).

## 4 DISCUSSION

Using a 38‐year temporal comparison, we demonstrated northwest movement of the black‐capped and Carolina chickadee hybrid zone in Missouri between 1978–1980 and 2016. The movement of this zone, in context of the results from other studies at the eastern end of this contact zone, appears to be consistent with contrasts in the degree of climate change (Bronson, Grubb, Sattler, et al., 2003; Harr & Price, 2014; Taylor, White, et al., 2014). However, we failed to identify pathways or genes potentially involved in reproductive isolation across the entire length of the chickadee hybrid zone.

### 4.1 Movement of the black‐capped and Carolina chickadee hybrid zone

Despite detecting a temporal movement of the hybrid zone, our results indicate that the zone in west‐central Missouri has not moved at the same pace during the past 36–38 years as in the eastern portion of the chickadee contact zone in southeastern Pennsylvania and Ohio (Bronson et al., 2005; Bronson, Grubb, Sattler, et al., 2003; Taylor, White, et al., 2014; Wagner et al., 2020). Even at the fastest potential pace suggested by our data – assuming that the zone moved from northwest of Rockville to the Pleasant Gap area (sampled only in 2016; Sites 6–9 top map of Figure 1)—the distance is only 8–9 km over 36–38 years (~0.2 km/year), well below the documented rates in the eastern areas of 1.2 km/year (Pennsylvania: Harr & Price, 2014; Taylor, White, et al., 2014) and 1.6 km/year (Ohio: Bronson, Grubb, Sattler, et al., 2003).

Analysing temperature trends across the region over the last 38 years, we found that eastern areas have warmed 50% more than the Osage Plains and surrounding areas in southwestern Missouri. Our climate data analysis also suggests little movement of the Illinois hybrid zone is expected, consistent with the stability of chickadee song types in this area (Enstrom & Bollinger, 2009). However, given the issues with song data, genetic data are needed to clarify the rate of movement of the Illinois hybrid zone.

However, even though climate is probably important, other factors may influence the movement and width of the hybrid zone. Despite being on average smaller (Rising, 1968), male Carolina chickadees tend to be dominant in heterospecific interactions, and females of both species appear to show a preference for them (Bronson, Grubb, Sattler, et al., 2003), particularly as extrapair partners (Reudink et al., 2006) and observations suggest that assortative mating of “black‐capped‐like” and “Carolina‐like” birds is not occurring within the hybrid zone (Robbins et al., 1986). Also, studies have documented no consistent differences in habitat preferences between parental species other than elevation in sky island populations of black‐capped chickadees in the Appalachians (Johnston, 1971).

Despite the documented movement of the hybrid zone, the proportion of individuals identified as hybrids (based on admixture and heterozygosity) remained relatively constant across our sampling periods (19% vs. 25%, *t* test *p*‐value = .679). Although this finding needs to be interpreted with caution due to the previously stated limitations for characterizing the width of the hybrid zone, this is consistent with strong selection against hybrids as demonstrated previously in eastern areas of the hybrid zone (Bronson et al., 2005; Bronson, Grubb, & Braun, 2003; McQuillan et al., 2018; Olson et al., 2010).

### 4.2 Genetic architecture of the black‐capped and Carolina chickadee hybrid zone

We compared the genomic location of the outlying loci identified in our Missouri transect with the previous studies of Taylor, Curry, et al. (2014) and Wagner et al. (2020), who examined birds from the Pennsylvania hybrid zone (Wagner et al., 2020 reanalysed the data of Taylor, Curry, et al., 2014; Taylor, White, et al., 2014 using a reference genome, so we focus on comparing to the reference‐based results here). Broadly (i.e., at chromosomal level), our results were very similar. The chromosome that contained the largest number of loci significantly resistant to introgression (i.e., positive β outliers) in our study was chromosome Z. This chromosome also had tracts of consecutive positive β outliers, potentially indicative of inversions/regions of reduced recombination. Wagner et al. (2020) found similar results, and the importance of chromosome Z in both studies is consistent with reduced introgression due to Haldane's rule and the large X(Z) effect (Irwin, 2018; Runemark et al., 2018).

However, at a finer scale, we were unable to detect overlapping outlying regions between our study of the Missouri transect and the outliers identified by either Taylor, Curry, et al. (2014) or Wagner et al. (2020) in the Pennsylvania transect. This outcome is not inconsistent with results from at least some other hybrid zones where multiple transects have been sampled (Table 1). However, like previous studies that examined patterns of introgression of specific genes between different geographic transects of the same hybrid system, we used reduced representation sequencing (Table 1). Given the limitations of reduced representation sequencing for detecting underlying loci under selection, it is likely that these studies, including our own, are underestimating the number of regions resistant to introgression that are concordant between different transects (Janoušek et al., 2012; Lowry et al., 2017). In addition, variation in laboratory methodology (e.g. restriction enzyme choice) and recombination landscapes among geographic locations (e.g., Nelson et al., 2019) could further impact the ability to identify underlying regions resistant to selection that are concordant among locations. Examining consistency across multiple hybrid‐zone transects of introgression patterns using whole genome resequencing data will allow the field to use quantitative assessments of the proportion of shared versus unique loci, rather than the somewhat subjective assessments currently captured in Table 1 (e.g., the column “Patterns of introgression across different transects”). The use of whole genome sequencing will also allow comparison across different hybrid systems of the factors influencing consistency between multiple transects, including the influence of local population ancestry or selective pressures on the outcome of introgression across hybrid zones (Gompert et al., 2017; Harrison & Larson, 2016; Teeter et al., 2010). However, even with whole genome sequencing, where the loci under selection are targeted directly, the detection rate of loci resistant to introgression will not be 100% (Gompert & Buerkle, 2011).

**TABLE 1:** Summary of studies that have compared locus‐specific patterns of introgression at multiple geographic transects for a given hybrid zone system, ordered by taxa.

Species system	Taxa	Method of identifying introgression outliers	Patterns of introgression across different transects	Subdivisions in taxa examined between transects	Natural hybrid zone	Marker type	Reference
*Helianthus annuus* and *H. petiolaris*	Plant	Frequency of individuals who had “*petiolaris*” band	**“Striking congruence of marker introgression patterns between widely separated hybrid zones in Nebraska and southern California”**	Yes, morphological differences	No^a^	RAPD markers (*n* = 61)	Buerkle and Rieseberg (2001)
*Pinus contorta* and *P. banksiana*	Plant	Gompert and Buerkle (2009, 2010)	**“Patterns of introgression were more similar between the zones than expected by chance, but there were significant differences between these regions at specific loci”**	No	Yes	SNPs (*n* = 29)	Burns et al. (2019)
*Gryllus pennsylvanicus* and *G. firmus*	Invertebrate	Gompert and Buerkle (2009)	**“Consistent patterns of introgression for individual loci”**	No	Yes	Sequenom MassARRAY (*n* = 110 SNPs)	Larson et al. (2014)
lineages of *Tigriopus californicus*	Invertebrate	Gompert and Buerkle (2009, 2010)	**“We observe blocks of linked markers with similar introgression patterns”**	No	Yes^b^	Sequenom MassARRAY (*n* = 54 SNPs)	Pritchard and Edmands (2013)
*Cottus perifretum* and *C. rhenanus*	Fish	Gompert and Buerkle (2009)	“Patterns observed at individual loci show little correlation between zones”	No	No^c^	Microsatellites (*n* = 168)	Nolte et al. (2009)
*Bufo* and *B. spinosus*	Amphibian	Gompert and Buerkle (2011, 2012)	**“Twenty‐six barrier markers are shared between transects […] which is more than would be expected by chance.”**	Genetic substructure within *B. bufo*	Yes	3RAD (*n* = 10,535 to 39,750 SNPs)	van Riemsdijk et al. (2020)
*Lissotriton montandoni* and *L. vulgaris*	Amphibian	Gompert and Buerkle (2011, 2012)	“We found limited overlap of cline outliers between transects”	Two lineages of *L. vulgaris*	Yes	Molecular inversion probes (*n* = 1233 loci)	Zieliński et al. (2019)
lineages of *Podarcis muralis*	Reptile	Gompert and Buerkle (2011, 2012)	“Putative barrier loci were enriched in genomic regions that were highly differentiated between the two lineages and showed low concordance between the transects. The exception was a consistently low genetic exchange around ATXN1, a gene that modulates social behaviour”	No (population structure present, but paired across transects)	Yes	ddRADseq SNPs (*n* = 1029)	Yang et al. (2020)
*Pipilo maculatus* and *P. ocai*	Bird	Gompert and Buerkle (2011)	**“Results are consistent with a history in which reproductive isolation has been influenced by a common set of loci in both hybrid zones, but where local environmental and stochastic factors also lead to genomic differentiation”**	Population structure within *P. ocai*	Yes	GBS (*n* = 41,000 SNPs)	Kingston et al. (2017)
*Poecile atricapillus* and *P. carolinensis*	Bird	Gompert and Buerkle (2011, 2012)	No overlapping loci found	No	Yes	GBS/RADseq, with different enzymes between studies (this study, *n* = 6784 SNPs; Wagner et al., 2020: *n* = 76,883 SNPs)	This study; Taylor, Curry, et al. (2014), Wagner et al. (2020)
*Mus domesticus* and *M. musculu*s	Mammal	Gompert and Buerkle (2009, 2010)	“Different patterns of introgression in the two transects highlight the challenge of using hybrid zones to identify genes underlying isolation and raise the possibility that the genetic basis of isolation between these species may be dependent on the local population genetic make‐up or the local ecological setting”	No	Yes	TaqMan probes (*n* = 41 SNPs)	Teeter et al. (2010)
*Mus domesticus* and *M. musculu*s	Mammal	Gompert and Buerkle (2009, 2010)	“Markers shared between transects is a relatively small subset of the markers identified in the two transects separately”	No	Yes	*n* = 1401 SNPs	Janoušek et al. (2012)
*Mus domesticus* and *M. musculu*s	Mammal	Gompert and Buerkle (2009, 2010)	**“There is some evidence of common architecture of reproductive isolation.”**	No	Yes	PCR (*n* = 24 X‐chromosome markers)	Macholán et al. (2011)

*Note*: Studies where patterns of introgression across different transects are largely consistent/congruent, have their entry for this column in bold. Potential factors that may influence the recovery of consistent introgression patterns are also given (method for identifying introgression outliers, subdivisions between transects, and whether the hybrid zone is natural or human‐mediated e.g., Kane et al., 2009).

^a^ *H. petiolaris* introduced to California from Great Plains, however, *H. annus* and *H. petiolaris* occur in sympatry in the Great Plains.

^b^ Mimicked with laboratory crosses.

^c^ *C. perifretum* is considered invasive.

This broad comparison across species (Table 1) suggests a need to standardize laboratory methodology (i.e., whole genome sequencing), the method of identifying outliers, and the threshold for deciding whether concordant patterns of introgression have been found between transects, before it can be concluded that variation in patterns of introgression could impact differential speed of movement of the chickadee hybrid zone. Focusing on transcriptomes and/or methylomes will also be important in identifying other (epi)genetic mechanisms that impact on hybrid performance, as not all adaptation/dysregulation due to hybridization is likely to be reflected in genomic sequence (Moran et al., 2020). An additional future avenue of research will be examining the degree to which the microbiome influences the reduced fitness of hybrids, as observed in hybrid zones of other species (Wang et al., 2015). However, currently, variation in climate is the most parsimonious explanation for the differences observed between Missouri and Pennsylvania.

## 5 CONCLUSION

Comparison of levels of admixture in contemporary and historical samples is a powerful method of documenting the impact of climate change and other anthropogenic pressures. Using museum samples, we documented movement of the black‐capped and Carolina chickadee hybrid zone in Missouri. Our results contrast with those from a study of the eastern portion of the zone, in Pennsylvania, where the rate of movement was faster. Human‐caused climate change has influenced distributions and abundances of species, and is probably elevating the probability of extinction for many taxa (Thomas et al., 2004). Although it can be tempting to make broad characterizations about how climate change will affect species with large distributions, geographic variation in hybrid zone movement rates suggests that the specific impacts on broadly distributed species will need to be assessed at both local and regional scales. As climate change phenomena continue to manifest, detailed characterization of their variation will be key in assembling a predictive view of their implications, with museum collections critical in this endeavour (Billerman et al., 2019; Lopez et al., 2020; Ryan et al., 2018; Schmitt et al., 2018).

## AUTHOR CONTRIBUTIONS

Mark B. Robbins conceptualized study and carried out fieldwork. Alana Alexander, Mark B. Robbins, and Jesse Holmes carried out laboratory work. Alana Alexander, A. Townsend Peterson, and Mark B. Robbins carried out analyses. Alana Alexander and A. Townsend Peterson visualized the results. Alana Alexander and Mark B. Robbins were responsible for data curation and wrote the manuscript. All authors reviewed and edited the manuscript. Mark B. Robbins, Alana Alexander, Robert G. Moyle and A. Townsend Peterson acquired or provided funding.

## CONFLICTS OF INTEREST

The authors have no conflicts of interest to declare.

## BENEFIT‐SHARING STATEMENT

Benefits from this research accrue from the sharing of our data, methods (i.e., code), and results on public databases as described above. A lay summary of the results has also been provided to the Kaskaskia (Peoria) and Osage peoples as traditional custodians of the area the study was conducted in (also available at https://github.com/laninsky/chickadees).

## Supporting information


Appendix S1

## Data Availability

Demultiplexed sequence data for each individual have been deposited in the NCBI SRA (BioProject no: PRJNA881039). All other data are available in the main text, the Supporting Information material, dryad (https://doi.org/10.5061/dryad.k98sf7m92) and/or at https://github.com/laninsky/chickadees (repository at 25 September 2022 corresponds to the version of scripts used in this manuscript, 34f868b).
