# Supplementary material for: Limited movement of an avian hybrid zone in relation to regional variation in magnitude of climate change
Source: Mol Ecol. 2022 Oct 21;31(24):6634–48. doi: 10.1111/mec.16727 (PMC9729445; doi:10.1111/mec.16727)
Supplement: Supplementary file 1 — Appendix S1 [file MEC-31-6634-s001.docx]

**Supplemental Information for:**

Limited movement of an avian hybrid zone in relation to regional variation in magnitude of climate change

Alana Alexander^†*^, Mark B. Robbins^†^, Jesse Holmes, Robert G. Moyle, and A. Townsend Peterson

^†^These authors contributed equally to this work.

*Corresponding author. Email: alana.alexander@otago.ac.nz

Table of Contents:

Supplementary methods [Page 2](#_Fig._S1.)

Supplementary results [Page 4](#_Supplementary_Results)

Fig. S1 [Page 6](#_Fig._S3)

Fig. S2 [Page 7](#_Fig._S9)

Fig. S3 [Page 9](#_Fig._S5)

Fig. S4 [Page 10](#_Fig._S6)

Fig. S5 [Page 11](#_Fig._S7)

Fig. S6 [Page 22](#_Fig._S12.)

Fig. S7 [Page 23](#_Fig._S13.)

Fig. S8 [Page 24](#_Fig._S14.)

Table S1 [Page 25](#_Table_S1)

Table S2 [Page 29](#_Table_S2)

Table S3 [Page 32](#_Table_S3)

Table S4 [Page 33](#_Table_S4)

Table S5 [Page 40](#_Table_S6.)

References [Page 42](#_References)

# Supplementary Methods

***Laboratory methods for ddRADseq***

DNA (500 ng) for each sample (except for two pools of 16 samples where 250 ng was used as input) was digested with 20 U each of *SbfI*-HF and *MspI*, and 1× CutSmart buffer (New England Biolabs®: NEB), made up to a total volume of 50 μL with PCR-grade H_2_O. A ¼ reaction was run with Lambda DNA (NEB) for each pool of chickadee samples as a positive control. Following digestion for at least five hours at 37°C, samples were purified with 1.5× volume of Agencourt® AMPure XP® beads (Beckman Coulter), using two washes of 200 μL fresh, cold 70% ethanol. Following the final wash, samples were eluted in 40 μL PCR-grade H_2_O and quantified using Qubit*^TM^* (Invitrogen).

To ligate adaptors, we set up reactions with up to 32 μL of cleaned, digested sample (standardized to the sample with the lowest concentration within the pool); 100 nM of sample-specific “P1” *SbfI* cut-site adaptor (with an internal barcode; **Table S2**); 1 μM of “P2” *MspI* cut-site adaptor (not sample-specific; **Table S2**); 400 U of T4 DNA ligase (NEB); and 1× T4 DNA ligase buffer (NEB), made up to a total volume of 40 μL with PCR-grade H_2_O. We also set up ligation reactions for our positive lambda controls. Samples were incubated at 23°C for 1 hour, heat killed at 65°C for 10 min, and then cooled by 2°C every 90 s until reaching room temperature (20°C). Following ligation, samples with unique P1 adaptors were pooled (8-16 per pool) and purified with 1.5× volume of Agencourt® AMPure XP® beads using two washes of 200 μL of fresh, cold 70% ethanol. Following the final wash, samples were eluted in 50 μL PCR-grade H_2_O, and a second round of purification carried out with 1.5× volume of Agencourt® AMPure XP® beads, using two washes with 200 μL of fresh, cold 70% ethanol. Following this final wash, pools were eluted in 35 μL of Buffer EB (QIAGEN). This process was conducted separately for our positive lambda controls.

To confirm that digestion and adaptor ligation were successful for our samples, we set up a test PCR with 400 nM each of “common” Primer 1 and pool-specific Primer 2 (**Table S2**), 1× Phusion® High-Fidelity PCR Master Mix with HF Buffer (NEB) and 1.5 μL of cleaned, pooled, post-ligation product, made up to a total volume of 25 μL with PCR-grade H_2_O. An initial denaturation step of 98°C for 30 s was followed by 11 cycles of 98°C for 10 s, 65°C for 30 s, 72°C for 60 s, followed by a final extension of 72°C for 10 min. This test PCR was also conducted separately for our positive lambda controls. Cleaned post-ligation products and test PCR products for our sample pools/positive lambda controls were run against undigested lambda DNA and 100 bp DNA ladder (Promega) for reference. Following successful digestion and adaptor ligation, no high-molecular weight crowns were observed, and post-ligation reactions were broad smears, potentially with some laddering for our pooled samples. PCR reactions produced narrower and brighter (than the post-ligation) smears for the sample pools. For the positive lambda control, fragments of the following size were expected following the test PCR: 154, 199, 495, 558, 1610 bp.

After confirming that the pools represented samples that were successfully digested and had adaptors ligated, fragments between 200-500 bp were selected using a 2% DNA Gel Cassette with Internal V1 marker on a BluePippin (Sage Science) following the manufacturer instructions. Size-selected sample pools were quantified with Qubit (the optional step of using Dynabeads® Invitrogen to select against fragments with P1 adaptors ligated to both ends was used successfully on only two pools before being dropped owing to difficulties with library loss during this step), before the final enrichment PCR that added pool-specific external barcodes. This PCR consisted of 400 nM each of “common” Primer 1 and pool-specific Primer 2 (**Table S2**), 1× Phusion® High-Fidelity PCR Master Mix with HF Buffer (NEB), and 21 μL of the size-selected sample pool, made up to a total volume of 50 μL with PCR-grade H_2_O. The thermoprofile used was the same as for the test PCR. The final enrichment PCR was then purified with 1.5× volume of Agencourt® AMPure XP® beads, using two washes of 200 μL fresh, cold 70% ethanol. Following this final wash, pools were eluted in 40 μL of Buffer EB and quantified using Qubit. [[back to *Contents*](#_top)]

# Supplementary Results

***Summary of genetic dataset***

The number of reads obtained across the initial test set of 8 samples ranged from 2,188,989 to 3,332,843 (mean = 2,760,725; s.e. = 145,973). After excluding one of the historical samples (catalog number: 99788; tissue number: 649257) that had extremely low sequencing coverage (8,855 reads in total), the number of reads among the remaining 156 samples ranged from 280,350 to 1,146,532 (mean = 663,743; s.e. = 11,303). This difference in sequencing coverage between the test and main samples was also reflected both in the number of clusters found in each individual (test mean = 36,065; s.e. = 1,924, vs. main mean = 25,210; s.e. = 356), and also in the number of loci found in the final data set for each bird (test mean = 10,211; s.e. = 30, vs. main mean = 9,750; s.e. = 62) (**Table S4**). Despite high levels of missing data in our ddRADseq data set (28.4%, **Fig. S1**), patterns of missing data were more consistent with variation in sequencing coverage than erosion of restriction enzyme sites as a function of phylogenetic distance (Eaton et al., 2017; Lee et al., 2018; Pante et al., 2015) (**Fig. S2**). [[back to *Contents*](#_top)]

***Correlation of hybrid zone movement with climate change***

Rate of temperature change was highly consistent between our study period of 1978-2014 and the study period of Taylor et al. (2014) of 2000-2010, with an *r*^2^ of 0.16 between the two time spans based on 10,000 random points distributed across the lower 48 states of the United States (**Fig. S6A**). In contrast, precipitation had far less consistency between sampling periods, with an *r*^2^ of 0.0019 (**Fig. S6B**). Given the low consistency of trends in the precipitation data, we focused our analyses on temperature. Comparing climatic trends between Missouri and Pennsylvania, we found that contrasts of climate based on the 10-year time period of the previous Pennsylvania study (Taylor et al., 2014) failed to detect climate warming at all in Missouri, although warming is indeed present in Missouri in the long-term 38-year contrast (**Fig. S7A**). [[back to *Contents*](#_top)]

# Fig. S1

Distribution of completeness across 8,056 SNPs used in the chickadee STRUCTURE analysis. **Fig. S1** generated using code presented at https://github.com/laninsky/chickadees [[back to *Contents*](#_top)]


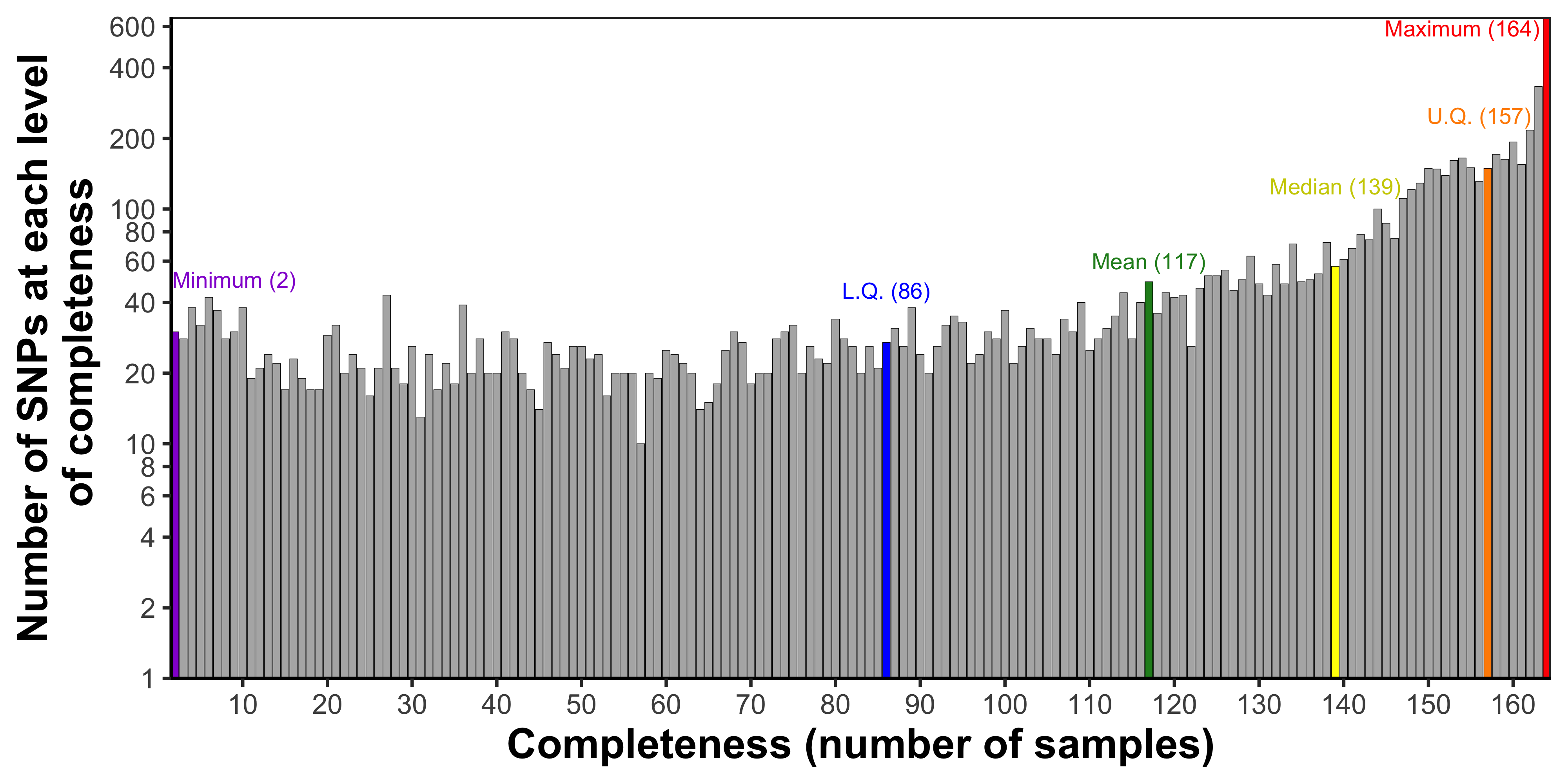


# Fig. S2

(next page)

The average number of loci recovered across the 13,366 pairwise comparisons between the *n* = 164 chickadees are shown by color within tiles. Bar graphs on the bottom and left of each plot show the numbers of pairs represented per tile. In (A) the genetic dissimilarity of samples within the pairs (inferred from STRUCTURE) is plotted on the horizontal axis, and whether the pairs of samples are on average more similar to black-capped or Carolina on the vertical axis (also inferred from STRUCTURE). If phylogenetic distance was driving the patterns of missingness in our data, we would expect greater numbers of overlapping loci (yellow vs blue) in tiles where both samples in a pair were similar to one of the parental species (e.g., the top and bottom left-hand side of the plot), which was not observed. In (B) dissimilarity is also on the horizontal axis, with average read-depth across both members of the pair on the vertical axis. If sequencing depth was driving patterns of missingness, we would expect greater numbers of overlapping loci between pairs of samples that both had higher sequencing coverage, which is what we observed. BC = black-capped chickadee, CC = Carolina chickadee. Code for generating components of these plots is given at https://github.com/laninsky/chickadees. [[back to *Contents*](#_top)]

(A)

(B)

# Fig. S3

Correlations between levels of missing data, STRUCTURE assignments to the black-capped chickadee genetic cluster, and PCA scores.

[[back to *Contents*](#_top)]

# Fig. S4

Spatial interpolations through tess3R (left) and geographic cline through HZAR (middle) based on normalized PC1 scores (similar patterns were observed in the spatial interpolations/geographic clines based on STRUCTURE assignments presented in **Fig. 2**), and heterozygosity results (right) for individuals from sampling sites where no equivalent sampling occurred during the other sampling period (top = Modern, bottom = Historical), coloured by STRUCTURE assignment (red = black-capped, blue = Carolina). [[back to *Contents*](#_top)]

# Fig. S5

Supplemental results from BGC genomic cline analyses. Outlier loci fall into the following categories: +α: an increase in the probability of black-capped ancestry in comparison to that predicted by hybrid index (i.e. more black-capped than expected) shown in light blue; -α: an increase in the probability of Carolina ancestry in comparison to that predicted by the hybrid index (i.e. more Carolina than expected) shown in dark blue; +β indicates excess ancestry‐based linkage disequilibrium (i.e. locus-specific ancestry restricted to matching genomic background, potentially indicating loci that are less free to introgress across the hybrid zone) shown in light red; -β indicates that locus-specific ancestry is less strongly associated with genomic background than in other loci (i.e. the loci are more free to introgress) shown in dark red. Significant combinations of α and β are shown as dark purple (significantly +α and -β: loci are more black-capped than expected, but also introgress more than expected). **(A)** Depiction of genomic cline, with average genomic background (“hybrid index”) ranging from 0 (Carolina) to 1 (black-capped), and locus-specific ancestry ranging from 0 (Carolina) to 1 (black-capped); **(B)** The relatively large number of positive β outliers on Chromosome Z shown in **Fig. 4** are not due to a large number of SNPs being found on this chromosome. **(C)** Median α (left) and β (right) plotted for loci relative to their genomic location on each chromosome/scaffold, with outliers marked by circles. No markers are present on Chromosome 16 due to the short amount of sequence available (1,017 bp: <https://www.ncbi.nlm.nih.gov/nuccore/CM022165.1>). Code for generating components of these plots is given at https://github.com/laninsky/chickadees [[back to *Contents*](#_top)]


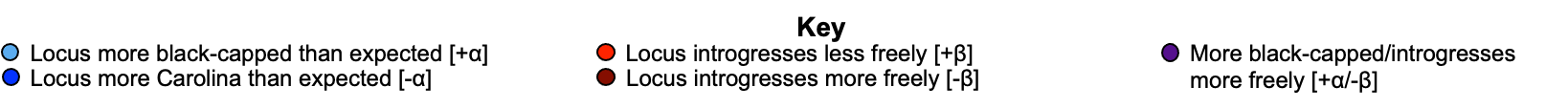


**(A)**

**
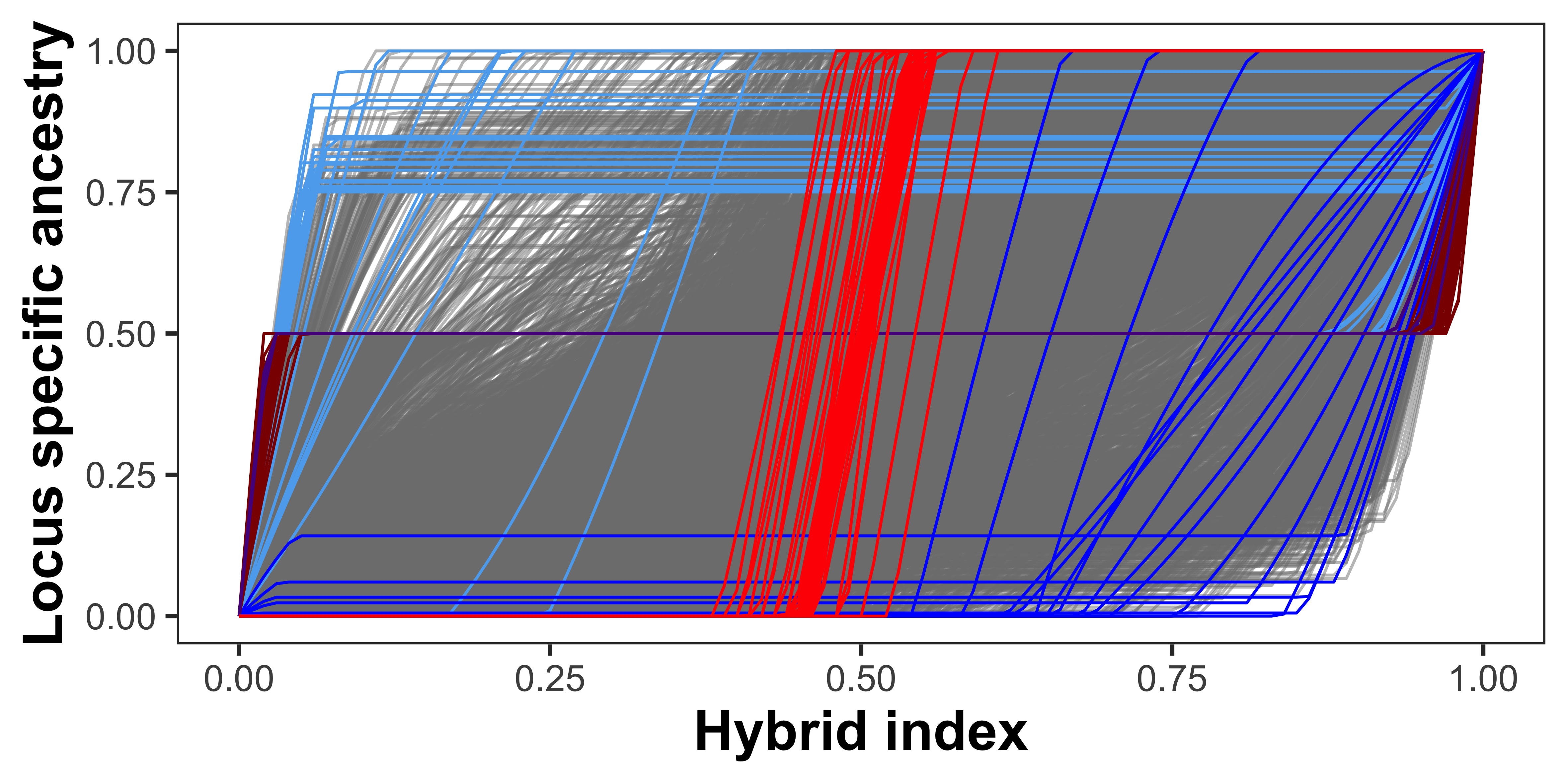
**

**(B)**

**
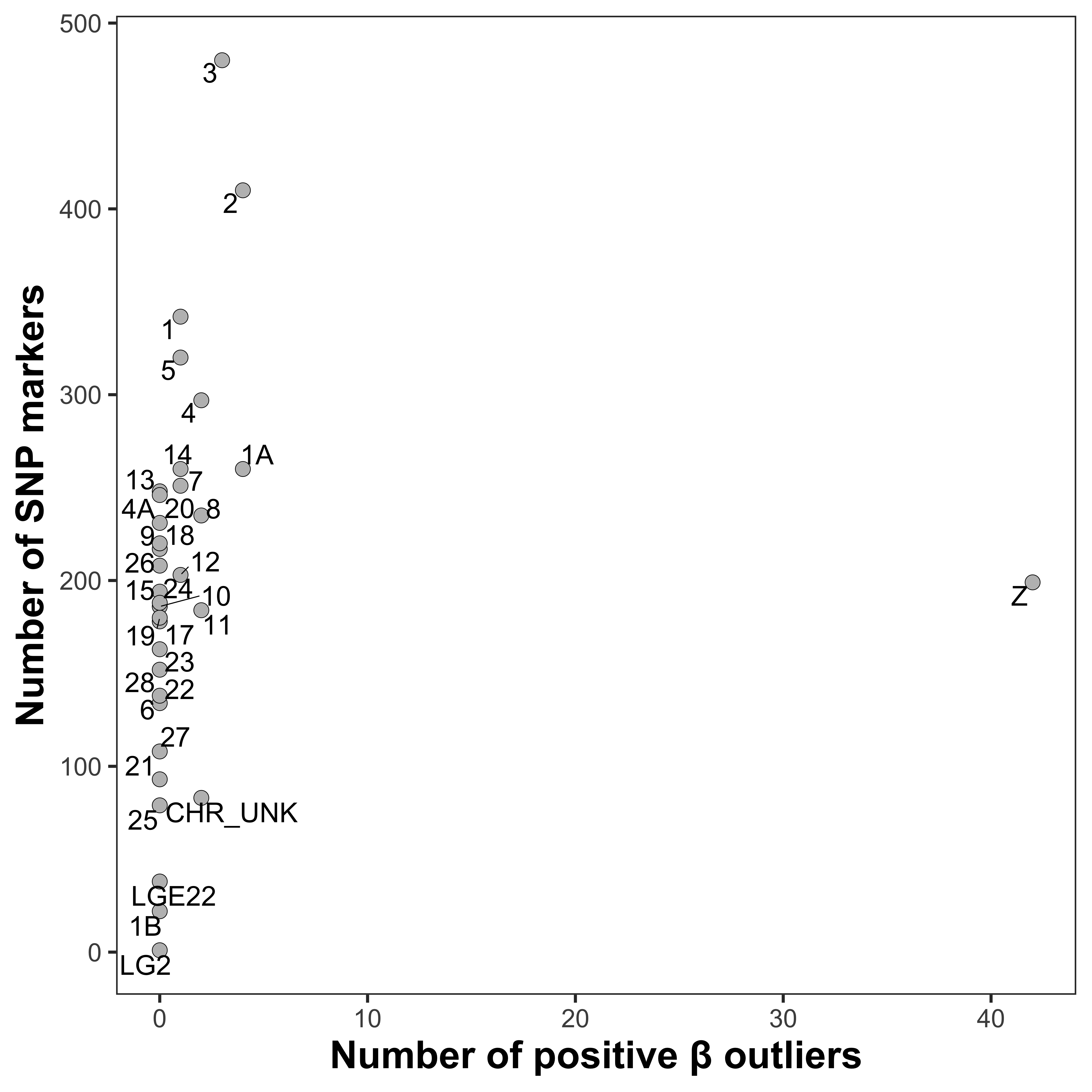
**


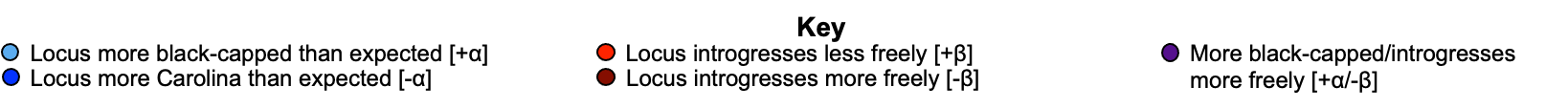
**(C)**

**Chromosome 1:** JAAMOC010000003.1 not displayed as only one RAD marker present

**
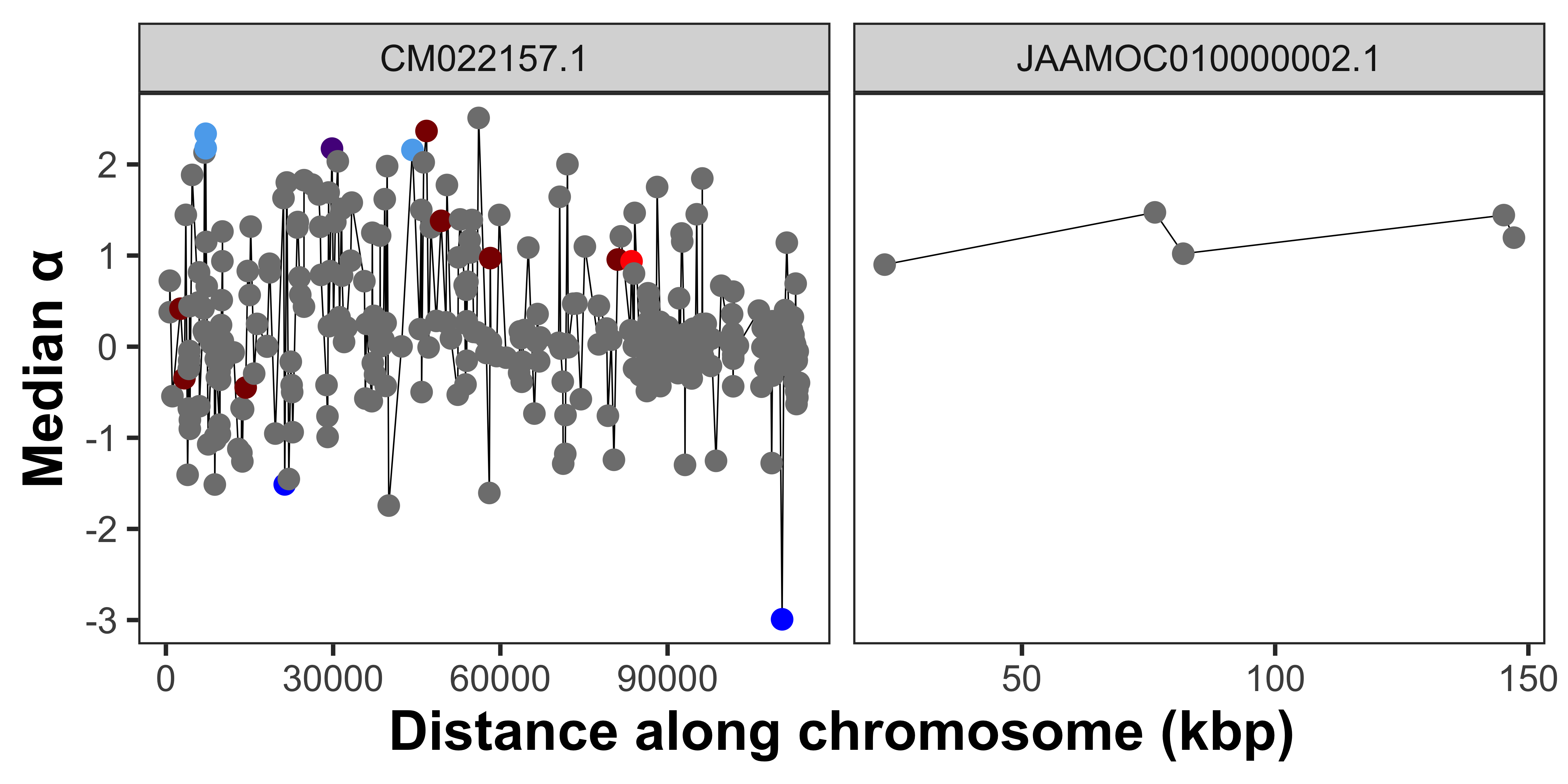

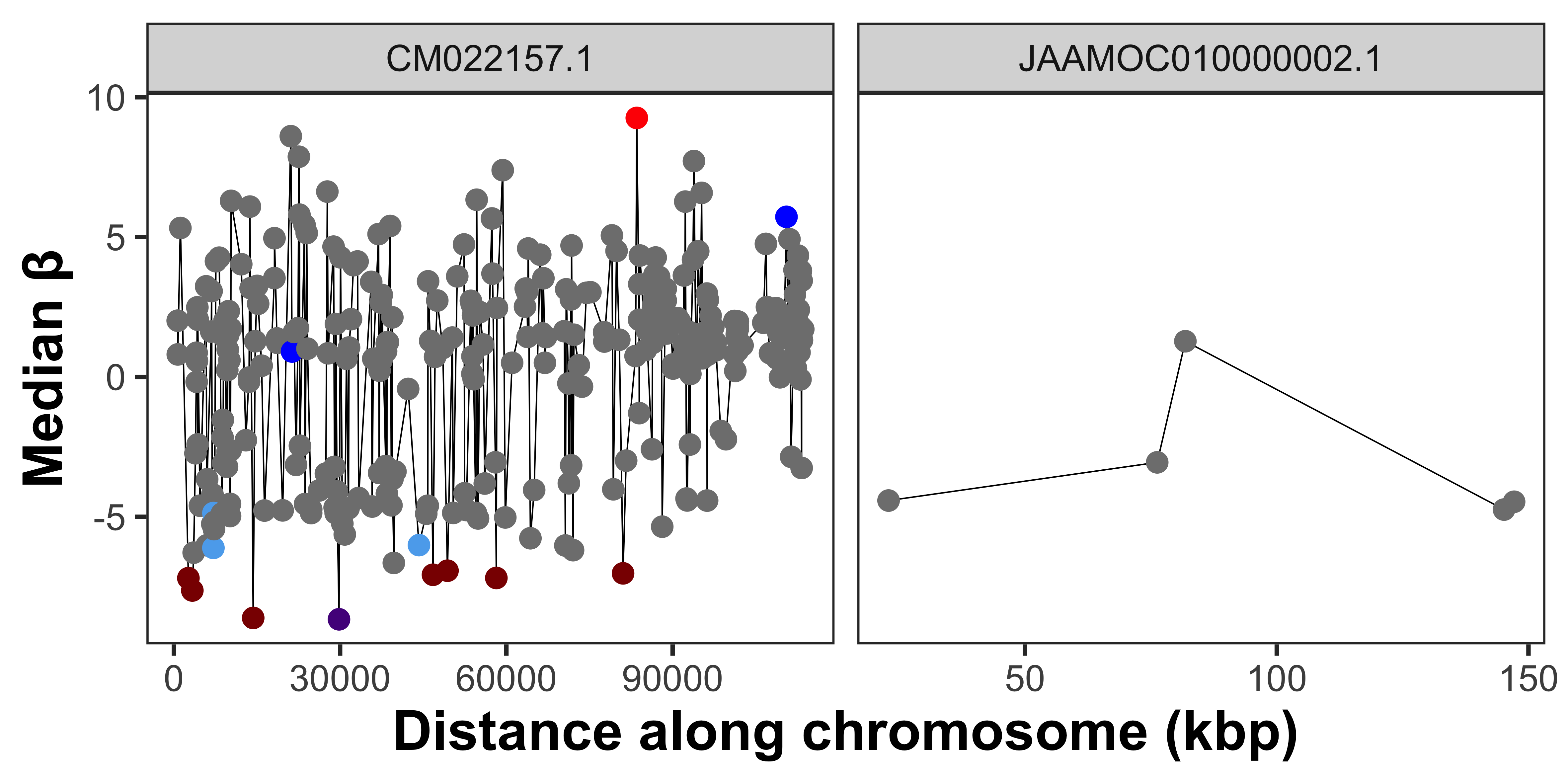
**

**Chromosome 1A:** JAAMOC010000546.1 not displayed as only one RAD marker present

**
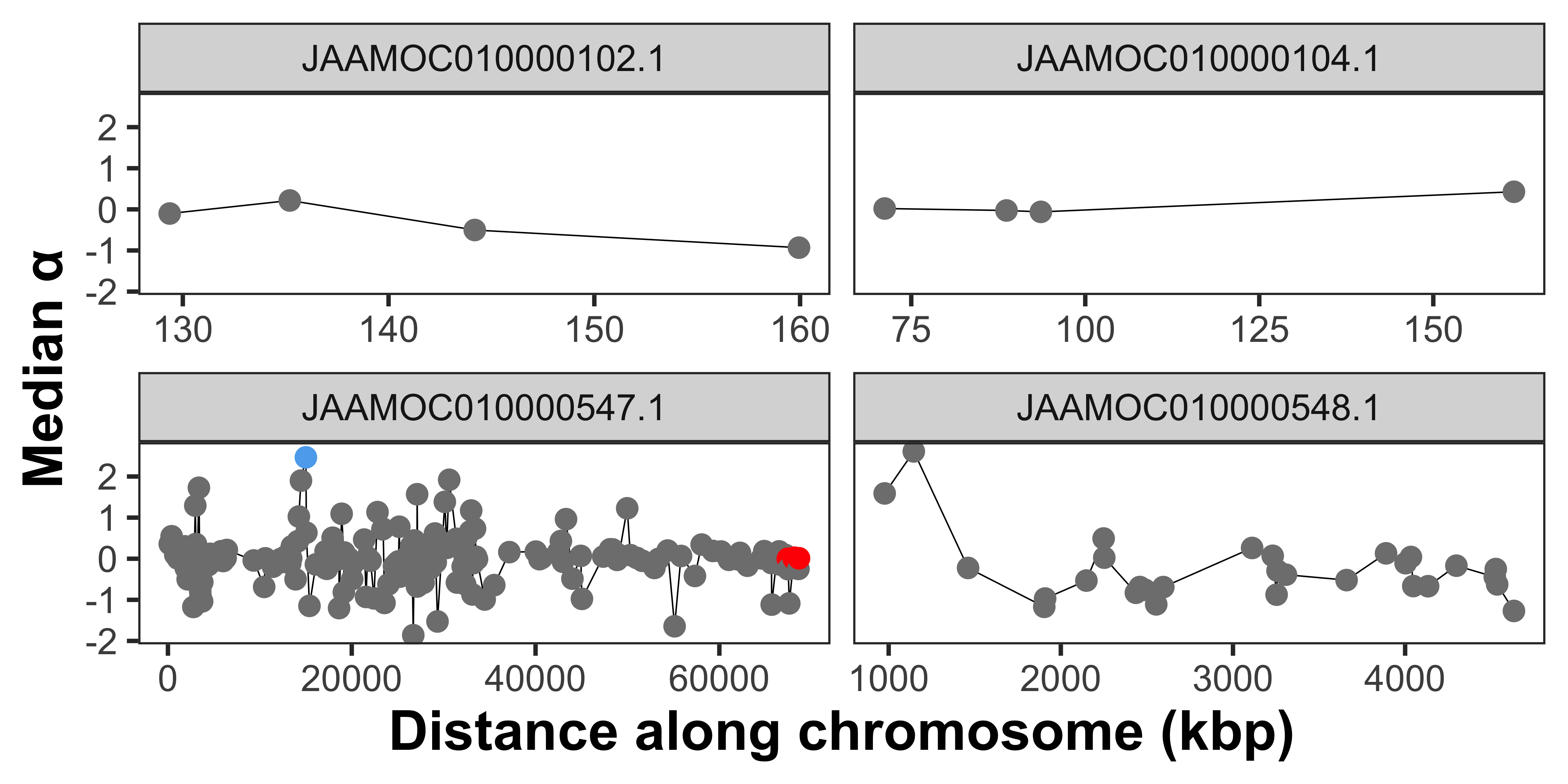

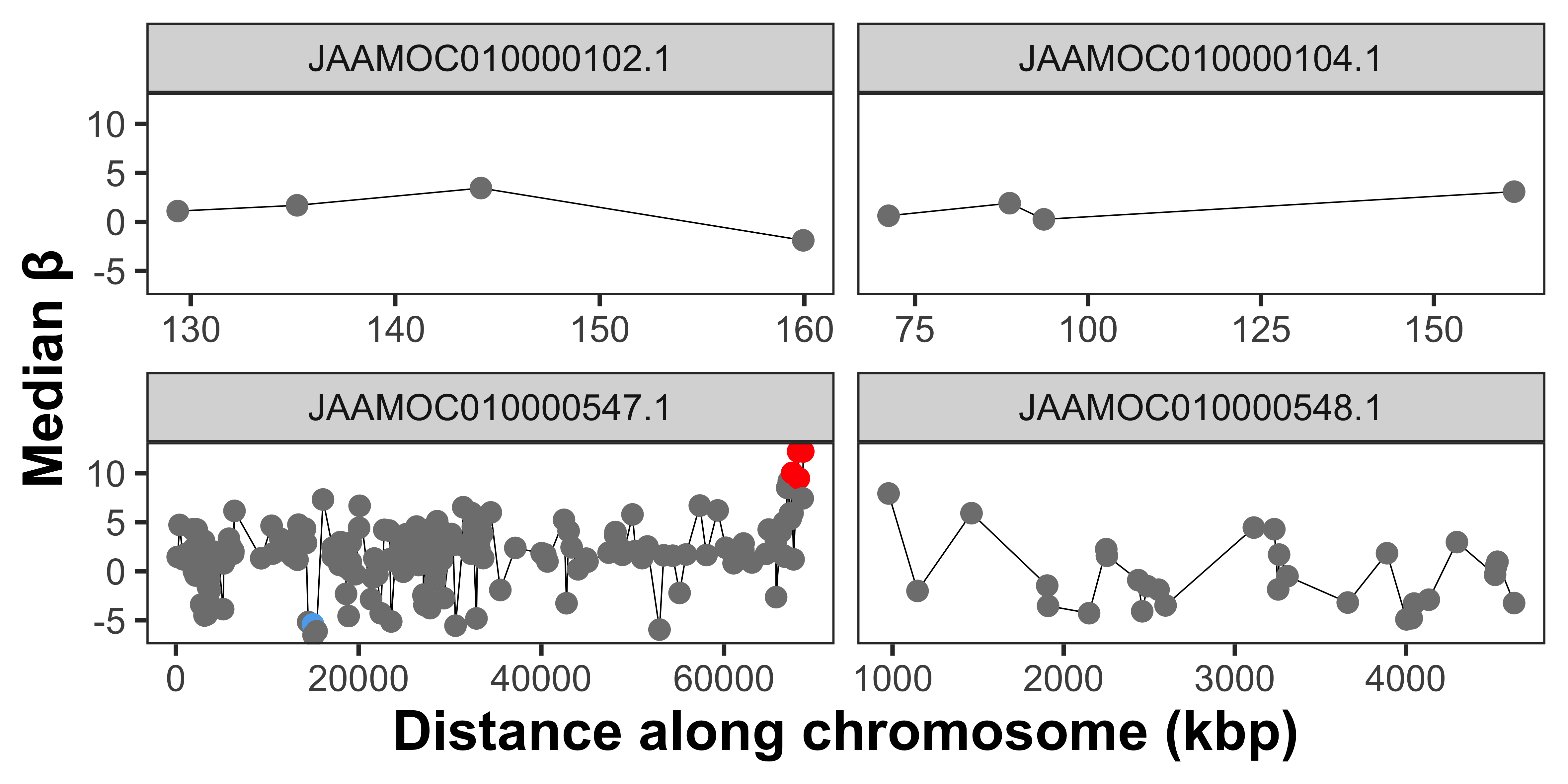
**

**Chromosome 1B**

**
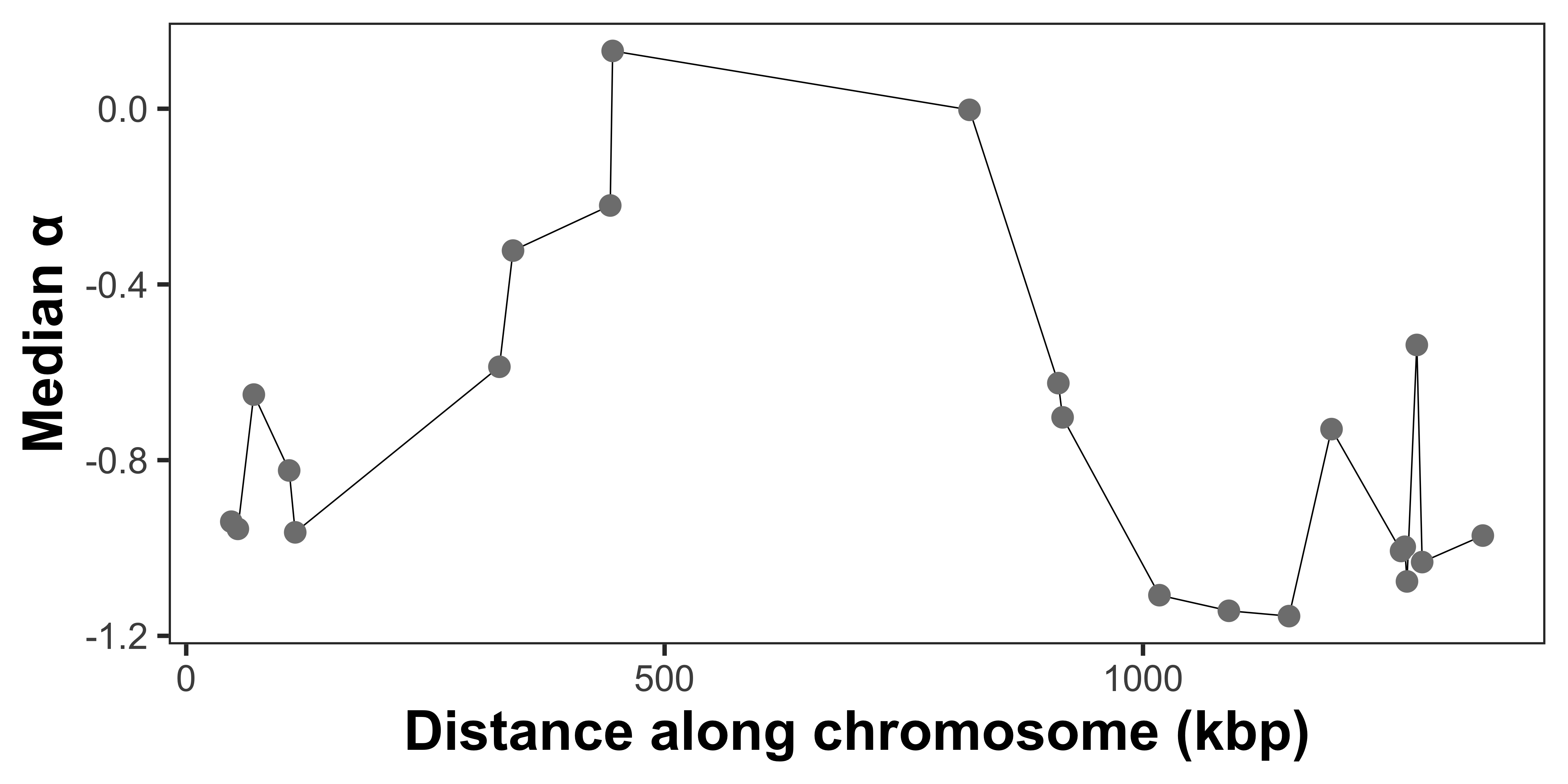

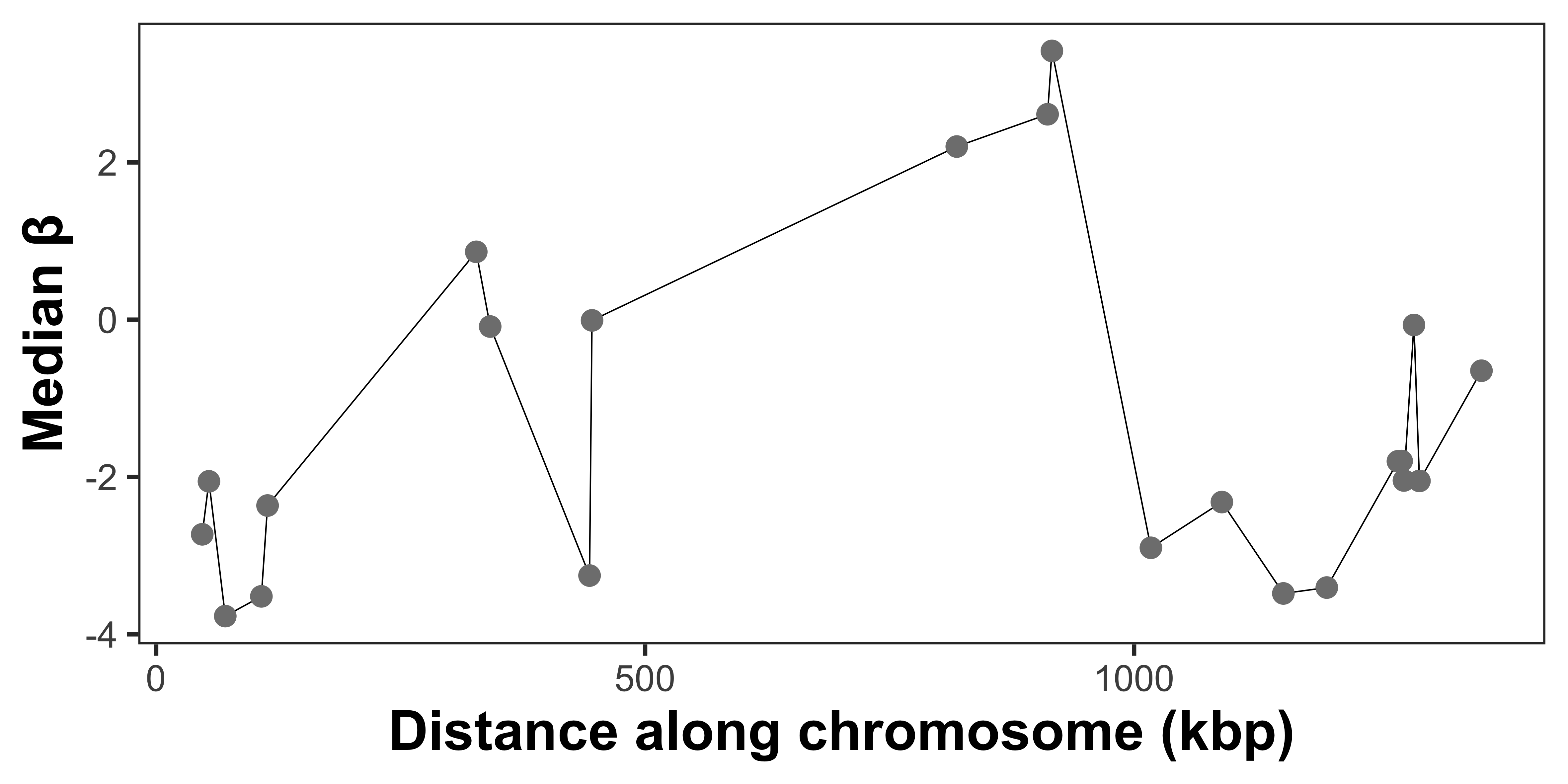
**

**Chromosome 2:** JAAMOC010000004.1 not displayed as only one RAD marker present

**
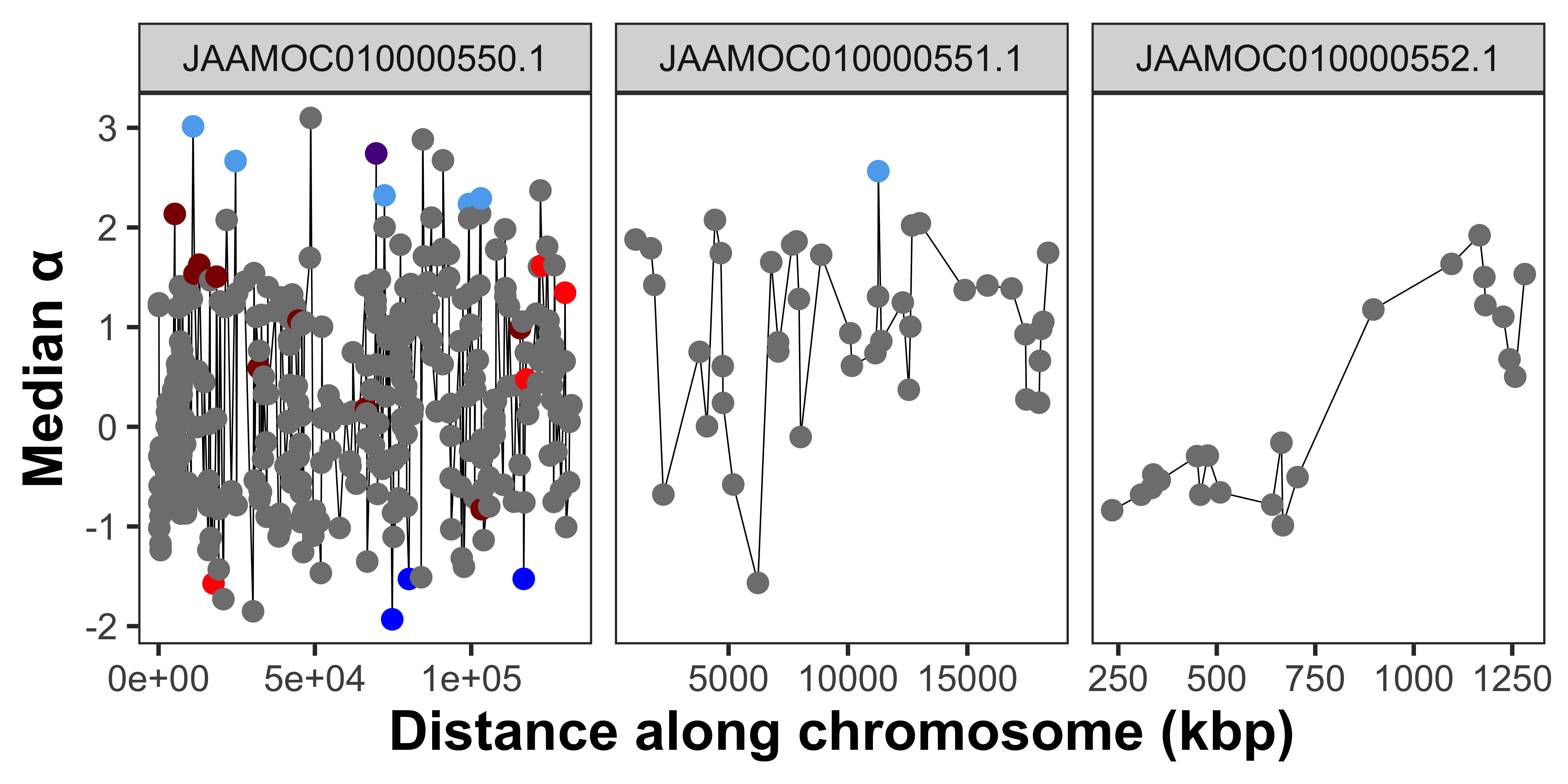

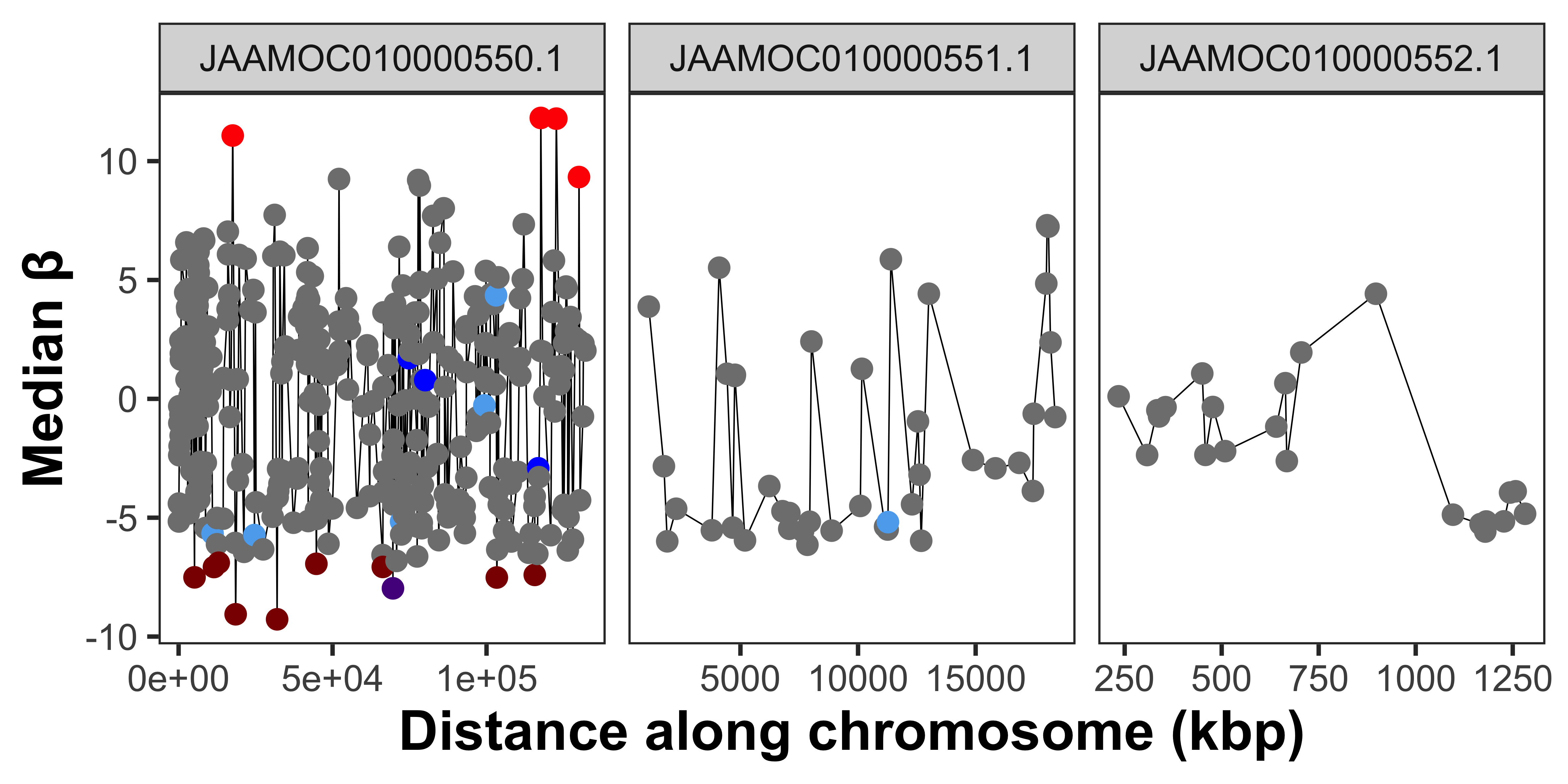
**


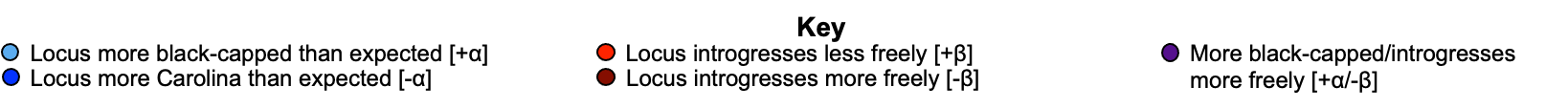
**Chromosome 3:** JAAMOC010000016.1 not displayed as only one RAD marker present

**
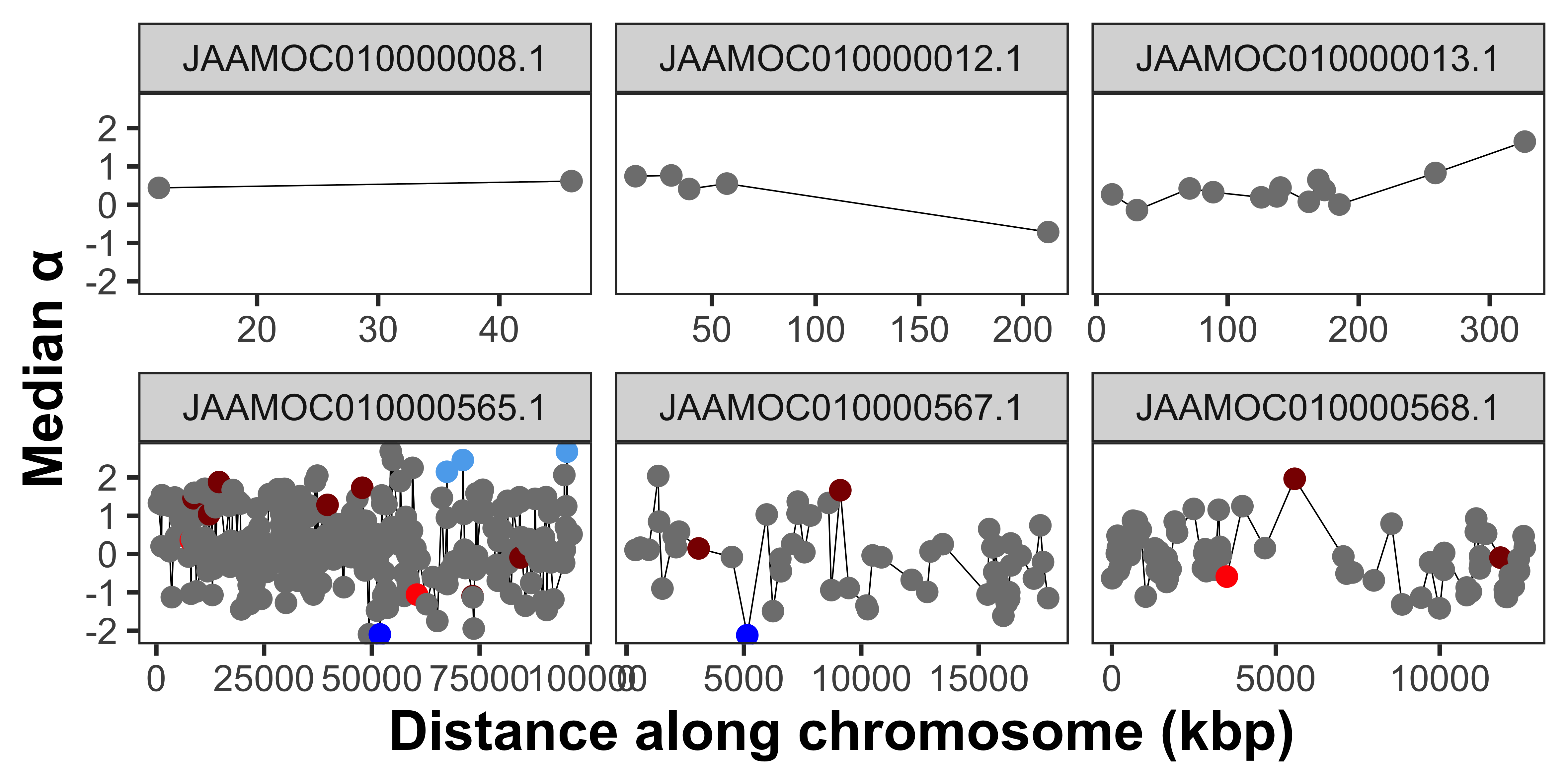

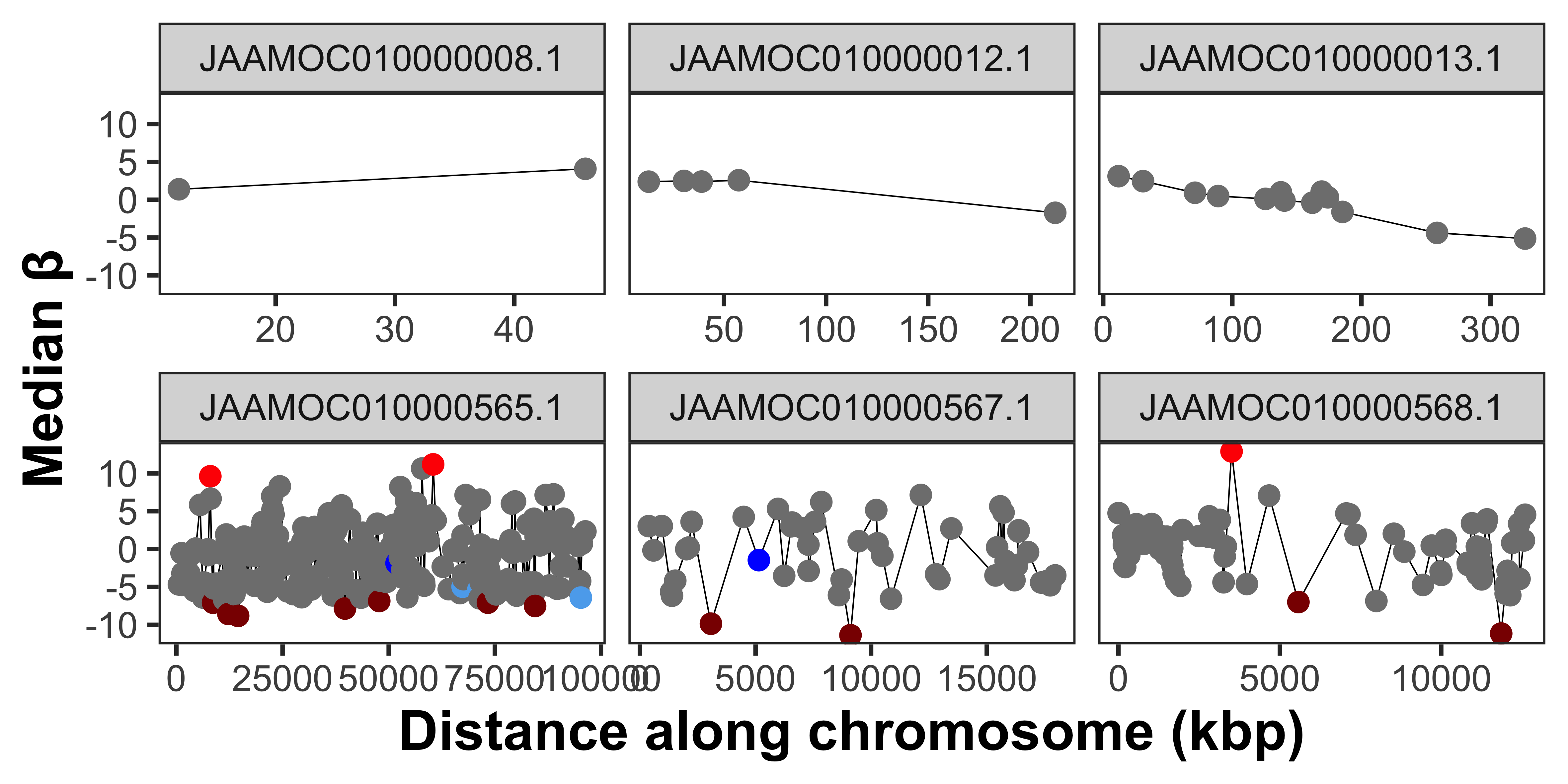
**

**Chromosome 4**

**
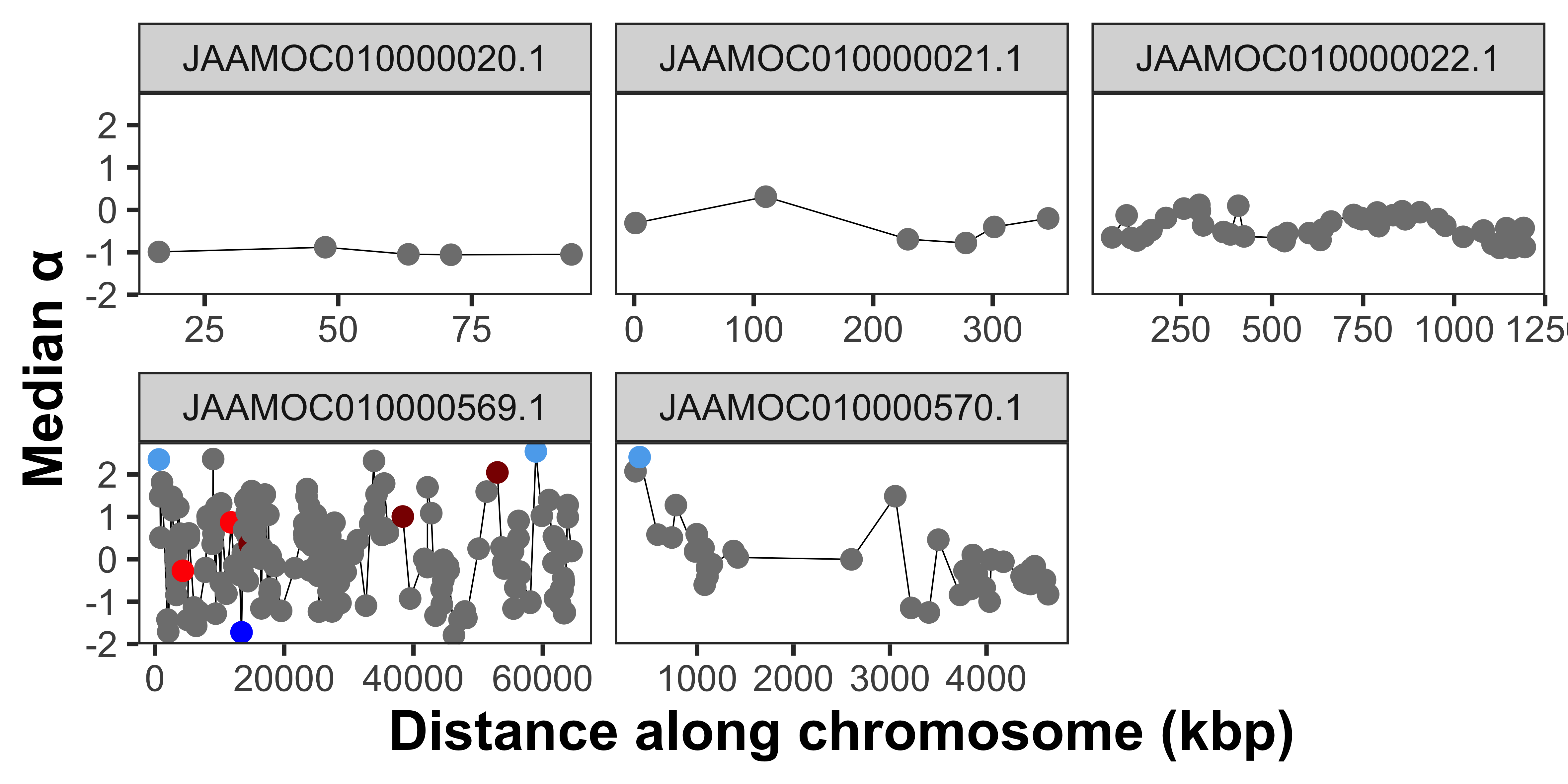

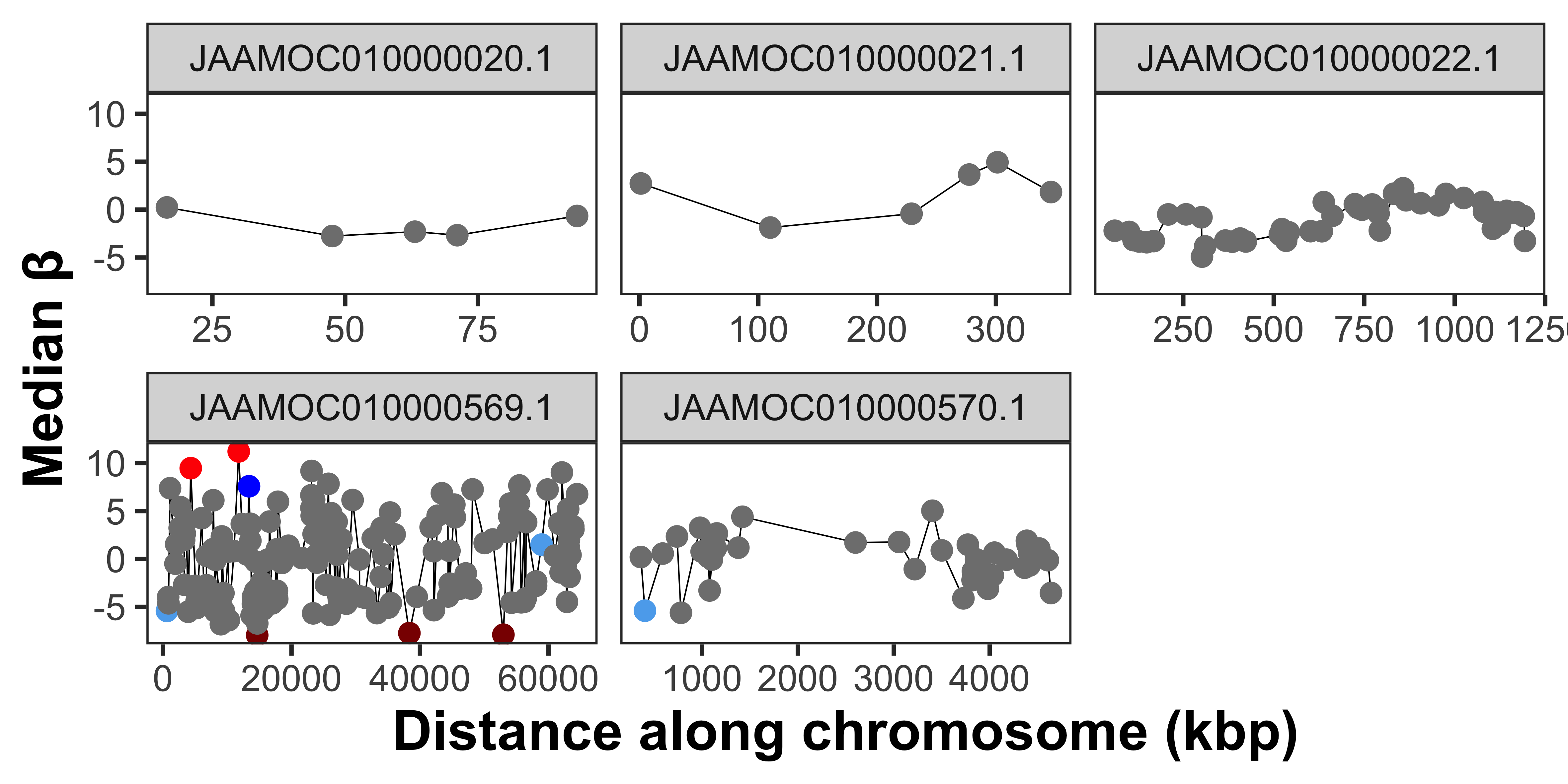
**

**Chromosome 4A**

**
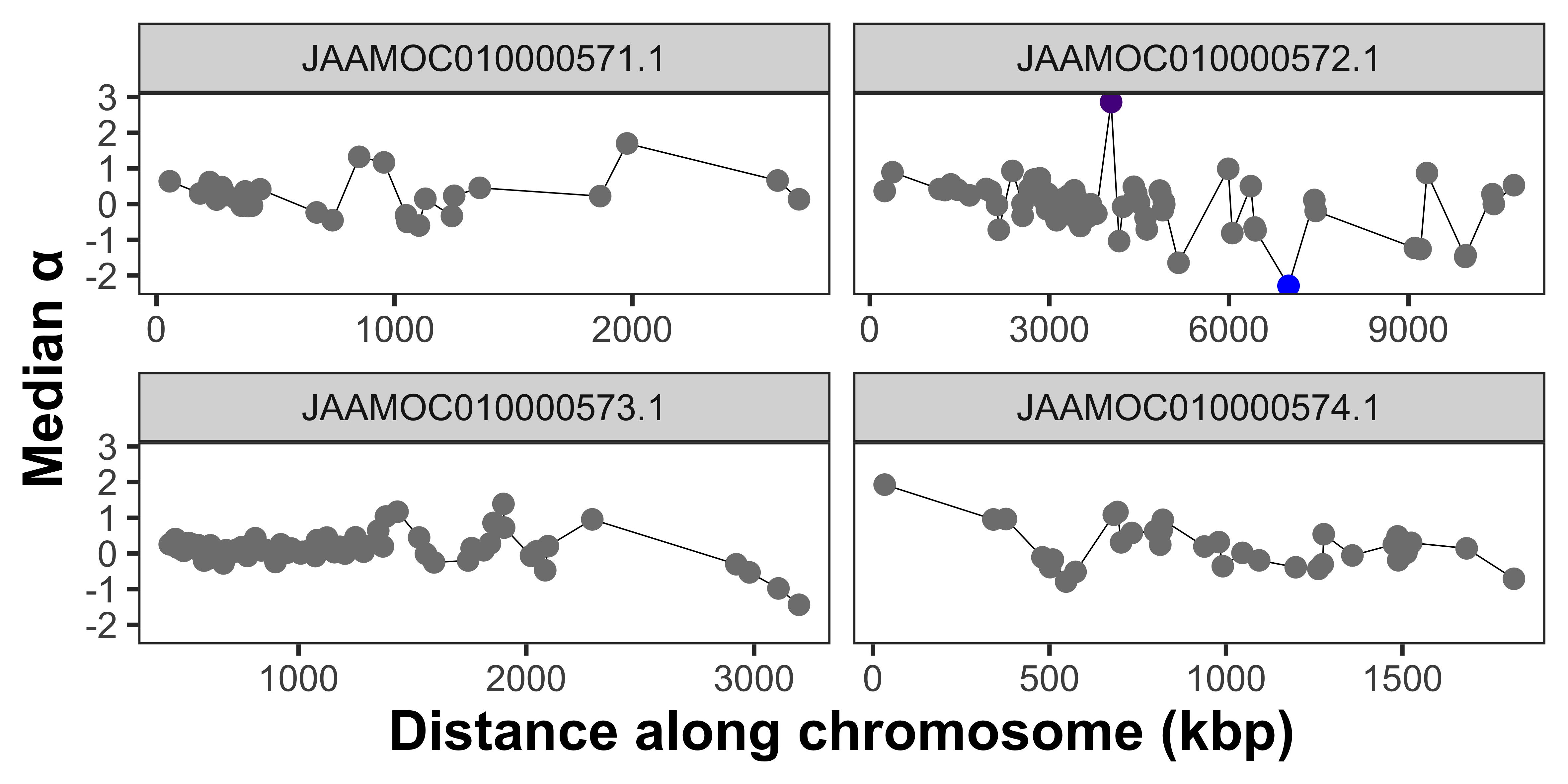

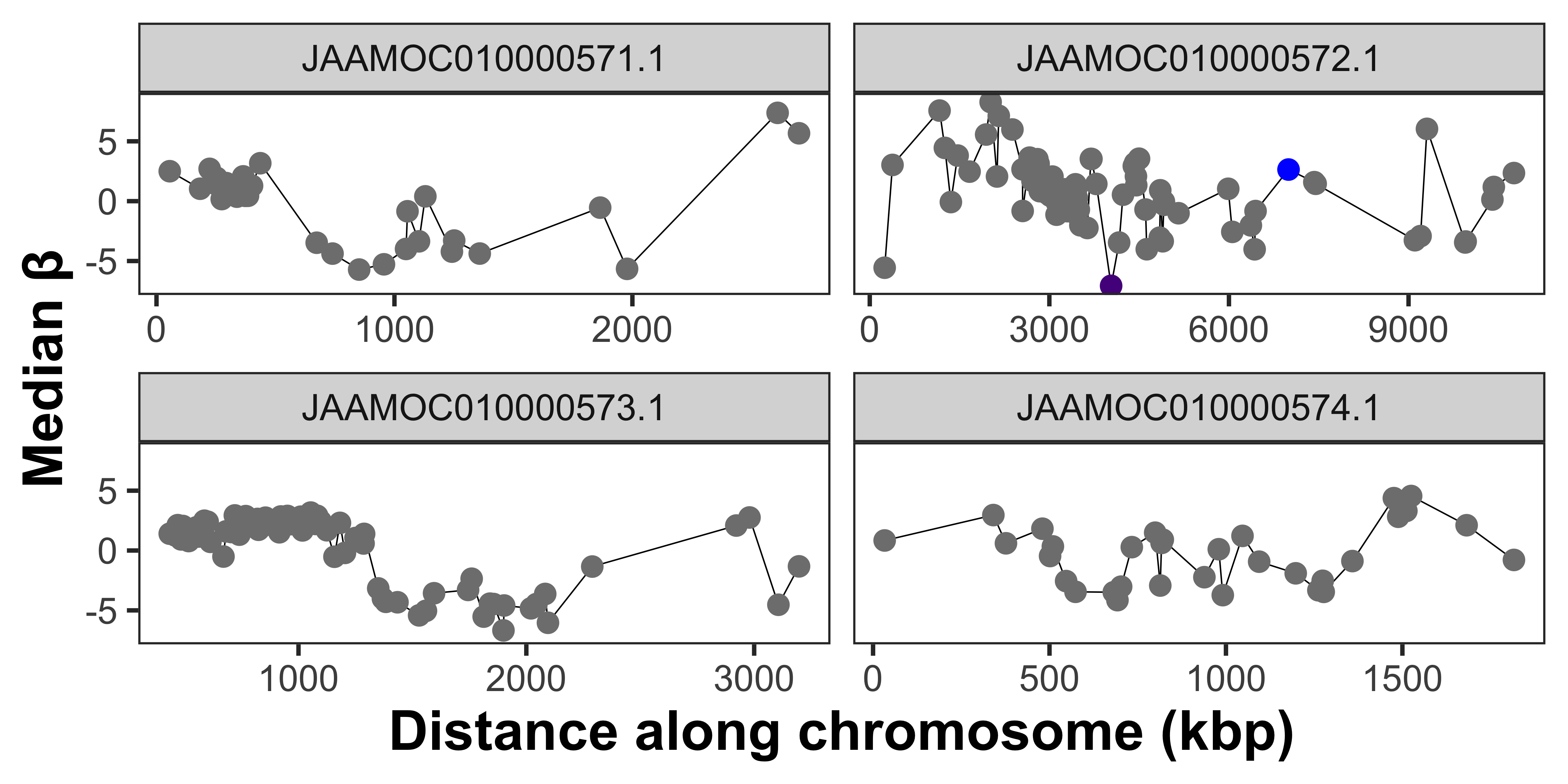
**

**Chromosome 5**

**
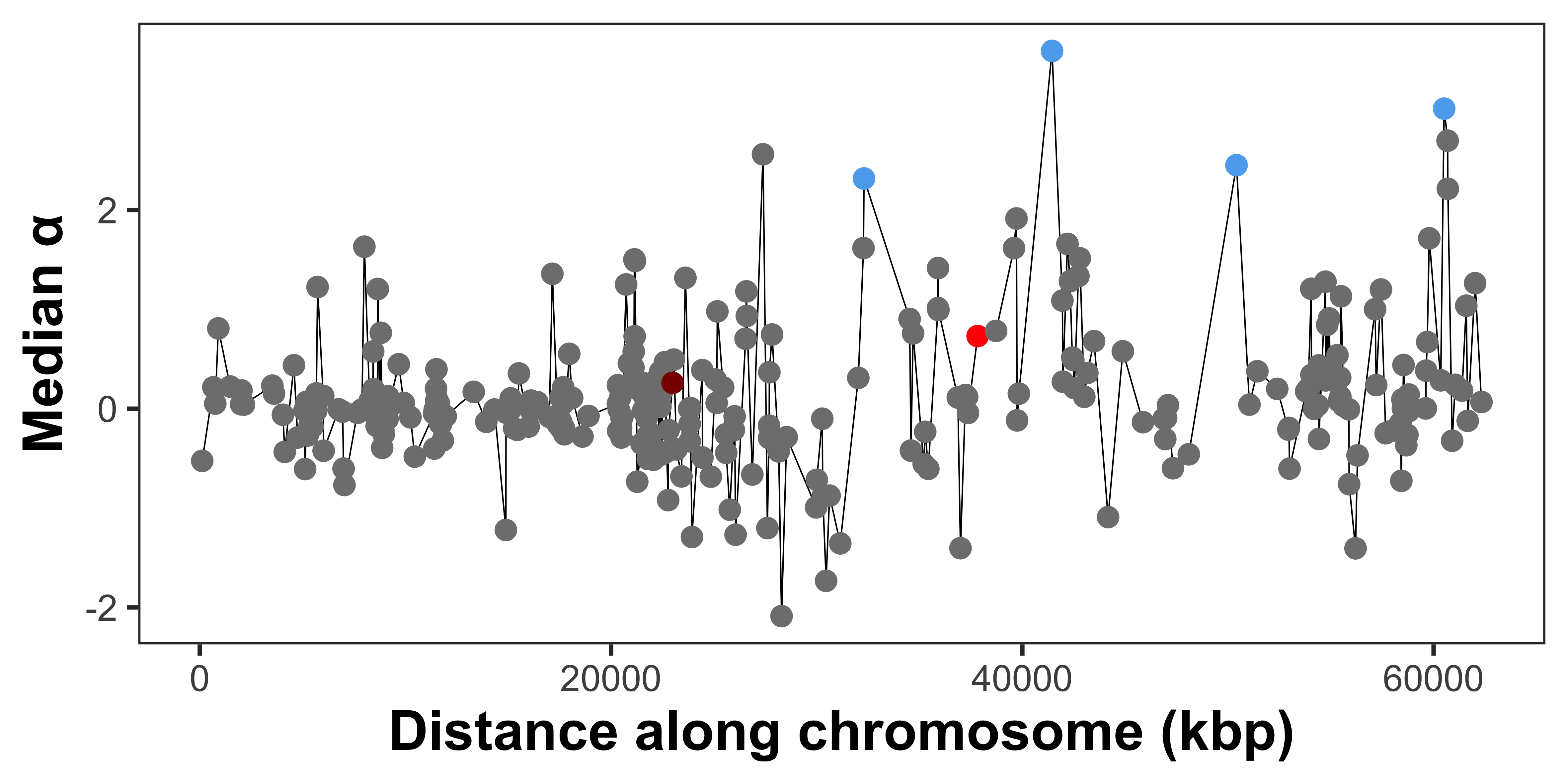

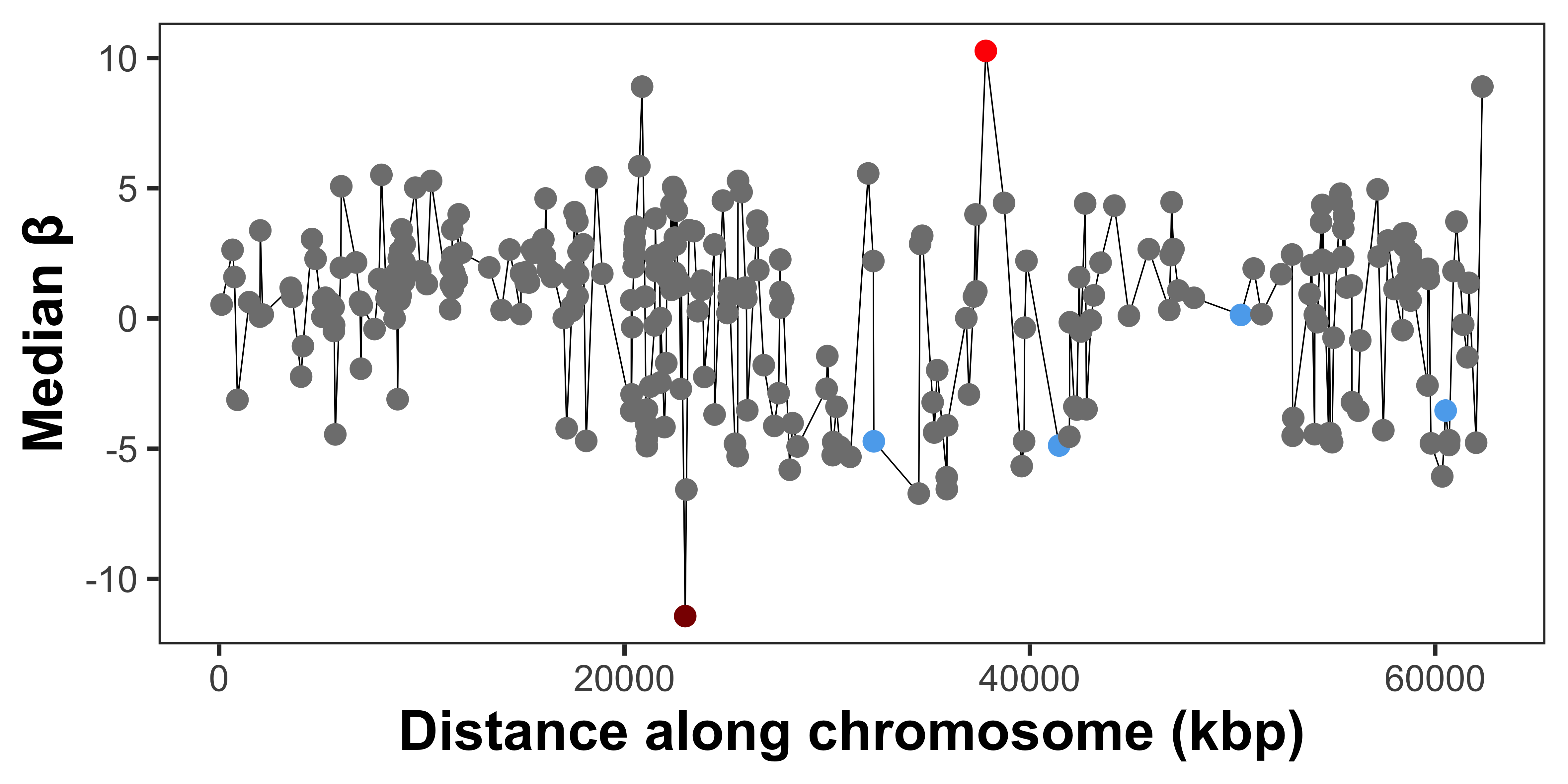
**


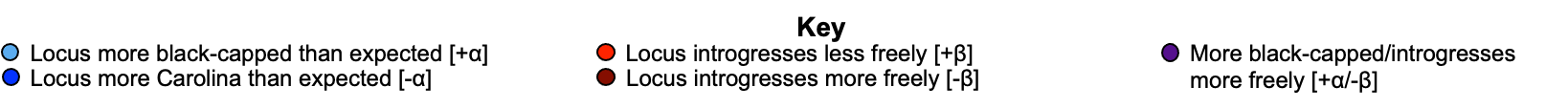
**Chromosome 6:** JAAMOC010000029.1 not displayed as only one RAD marker present

**
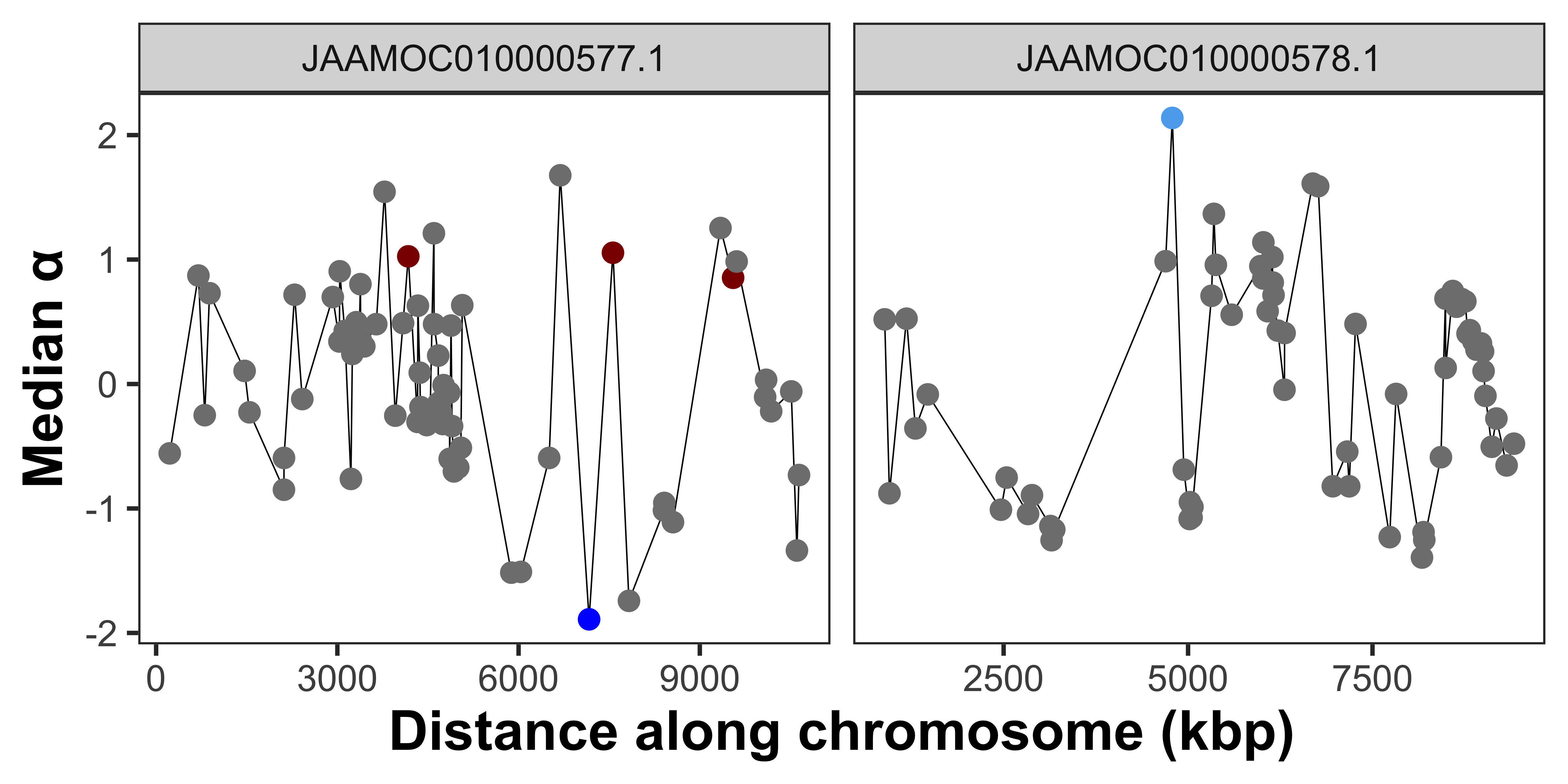

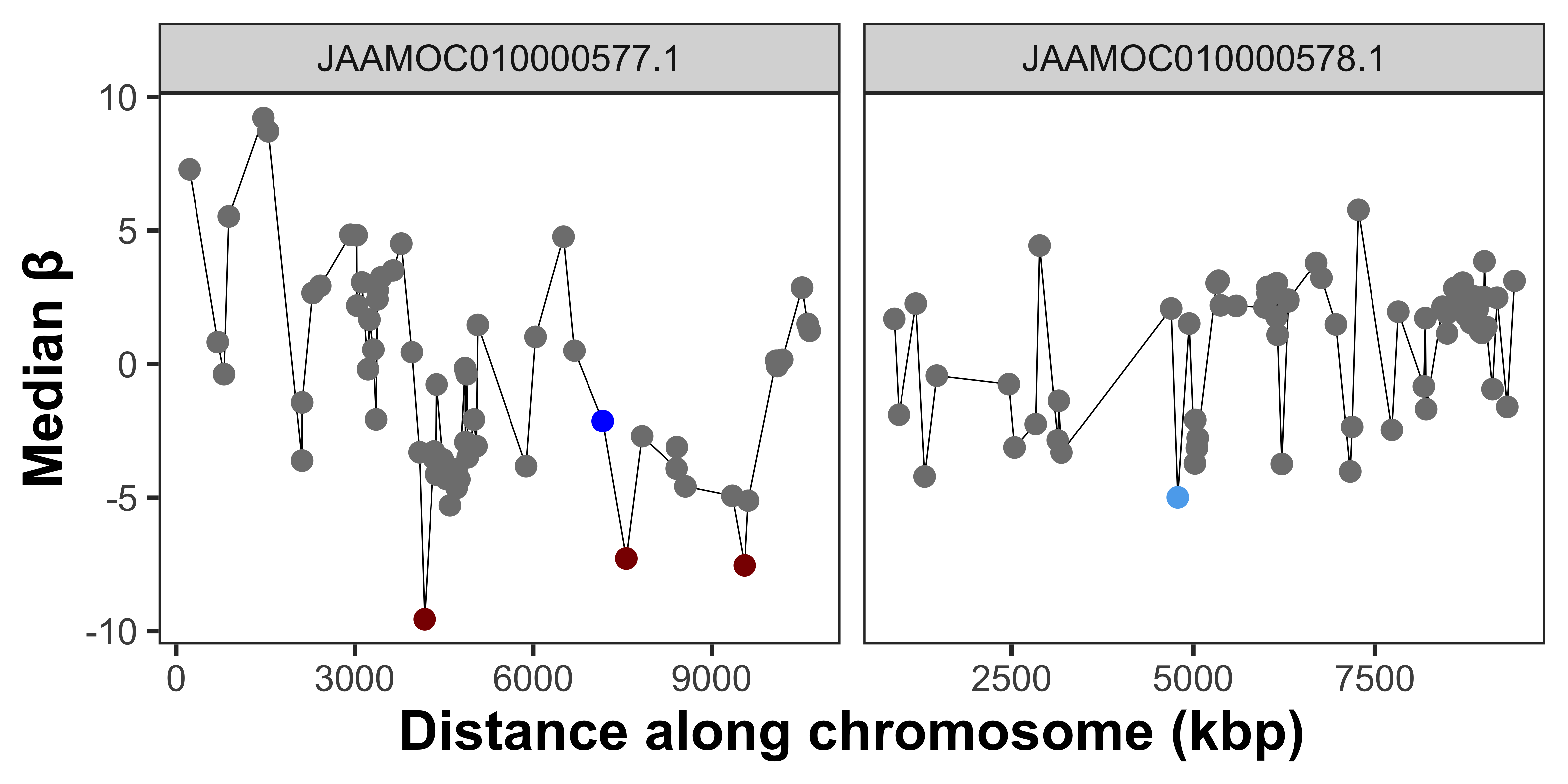
**

**Chromosome 7**

**
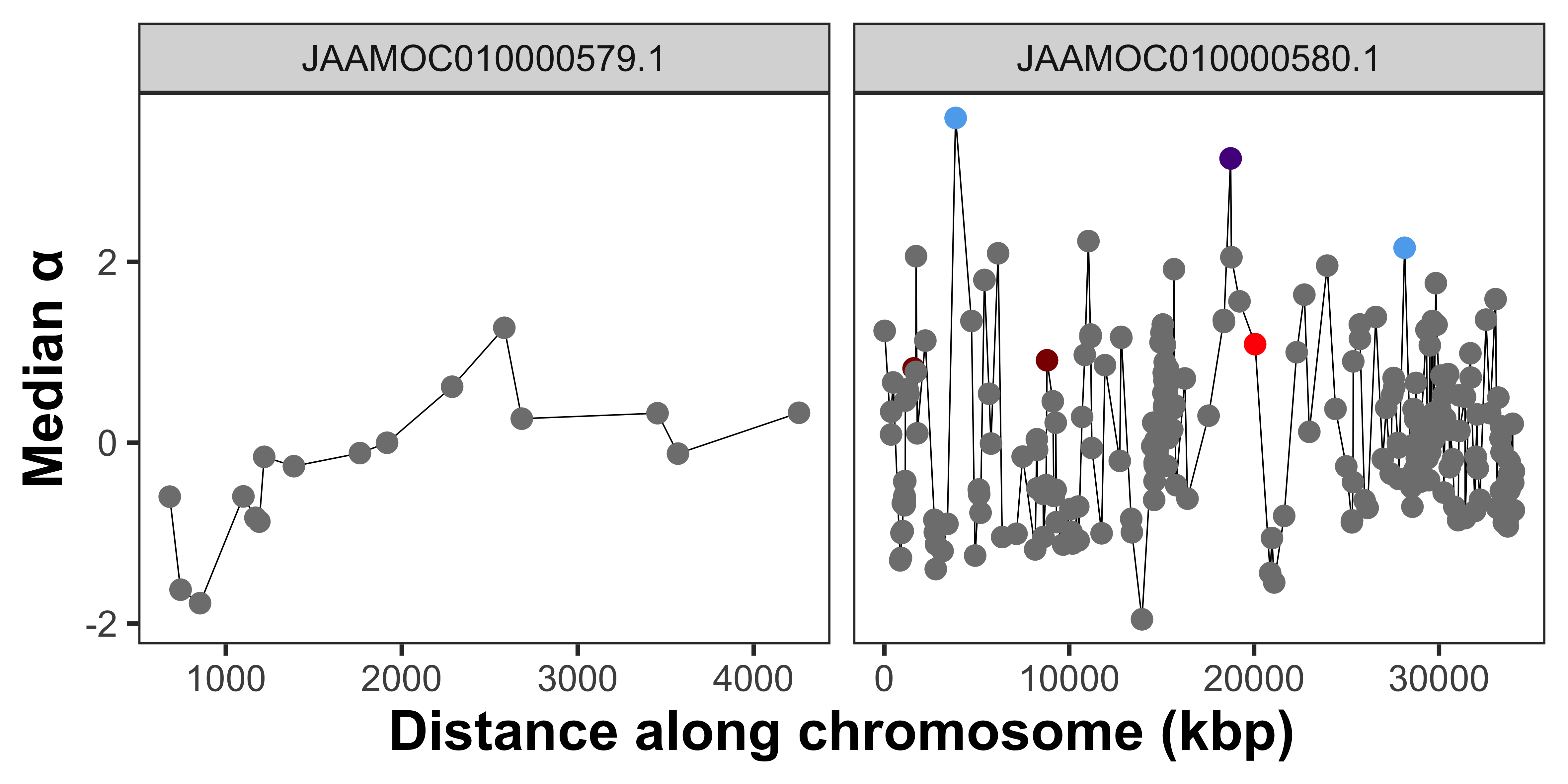

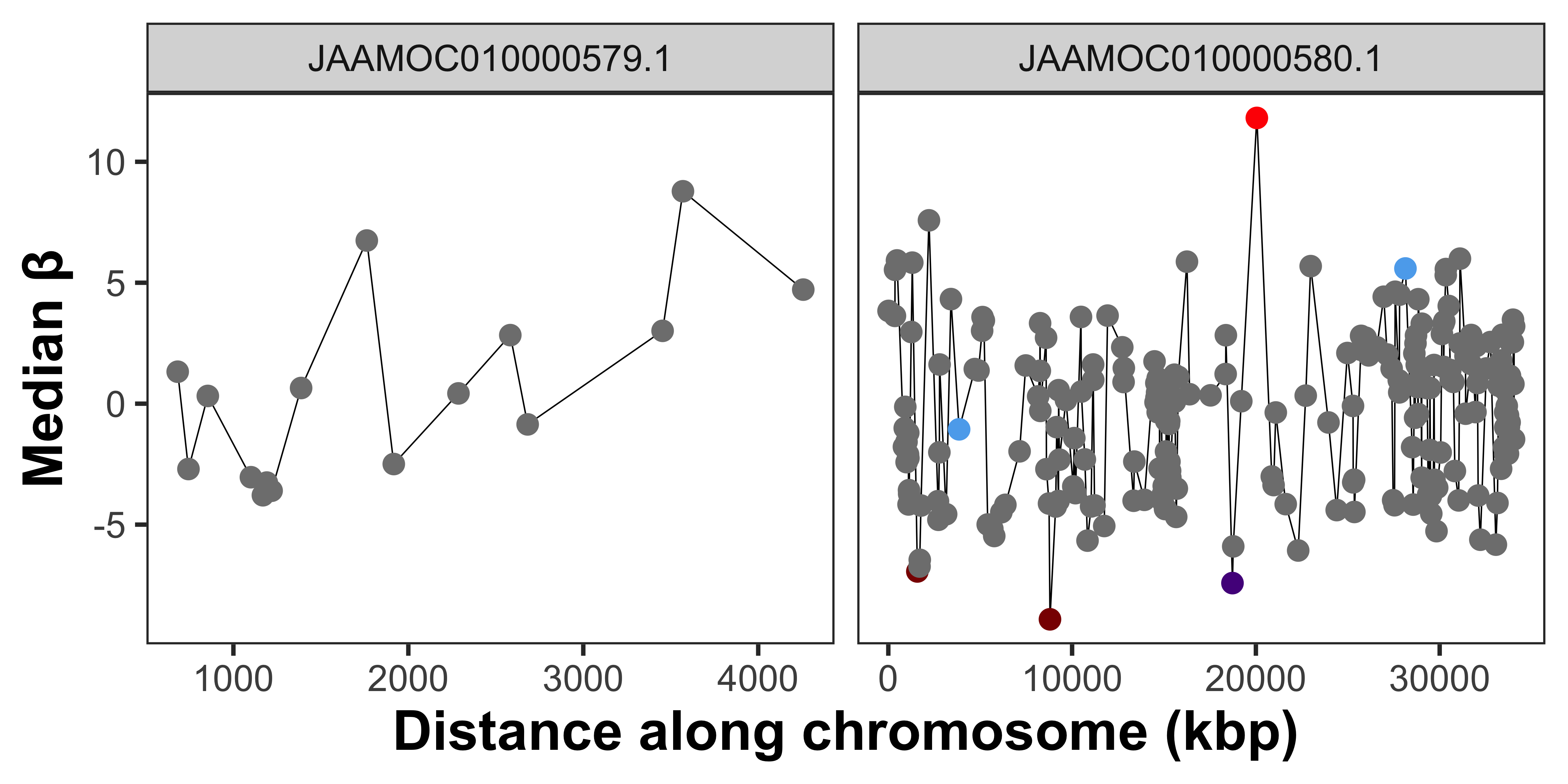
**

**Chromosome 8**

**
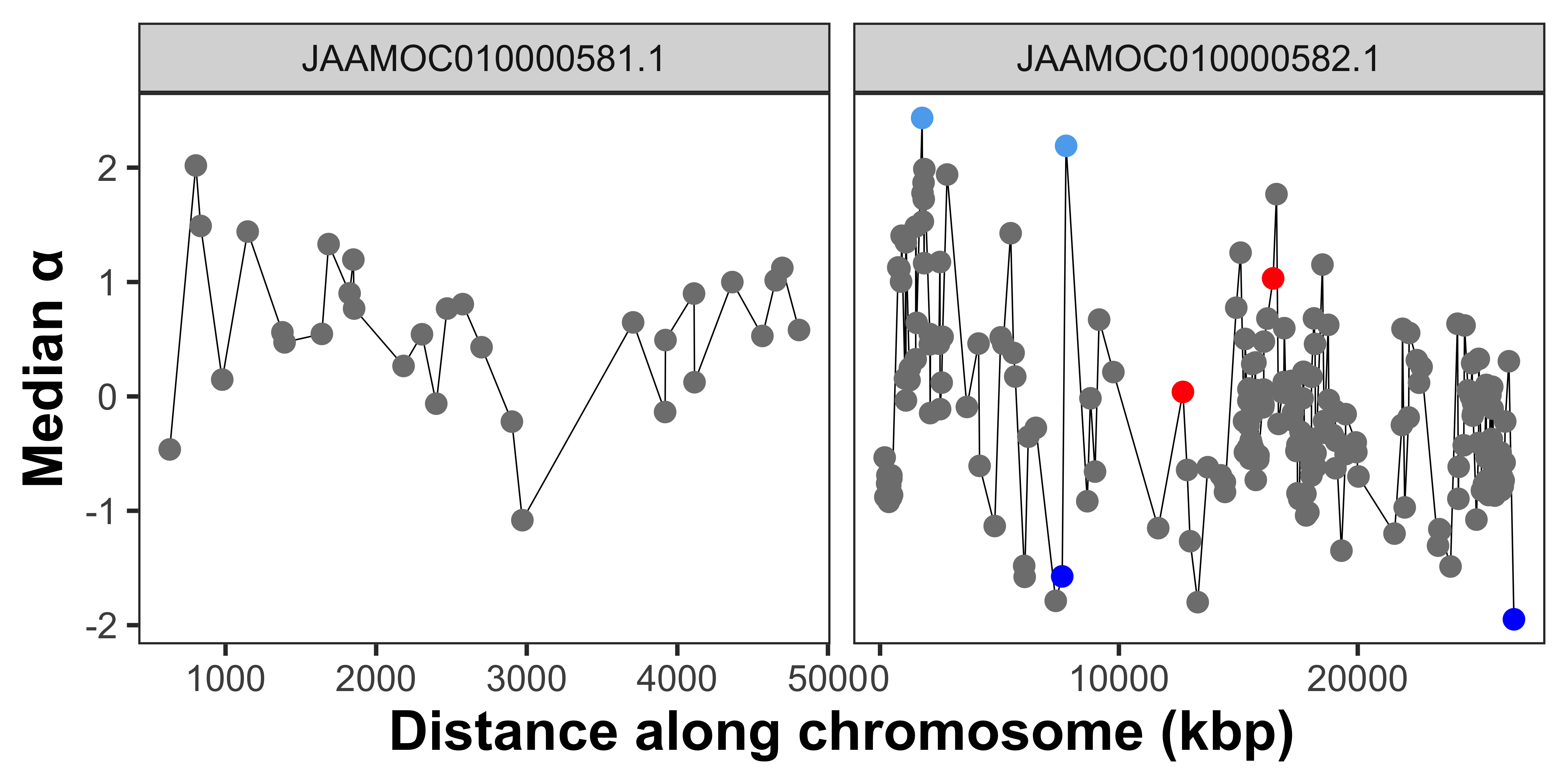

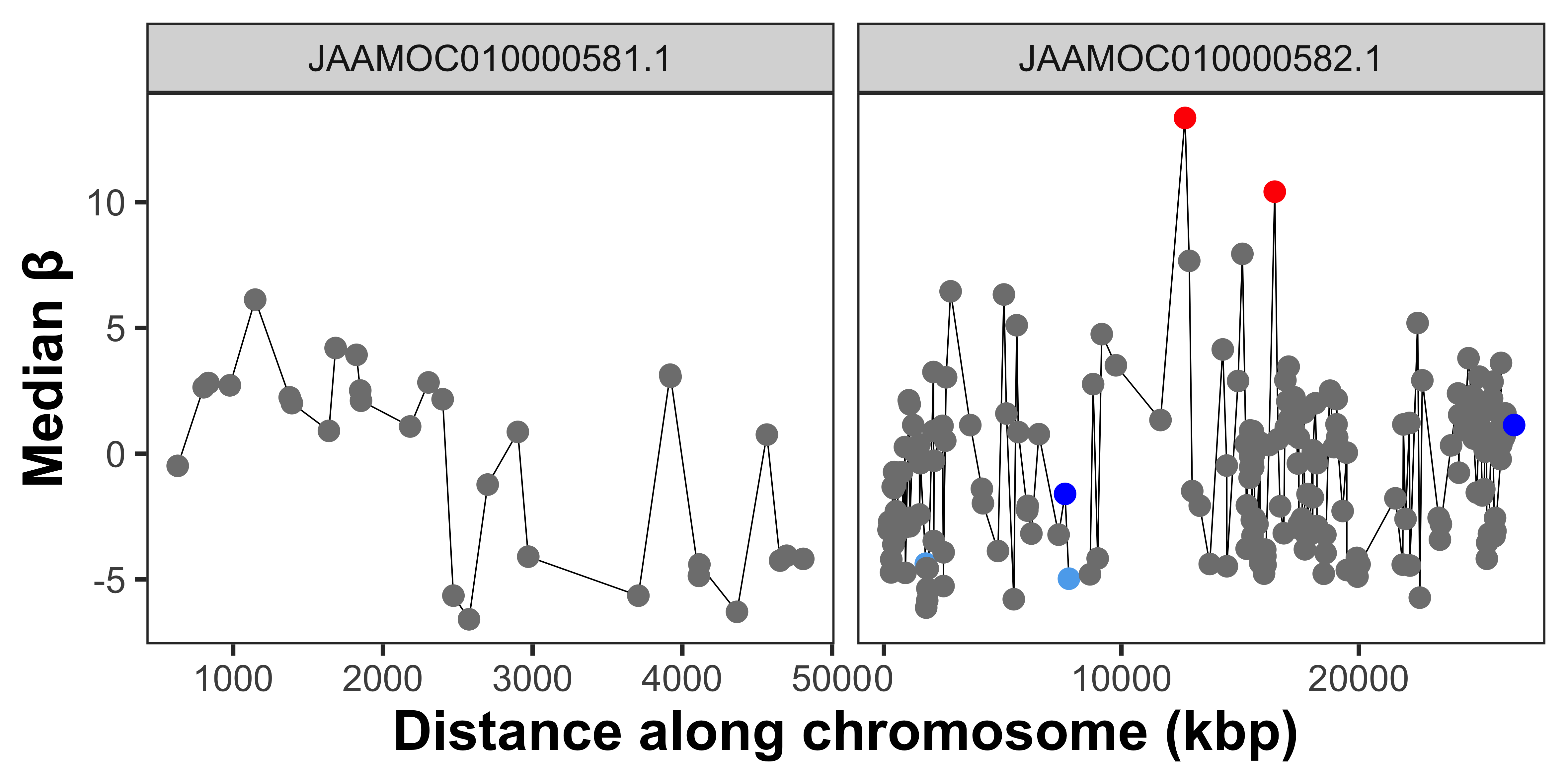
**

**Chromosome 9**

**
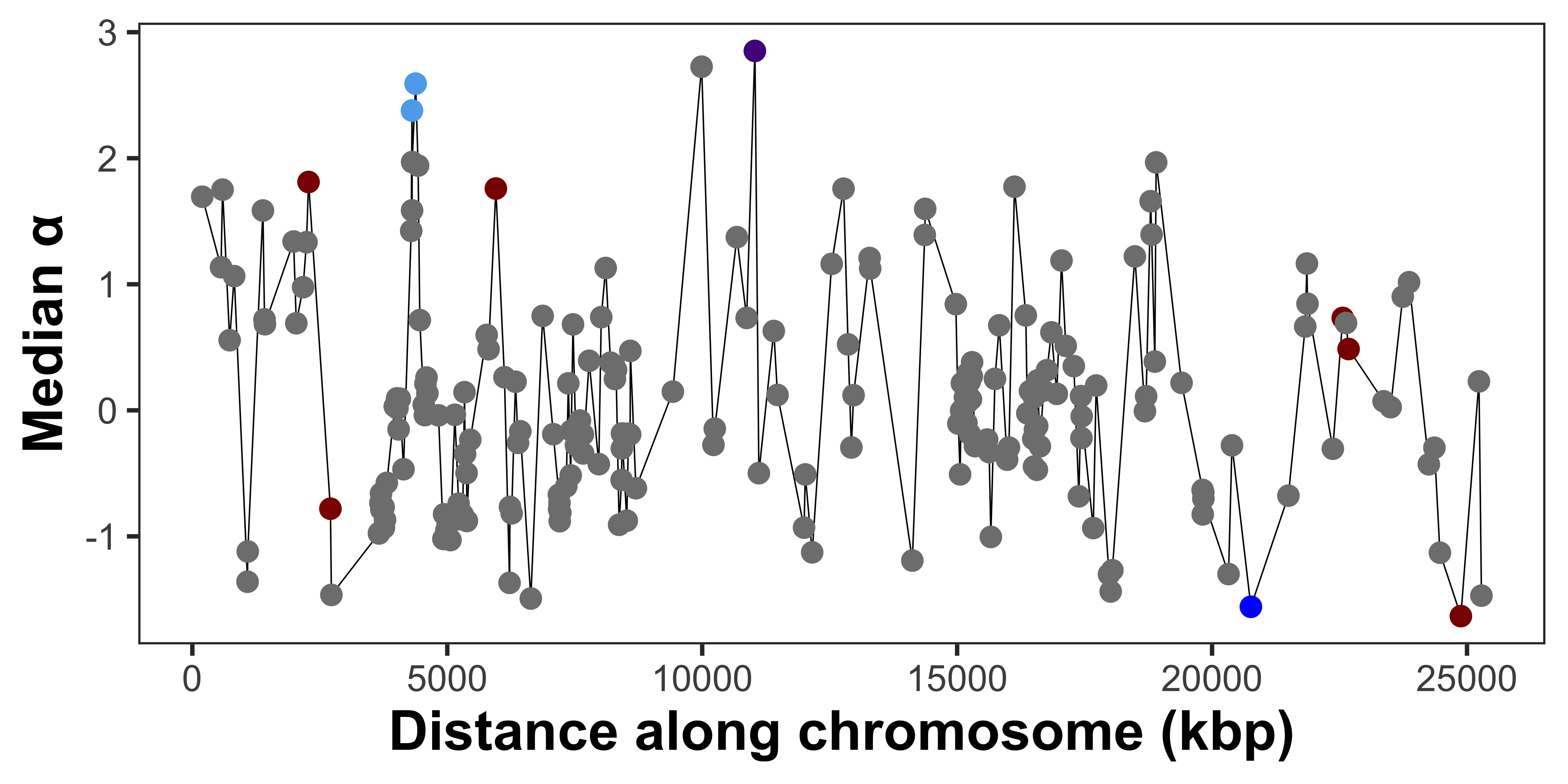

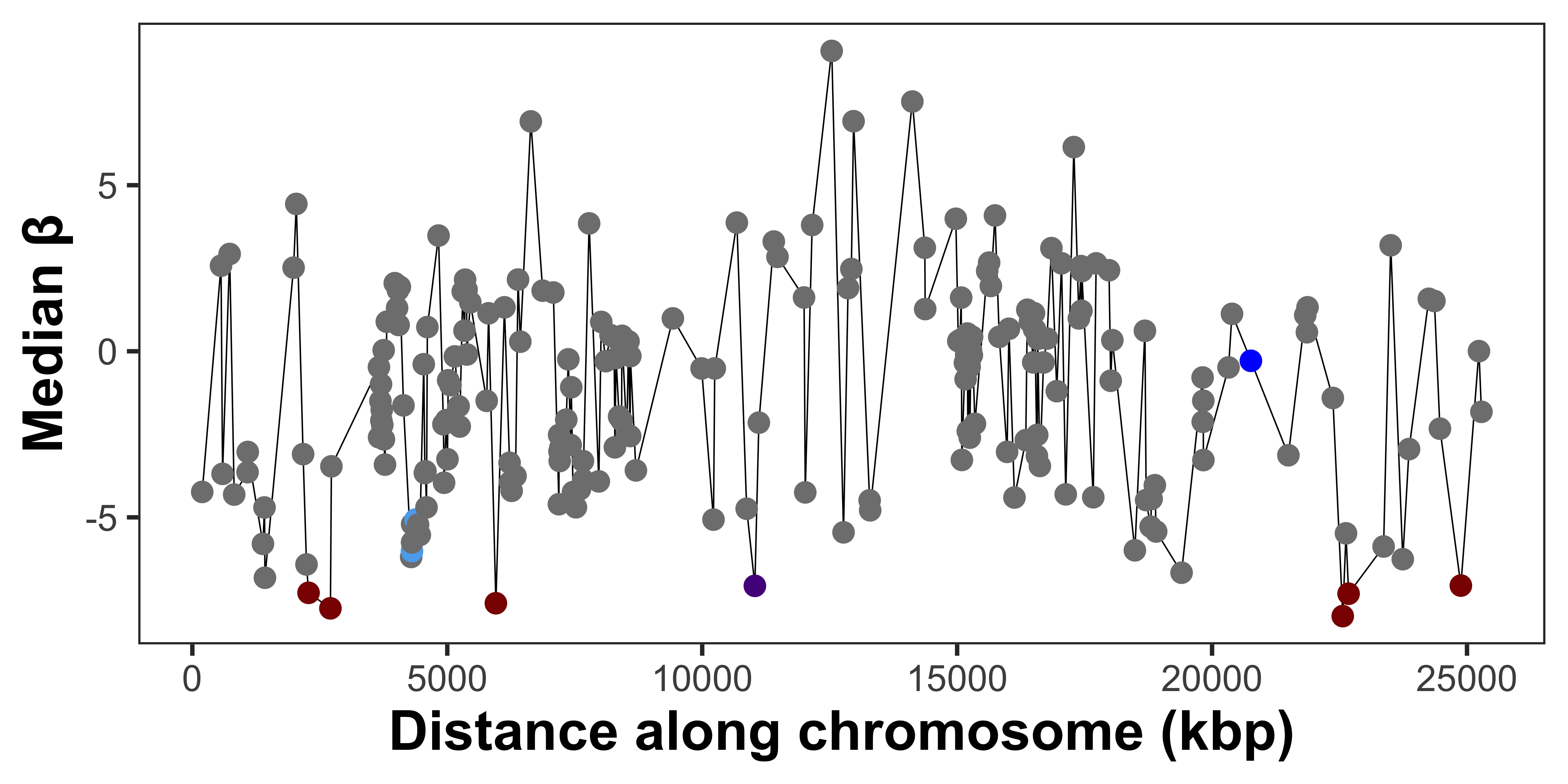
**


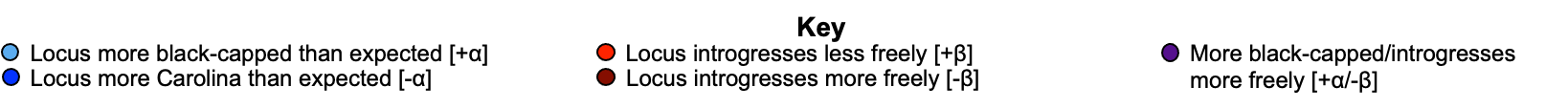
**Chromosome 10**

**
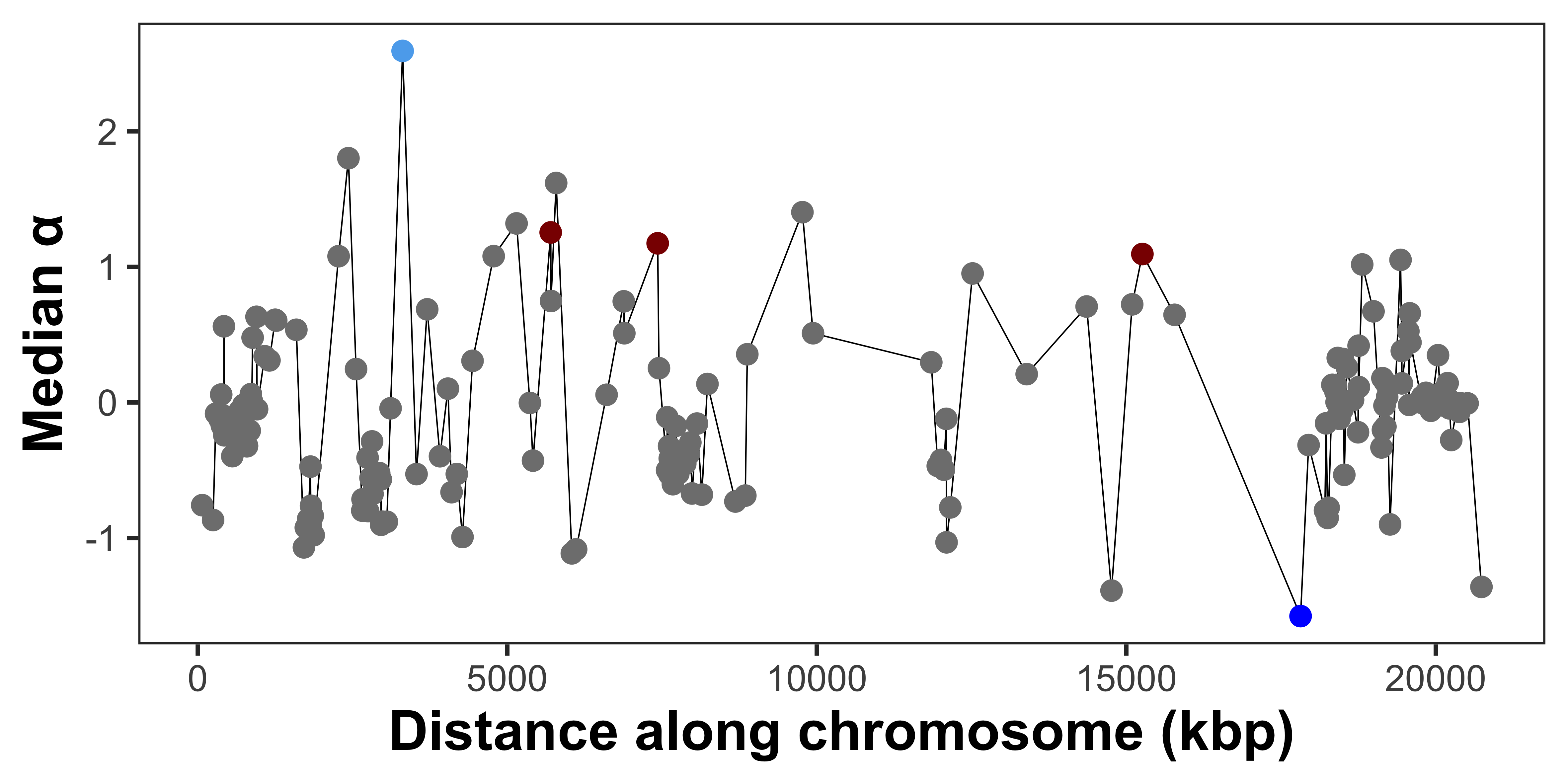

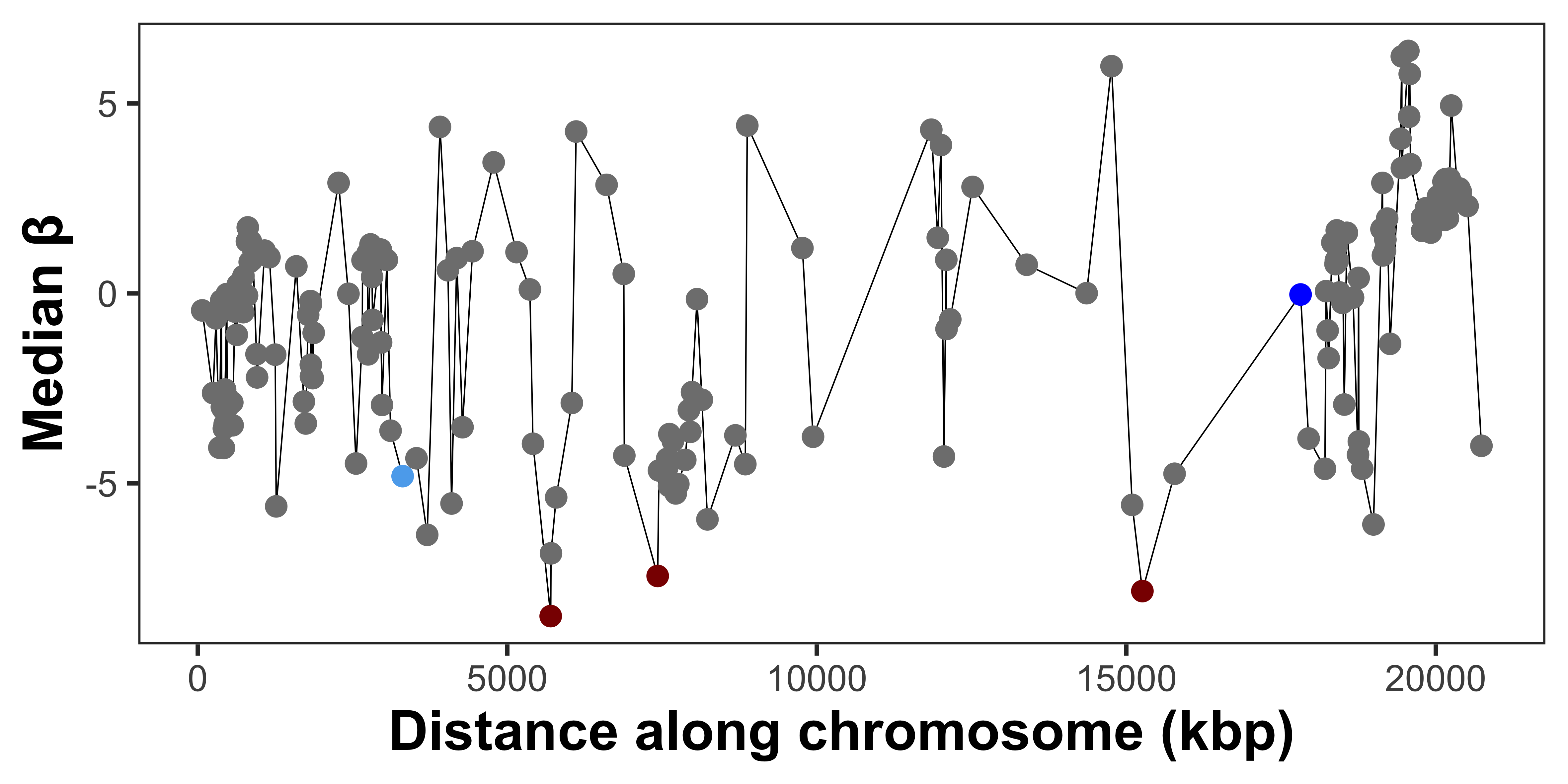
**

**Chromosome 11**

**
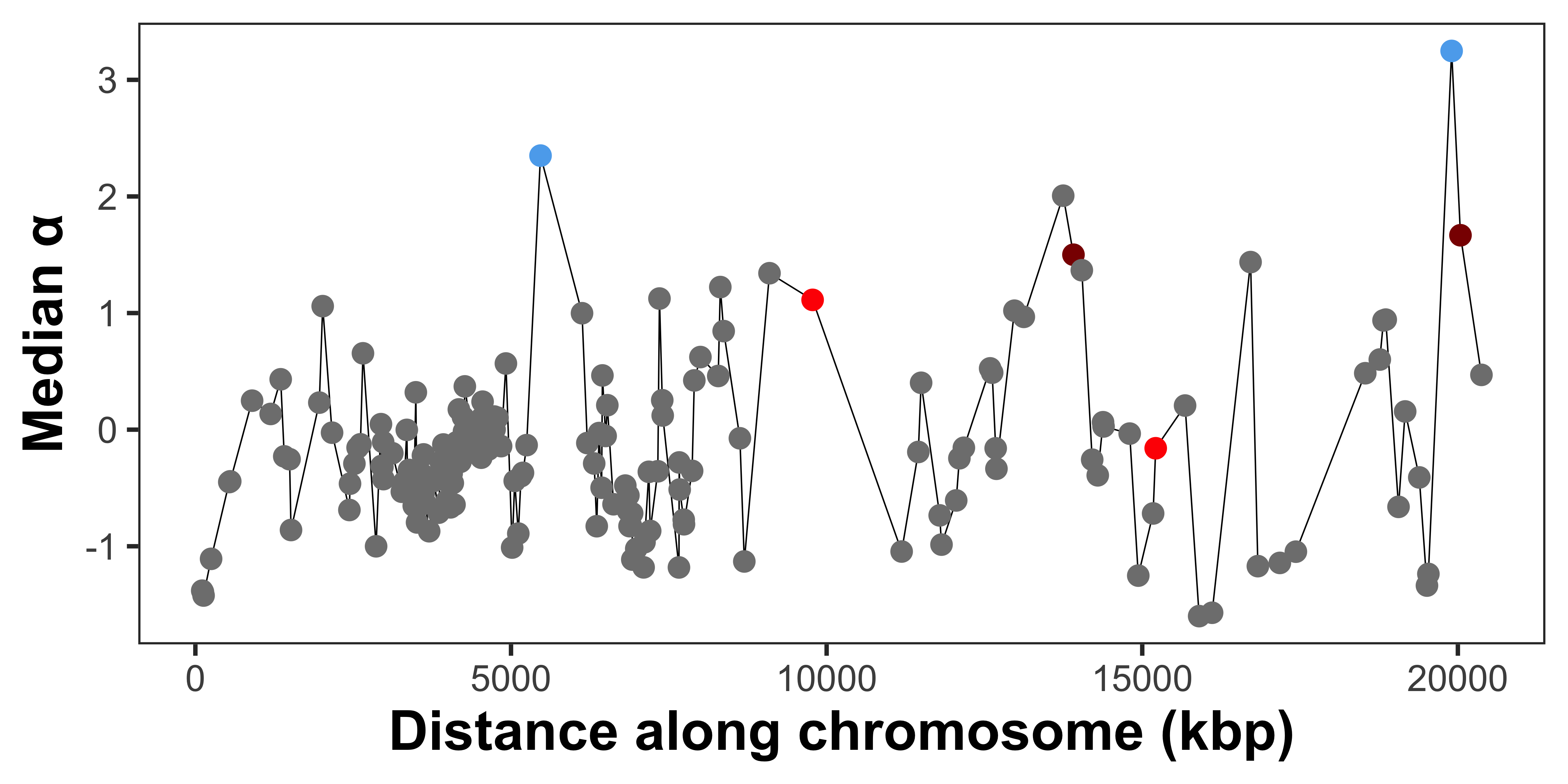

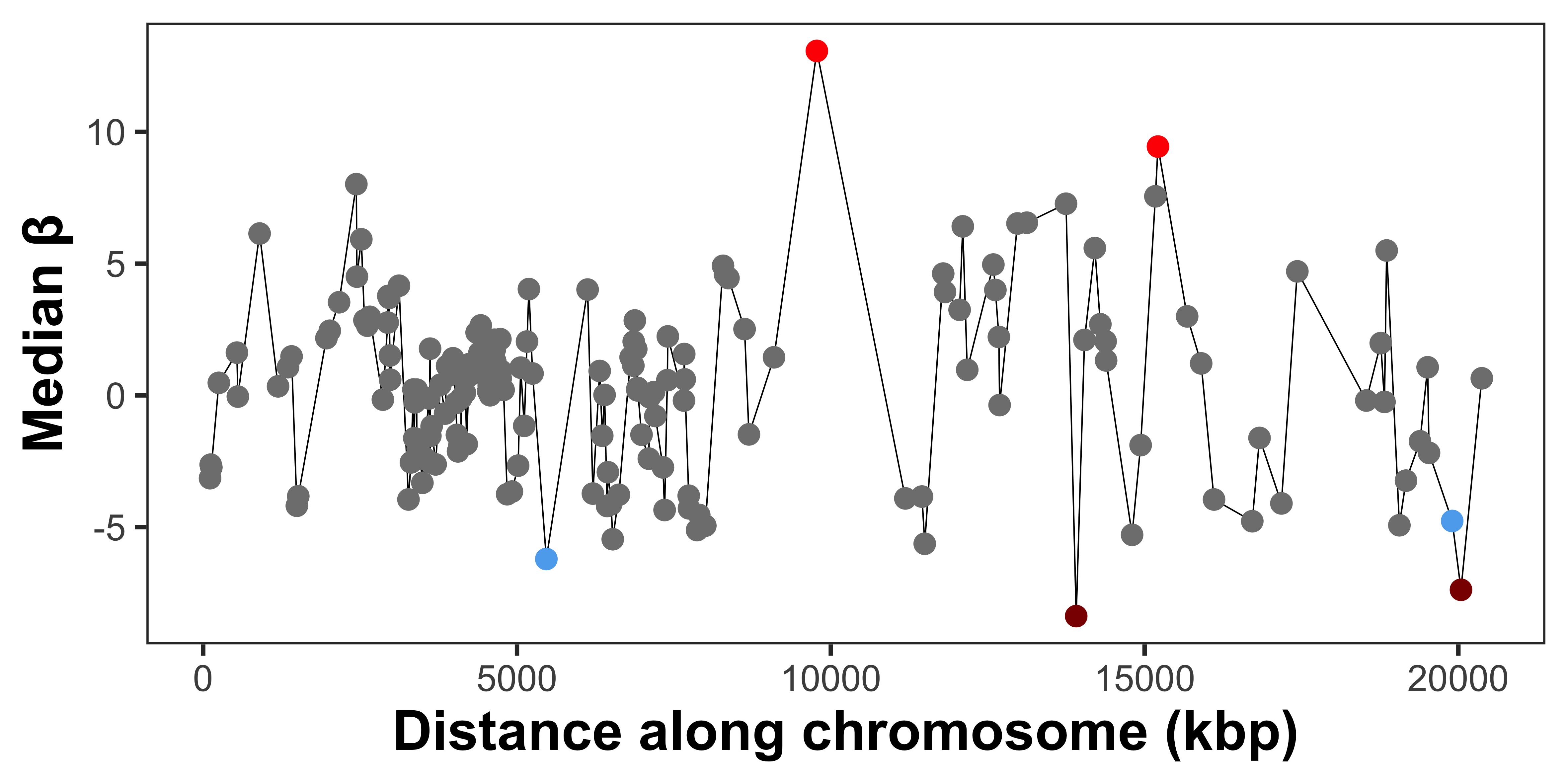
**

**Chromosome 12**

**
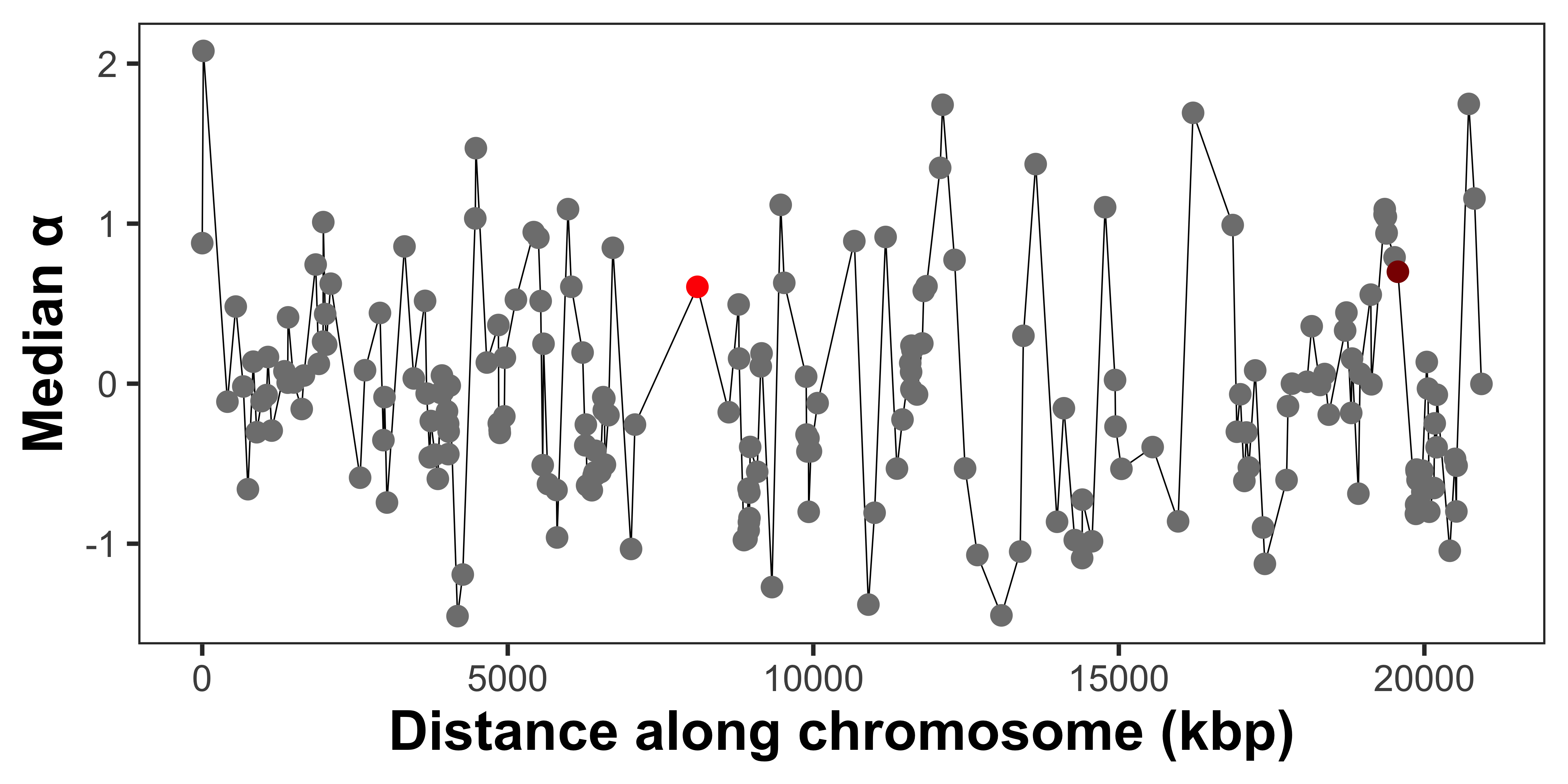

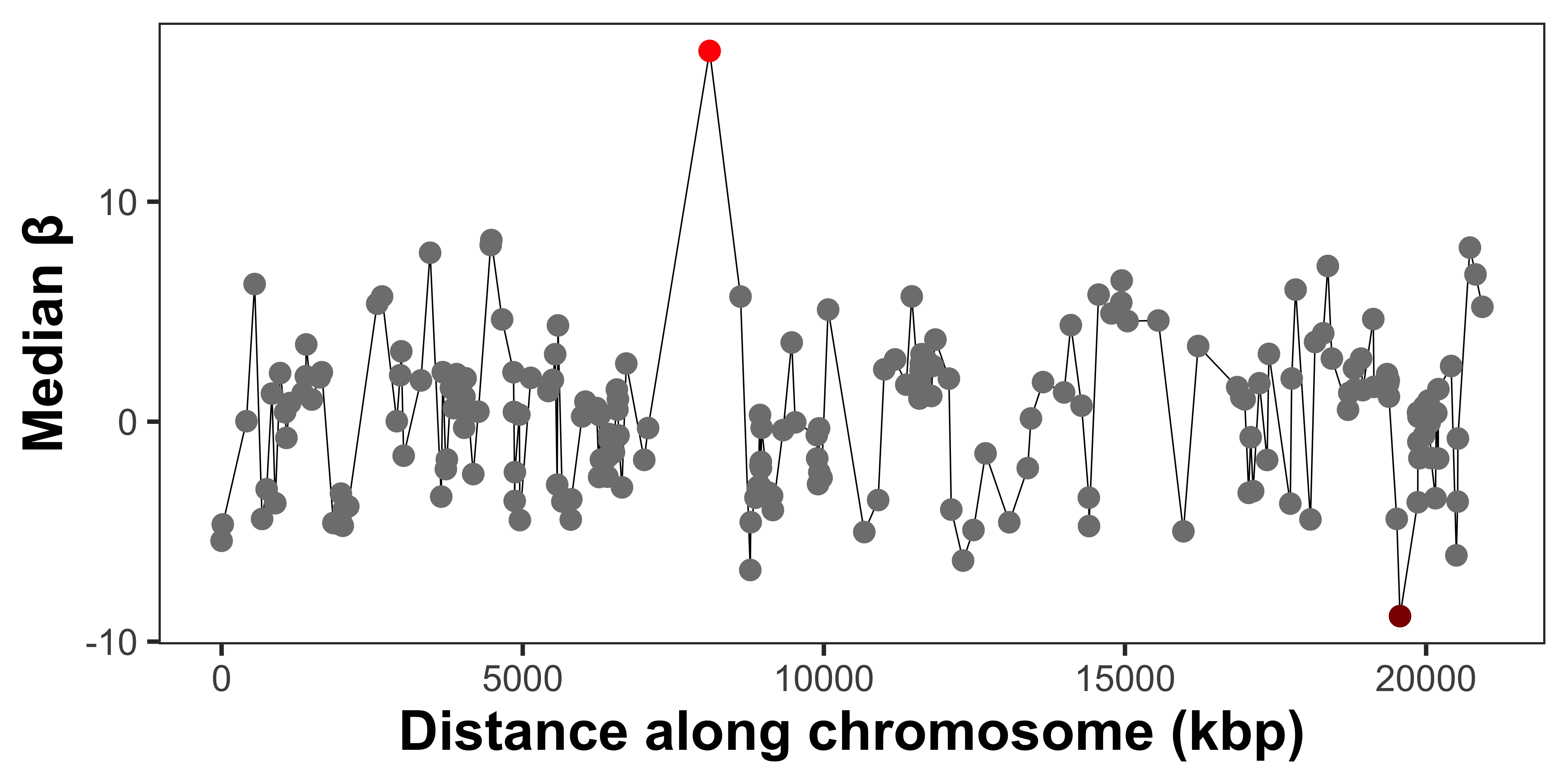
**

**Chromosome 13**

**
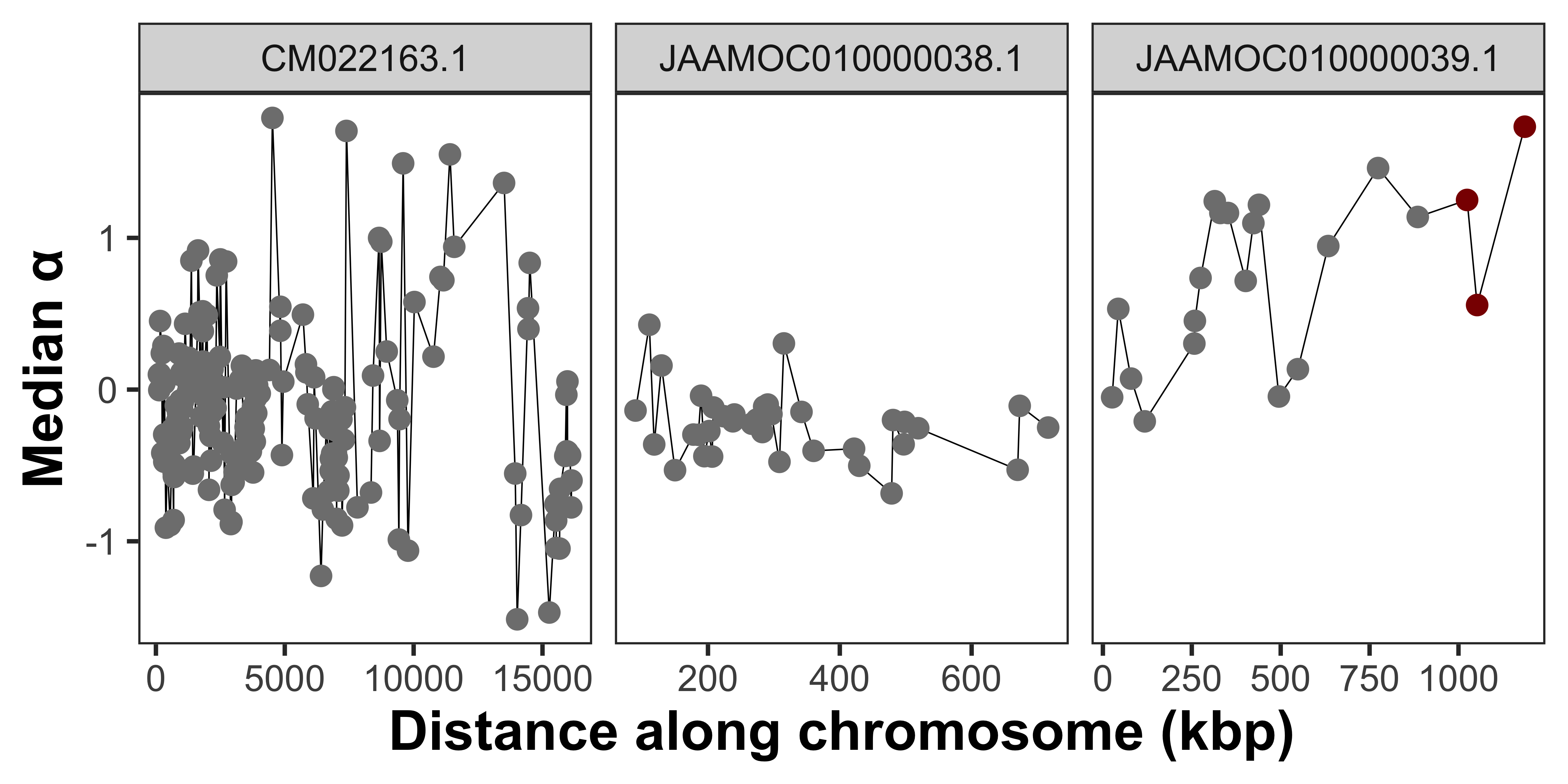

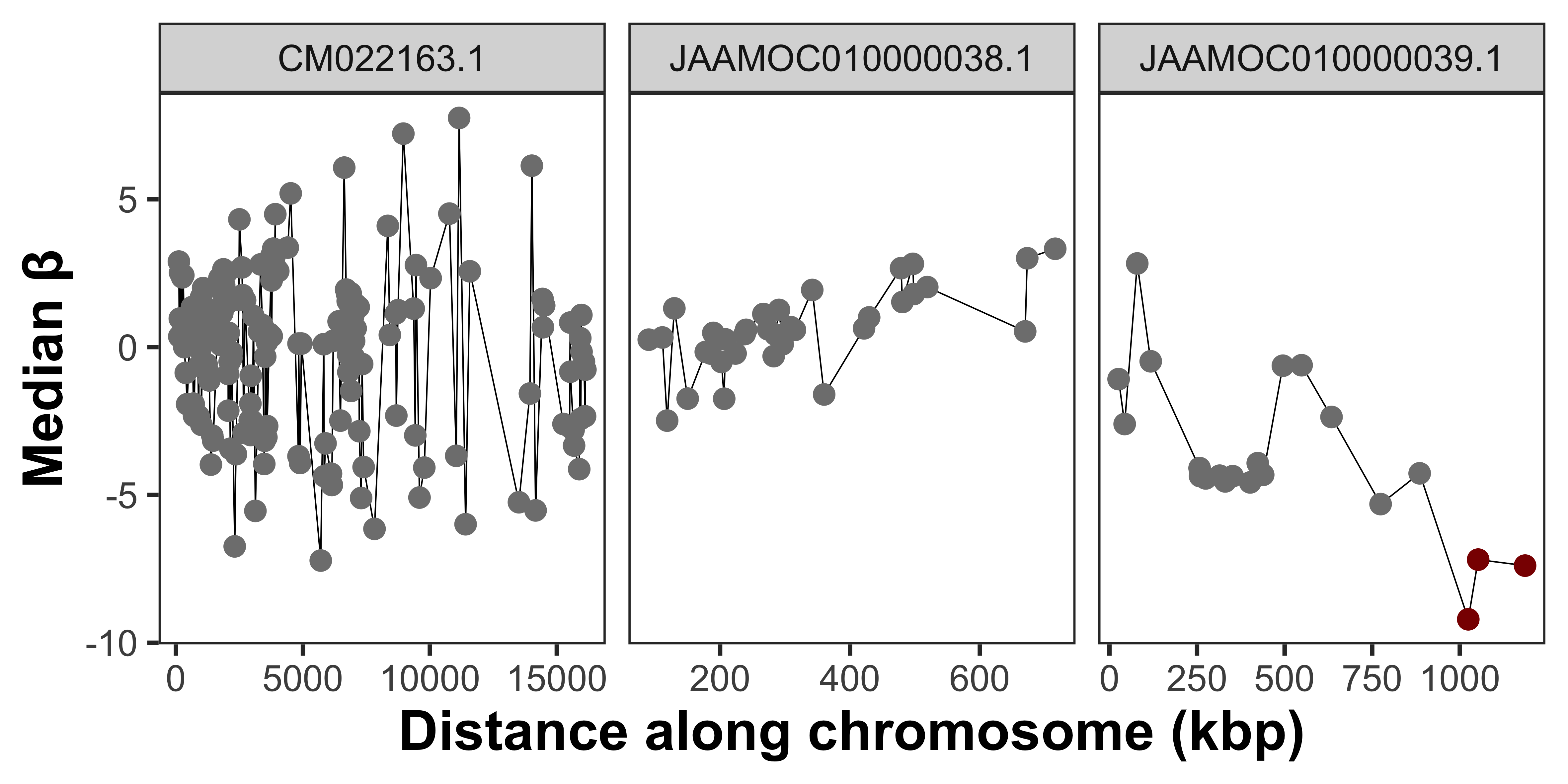
**


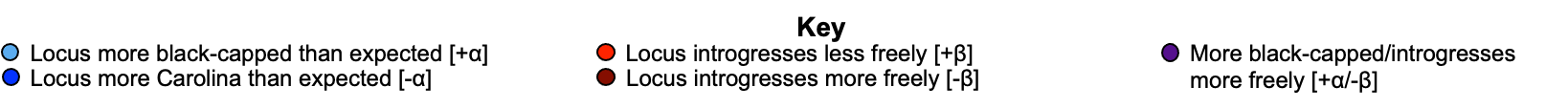
**Chromosome 14**

**
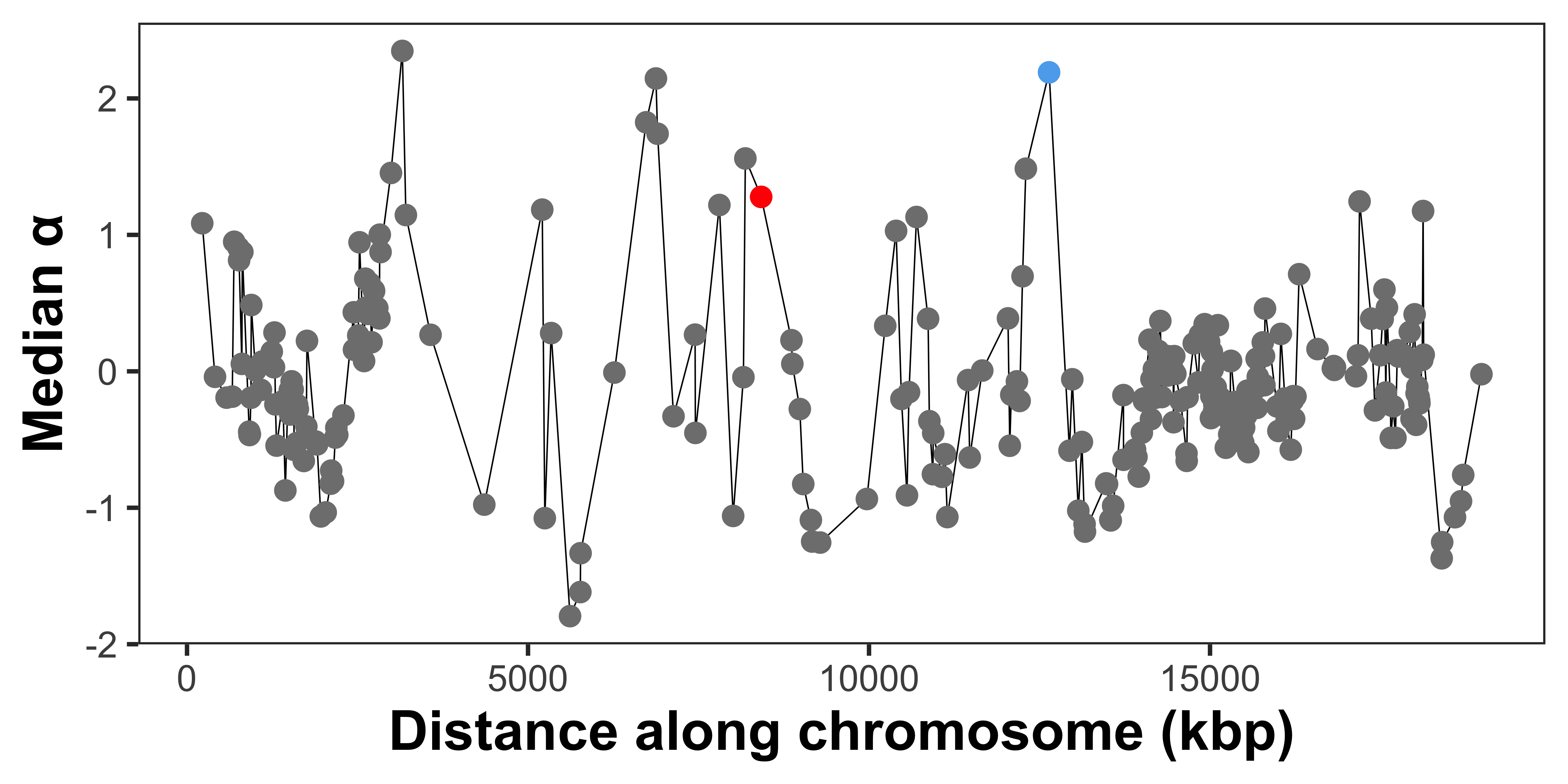

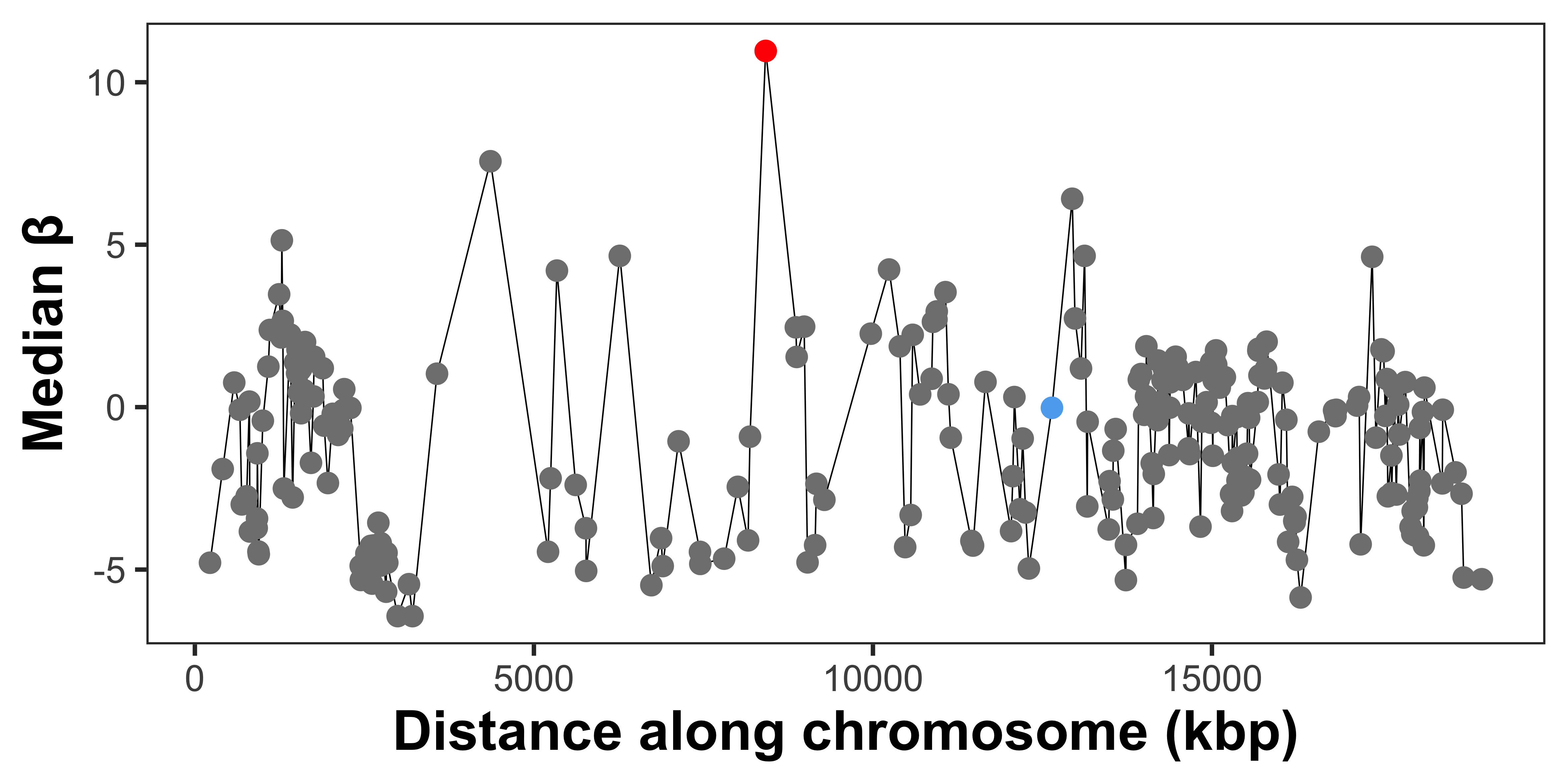
**

**Chromosome 15**

**
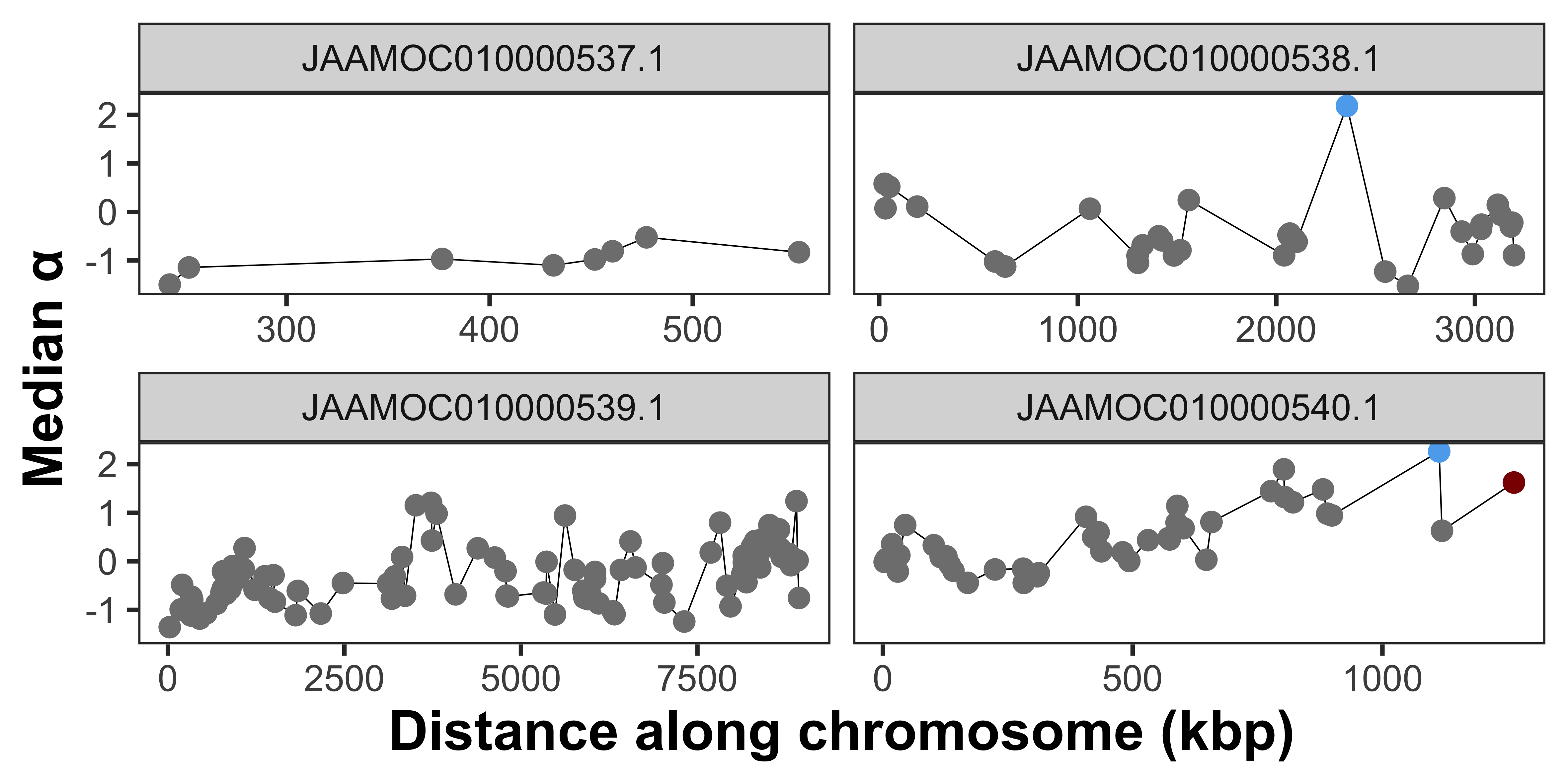

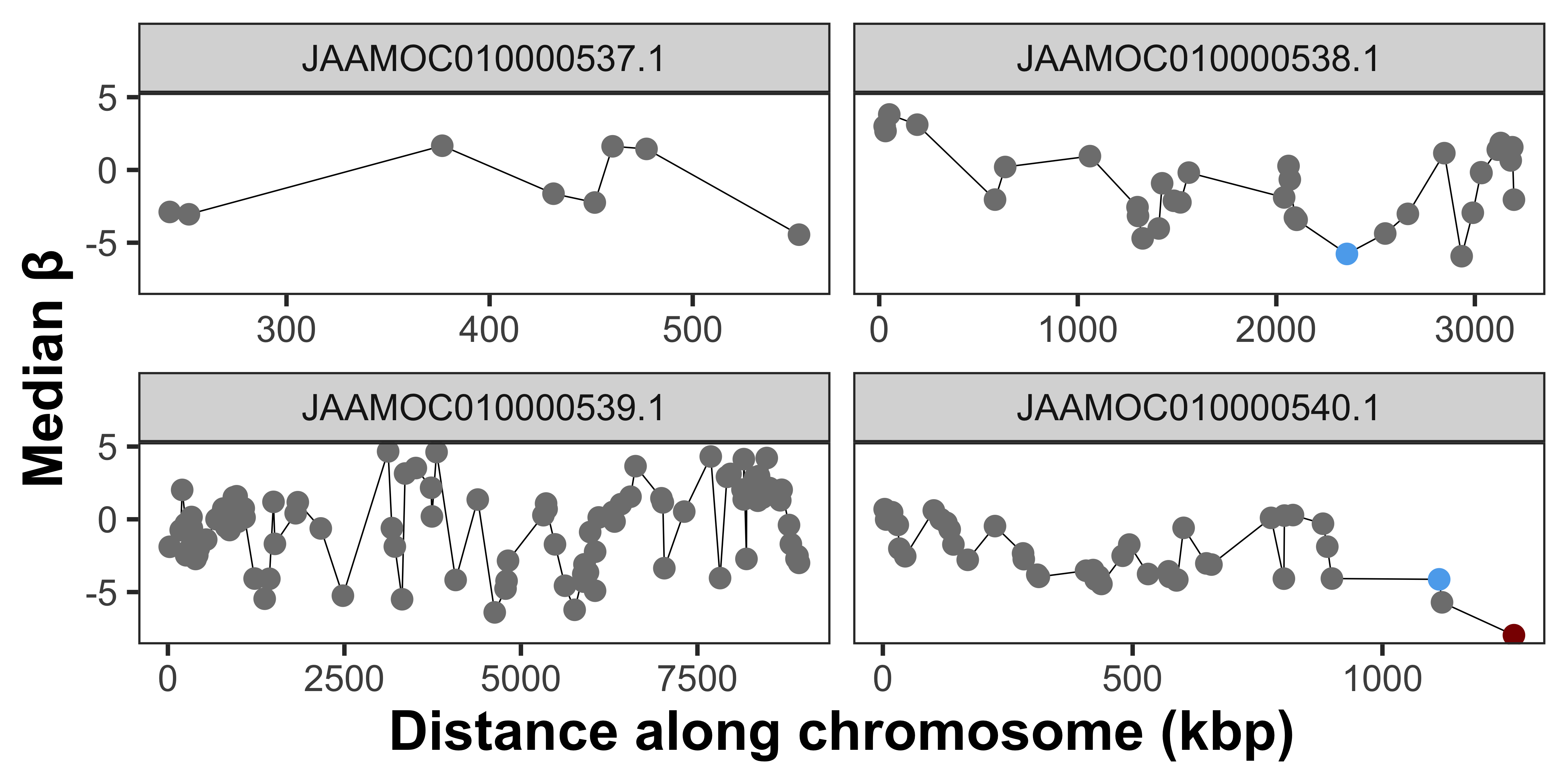
**

**Chromosome 17**

**
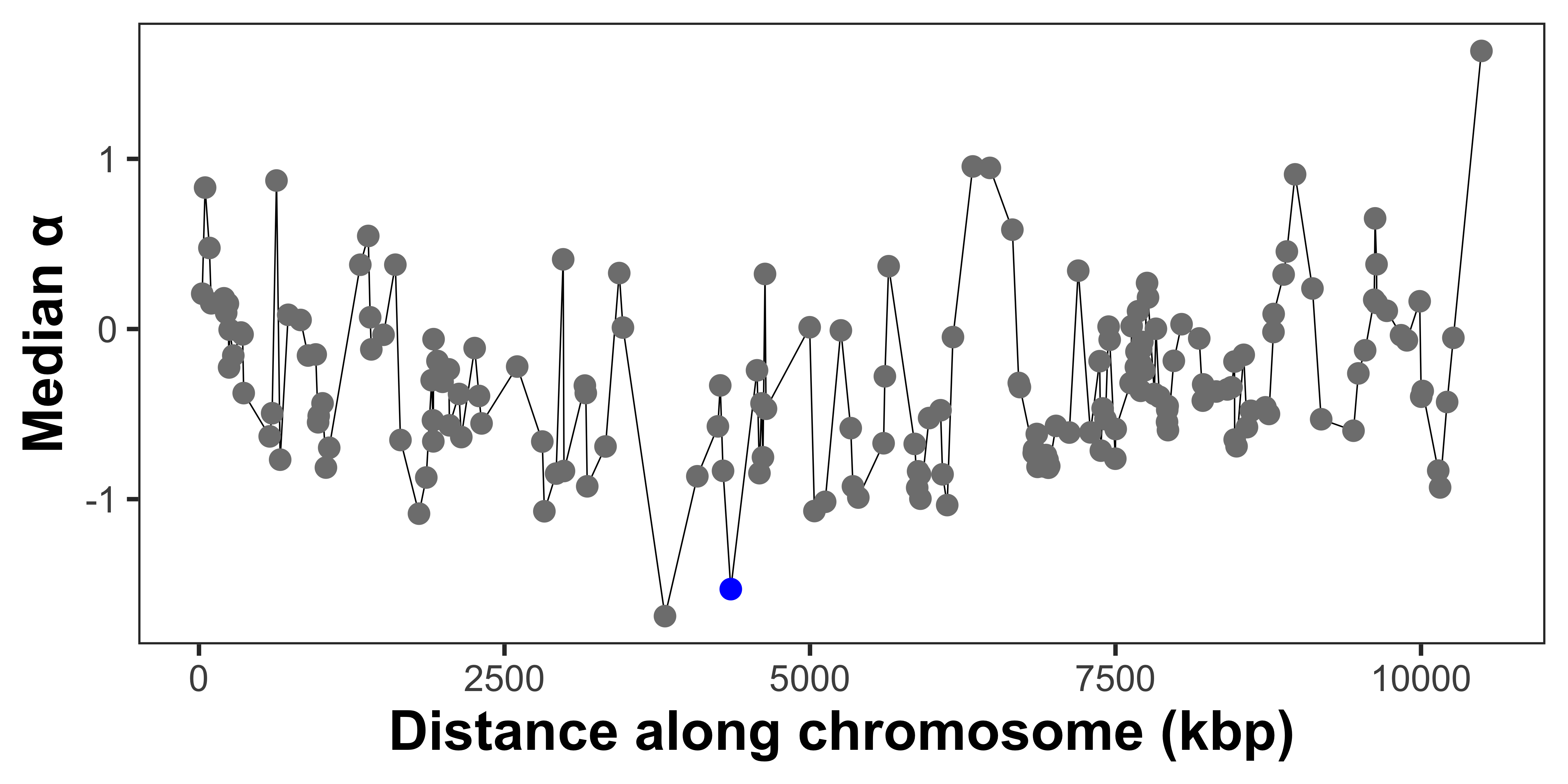

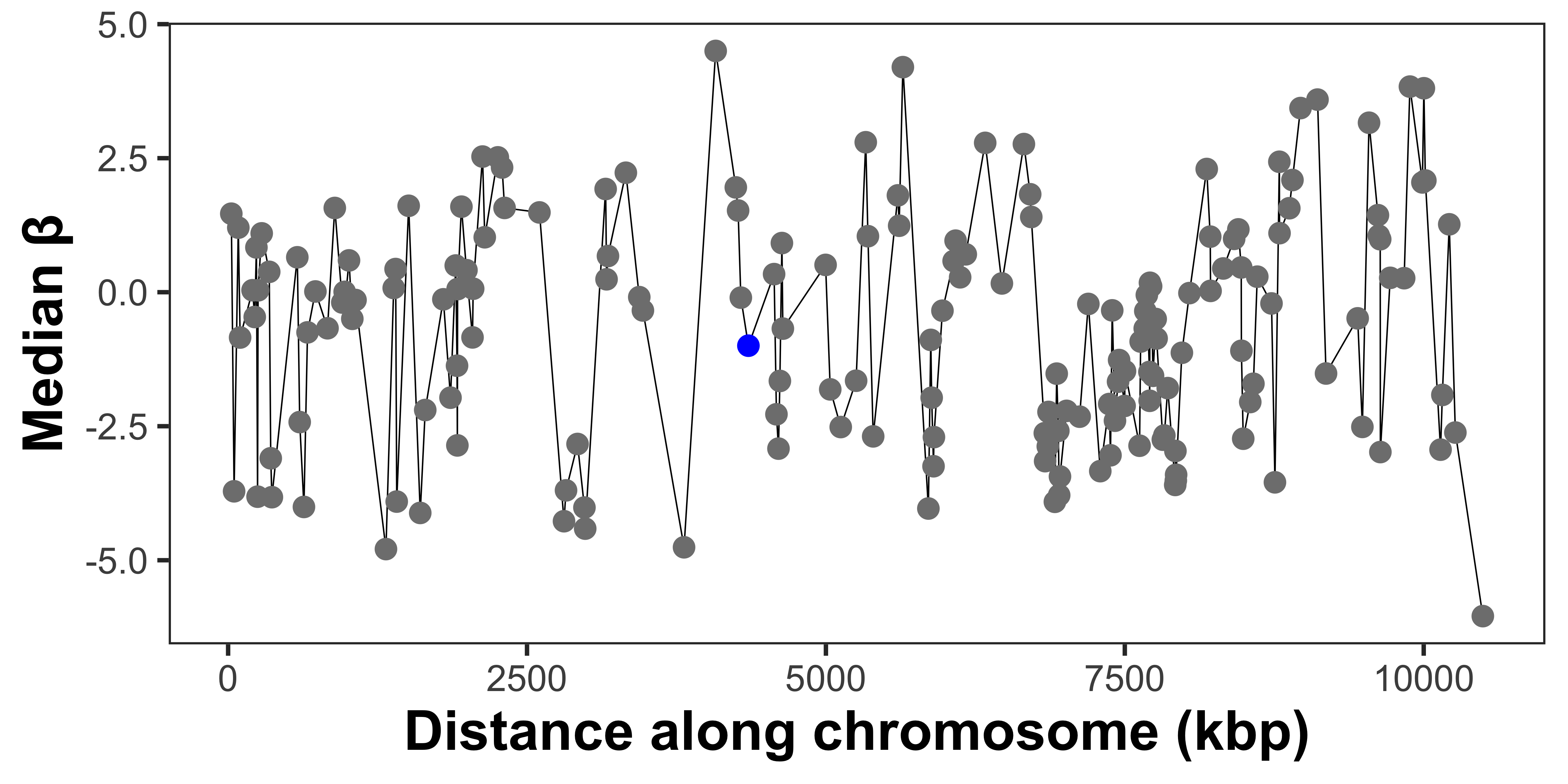
**

**Chromosome 18**

**
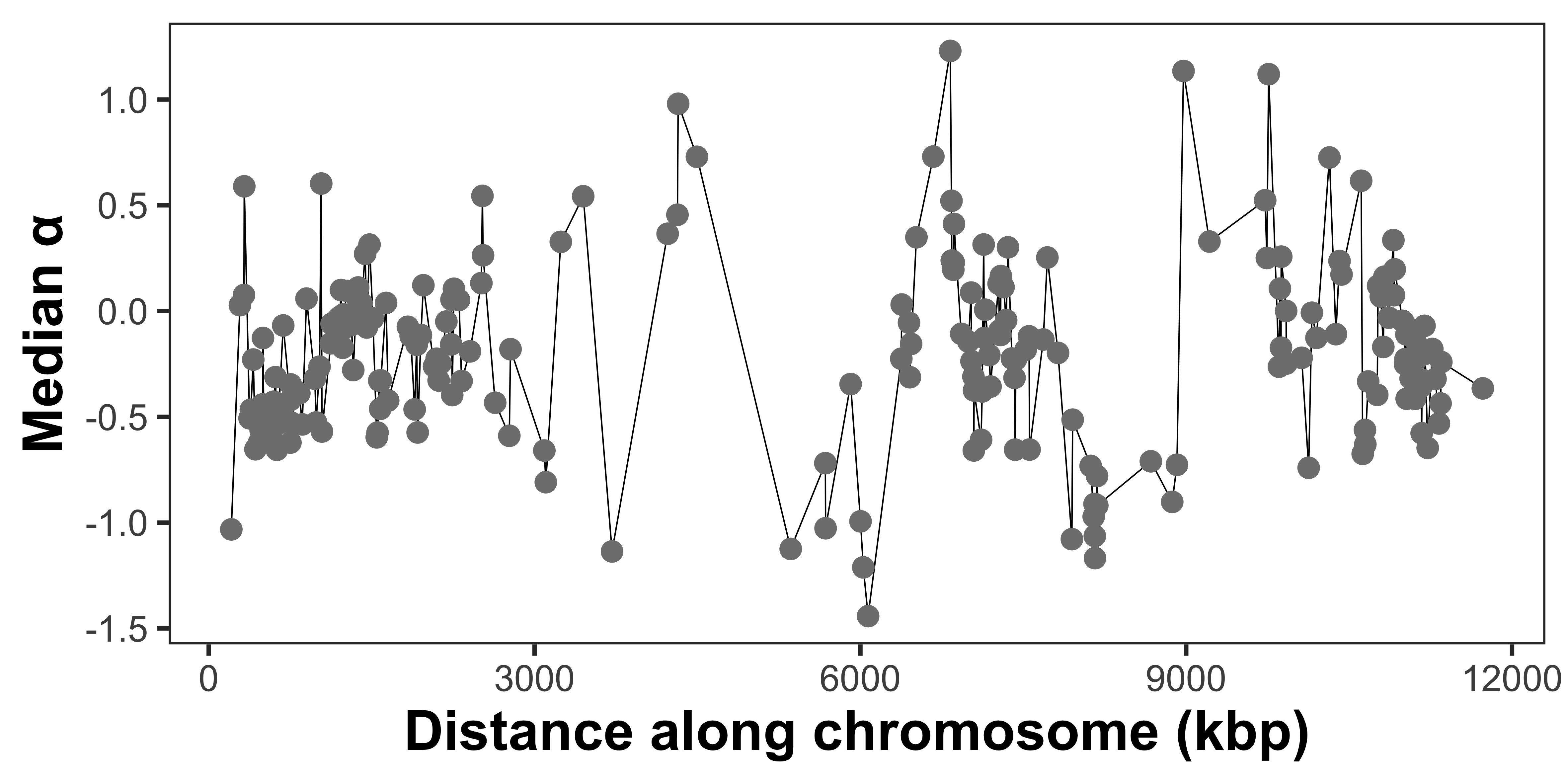

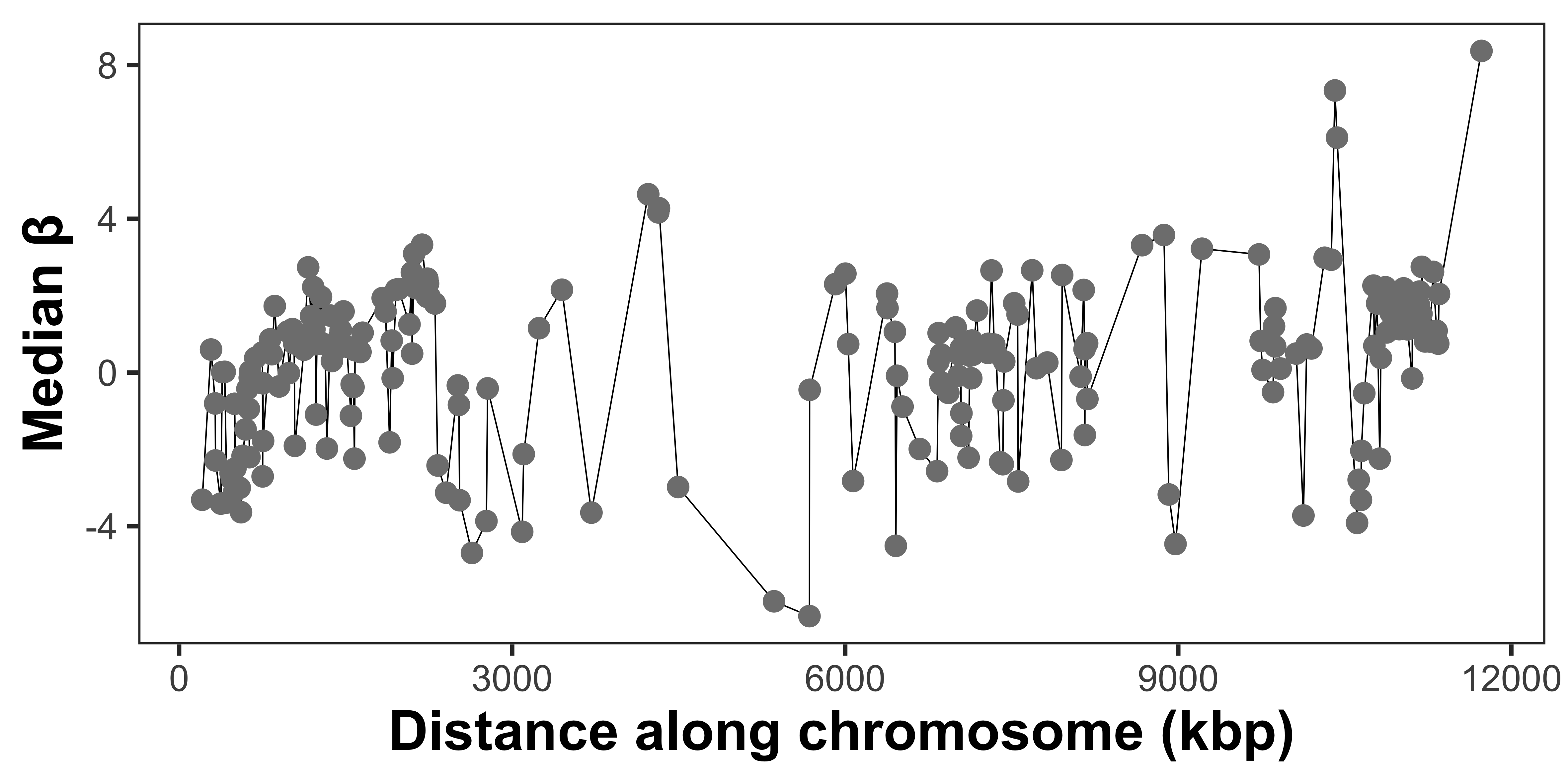
**


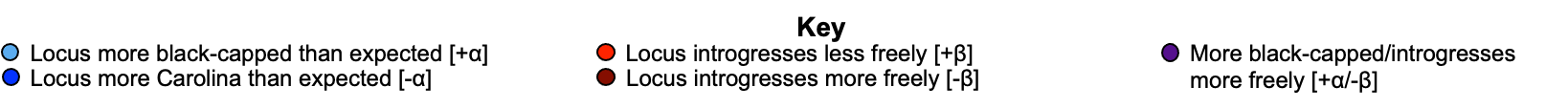
**Chromosome 19**

**
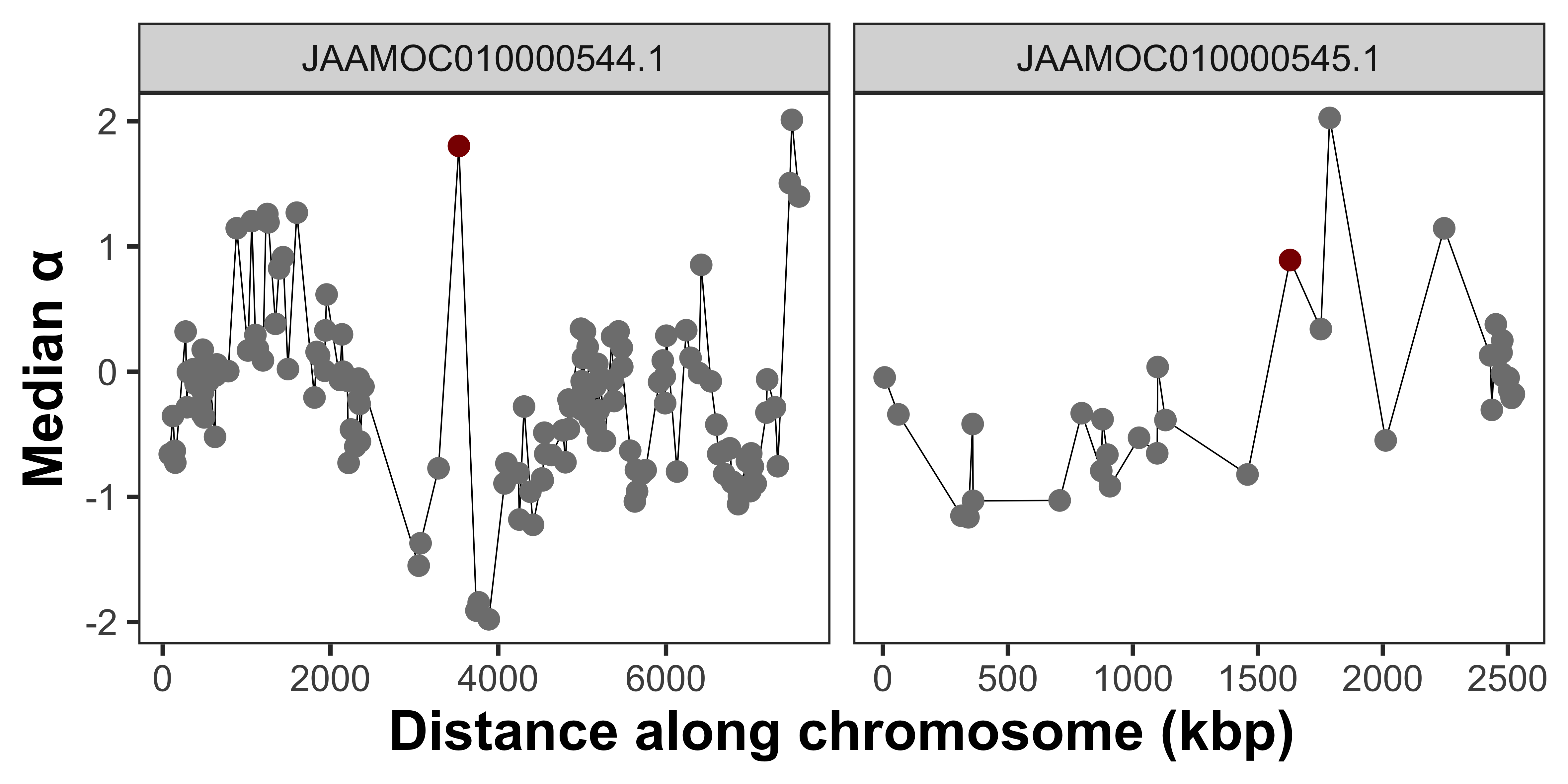

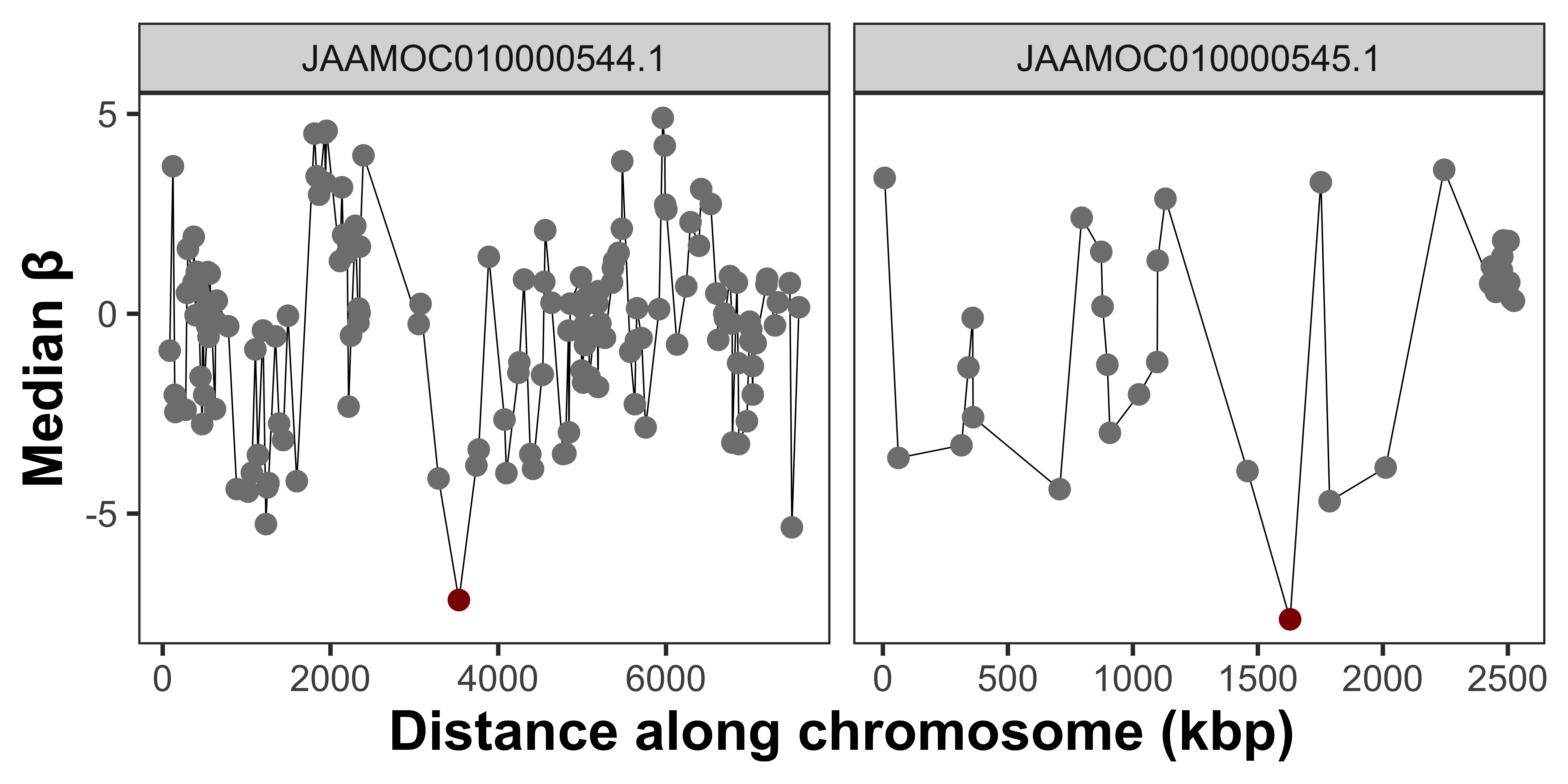
**

**Chromosome 20**

**
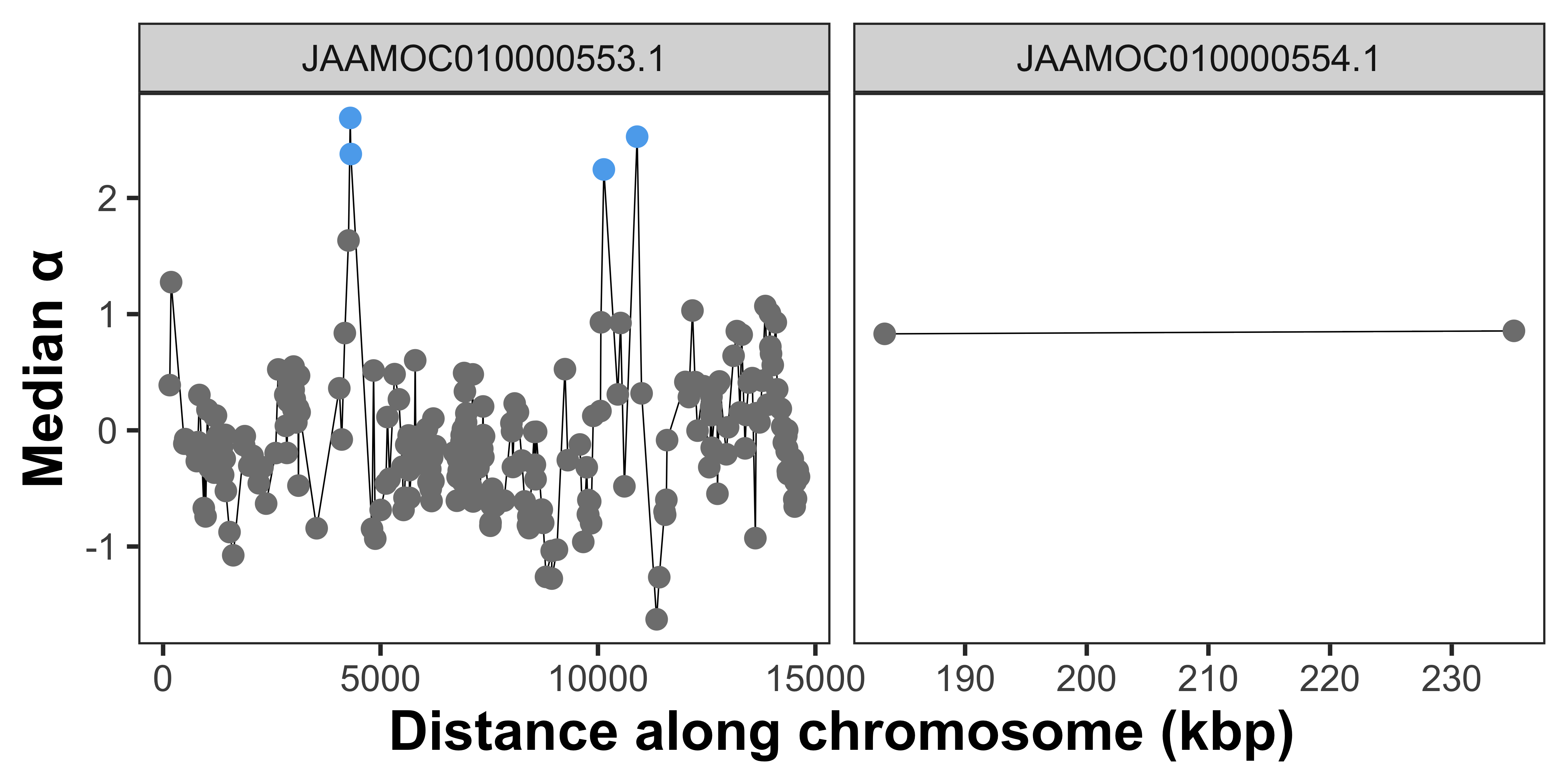

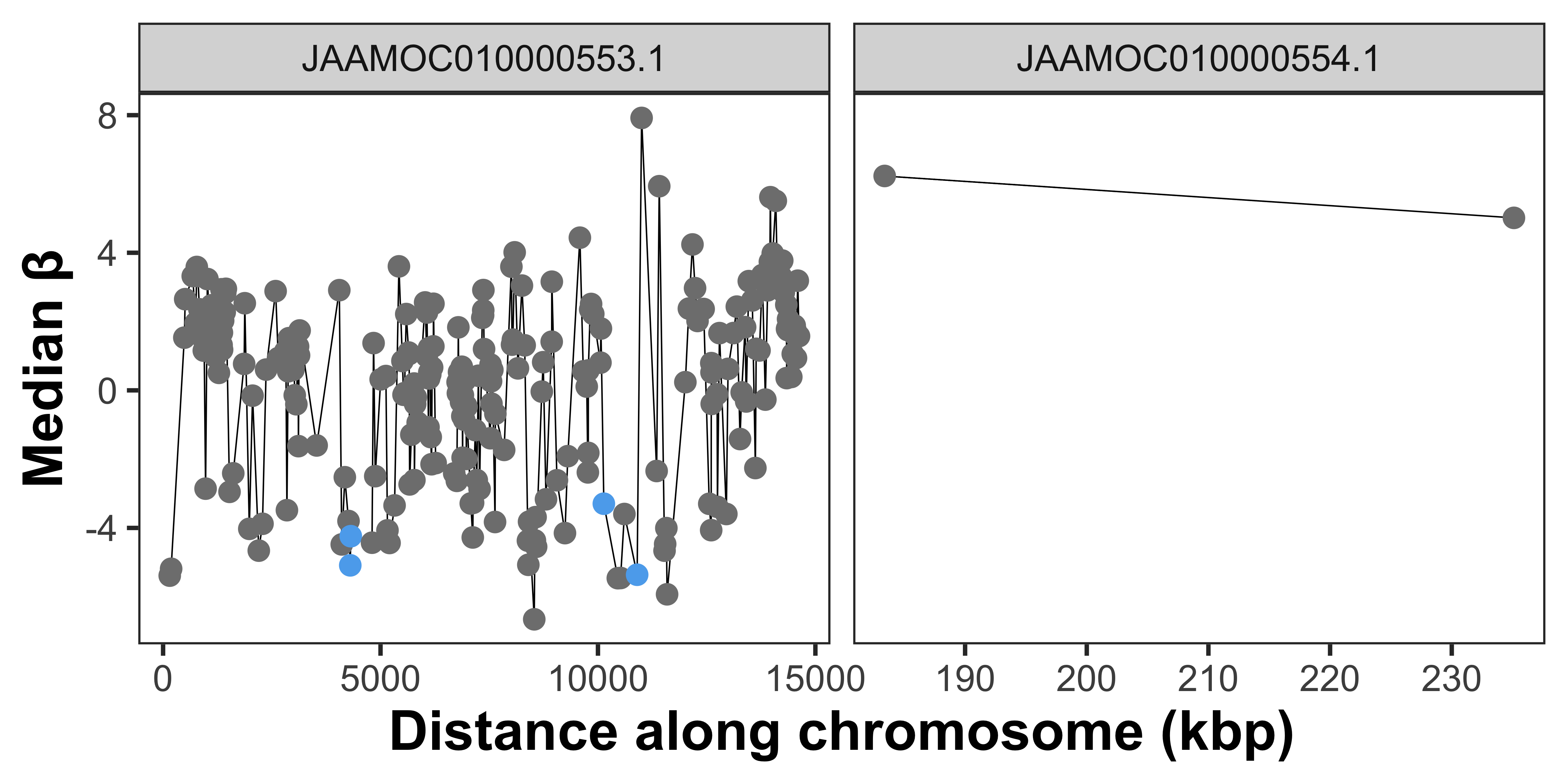
**

**Chromosome 21**

**
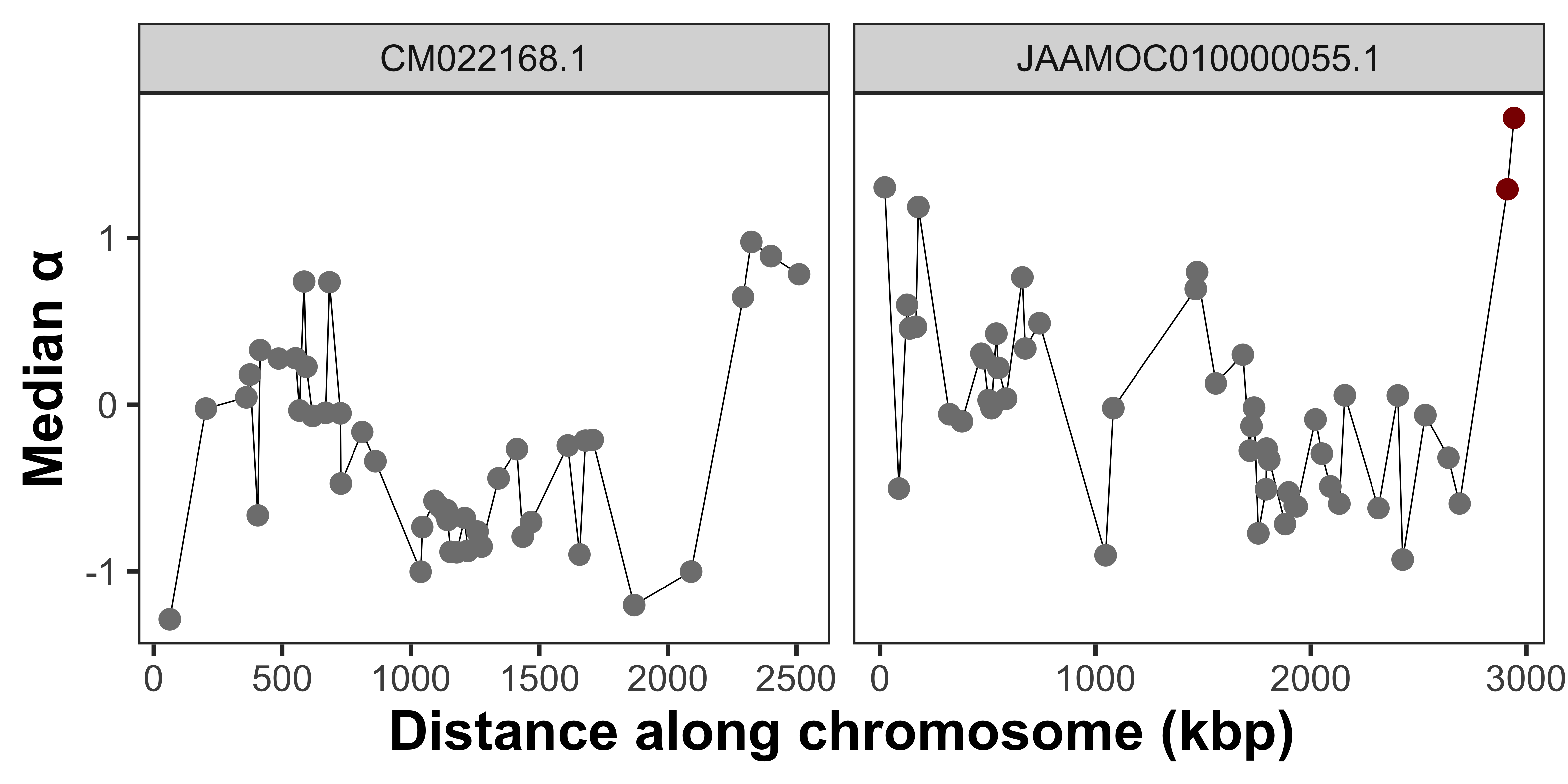

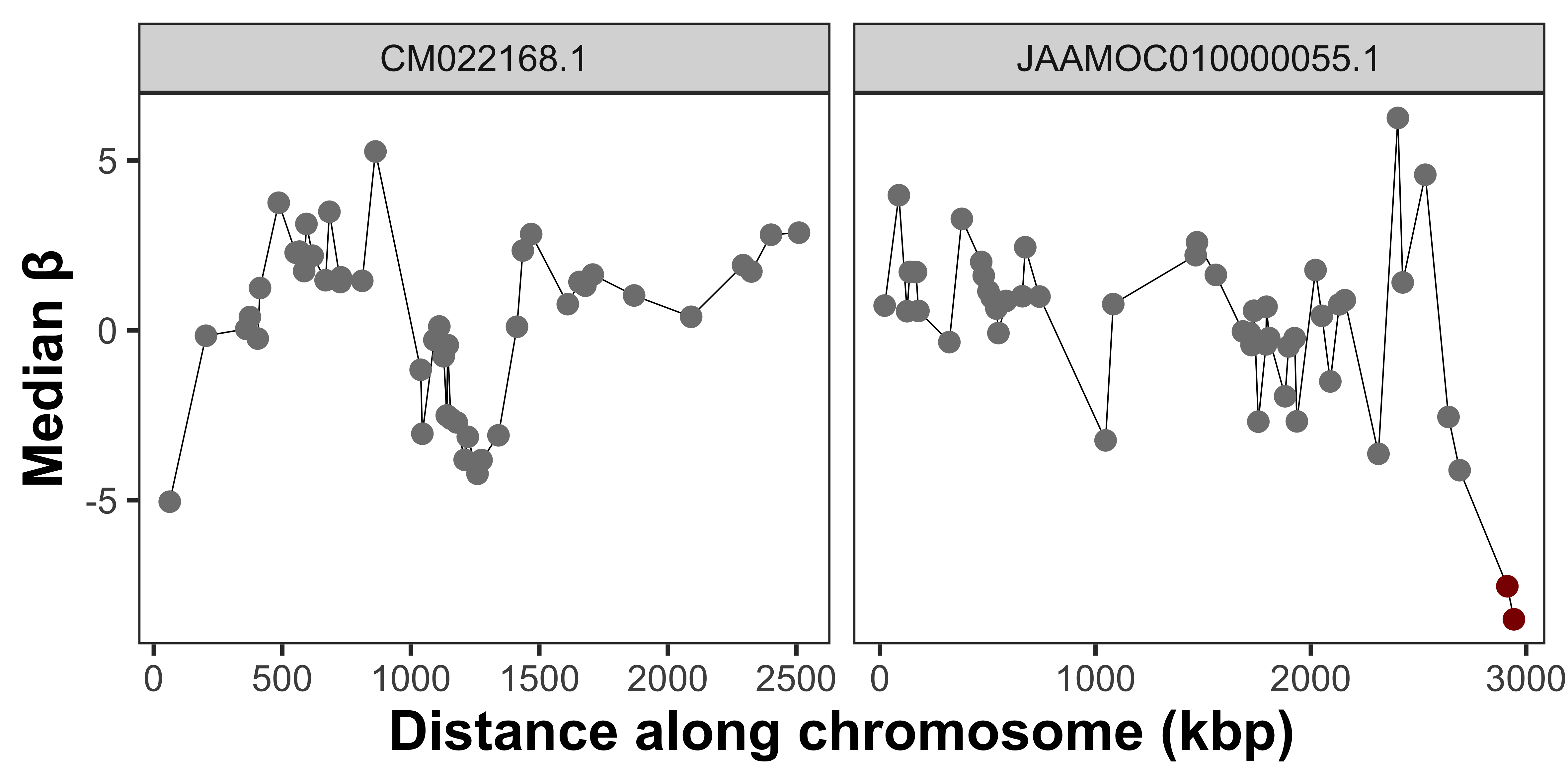
**

**Chromosome 22:** JAAMOC010000061.1 not displayed as only one RAD marker present

**
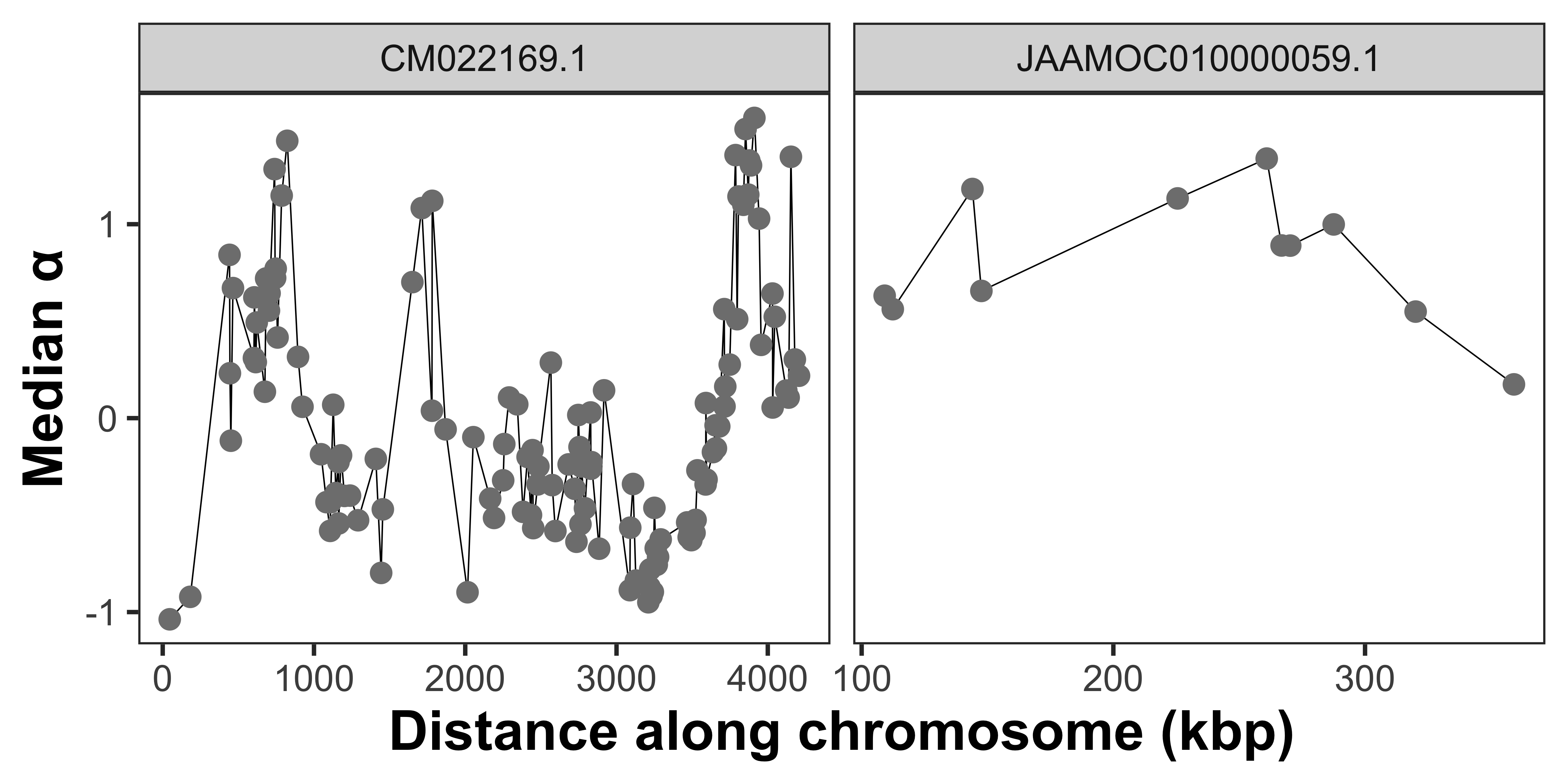

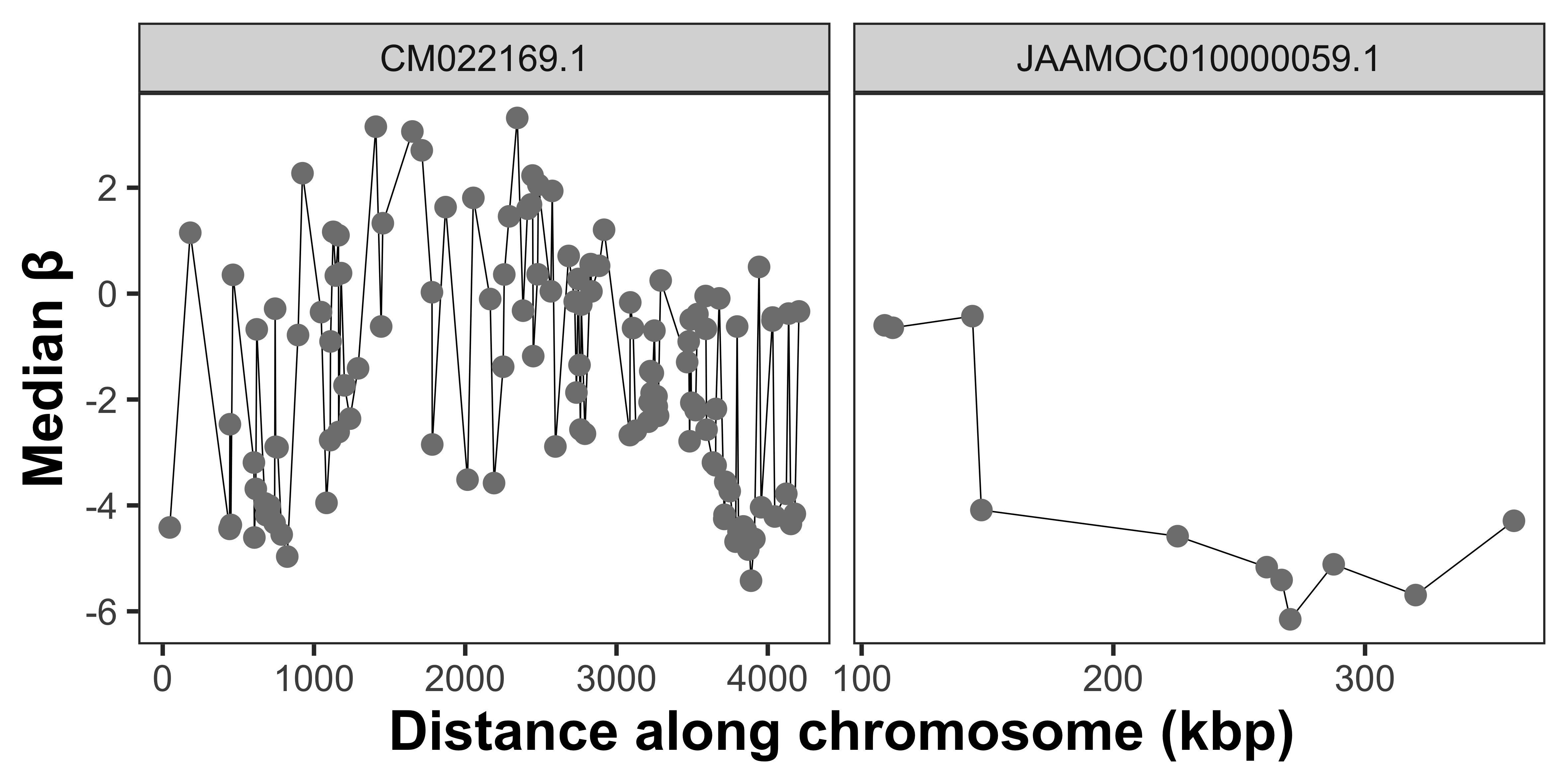
**


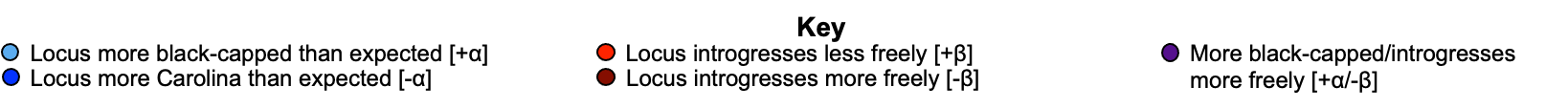


**Chromosome 23:** JAAMOC010000063.1 and JAAMOC010000065.1 not displayed as only one RAD marker present

**
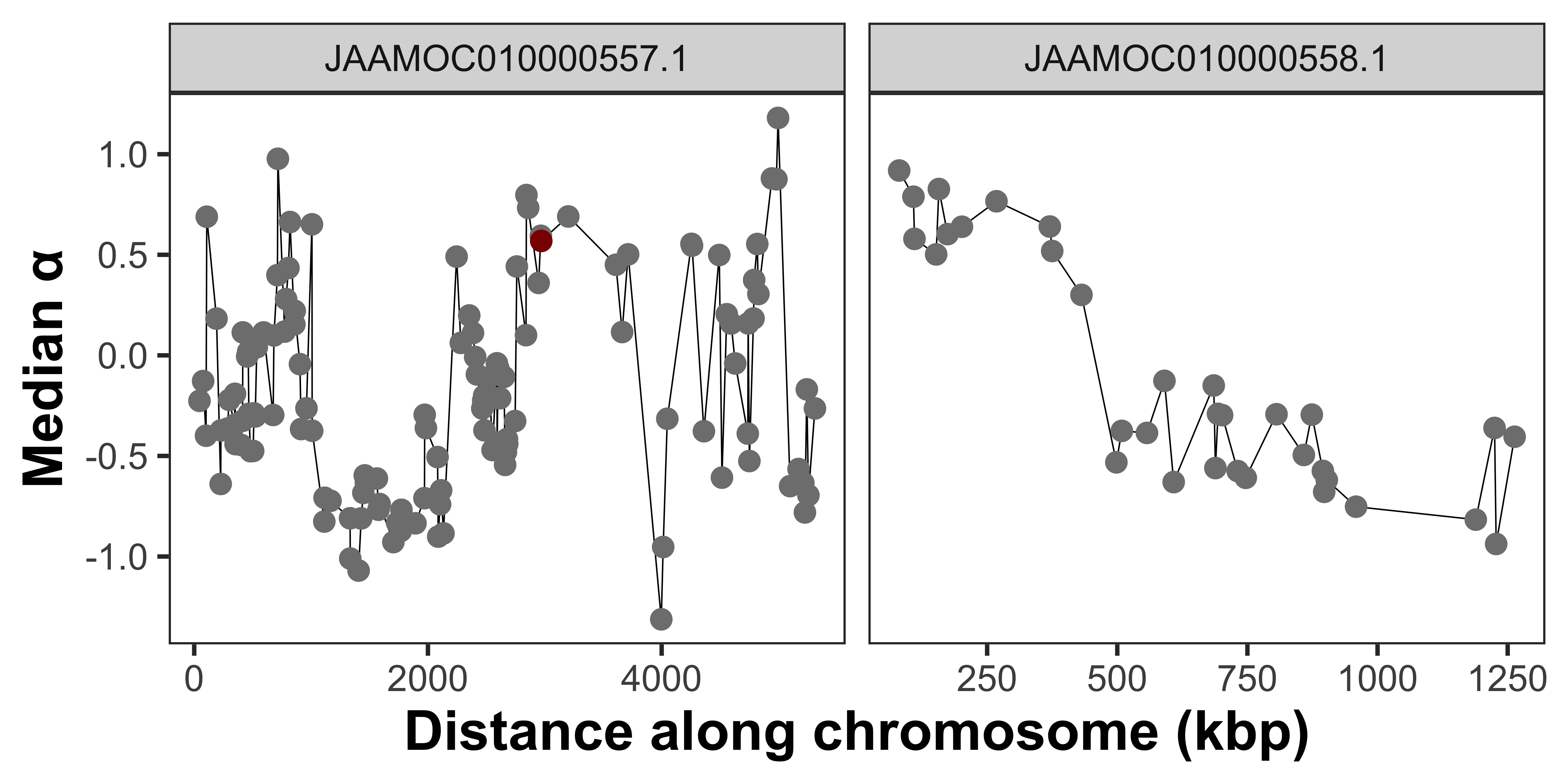

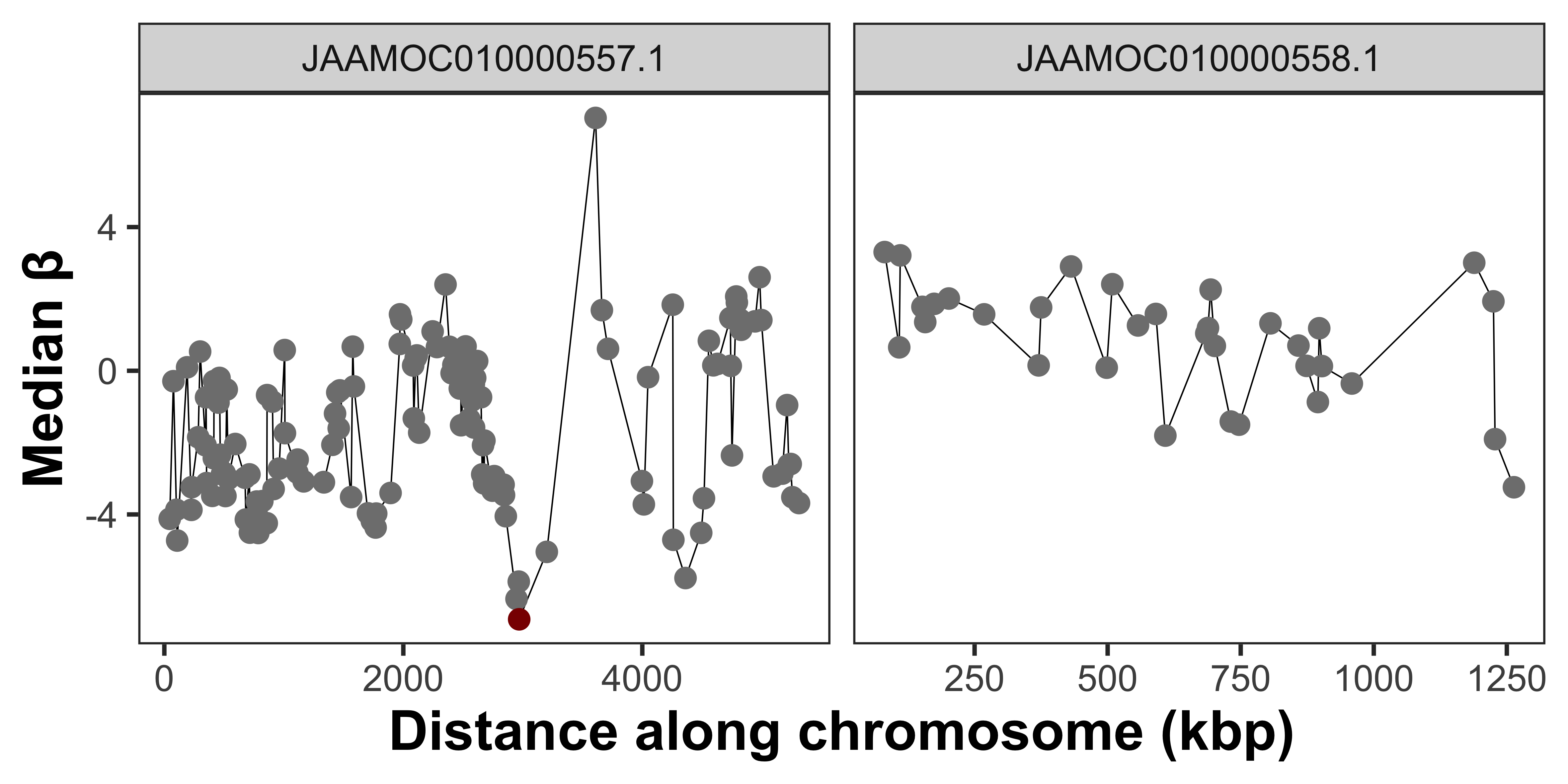
**

**Chromosome 24**

**
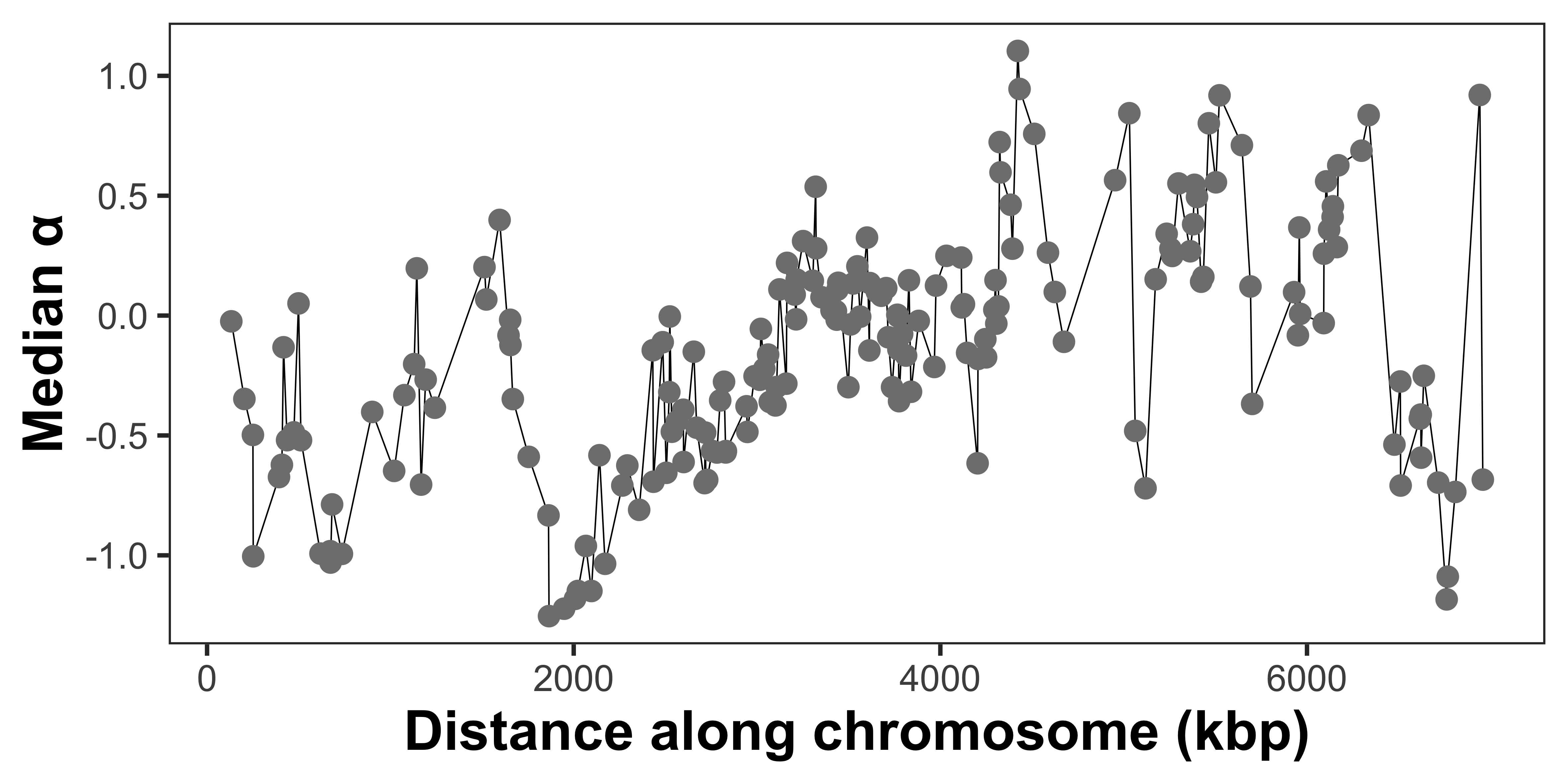

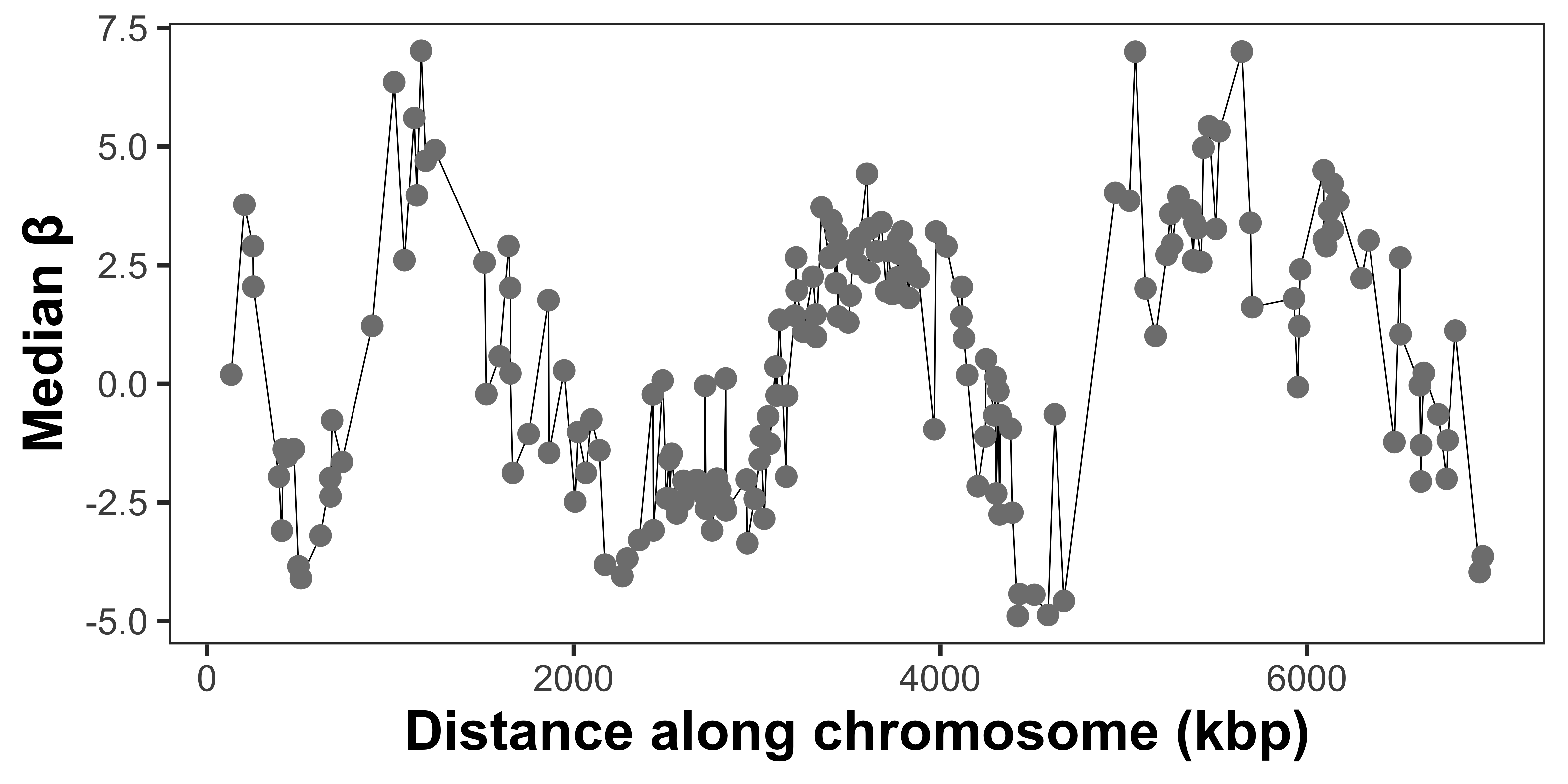
**

**Chromosome 25:** JAAMOC010000074.1 not displayed as only one RAD marker present

**
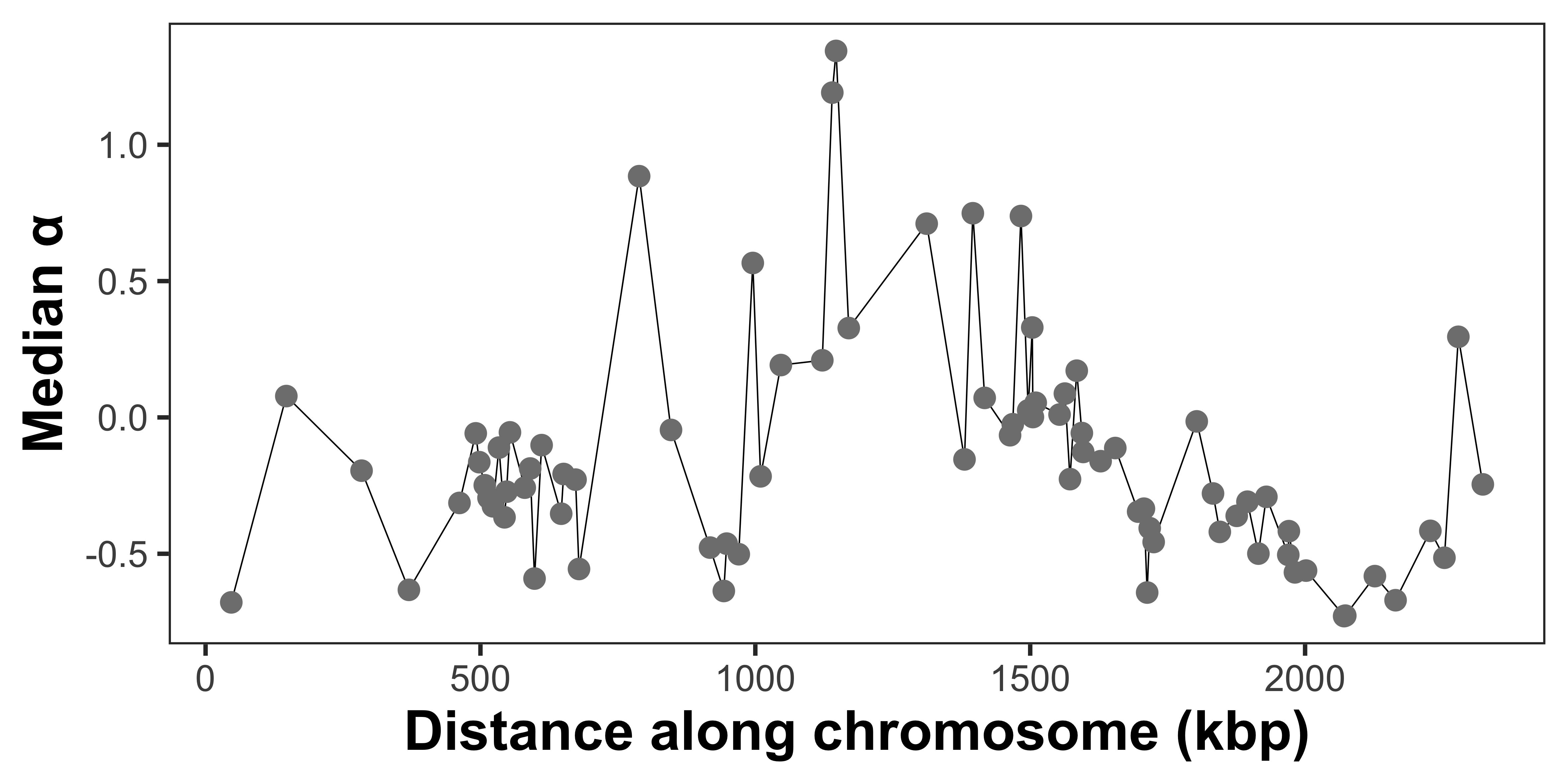

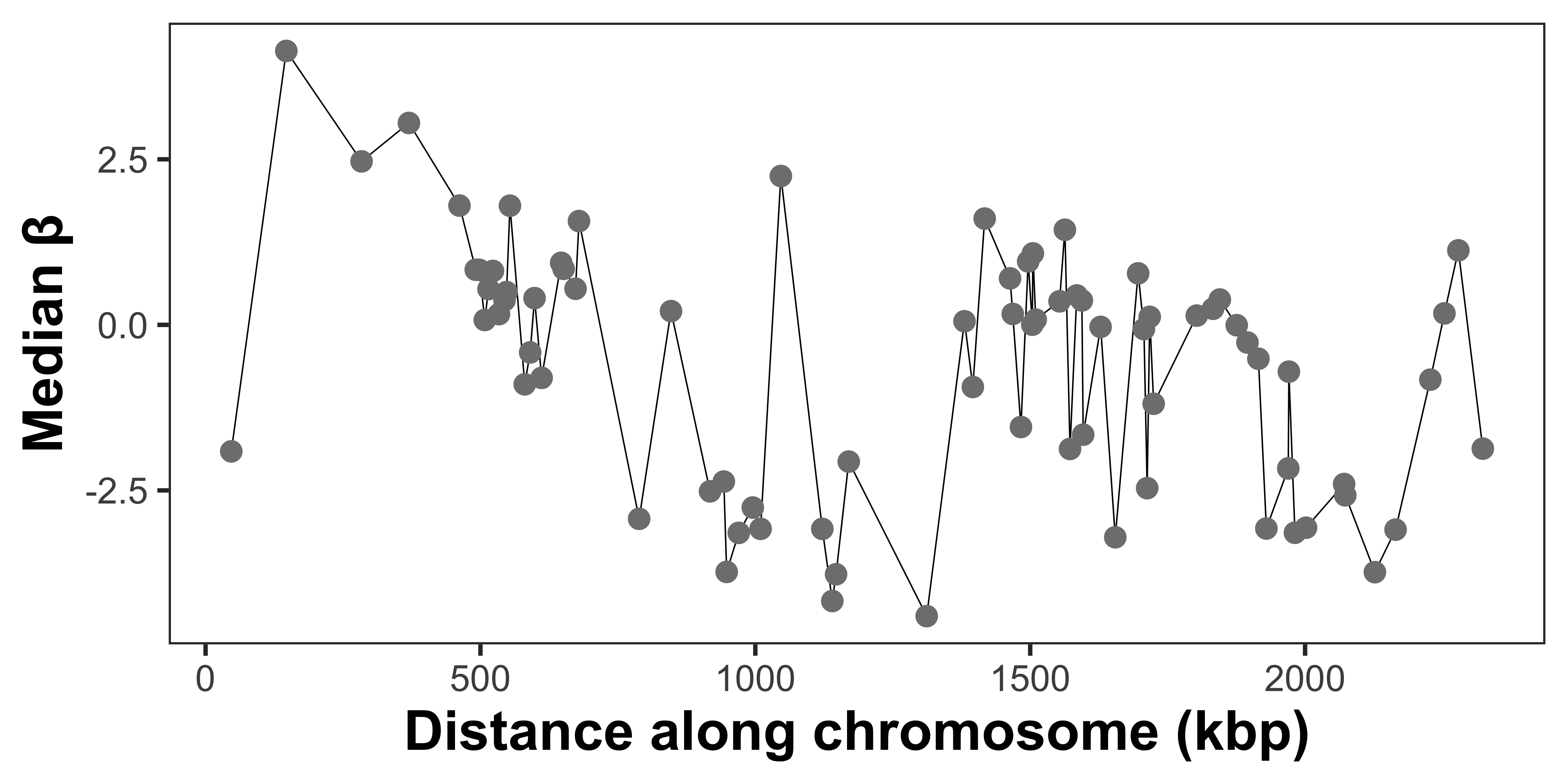
**

**Chromosome 26**

**
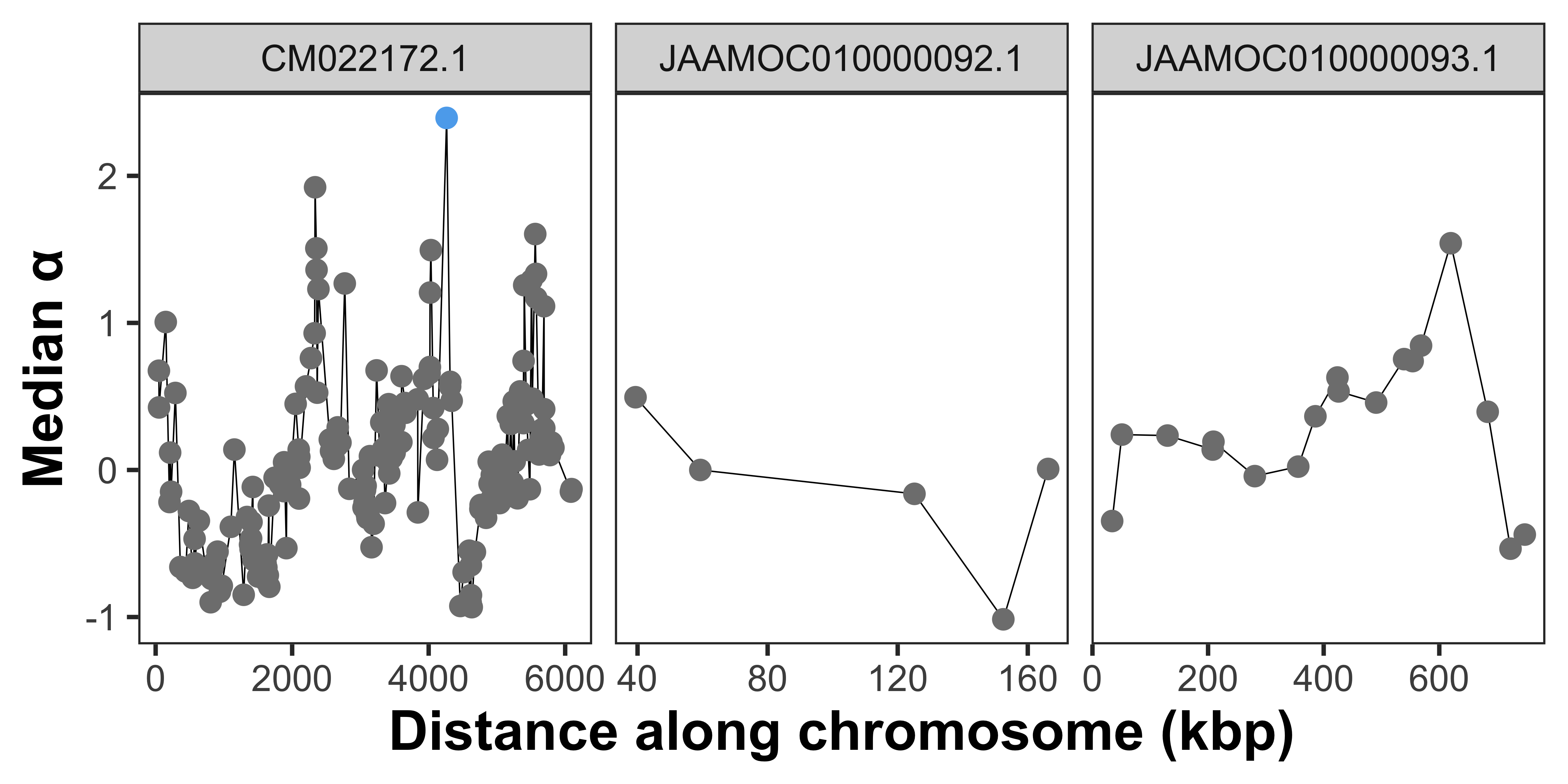

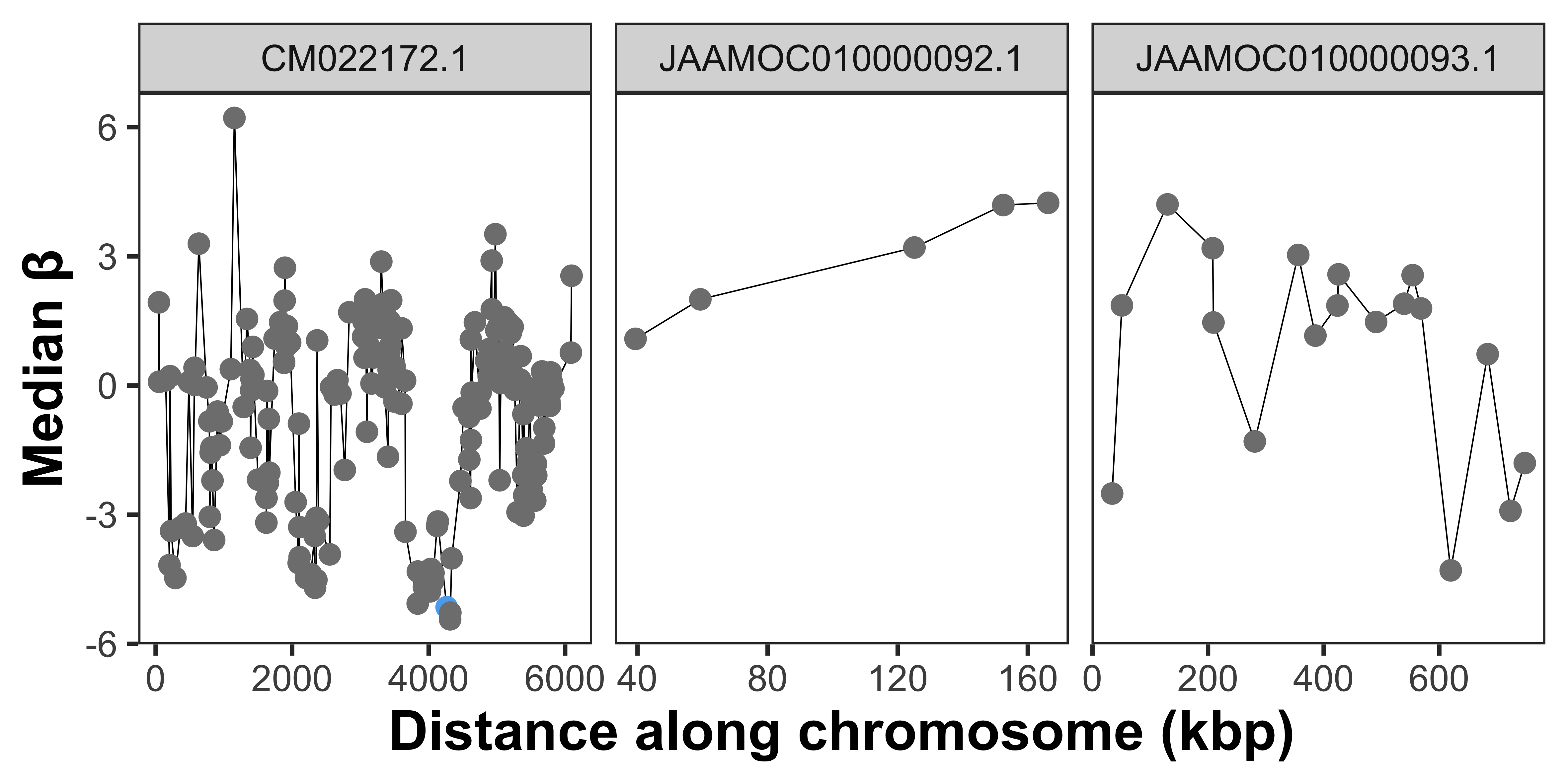
**


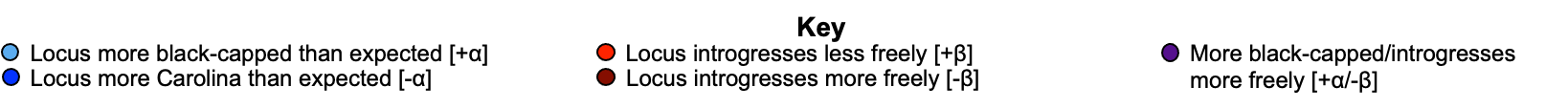


**Chromosome 27**

**
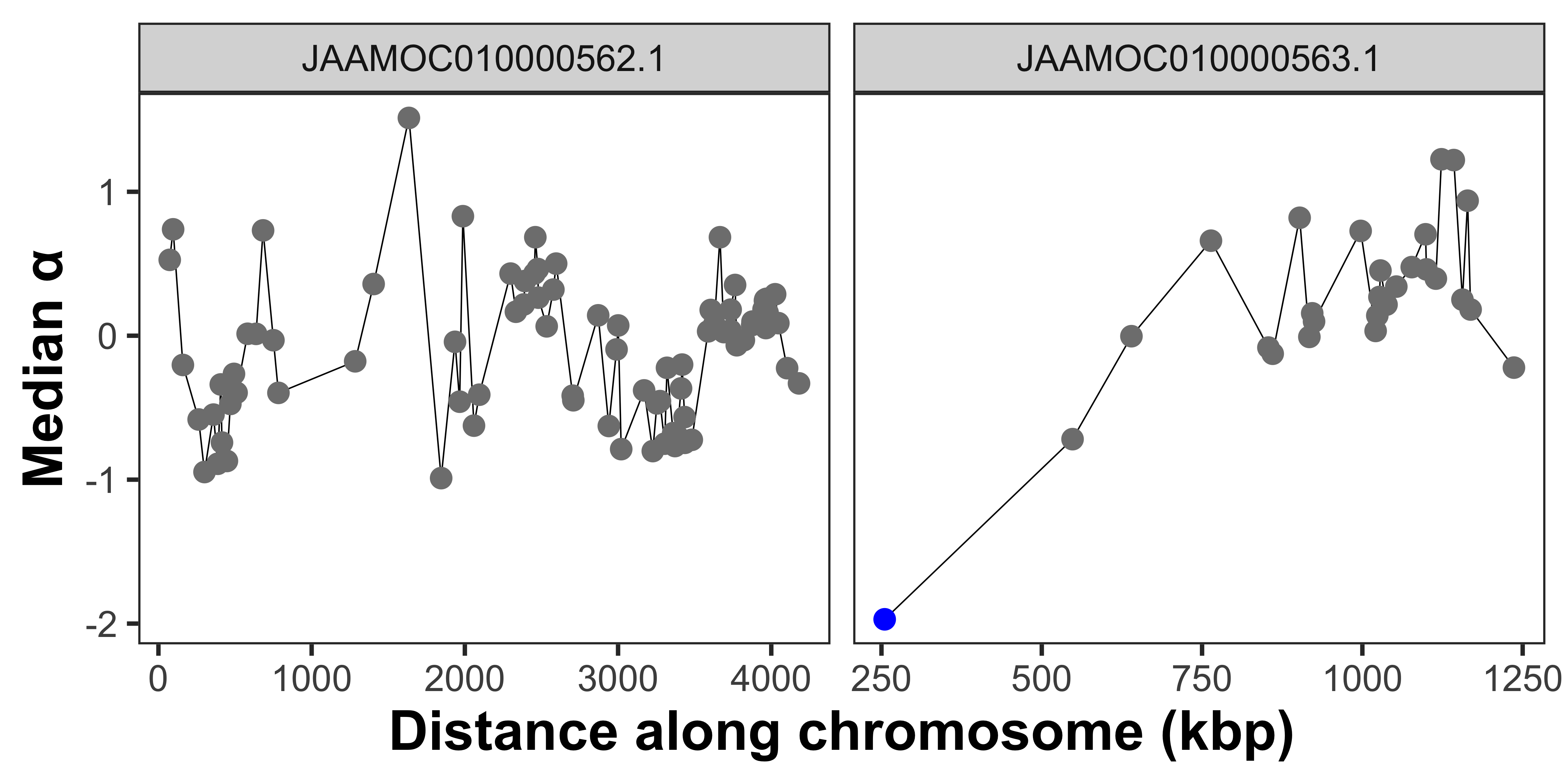

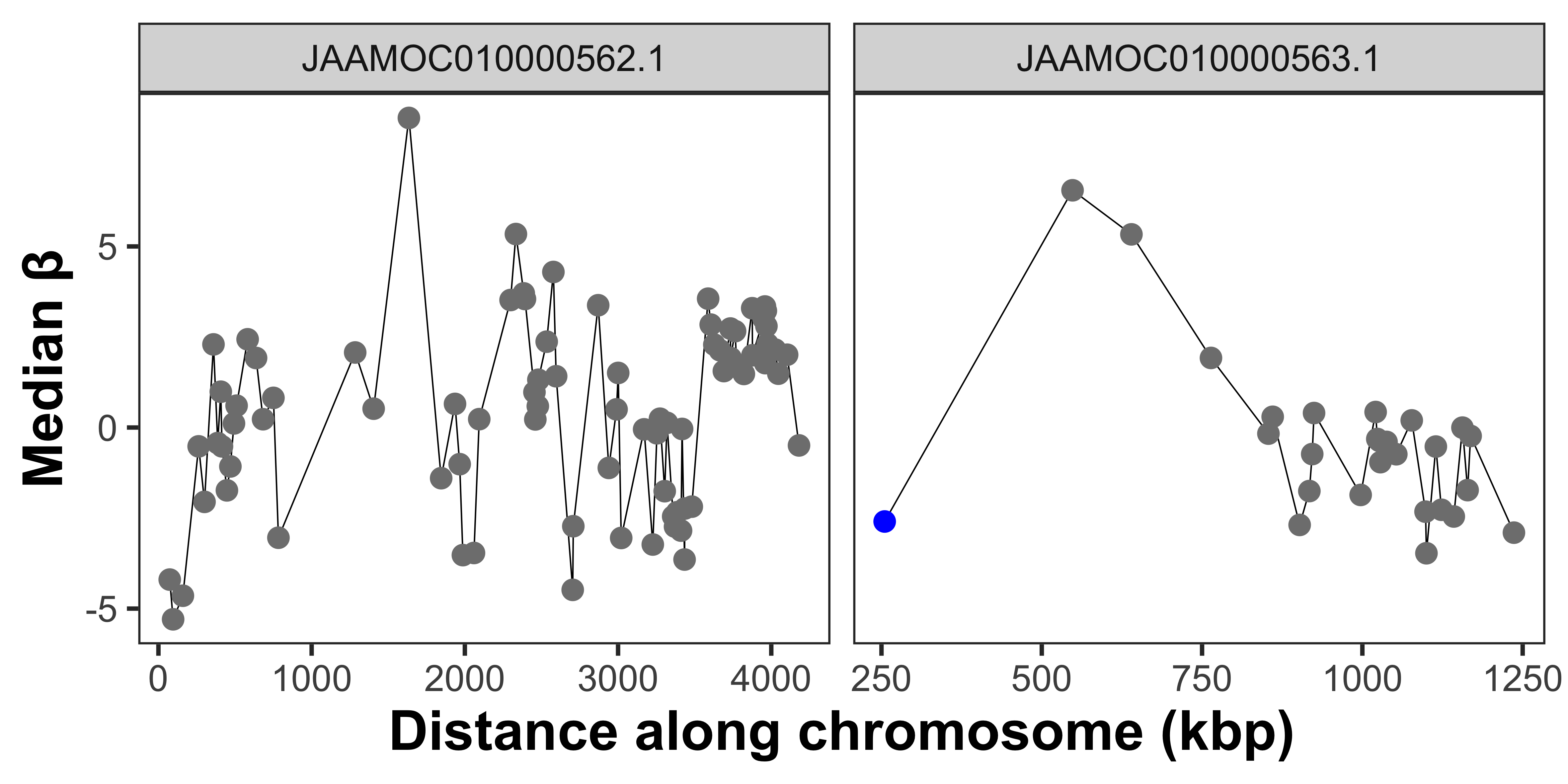
**

**Chromosome 28:** JAAMOC010000095.1 not displayed as only one RAD marker present

**
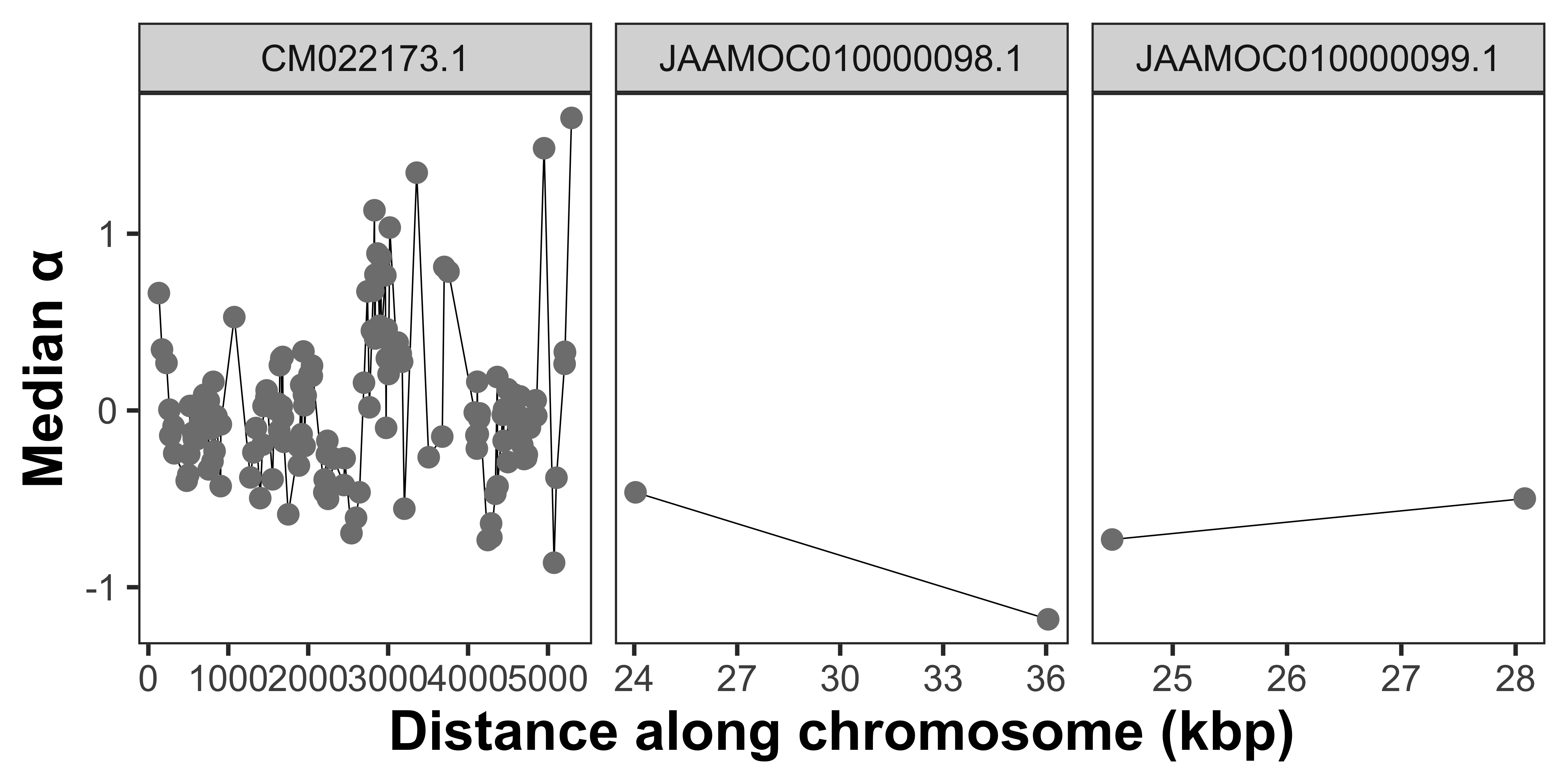

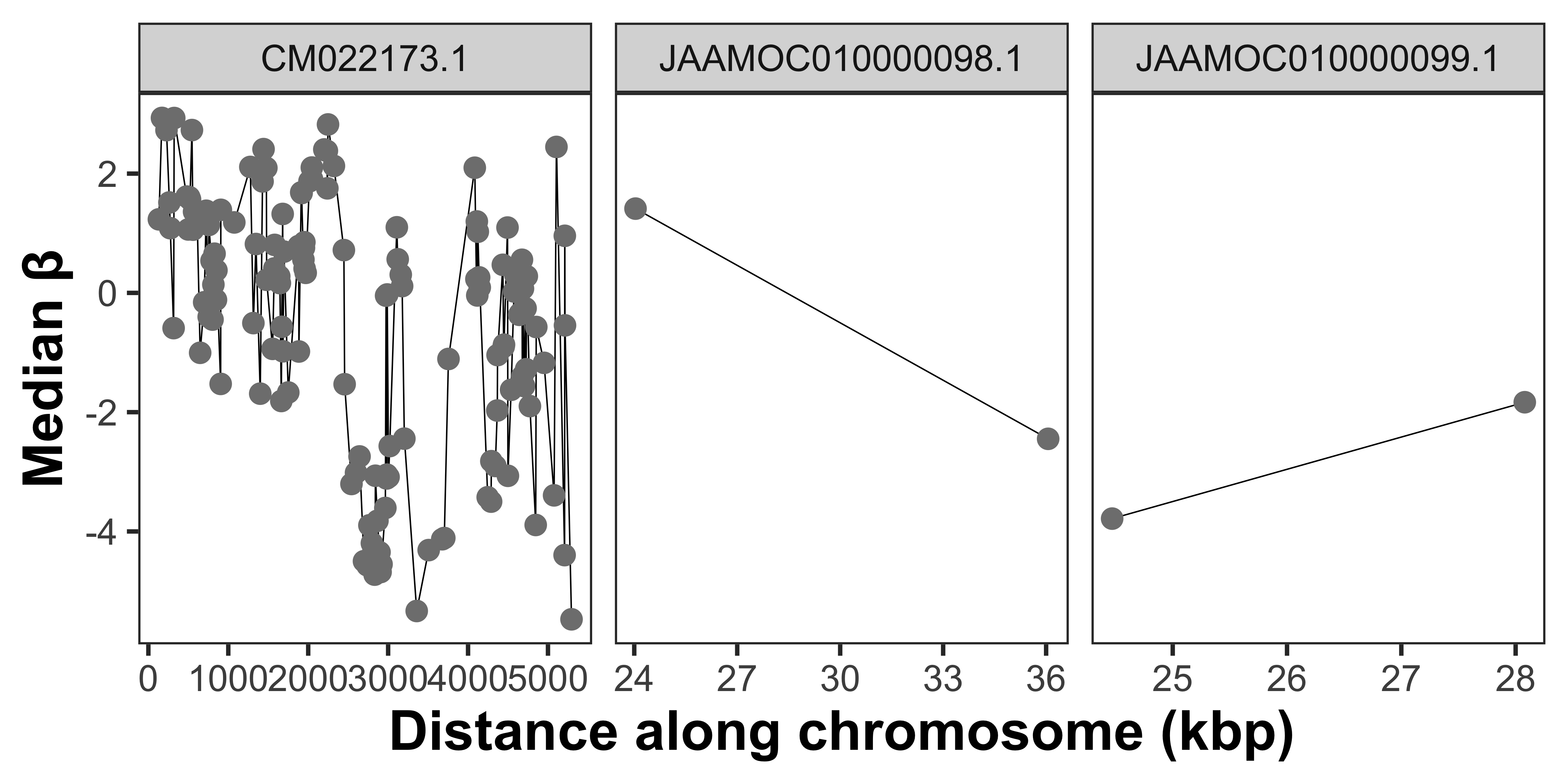
**

**Chromosome Z**


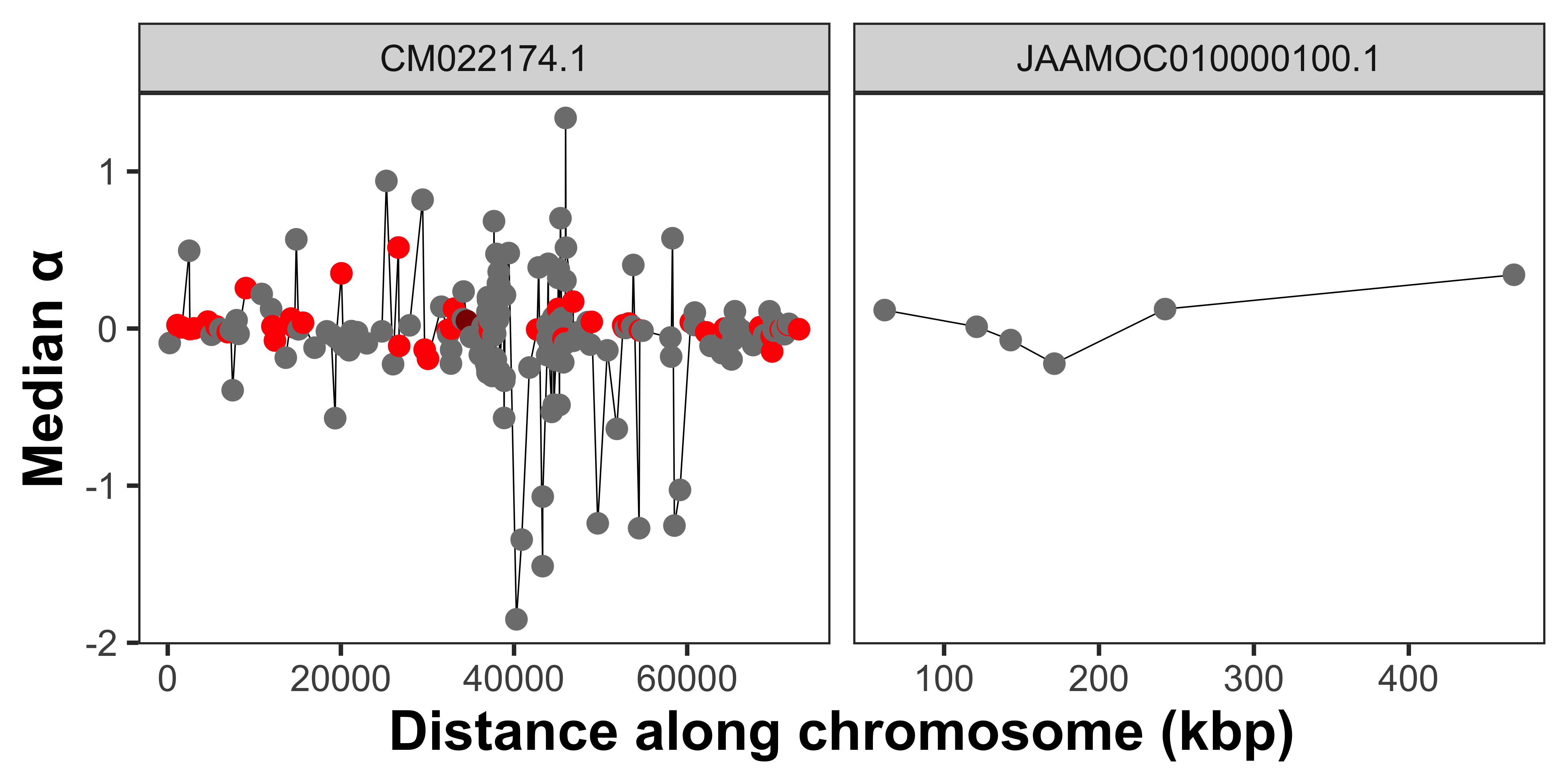

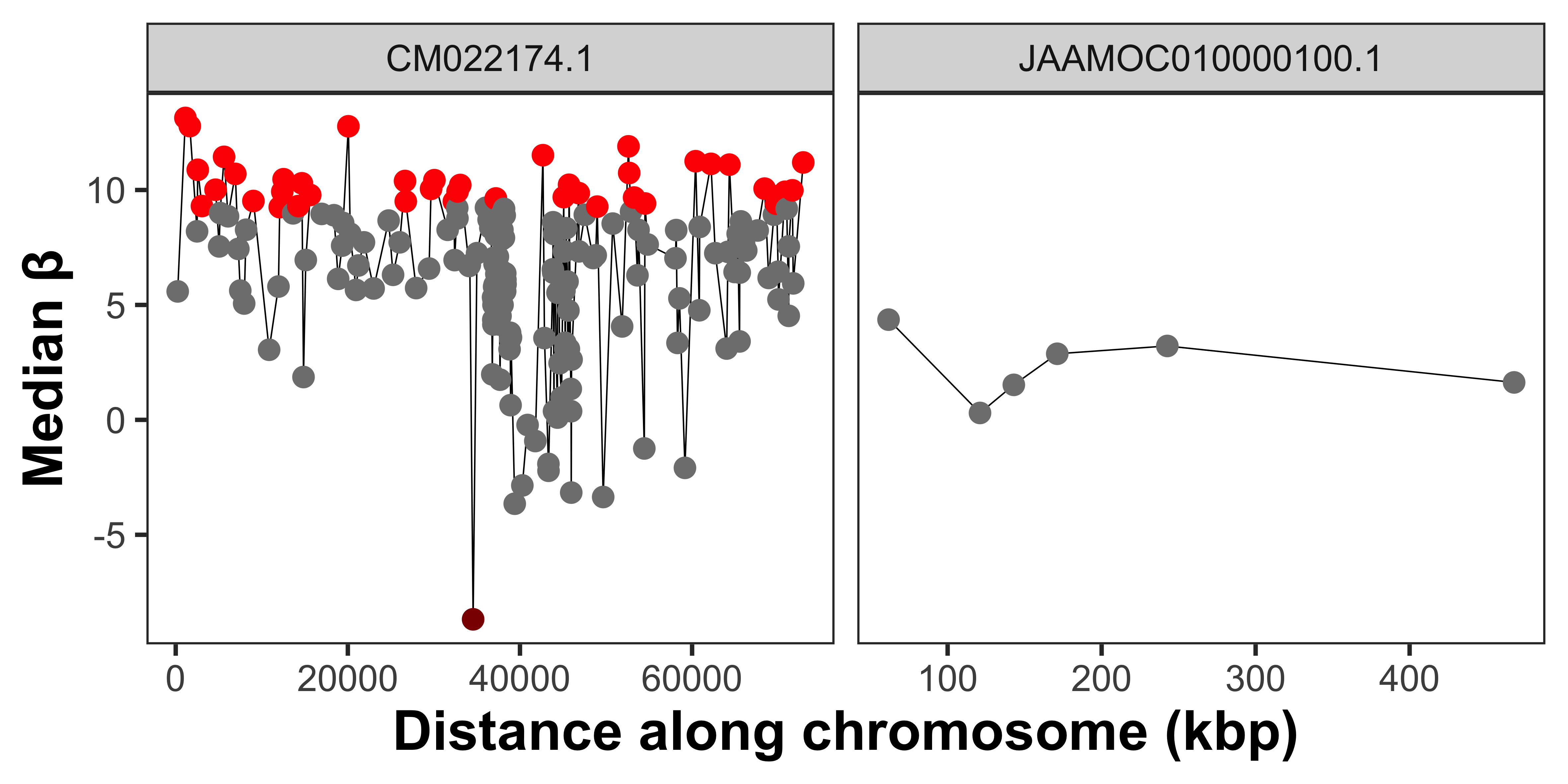


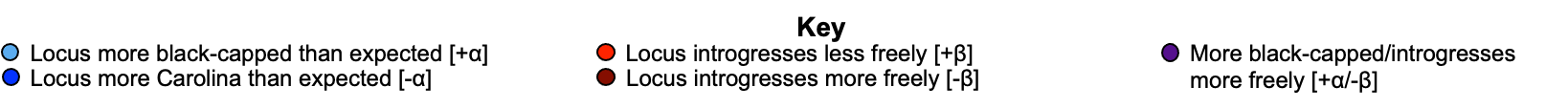
**Chromosome UNK:** JAAMOC010000119.1, JAAMOC010000135.1, JAAMOC010000148.1, JAAMOC010000213.1, JAAMOC010000238.1, JAAMOC010000250.1, JAAMOC010000267.1, JAAMOC010000305.1, JAAMOC010000332.1, JAAMOC010000356.1, JAAMOC010000366.1, JAAMOC010000372.1, JAAMOC010000412.1, JAAMOC010000432.1, JAAMOC010000459.1, JAAMOC010000470.1, JAAMOC010000493.1, JAAMOC010000503.1, JAAMOC010000523.1, JAAMOC010000528.1, JAAMOC010000700.1, JAAMOC010000746.1, JAAMOC010000779.1, JAAMOC010000841.1, JAAMOC010000854.1, JAAMOC010001547.1 not displayed as only one RAD marker present


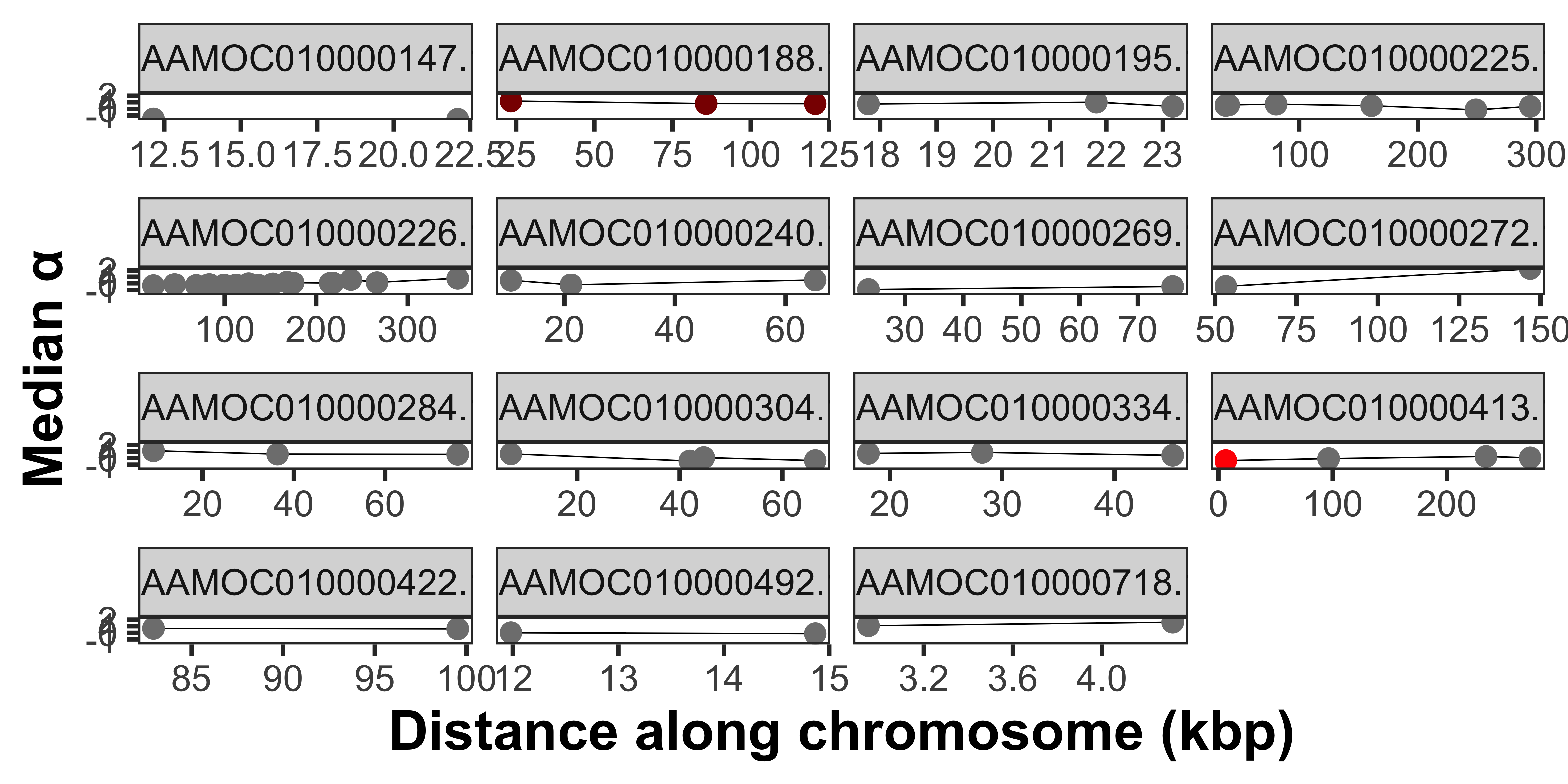

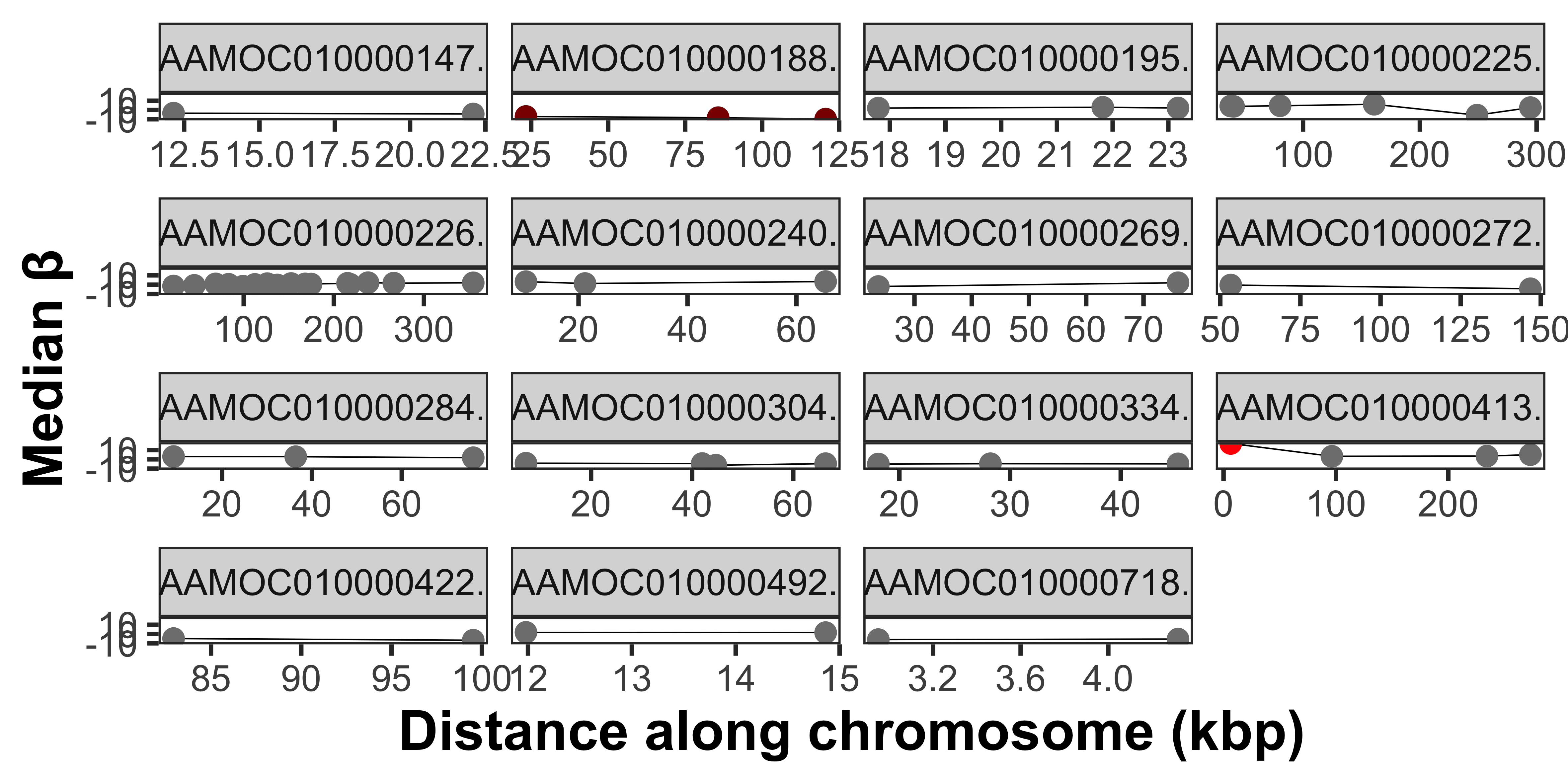


**Chromosome LG2** (only 1 non-significant locus found on this linkage group - JAAMOC010000584.1 - so not displayed)

**Chromosome LGE22:** JAAMOC010000588.1 not displayed as only one RAD marker present

**
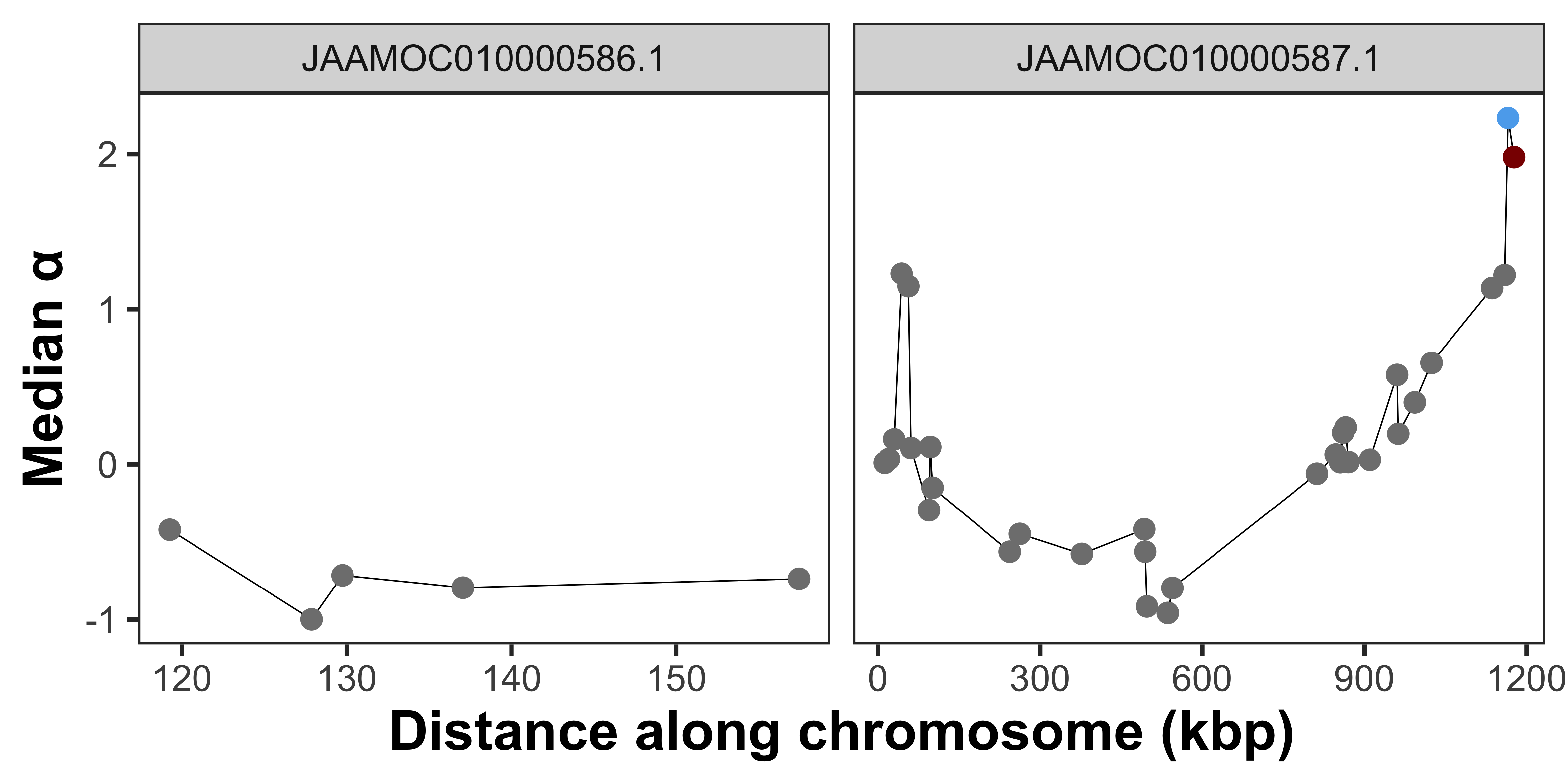

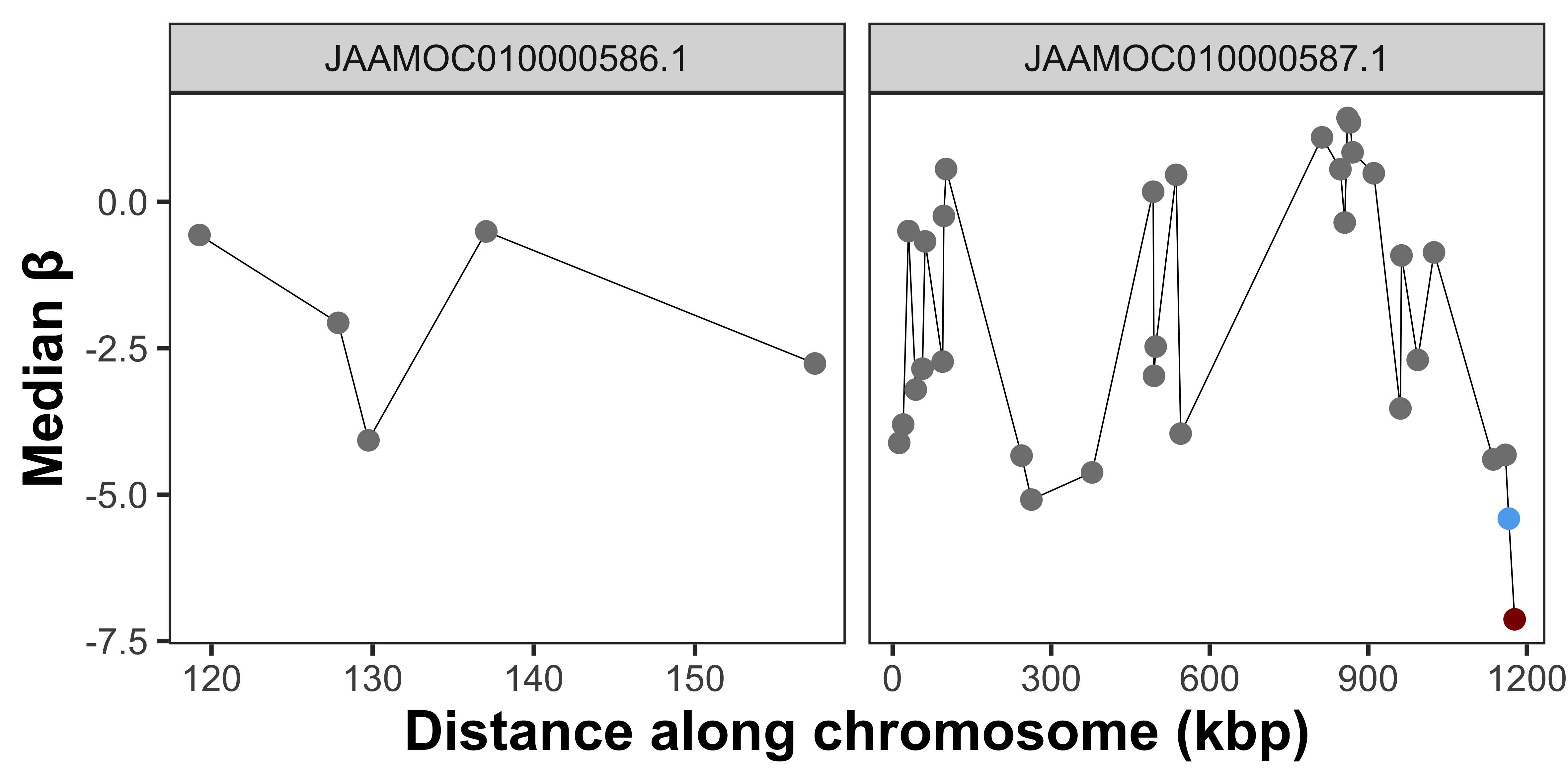
**

# Fig. S6

Before carrying out comparisons, we validated that the two time periods used by our study (1978 to 2014, y-axis) and the Pennsylvania study (2000 to 2010, x-axis) were correlated for (a) temperature and (b) precipitation, using 10,000 random points across the lower 48 states of the United States. Although temperature was correlated, precipitation was only weakly correlated across the time periods, so we restricted our analyses to temperature. Code for generating these plots is given at <https://github.com/laninsky/chickadees>.

[[back to *Contents*](#_top)]

**(a)**


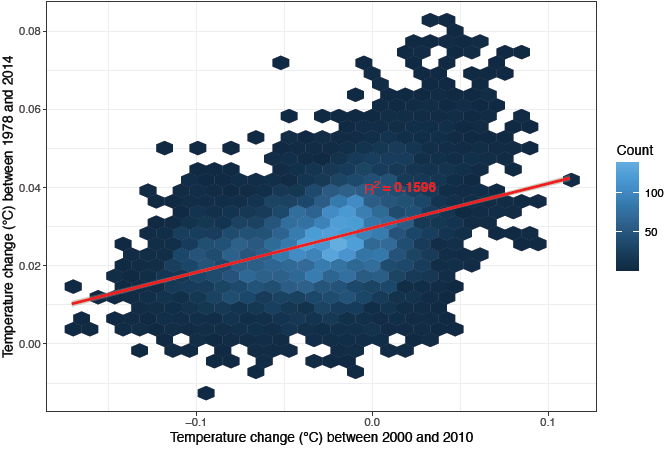


**(b)**


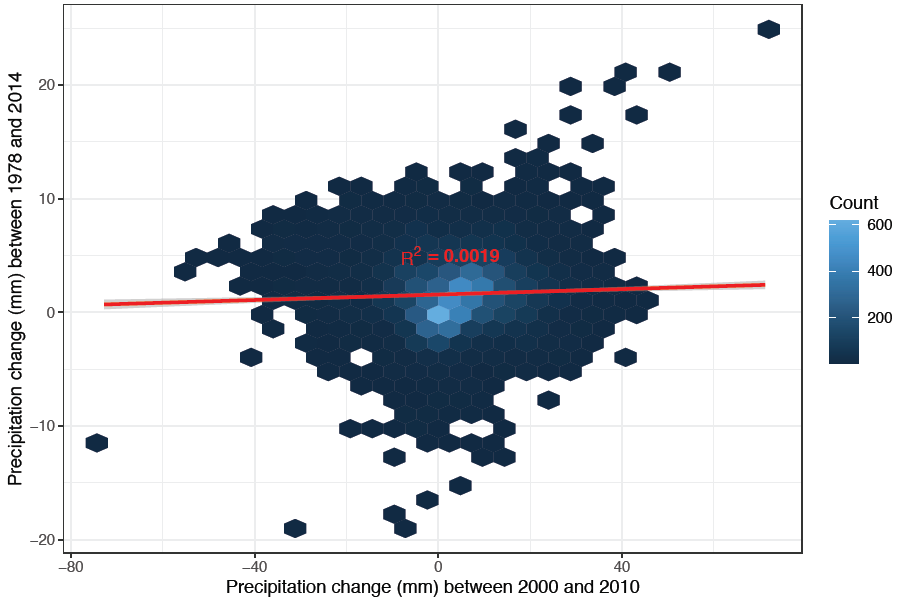


# Fig. S7

Contrasts of rate of change based on Pennsylvania study length (1998-2002 versus 2008-2012, left column for each state) and Missouri study length (1976-1980 versus 2012-2016, right column for each state) between Missouri (left) and Pennsylvania (right) for (A) temperature and (B) precipitation. [[back to *Contents*](#_top)]


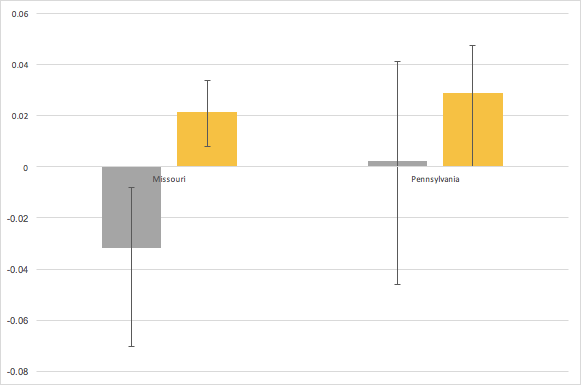
(A)


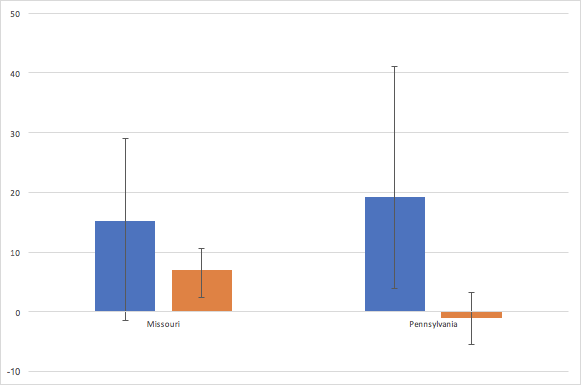
(B)

# Fig. S8

Histogram of temperature change between the start (average temperature across 1976-1980) and the end (2012-2016) of our study showing larger average increase in temperature in Pennsylvania versus Missouri, using PRISM sites located within 50 km of the Missouri (grey) and Pennsylvania transect (orange). Mean values for each location are shown by the dashed lines. Code for generating this plot is given at https://github.com/laninsky/chickadees [[back to *Contents*](#_top)]


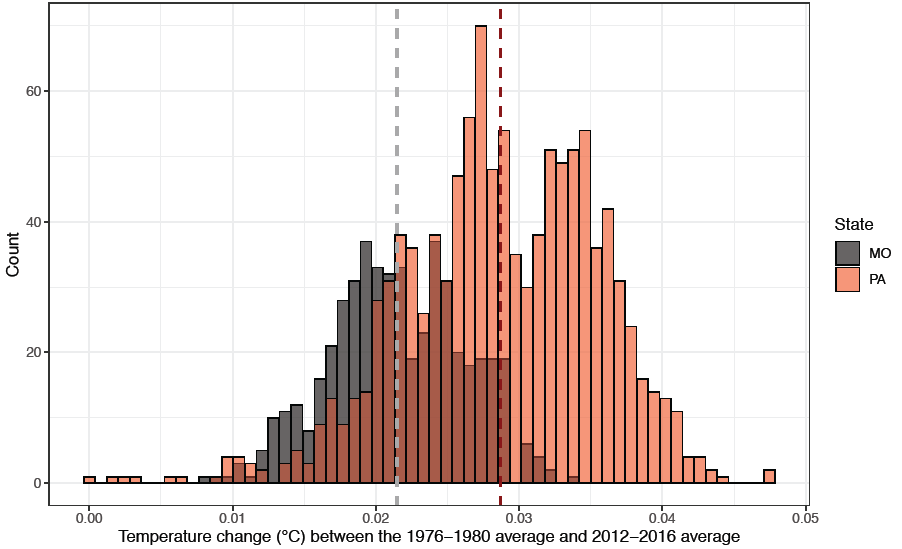


# Table S1

(next page)

Summarized metadata for this study. Individual samples given in rows and the following variables in columns: **Catalog_number**: 2016 and reference (samples from outside the hybrid zone) voucher specimens archived with the University of Kansas Biodiversity Institute (KUBI) or 1978-1980 samples with voucher specimens deposited at Louisiana State University of Natural Science, Baton Rouge, Louisiana. **Tissue_number:** genetic sample ID archived with KUBI (Sampling period: MODERN/REFERENCE_BC/REFERENCE_CC) or the Smithsonian Institution Division of Birds (Sampling period: SMITHSONIAN). **BC_genetic_cluster_assignment**: proportion of the bird’s genome assigned to the black-capped chickadee STRUCTURE cluster. **CC_genetic_cluster_assignment**: proportion of the bird’s genome assigned to the Carolina chickadee STRUCTURE cluster. **DecimalLongitude**: longitude where the sample was taken, **DecimalLatitude** the latitude. **Sampling_period**: whether the sample was obtained in 2016 e.g., ‘MODERN’, 1978-1980 e.g., ‘SMITHSONIAN’, or was included as a reference e.g., ‘REFERENCE_BC’ and ‘REFERENCE_CC’. **Location_code:** sites displayed in the figures/analyses of this manuscript. Note, due to different sampling regimes between the temporal samples, site numbers are not equivalent between 1978-1980 and 2016. **Included_in_tess3R:** whether an individual was included in the spatial interpolation/geographic cline analyses (based on **Location_Code**). Sample **Sex** is male, female, or unknown. **Specific_locality:** text description of sampling locality. **County** records the county, and **State** the U.S. State the individual was sampled in. **Remarks:** observations made during the sampling event (e.g., behavior, co-sampled individuals). **Day**, **Month**, and **Year**: when sample was taken. **PC1** gives the scores per bird from the PCA analysis. **Hybrid_Index** gives the Hybrid Index per bird (relative to pure Carolina chickadee = 0, pure black-capped = 1). **Het** gives heterozygosity estimates based on a subset of SNPs fixed between samples with more than 99% STRUCTURE assignment to BC and CC genetic clusters. Please zoom in to read details of table, or alternately, a *tab delimited version of this table is available for download at: <https://github.com/laninsky/chickadees/blob/master/data/Table_S1.txt> [[back to *Contents*](#_top)]

| **Catalog_number** | **Tissue_number** | **BC_genetic_cluster_assignment** | **CC_genetic_cluster_assignment** | **DecimalLongitude** | **DecimalLatitude** | **Sampling_period** | **Location_code** | **Included_in_tess3r** | **Sex** | **Specific_locality** | **County** | **State** | **Remarks** | **Day** | **Month** | **Year** | **PC1** | **Hybrid_Index** | **Het** |
| --- | --- | --- | --- | --- | --- | --- | --- | --- | --- | --- | --- | --- | --- | --- | --- | --- | --- | --- | --- |
| 132041 | 32562 | 0.001 | 0.999 | -94.1084 | 38.0112 | MODERN | 47 | YES | male | Schell-Osage Conservation Area, southwest section | Vernon | Missouri | with MBR # 9033 (KU 132042); | 5 | Apr | 2016 | 8.726140897 | 0.6502 | 0.040816327 |
| 132042 | 32561 | 0.001 | 0.999 | -94.1084 | 38.0112 | MODERN | 47 | YES | female | Schell-Osage Conservation Area, southwest section | Vernon | Missouri | with MBR # 9034 (KU 132041) | 5 | Apr | 2016 | 7.537696186 | 0.6836 | 0 |
| 132043 | 32565 | 0.001 | 0.999 | -94.1084 | 38.0112 | MODERN | 47 | YES | male | Schell-Osage Conservation Area, southwest section | Vernon | Missouri | with female, but she was not collected | 5 | Apr | 2016 | 8.007395406 | 0.6609 | 0.02173913 |
| 132044 | 32606 | 0.001 | 0.999 | -94.1034 | 38.0116 | MODERN | 48 | YES | female | Schell-Osage Conservation Area, southwest section | Vernon | Missouri | with KU 132045 | 5 | Apr | 2016 | 7.746775958 | 0.6589 | 0.022222222 |
| 132045 | 32605 | 0.001 | 0.999 | -94.1034 | 38.0116 | MODERN | 48 | YES | male | Schell-Osage Conservation Area, southwest section | Vernon | Missouri | with KU 132044 | 5 | Apr | 2016 | 7.454238843 | 0.6648 | 0.039215686 |
| 132046 | 32564 | 0.001 | 0.999 | -94.1107 | 38.0125 | MODERN | 44 | YES | female | Schell-Osage Conservation Area, southwest section | Vernon | Missouri | with KU 132047 | 5 | Apr | 2016 | 5.312526571 | 0.6799 | 0 |
| 132047 | 32563 | 0.001 | 0.999 | -94.1107 | 38.0125 | MODERN | 44 | YES | male | Schell-Osage Conservation Area, southwest section | Vernon | Missouri | with KU 132046 | 5 | Apr | 2016 | 6.555044225 | 0.6432 | 0.063829787 |
| 132048 | 32619 | 0.001 | 0.999 | -93.713 | 37.875 | MODERN | 50 | NO | male | Bird Song Conservation Area | St. Clair | Missouri | with KU 132049 | 4 | Apr | 2016 | 6.936238132 | 0.7057 | 0.027777778 |
| 132049 | 32602 | 0.001 | 0.999 | -93.713 | 37.875 | MODERN | 50 | NO | female | Bird Song Conservation Area | St. Clair | Missouri | with KU 132048 | 4 | Apr | 2016 | 6.033301073 | 0.6715 | 0.044444444 |
| 132050 | 32620 | 0.001 | 0.999 | -93.713 | 37.875 | MODERN | 50 | NO | female | Bird Song Conservation Area | St. Clair | Missouri | this bird is in the background giving calls as the male sing's audio ref cut, MBR-MO-2016-025 (original cut 49), male was not collected | 4 | Apr | 2016 | 8.861601013 | 0.6527 | 0 |
| 132051 | 32626 | 0 | 1 | -93.713 | 37.875 | MODERN | 50 | NO | male | Bird Song Conservation Area | St. Clair | Missouri | with presumed female who was carrying nesting material | 4 | Apr | 2016 | 6.7178534 | 0.6655 | 0.023255814 |
| 132052 | 32625 | 0.001 | 0.999 | -93.713 | 37.875 | MODERN | 50 | NO | male | Bird Song Conservation Area | St. Clair | Missouri | NA | 4 | Apr | 2016 | 7.547966373 | 0.6596 | 0 |
| 132053 | 32624 | 0.001 | 0.999 | -93.713 | 37.875 | MODERN | 50 | NO | male | Bird Song Conservation Area | St. Clair | Missouri | NA | 4 | Apr | 2016 | 6.505887311 | 0.6483 | 0.054545455 |
| 132054 | 32618 | 0.001 | 0.999 | -93.713 | 37.875 | MODERN | 50 | NO | male | Bird Song Conservation Area | St. Clair | Missouri | in adjacent territory to KU 132048 | 4 | Apr | 2016 | 6.607619855 | 0.6995 | 0.048780488 |
| 132055 | 32621 | 0.002 | 0.998 | -93.713 | 37.875 | MODERN | 50 | NO | female | Bird Song Conservation Area | St. Clair | Missouri | with KU # 132056 | 4 | Apr | 2016 | 7.64718778 | 0.6469 | 0.041666667 |
| 132056 | 32622 | 0.001 | 0.999 | -93.713 | 37.875 | MODERN | 50 | NO | male | Bird Song Conservation Area | St. Clair | Missouri | with KU # 132055 | 4 | Apr | 2016 | 7.182920913 | 0.6593 | 0 |
| 132057 | 32623 | 0.001 | 0.999 | -93.713 | 37.875 | MODERN | 50 | NO | male | Bird Song Conservation Area | St. Clair | Missouri | NA | 4 | Apr | 2016 | 5.566332018 | 0.7119 | 0.021276596 |
| 132058 | 32617 | 0.9998 | 2.00E-04 | -94.4273 | 38.2794 | MODERN | 4 | NO | male | north side of Butler Lake, Miami Creek | Bates | Missouri | with KU 132059 | 13 | Apr | 2016 | -11.77526863 | 0.2843 | 0 |
| 132059 | 32616 | 0.999 | 0.001 | -94.4273 | 38.2794 | MODERN | 4 | NO | female | north side of Butler Lake, Miami Creek | Bates | Missouri | with KU 132058 | 13 | Apr | 2016 | -8.563600085 | 0.2986 | 0.022727273 |
| 132060 | 32615 | 0.999 | 0.001 | -94.4342 | 38.2843 | MODERN | 1 | NO | male | north side of Butler Lake, Miami Creek | Bates | Missouri | NA | 13 | Apr | 2016 | -10.0185125 | 0.2894 | 0 |
| 132061 | 32612 | 0.999 | 0.001 | -94.4279 | 38.2809 | MODERN | 3 | NO | male | north side of Butler Lake, Miami Creek | Bates | Missouri | with KU 132062 | 13 | Apr | 2016 | -6.798119927 | 0.2892 | 0 |
| 132062 | 32611 | 0.999 | 0.001 | -94.4279 | 38.2809 | MODERN | 3 | NO | female | north side of Butler Lake, Miami Creek | Bates | Missouri | with KU 132061 | 13 | Apr | 2016 | -10.63398592 | 0.292 | 0 |
| 132063 | 32614 | 0.9992 | 8.00E-04 | -94.4336 | 38.2822 | MODERN | 2 | NO | male | north side of Butler Lake, Miami Creek | Bates | Missouri | with KU 132064 | 13 | Apr | 2016 | -9.8525509 | 0.2863 | 0 |
| 132064 | 32613 | 1 | 0 | -94.4336 | 38.2822 | MODERN | 2 | NO | female | north side of Butler Lake, Miami Creek | Bates | Missouri | with KU 132063 | 13 | Apr | 2016 | -10.83535717 | 0.2961 | 0 |
| 132065 | 32601 | 0.001 | 0.999 | -94.0333 | 38.001 | MODERN | 49 | YES | male | Schell-Osage Conservation Area, east side | St. Clair | Missouri | NA | 4 | Apr | 2016 | 8.457057306 | 0.7022 | 0.073170732 |
| 132066 | 32599 | 0.002 | 0.998 | -94.099 | 38.0304 | MODERN | 46 | YES | male | Schell-Osage Conservation Area, northeast section | St. Clair | Missouri | with KU 132067 | 4 | Apr | 2016 | 7.332764634 | 0.6658 | 0.038461538 |
| 132067 | 32600 | 0 | 1 | -94.099 | 38.0304 | MODERN | 46 | YES | female | Schell-Osage Conservation Area, northeast section | St. Clair | Missouri | with KU 132066 | 4 | Apr | 2016 | 9.697328319 | 0.6947 | 0.035714286 |
| 132068 | 32609 | 0.001 | 0.999 | -94.1045 | 38.0452 | MODERN | 38 | YES | female | Schell-Osage Conservation Area, northwest section | Vernon | Missouri | sang soft (quiet) Carolina song; carrying nesting material; in almost the exact same site as KU 132069; male was not collected | 5 | Apr | 2016 | 8.532706882 | 0.6455 | 0.076923077 |
| 132069 | 32610 | 2.00E-04 | 0.9998 | -94.1045 | 38.0452 | MODERN | 38 | YES | female | Schell-Osage Conservation Area, northwest section | Vernon | Missouri | almost in the exact same site as KU 132068, who was in breeding condition; male was not collected | 5 | Apr | 2016 | 9.649195198 | 0.6886 | 0.065217391 |
| 132070 | 32598 | 0.002 | 0.998 | -94.1033 | 38.0477 | MODERN | 39 | YES | female | Schell-Osage Conservation Area, northwest section | Vernon | Missouri | with male, who was not collected, but was audio recorded (audio ref #: MBR-MO-2016-044, original cuts 85 & 87) | 5 | Apr | 2016 | 8.596575639 | 0.6756 | 0 |
| 132071 | 32604 | 0.001 | 0.999 | -94.1033 | 38.0441 | MODERN | 42 | YES | male | Schell-Osage Conservation Area, northwest section | Vernon | Missouri | NA | 5 | Apr | 2016 | 7.63855926 | 0.697 | 0 |
| 132072 | 32603 | 0 | 1 | -94.1011 | 38.047 | MODERN | 40 | YES | male | Schell-Osage Conservation Area, northwest section | Vernon | Missouri | NA | 5 | Apr | 2016 | 9.034296767 | 0.7036 | 0.036363636 |
| 132073 | 32593 | 0.9942 | 0.0058 | -94.2187 | 38.1737 | MODERN | 7 | NO | male | Pleasant Gap, west of | Bates | Missouri | NA | 28 | Apr | 2016 | -9.988241583 | 0.2818 | 0.045454545 |
| 132074 | 32591 | 0.9982 | 0.0018 | -94.1942 | 38.1713 | MODERN | 9 | NO | male | Pleasant Gap, west of | Bates | Missouri | with KU 132075 | 28 | Apr | 2016 | -6.78415185 | 0.3055 | 0 |
| 132075 | 32592 | 0.025 | 0.975 | -94.1942 | 38.1713 | MODERN | 9 | NO | female | Pleasant Gap, west of | Bates | Missouri | with KU 132074 | 28 | Apr | 2016 | 6.1503072 | 0.6248 | 0.085106383 |
| 132076 | 32608 | 0.5158 | 0.4842 | -94.2007 | 38.1717 | MODERN | 8 | NO | male | Pleasant Gap, west of | Bates | Missouri | with KU 132077 | 28 | Apr | 2016 | -1.999815542 | 0.4503 | 0.592592593 |
| 132077 | 32607 | 0.999 | 0.001 | -94.2007 | 38.1717 | MODERN | 8 | NO | female | Pleasant Gap, west of | Bates | Missouri | with KU 132076 | 28 | Apr | 2016 | -9.103707917 | 0.2545 | 0.023809524 |
| 132078 | 32595 | 0.999 | 0.001 | -94.2072 | 38.1764 | MODERN | 5 | NO | male | Pleasant Gap, west of | Bates | Missouri | with KU 132079 | 28 | Apr | 2016 | -8.968278011 | 0.2711 | 0 |
| 132079 | 32596 | 0.999 | 0.001 | -94.2072 | 38.1764 | MODERN | 5 | NO | female | Pleasant Gap, west of | Bates | Missouri | with KU 132078 | 28 | Apr | 2016 | -9.096739272 | 0.3184 | 0 |
| 132080 | 32594 | 0.939 | 0.061 | -94.2011 | 38.1827 | MODERN | 6 | NO | male | Pleasant Gap, west of | Bates | Missouri | NA | 28 | Apr | 2016 | -7.262995866 | 0.3277 | 0.037037037 |
| 132081 | 32576 | 0.001 | 0.999 | -94.154 | 38.0771 | MODERN | 16 | NO | male | Rockville, 4.1 miles west (east of Prairie City) | Bates | Missouri | with KU 132086 in riparian woodland strip | 8 | Apr | 2016 | 7.643447113 | 0.6784 | 0.018181818 |
| 132082 | 32577 | 0.0092 | 0.9908 | -94.154 | 38.0771 | MODERN | 16 | NO | male | Rockville, 4.1 miles west (east of Prairie City) | Bates | Missouri | NA | 8 | Apr | 2016 | 6.485050444 | 0.6506 | 0.017857143 |
| 132083 | 32572 | 0.6372 | 0.3628 | -94.154 | 38.0771 | MODERN | 16 | NO | female | Rockville, 4.1 miles west (east of Prairie City) | Bates | Missouri | with KU 132084 | 8 | Apr | 2016 | -2.278160659 | 0.4583 | 0.2 |
| 132084 | 32573 | 0.9334 | 0.0666 | -94.154 | 38.0771 | MODERN | 16 | NO | male | Rockville, 4.1 miles west (east of Prairie City) | Bates | Missouri | with KU 132083 | 8 | Apr | 2016 | -9.336754906 | 0.2843 | 0.066666667 |
| 132085 | 32566 | 0.002 | 0.998 | -94.154 | 38.0771 | MODERN | 16 | NO | male | Rockville, 4.1 miles west (east of Prairie City) | Bates | Missouri | NA | 8 | Apr | 2016 | 4.119285201 | 0.6707 | 0.020833333 |
| 132086 | 32575 | 0.0024 | 0.9976 | -94.154 | 38.0771 | MODERN | 16 | NO | female | Rockville, 4.1 miles west (east of Prairie City) | Bates | Missouri | with KU 132081 | 8 | Apr | 2016 | 8.133750902 | 0.6535 | 0.036363636 |
| 132087 | 32556 | 0.525 | 0.475 | -94.0863 | 38.1168 | MODERN | 34 | YES | male | Rockville, 3.2 miles north | Bates | Missouri | NA | 9 | Apr | 2016 | 0.246391893 | 0.4626 | 0.693877551 |
| 132088 | 32559 | 0.001 | 0.999 | -94.0858 | 38.1178 | MODERN | 35 | YES | male | Rockville, 3.2 miles north | Bates | Missouri | with KU 132089 | 9 | Apr | 2016 | 9.316788451 | 0.6781 | 0.018518519 |
| 132089 | 32560 | 0 | 1 | -94.0858 | 38.1178 | MODERN | 35 | YES | female | Rockville, 3.2 miles north | Bates | Missouri | with KU 132088 | 9 | Apr | 2016 | 8.25536505 | 0.692 | 0.022727273 |
| 132090 | 32586 | 0.999 | 0.001 | -94.0847 | 38.1161 | MODERN | 37 | YES | female | Rockville, 3.2 miles north | Bates | Missouri | with KU 132091 | 9 | Apr | 2016 | -9.310092384 | 0.3188 | 0 |
| 132091 | 32585 | 0.001 | 0.999 | -94.0847 | 38.1161 | MODERN | 37 | YES | male | Rockville, 3.2 miles north | Bates | Missouri | with KU 132090 | 9 | Apr | 2016 | 6.503960744 | 0.6416 | 0.047619048 |
| 132092 | 32553 | 0.002 | 0.998 | -94.0878 | 38.1191 | MODERN | 22 | YES | male | Rockville, 3.2 miles north | Bates | Missouri | NA | 9 | Apr | 2016 | 5.947438054 | 0.6685 | 0.020833333 |
| 132093 | 32568 | 0.999 | 0.001 | -94.0872 | 38.1193 | MODERN | 23 | YES | male | Rockville, 3.2 miles north | Bates | Missouri | with KU 132094 | 9 | Apr | 2016 | -9.727053262 | 0.2449 | 0 |
| 132094 | 32555 | 0.9352 | 0.0648 | -94.0872 | 38.1193 | MODERN | 23 | YES | female | Rockville, 3.2 miles north | Bates | Missouri | with KU 132093 | 9 | Apr | 2016 | -7.900280727 | 0.3401 | 0.033333333 |
| 132095 | 32571 | 0.8948 | 0.1052 | -94.1182 | 38.1198 | MODERN | 10 | YES | male | Rockville, 4 miles northwest | Bates | Missouri | NA | 9 | Apr | 2016 | -8.52919845 | 0.3088 | 0.039215686 |
| 132096 | 32574 | 0.6464 | 0.3536 | -94.1163 | 38.1195 | MODERN | 13 | YES | male | Rockville, 4 miles northwest | Bates | Missouri | NA | 9 | Apr | 2016 | -3.268479827 | 0.4102 | 0.508474576 |
| 132097 | 32589 | 0 | 1 | -94.1163 | 38.1195 | MODERN | 13 | YES | male | Rockville, 4 miles northwest | Bates | Missouri | with KU 132098 | 9 | Apr | 2016 | 8.473106249 | 0.669 | 0.01754386 |
| 132098 | 32590 | 0.582 | 0.418 | -94.1163 | 38.1195 | MODERN | 13 | YES | female | Rockville, 4 miles northwest | Bates | Missouri | with KU 132097 | 9 | Apr | 2016 | -2.770004898 | 0.4238 | 0.25 |
| 132099 | 32584 | 0.0386 | 0.9614 | -94.1198 | 38.0525 | MODERN | 31 | YES | male | Rockville, 2.3 miles west | Bates | Missouri | with female, who was carrying nesting material; she gave muted Carolina song after male was collected; she was not collected | 8 | Apr | 2016 | 8.221493837 | 0.6146 | 0.049180328 |
| 132100 | 32587 | 0.5086 | 0.4914 | -94.1213 | 38.0639 | MODERN | 19 | YES | male | Rockville, 2.3 miles west | Bates | Missouri | with KU 132101 | 8 | Apr | 2016 | -0.672227082 | 0.4482 | 0.508196721 |
| 132101 | 32588 | 0.003 | 0.997 | -94.1213 | 38.0639 | MODERN | 19 | YES | female | Rockville, 2.3 miles west | Bates | Missouri | with KU 132100 | 8 | Apr | 2016 | 4.889396289 | 0.6811 | 0 |
| 132102 | 32567 | 0.4576 | 0.5424 | -94.1211 | 38.0641 | MODERN | 20 | YES | male | Rockville, 2.3 miles west | Bates | Missouri | initially audio recorder on 5 April, recorded again and collected on 8 April | 8 | Apr | 2016 | -2.098054088 | 0.4754 | 0.542372881 |
| 132103 | 32578 | 0.4882 | 0.5118 | -94.1848 | 38.0774 | MODERN | 14 | NO | male | Rockville, 5.8 miles west (west of Prairie City) | Bates | Missouri | with KU 132104 | 8 | Apr | 2016 | 0.181976978 | 0.5031 | 0.645833333 |
| 132104 | 32579 | 0.006 | 0.994 | -94.1848 | 38.0774 | MODERN | 14 | NO | female | Rockville, 5.8 miles west (west of Prairie City) | Bates | Missouri | with KU 132104 | 8 | Apr | 2016 | 7.504982293 | 0.6666 | 0.023809524 |
| 132105 | 32542 | 0.6352 | 0.3648 | -94.1857 | 38.0781 | MODERN | 11 | NO | female | Rockville, 5.8 miles west (west of Prairie City) | Bates | Missouri | with KU 132106 | 12 | Apr | 2016 | -3.898743781 | 0.4123 | 0.204545455 |
| 132106 | 32541 | 0.0178 | 0.9822 | -94.1857 | 38.0781 | MODERN | 11 | NO | male | Rockville, 5.8 miles west (west of Prairie City) | Bates | Missouri | with KU 132105 | 12 | Apr | 2016 | 5.605900337 | 0.6566 | 0.045454545 |
| 132107 | 32580 | 0.4978 | 0.5022 | -94.0548 | 38.0655 | MODERN | 41 | YES | male | Rockville, 1.5 miles southeast | St. Clair | Missouri | with female (not collected); male responded to Carolina song playback by giving Carolina song (not recorded, too windy) | 8 | Apr | 2016 | -2.200099594 | 0.4805 | 0.596153846 |
| 132108 | 32570 | 0.001 | 0.999 | -94.0528 | 38.0674 | MODERN | 43 | YES | female | Rockville, 1.5 miles southeast | St. Clair | Missouri | with KU 132109; male gave Carolina song in response to Carolina song playback | 8 | Apr | 2016 | 5.887277644 | 0.6314 | 0.036363636 |
| 132109 | 32569 | 0.001 | 0.999 | -94.0528 | 38.0674 | MODERN | 43 | YES | male | Rockville, 1.5 miles southeast | St. Clair | Missouri | with KU 132108; male sang Carolina song in response to Carolina playback (not recorded, too windy) | 8 | Apr | 2016 | 8.022602801 | 0.6537 | 0.015384615 |
| 132110 | 32558 | 0.003 | 0.997 | -94.0537 | 38.0636 | MODERN | 45 | YES | male | Rockville, 1.5 miles southeast | St. Clair | Missouri | with KU 132111 | 8 | Apr | 2016 | 4.185659502 | 0.6612 | 0 |
| 132111 | 32557 | 0.0186 | 0.9814 | -94.0537 | 38.0636 | MODERN | 45 | YES | female | Rockville, 1.5 miles southeast | St. Clair | Missouri | with KU 132110 | 8 | Apr | 2016 | 3.733820519 | 0.6503 | 0 |
| 132112 | 32581 | 0.001 | 0.999 | -94.0118 | 38.1753 | MODERN | 30 | YES | male | Appleton City, 1.7 miles southeast | St. Clair | Missouri | with female, neither sang; in reclaimed min with scrubby woods that had dense understory of Japanese honeysuckle | 9 | Apr | 2016 | 5.995891958 | 0.6785 | 0.03030303 |
| 132113 | 32536 | 0.0124 | 0.9876 | -94.0046 | 38.1759 | MODERN | 24 | YES | male | Appleton City, 1.7 miles southeast | St. Clair | Missouri | Heard singing both Carolina & Black-capped songs; counter singing with 2 other males ((KU 132114-5) in reclaimed min with scrubby woods that had dense understory of Japanese honeysuckle | 12 | Apr | 2016 | 6.515826654 | 0.6847 | 0.066666667 |
| 132114 | 32534 | 0.4874 | 0.5126 | -94.0046 | 38.1749 | MODERN | 33 | YES | male | Appleton City, 1.7 miles southeast | St. Clair | Missouri | counter singing with 2 other males ((KU 132113 & 132115) in reclaimed min with scrubby woods that had dense understory of Japanese honeysuckle | 12 | Apr | 2016 | -0.364269445 | 0.4846 | 0.491803279 |
| 132115 | 32543 | 0.4226 | 0.5774 | -94.0046 | 38.1749 | MODERN | 33 | YES | male | Appleton City, 1.7 miles southeast | St. Clair | Missouri | counter singing with 2 other males (KU 132113-4) in reclaimed min with scrubby woods that had dense understory of Japanese honeysuckle | 12 | Apr | 2016 | 3.150818989 | 0.5259 | 0.571428571 |
| 132116 | 32537 | 0.5028 | 0.4972 | -93.992 | 38.1745 | MODERN | 36 | YES | male | Appleton City, 2.2 miles southeast | St. Clair | Missouri | with mate in reclaimed mine | 12 | Apr | 2016 | -1.964693087 | 0.4606 | 0.58 |
| 132117 | 32583 | 0.0024 | 0.9976 | -94.0251 | 38.1735 | MODERN | 32 | YES | female | Appleton City Cemetery | St. Clair | Missouri | with KU 132118 | 9 | Apr | 2016 | 4.611155132 | 0.6433 | 0 |
| 132118 | 32582 | 0.5016 | 0.4984 | -94.0251 | 38.1735 | MODERN | 32 | YES | male | Appleton City Cemetery | St. Clair | Missouri | with KU 132117 in scrubby woodland (reclaimed mine); sang only Black-capped song, but not recorded | 9 | Apr | 2016 | -1.231740409 | 0.4597 | 0.652173913 |
| 132119 | 32554 | 0.1116 | 0.8884 | -94.0221 | 38.1748 | MODERN | 29 | YES | male | Appleton City Cemetery | St. Clair | Missouri | heard singing Black-capped song, but not recorded; in reclaimed mine scrubby woodland | 9 | Apr | 2016 | 5.433298492 | 0.6214 | 0.055555556 |
| 132120 | 32545 | 0.001 | 0.999 | -94.0207 | 38.1757 | MODERN | 21 | YES | female | Appleton City Cemetery, adjacent to east side | St. Clair | Missouri | with KU 132121 in reclaimed mine scrubby woodland | 12 | Apr | 2016 | 7.503773024 | 0.6641 | 0 |
| 132121 | 32546 | 0.1604 | 0.8396 | -94.0207 | 38.1757 | MODERN | 21 | YES | male | Appleton City Cemetery, adjacent to east side | St. Clair | Missouri | with KU 132120 in reclaimed mine scrubby woodland | 12 | Apr | 2016 | -1.641437917 | 0.6169 | 0.042553191 |
| 132122 | 32538 | 0.5858 | 0.4142 | -94.3459 | 37.9701 | MODERN | 25 | NO | male | Four Rivers Conservation Area, Unit 1, Prairie tract | Vernon | Missouri | recorded over different days; sang both Black-capped & Carolina songs, as did other individuals at this site | 13 | Apr | 2016 | -1.216406241 | 0.4706 | 0.659574468 |
| 132123 | 32551 | 0.9864 | 0.0136 | -94.2394 | 38.0344 | MODERN | 15 | NO | male | Four Rivers Conservation Area, east side of Unit 2, pool 12 | Vernon | Missouri | with KU 132124 | 12 | Apr | 2016 | -6.589422295 | 0.2982 | 0.026315789 |
| 132124 | 32552 | 0.284 | 0.716 | -94.2394 | 38.0344 | MODERN | 15 | NO | female | Four Rivers Conservation Area, east side of Unit 2, pool 12 | Vernon | Missouri | with KU 132123 | 12 | Apr | 2016 | 3.392237946 | 0.5734 | 0.203703704 |
| 132125 | 32548 | 0.502 | 0.498 | -94.2208 | 38.0161 | MODERN | 27 | NO | male | Four Rivers Conservation Area, east side of Unit 4 | Vernon | Missouri | with KU 132126 | 13 | Apr | 2016 | 0.361703508 | 0.5011 | 0.625 |
| 132126 | 32547 | 0.4078 | 0.5922 | -94.2208 | 38.0161 | MODERN | 27 | NO | female | Four Rivers Conservation Area, east side of Unit 4 | Vernon | Missouri | with KU 132125 | 13 | Apr | 2016 | 0.198787343 | 0.5353 | 0.156862745 |
| 132127 | 32549 | 0.4824 | 0.5176 | -94.2206 | 38.0181 | MODERN | 28 | NO | male | Four Rivers Conservation Area, east side of Unit 4 | Vernon | Missouri | with KU 132128 | 13 | Apr | 2016 | 0.056396222 | 0.5117 | 0.704545455 |
| 132128 | 32550 | 0.999 | 0.001 | -94.2206 | 38.0181 | MODERN | 28 | NO | female | Four Rivers Conservation Area, east side of Unit 4 | Vernon | Missouri | with KU 132127 | 13 | Apr | 2016 | -8.074208052 | 0.2944 | 0 |
| 132129 | 32540 | 0.135 | 0.865 | -94.2216 | 38.0207 | MODERN | 18 | NO | female | Four Rivers Conservation Area, east side of Unit 4 | Vernon | Missouri | with mate, not collected | 13 | Apr | 2016 | 7.743448933 | 0.6293 | 0.215686275 |
| 132130 | 32535 | 0.7626 | 0.2374 | -94.2243 | 38.0236 | MODERN | 17 | NO | unknown | Four Rivers Conservation Area, east side of Unit 4 | Vernon | Missouri | note: if this is not a female, then the mate of this individual was audio recorded (see above) | 13 | Apr | 2016 | -6.756797009 | 0.379 | 0.19047619 |
| 132131 | 32597 | 0.8492 | 0.1508 | -94.3209 | 38 | MODERN | 26 | NO | male | Four Rivers Conservation Area, south end of Unit 1 | Vernon | Missouri | with female (not collected); male sang Black-capped in response to playback | 13 | Apr | 2016 | -6.158663483 | 0.3685 | 0 |
| 132132 | 32539 | 0.596 | 0.404 | -94.2539 | 38.0449 | MODERN | 12 | NO | male | Four Rivers Conservation Area, east side of Unit 2 | Vernon | Missouri | NA | 13 | Apr | 2016 | -1.501103214 | 0.4584 | 0.688888889 |
| 90612 | 3474 | 0.999 | 0.001 | -95.259 | 38.954 | REFERENCE_BC | NA | NA | male | Lawrence | Douglas | Kansas | NA | 2 | Feb | 2000 | -7.638919082 | 0 | 0 |
| 95776 | 6281 | 0.9346 | 0.0654 | -95.388 | 39.226 | REFERENCE_BC | NA | NA | female | KU NESA Succession Plots | Jefferson | Kansas | NA | 10 | Mar | 2003 | -8.129646376 | 0.2549 | 0.02173913 |
| 131638 | 29898 | 0.999 | 0.001 | -95.206 | 39.032 | REFERENCE_BC | NA | NA | female | 3 miles north of Lawrence | Douglas | Kansas | NA | 9 | Feb | 2016 | -10.78473099 | 0 | 0.023809524 |
| 92269 | 7420 | 0.001 | 0.999 | -93.327 | 36.601 | REFERENCE_CC | NA | NA | male | northern tip of highway JJ, Table Rock Lake | Stone | Missouri | NA | 21 | Nov | 2001 | 5.768207191 | 1 | 0.018518519 |
| 92270 | 7421 | 0.001 | 0.999 | -93.327 | 36.601 | REFERENCE_CC | NA | NA | male | northern tip of highway JJ, Table Rock Lake | Stone | Missouri | NA | 21 | Nov | 2001 | 7.820299359 | 1 | 0.046153846 |
| 99703 | 649325 | 0.9998 | 2.00E-04 | -94.497 | 38.31 | SMITHSONIAN | 1 | NO | male | Miami Cr. Drainage, 3.0 mi N & 8.0 mi W Butler | Bates | Missouri | NA | 1 | Apr | 1980 | -10.81894375 | 0.292 | 0 |
| 99704 | 649329 | 0.999 | 0.001 | -94.497 | 38.31 | SMITHSONIAN | 1 | NO | female | Miami Cr. Drainage, 3.0 mi N & 8.0 mi W Butler | Bates | Missouri | NA | 1 | Apr | 1980 | -12.9144133 | 0.289 | 0.027777778 |
| 99705 | 649330 | 0.999 | 0.001 | -94.497 | 38.31 | SMITHSONIAN | 1 | NO | male | Miami Cr. Drainage, 3.0 mi N & 8.0 mi W Butler | Bates | Missouri | NA | 1 | Apr | 1980 | -9.494649459 | 0.2891 | 0 |
| 99706 | 649331 | 0.999 | 0.001 | -94.497 | 38.31 | SMITHSONIAN | 1 | NO | female | Miami Cr. Drainage, 3.0 mi N & 8.0 mi W Butler | Bates | Missouri | NA | 1 | Apr | 1980 | -8.210630717 | 0.2841 | 0.048780488 |
| 99712 | 649326 | 0.999 | 0.001 | -94.444 | 38.295 | SMITHSONIAN | 2 | NO | female | Miami Cr. Drainage, 3.0 mi N & 5.0 mi W Butler | Bates | Missouri | NA | 1 | Apr | 1980 | -10.12104186 | 0.277 | 0 |
| 99713 | 649327 | 0.999 | 0.001 | -94.444 | 38.295 | SMITHSONIAN | 2 | NO | female | Miami Cr. Drainage, 3.0 mi N & 5.0 mi W Butler | Bates | Missouri | NA | 1 | Apr | 1980 | -9.527152654 | 0.3062 | 0 |
| 99714 | 649328 | 0.9992 | 8.00E-04 | -94.444 | 38.295 | SMITHSONIAN | 2 | NO | male | Miami Cr. Drainage, 3.0 mi N & 5.0 mi W Butler | Bates | Missouri | NA | 1 | Apr | 1980 | -10.4450217 | 0.2863 | 0 |
| 99715 | 649332 | 1 | 0 | -94.444 | 38.295 | SMITHSONIAN | 2 | NO | male | Miami Cr. Drainage, 3.0 mi N & 5.0 mi W Butler | Bates | Missouri | NA | 1 | Apr | 1980 | -10.93232776 | 0.3081 | 0.022727273 |
| 99716 | 649333 | 0.9994 | 6.00E-04 | -94.444 | 38.295 | SMITHSONIAN | 2 | NO | female | Miami Cr. Drainage, 3.0 mi N & 5.0 mi W Butler | Bates | Missouri | NA | 1 | Apr | 1980 | -12.22899511 | 0.2861 | 0 |
| 99717 | 649334 | 1 | 0 | -94.444 | 38.295 | SMITHSONIAN | 2 | NO | male | Miami Cr. Drainage, 3.0 mi N & 5.0 mi W Butler | Bates | Missouri | NA | 1 | Apr | 1980 | -9.887512605 | 0.2799 | 0.022727273 |
| 99718 | 649353 | 0.999 | 0.001 | -94.128 | 38.134 | SMITHSONIAN | 3 | YES | male | 5.0 mi N & 2.5 mi W Rockville | Bates | Missouri | NA | 4 | Apr | 1980 | -10.12710665 | 0.2996 | 0 |
| 99719 | 649359 | 0.9958 | 0.0042 | -94.128 | 38.134 | SMITHSONIAN | 3 | YES | female | 5.0 mi N & 2.5 mi W Rockville | Bates | Missouri | NA | 4 | Apr | 1980 | -8.340479913 | 0.3132 | 0 |
| 99720 | 649352 | 0.999 | 0.001 | -94.119 | 38.119 | SMITHSONIAN | 4 | YES | male | 4.0 mi N & 2.5 mi W Rockville | Bates | Missouri | NA | 4 | Apr | 1980 | -10.02222643 | 0.2927 | 0 |
| 99741 | 649354 | 0.9998 | 2.00E-04 | -94.119 | 38.119 | SMITHSONIAN | 4 | YES | male | 4.0 mi N & 2.5 mi W Rockville | Bates | Missouri | NA | 4 | Apr | 1980 | -8.692150278 | 0.2942 | 0.023809524 |
| 99742 | 649355 | 0.9414 | 0.0586 | -94.119 | 38.119 | SMITHSONIAN | 4 | YES | male | 4.0 mi N & 2.5 mi W Rockville | Bates | Missouri | NA | 4 | Apr | 1980 | -9.696042379 | 0.3163 | 0 |
| 99743 | 649356 | 0.4876 | 0.5124 | -94.119 | 38.119 | SMITHSONIAN | 4 | YES | female | 4.0 mi N & 2.5 mi W Rockville | Bates | Missouri | NA | 4 | Apr | 1980 | -1.330242857 | 0.4731 | 0.230769231 |
| 99744 | 649357 | 0.9162 | 0.0838 | -94.119 | 38.119 | SMITHSONIAN | 4 | YES | female | 4.0 mi N & 2.5 mi W Rockville | Bates | Missouri | NA | 4 | Apr | 1980 | -7.86404176 | 0.3228 | 0 |
| 99745 | 649358 | 0.9982 | 0.0018 | -94.119 | 38.119 | SMITHSONIAN | 4 | YES | female | 4.0 mi N & 2.5 mi W Rockville | Bates | Missouri | NA | 4 | Apr | 1980 | -10.70314865 | 0.3242 | 0.026315789 |
| 99746 | 649360 | 0.998 | 0.002 | -94.119 | 38.119 | SMITHSONIAN | 4 | YES | male | 4.0 mi N & 2.5 mi W Rockville | Bates | Missouri | NA | 4 | Apr | 1980 | -7.326197342 | 0.322 | 0.021276596 |
| 99747 | 649361 | 0.999 | 0.001 | -94.119 | 38.119 | SMITHSONIAN | 4 | YES | male | 4.0 mi N & 2.5 mi W Rockville | Bates | Missouri | NA | 4 | Apr | 1980 | -8.88117278 | 0.3067 | 0.023809524 |
| 99748 | 649362 | 0.5956 | 0.4044 | -94.119 | 38.119 | SMITHSONIAN | 4 | YES | male | 4.0 mi N & 2.5 mi W Rockville | Bates | Missouri | NA | 4 | Apr | 1980 | -3.409923988 | 0.4317 | 0.743589744 |
| 99749 | 649363 | 0.5376 | 0.4624 | -94.119 | 38.119 | SMITHSONIAN | 4 | YES | female | 4.0 mi N & 2.5 mi W Rockville | Bates | Missouri | NA | 4 | Apr | 1980 | -1.415680371 | 0.4478 | 0.255813953 |
| 99750 | 649364 | 0.9664 | 0.0336 | -94.119 | 38.119 | SMITHSONIAN | 4 | YES | male | 4.0 mi N & 2.5 mi W Rockville | Bates | Missouri | NA | 4 | Apr | 1980 | -8.992401066 | 0.2861 | 0.020833333 |
| 99751 | 649365 | 0.9658 | 0.0342 | -94.119 | 38.119 | SMITHSONIAN | 4 | YES | female | 4.0 mi N & 2.5 mi W Rockville | Bates | Missouri | NA | 4 | Apr | 1980 | -6.683053163 | 0.316 | 0.022222222 |
| 99752 | 649252 | 0.9238 | 0.0762 | -94.113 | 38.105 | SMITHSONIAN | 6 | YES | unknown | 2.0 mi N & 2.0 mi W Rockville | Bates | Missouri | NA | 5 | Apr | 1980 | -6.536965867 | 0.3 | 0.022222222 |
| 99754 | 649249 | 0 | 1 | -93.682 | 37.845 | SMITHSONIAN | 22 | NO | male | 3.0 mi S & 3.0 mi W Collins | St. Clair | Missouri | NA | 30 | Mar | 1980 | 8.280389493 | 0.684 | 0 |
| 99755 | 649279 | 0.001 | 0.999 | -93.682 | 37.845 | SMITHSONIAN | 22 | NO | female | 3.0 mi S & 3.0 mi W Collins | St. Clair | Missouri | NA | 31 | Mar | 1980 | 11.37366471 | 0.6792 | 0 |
| 99760 | 649278 | 0.001 | 0.999 | -93.682 | 37.845 | SMITHSONIAN | 22 | NO | male | 3.0 mi S & 3.0 mi W Collins | St. Clair | Missouri | NA | 30 | Mar | 1980 | 8.815978351 | 0.6618 | 0.020833333 |
| 99764 | 649342 | 0.001 | 0.999 | -93.682 | 37.845 | SMITHSONIAN | 22 | NO | male | 3.0 mi S & 3.0 mi W Collins | St. Clair | Missouri | NA | 31 | Mar | 1980 | 6.177699636 | 0.6808 | 0 |
| 99765 | 649344 | 0.001 | 0.999 | -93.682 | 37.845 | SMITHSONIAN | 22 | NO | female | 3.0 mi S & 3.0 mi W Collins | St. Clair | Missouri | NA | 31 | Mar | 1980 | 7.839359823 | 0.7016 | 0.02 |
| 99767 | 649346 | 0.001 | 0.999 | -93.682 | 37.845 | SMITHSONIAN | 22 | NO | female | 3.0 mi S & 3.0 mi W Collins | St. Clair | Missouri | NA | 31 | Mar | 1980 | 9.133826796 | 0.686 | 0 |
| 99772 | 649247 | 2.00E-04 | 0.9998 | -93.709 | 37.875 | SMITHSONIAN | 20 | NO | male | 1.0 mi S & 4.5 mi W Collins | St. Clair | Missouri | NA | 29 | Mar | 1980 | 9.709669411 | 0.6878 | 0 |
| 99773 | 649348 | 0.001 | 0.999 | -93.709 | 37.875 | SMITHSONIAN | 20 | NO | female | 1.0 mi S & 4.5 mi W Collins | St. Clair | Missouri | NA | 29 | Mar | 1980 | 10.40065714 | 0.7055 | 0.040816327 |
| 99774 | 649246 | 0.001 | 0.999 | -93.708 | 37.89 | SMITHSONIAN | 21 | NO | male | 4.5 mi W Collins | St. Clair | Missouri | NA | 29 | Mar | 1980 | 5.377991772 | 0.6725 | 0.045454545 |
| 99775 | 649248 | 0.001 | 0.999 | -93.708 | 37.89 | SMITHSONIAN | 21 | NO | male | 4.5 mi W Collins | St. Clair | Missouri | NA | 29 | Mar | 1980 | 6.169466713 | 0.6548 | 0.040816327 |
| 99786 | 649255 | 0.9332 | 0.0668 | -94.01 | 38.183 | SMITHSONIAN | 5 | YES | male | 1.0 mi E & 0.5 mi S Appleton City | St. Clair | Missouri | NA | 3 | Apr | 1980 | -7.912953819 | 0.3377 | 0 |
| 99787 | 649256 | 0.002 | 0.998 | -94.01 | 38.183 | SMITHSONIAN | 5 | YES | female | 1.0 mi E & 0.5 mi S Appleton City | St. Clair | Missouri | NA | 3 | Apr | 1980 | 6.276274141 | 0.6506 | 0 |
| 99788 | 649257 | NA | NA | -94.01 | 38.183 | SMITHSONIAN | 5 | YES | female | 1.0 mi E & 0.5 mi S Appleton City | St. Clair | Missouri | NA | 3 | Apr | 1980 | NA | NA | NA |
| 99789 | 649258 | 0.999 | 0.001 | -94.01 | 38.183 | SMITHSONIAN | 5 | YES | female | 1.0 mi E & 0.5 mi S Appleton City | St. Clair | Missouri | NA | 3 | Apr | 1980 | -11.43913175 | 0.297 | 0 |
| 99790 | 649259 | 0.9996 | 4.00E-04 | -94.01 | 38.183 | SMITHSONIAN | 5 | YES | male | 1.0 mi E & 0.5 mi S Appleton City | St. Clair | Missouri | NA | 3 | Apr | 1980 | -11.93195649 | 0.2675 | 0 |
| 99791 | 649251 | 0.4928 | 0.5072 | -94.01 | 38.183 | SMITHSONIAN | 5 | YES | male | 1.0 mi E & 0.5 mi S Appleton City | St. Clair | Missouri | NA | 3 | Apr | 1980 | -1.981892233 | 0.4675 | 0.529411765 |
| 99792 | 649230 | 0.364 | 0.636 | -93.961 | 38.147 | SMITHSONIAN | 9 | YES | male | 3.5 mi E & 2.5 mi S Appleton City | St. Clair | Missouri | NA | 23 | Apr | 1978 | 1.043185413 | 0.546 | 0.6 |
| 99793 | 649231 | 2.00E-04 | 0.9998 | -93.961 | 38.14 | SMITHSONIAN | 10 | YES | male | 3.5 mi E & 3.0 mi S Appleton City | St. Clair | Missouri | NA | 23 | Apr | 1978 | 9.574354214 | 0.7078 | 0 |
| 99794 | 649240 | 0.0034 | 0.9966 | -93.961 | 38.14 | SMITHSONIAN | 10 | YES | male | 3.5 mi E & 3.0 mi S Appleton City | St. Clair | Missouri | NA | 23 | Apr | 1978 | 6.087512014 | 0.6334 | 0.042553191 |
| 99795 | 649241 | 0.999 | 0.001 | -93.961 | 38.14 | SMITHSONIAN | 10 | YES | female | 3.5 mi E & 3.0 mi S Appleton City | St. Clair | Missouri | NA | 23 | Apr | 1978 | -10.5830196 | 0.2812 | 0 |
| 99796 | 649234 | 0.4658 | 0.5342 | -93.943 | 38.14 | SMITHSONIAN | 14 | YES | male | 4.5 mi E & 3.0 mi S Appleton City | St. Clair | Missouri | NA | 23 | Apr | 1978 | -0.60643262 | 0.5049 | 0.695652174 |
| 99797 | 649239 | 0.9772 | 0.0228 | -93.979 | 38.13 | SMITHSONIAN | 13 | YES | male | 4.0 mi S & 2.5 mi E Appleton City | St. Clair | Missouri | NA | 22 | Apr | 1978 | -8.138476353 | 0.3088 | 0.045454545 |
| 99798 | 649232 | 0.4472 | 0.5528 | -93.961 | 38.147 | SMITHSONIAN | 9 | YES | male | 3.5 mi E & 2.5 mi S Appleton City | St. Clair | Missouri | NA | 23 | Apr | 1978 | 0.079146301 | 0.4992 | 0.695652174 |
| 99800 | 649315 | 0.001 | 0.999 | -94.054 | 38.065 | SMITHSONIAN | 18 | YES | female | Campbell Branch Cr., 1.0 mi E Rockville | St. Clair | Missouri | NA | 2 | Apr | 1980 | 6.418584662 | 0.6506 | 0 |
| 99801 | 649323 | 0.457 | 0.543 | -94.054 | 38.065 | SMITHSONIAN | 18 | YES | male | Campbell Branch Cr., 1.0 mi E Rockville | St. Clair | Missouri | NA | 31 | Mar | 1980 | -0.530365403 | 0.4873 | 0.647058824 |
| 99802 | 649313 | 0.002 | 0.998 | -94.054 | 38.065 | SMITHSONIAN | 18 | YES | male | Campbell Branch Cr., 1.0 mi E Rockville | St. Clair | Missouri | NA | 2 | Apr | 1980 | 9.372990102 | 0.6506 | 0.02173913 |
| 99803 | 649314 | 0.479 | 0.521 | -94.054 | 38.065 | SMITHSONIAN | 18 | YES | male | Campbell Branch Cr., 1.0 mi E Rockville | St. Clair | Missouri | NA | 2 | Apr | 1980 | 1.082555437 | 0.4868 | 0.681818182 |
| 99804 | 649317 | 0.001 | 0.999 | -94.055 | 38.105 | SMITHSONIAN | 8 | YES | female | Campbell Branch Cr., 2.0 mi N & 1.0 mi E Rockville | St. Clair | Missouri | NA | 2 | Apr | 1980 | 6.368601454 | 0.6657 | 0.026315789 |
| 99805 | 649318 | 0.545 | 0.455 | -94.054 | 38.065 | SMITHSONIAN | 18 | YES | female | Campbell Branch Cr., 1.0 mi E Rockville | St. Clair | Missouri | NA | 2 | Apr | 1980 | -1.91825212 | 0.4672 | 0.612244898 |
| 99806 | 649319 | 0.999 | 0.001 | -94.054 | 38.065 | SMITHSONIAN | 18 | YES | female | Campbell Branch Cr., 1.0 mi E Rockville | St. Clair | Missouri | NA | 2 | Apr | 1980 | -6.728009643 | 0.3146 | 0 |
| 99807 | 649320 | 0.9958 | 0.0042 | -94.054 | 38.065 | SMITHSONIAN | 18 | YES | female | Campbell Branch Cr., 1.0 mi E Rockville | St. Clair | Missouri | NA | 2 | Apr | 1980 | -7.619606378 | 0.3392 | 0 |
| 99808 | 649321 | 0 | 1 | -94.054 | 38.065 | SMITHSONIAN | 18 | YES | female | Campbell Branch Cr., 1.0 mi E Rockville | St. Clair | Missouri | NA | 2 | Apr | 1980 | 8.98630614 | 0.6787 | 0.02 |
| 99809 | 649322 | 0.5542 | 0.4458 | -94.054 | 38.065 | SMITHSONIAN | 18 | YES | female | Campbell Branch Cr., 1.0 mi E Rockville | St. Clair | Missouri | NA | 2 | Apr | 1980 | -3.053101188 | 0.4421 | 0.203703704 |
| 99810 | 649324 | 0.4616 | 0.5384 | -94.054 | 38.065 | SMITHSONIAN | 18 | YES | male | Campbell Branch Cr., 1.0 mi E Rockville | St. Clair | Missouri | NA | 2 | Apr | 1980 | 0.252714947 | 0.514 | 0.653061224 |
| 99811 | 649316 | 0.0034 | 0.9966 | -94.055 | 38.105 | SMITHSONIAN | 8 | YES | male | Campbell Branch Cr., 2.0 mi N & 1.0 mi E Rockville | St. Clair | Missouri | NA | 2 | Apr | 1980 | 8.72572009 | 0.64 | 0 |
| 99812 | 649253 | 0.001 | 0.999 | -94.011 | 38 | SMITHSONIAN | 19 | YES | male | 0.5 mi S & 1.0 mi W Taberville | St. Clair | Missouri | NA | 5 | Apr | 1980 | 6.008556977 | 0.6958 | 0 |
| 99813 | 649254 | 0.001 | 0.999 | -94.011 | 38 | SMITHSONIAN | 19 | YES | female | 0.5 mi S & 1.0 mi W Taberville | St. Clair | Missouri | NA | 5 | Apr | 1980 | 8.88966394 | 0.6765 | 0 |
| 99851 | 649235 | 0.999 | 0.001 | -94.439 | 37.565 | SMITHSONIAN | 15 | NO | male | 4.5 mi E Liberal | Barton | Missouri | NA | 15 | Apr | 1978 | -7.418861755 | 0.3128 | 0.023255814 |
| 99854 | 649229 | 0.001 | 0.999 | -94.089 | 38.075 | SMITHSONIAN | 7 | YES | male | 0.25 mi W Rockville | Bates | Missouri | NA | 22 | Apr | 1978 | 6.89644078 | 0.6703 | 0.024390244 |
| 99858 | 649236 | 0.4724 | 0.5276 | -94.424 | 37.755 | SMITHSONIAN | 11 | NO | male | 1.5 mi E Moundville | Vernon | Missouri | NA | 15 | Apr | 1978 | -2.781346285 | 0.5109 | 0.630434783 |
| 99859 | 649233 | 0.001 | 0.999 | -94.406 | 37.757 | SMITHSONIAN | 12 | NO | male | 2.5 mi E Moundville | Vernon | Missouri | NA | 15 | Apr | 1978 | 6.916356145 | 0.6447 | 0.025 |
| 99861 | 649237 | 0.0076 | 0.9924 | -94.425 | 37.7 | SMITHSONIAN | 16 | NO | male | 2.5 mi E & 0.5 mi N Bronaugh | Vernon | Missouri | NA | 15 | Apr | 1978 | 6.936380907 | 0.6734 | 0.041666667 |
| 99862 | 649238 | 0.9916 | 0.0084 | -94.424 | 37.707 | SMITHSONIAN | 17 | NO | male | 2.5 mi E & 1.0 mi N Bronaugh | Vernon | Missouri | NA | 15 | Apr | 1978 | -8.171663253 | 0.2966 | 0 |

# Table S2

Oligo/barcode combinations for the samples sequenced in this study. A) gives the internal P1 barcode oligos; B) gives the external PCR2 barcode oligos, and C) gives the combination of these barcodes used for each sample as identified by the **Tissue_number** variable also found in [**Table S1**](#_Fig._S15.). PCR primer 1 (AATGATACGGCGACCACCGAGATCTACACTCTTTCCCTACACGACG) and the *MspI* adaptor were common to all samples. The *MspI* adaptor was comprised of the following oligos:

MspI_P2.1 GTGACTGGAGTTCAGACGTGTGCTCTTCCGATCT

MspI_P2.2 /5Phos/CGAGATCGGAAGAGCGAGAACAA [[back to *Contents*](#_top)]

A)

| P1 barcode name | P1 barcode | P1.1 sequence | P1.2 sequence |
| --- | --- | --- | --- |
| SbfI_1A | ACACA | ACACTCTTTCCCTACACGACGCTCTTCCGATCTACACATGCA | /5Phos/TGTGTAGATCGGAAGAGCGTCGTGTAGGGAAAGAGTGT |
| SbfI_5E | ACGGT | ACACTCTTTCCCTACACGACGCTCTTCCGATCTACGGTTGCA | /5Phos/ACCGTAGATCGGAAGAGCGTCGTGTAGGGAAAGAGTGT |
| SbfI_3A | ACTGG | ACACTCTTTCCCTACACGACGCTCTTCCGATCTACTGGTGCA | /5Phos/CCAGTAGATCGGAAGAGCGTCGTGTAGGGAAAGAGTGT |
| SbfI_1H | AGCTA | ACACTCTTTCCCTACACGACGCTCTTCCGATCTAGCTATGCA | /5Phos/TAGCTAGATCGGAAGAGCGTCGTGTAGGGAAAGAGTGT |
| SbfI_3F | ATGAG | ACACTCTTTCCCTACACGACGCTCTTCCGATCTATGAGTGCA | /5Phos/CTCATAGATCGGAAGAGCGTCGTGTAGGGAAAGAGTGT |
| SbfI_1B | CGATC | ACACTCTTTCCCTACACGACGCTCTTCCGATCTCGATCTGCA | /5Phos/GATCGAGATCGGAAGAGCGTCGTGTAGGGAAAGAGTGT |
| SbfI_1E | CGGTA | ACACTCTTTCCCTACACGACGCTCTTCCGATCTCGGTATGCA | /5Phos/TACCGAGATCGGAAGAGCGTCGTGTAGGGAAAGAGTGT |
| SbfI_1G | CGTAC | ACACTCTTTCCCTACACGACGCTCTTCCGATCTCGTACTGCA | /5Phos/GTACGAGATCGGAAGAGCGTCGTGTAGGGAAAGAGTGT |
| SbfI_1F | CTGAT | ACACTCTTTCCCTACACGACGCTCTTCCGATCTCTGATTGCA | /5Phos/ATCAGAGATCGGAAGAGCGTCGTGTAGGGAAAGAGTGT |
| SbfI_1D | GCCGT | ACACTCTTTCCCTACACGACGCTCTTCCGATCTGCCGTTGCA | /5Phos/ACGGCAGATCGGAAGAGCGTCGTGTAGGGAAAGAGTGT |
| SbfI_3G | GGATA | ACACTCTTTCCCTACACGACGCTCTTCCGATCTGGATATGCA | /5Phos/TATCCAGATCGGAAGAGCGTCGTGTAGGGAAAGAGTGT |
| SbfI_3E | GGTTG | ACACTCTTTCCCTACACGACGCTCTTCCGATCTGGTTGTGCA | /5Phos/CAACCAGATCGGAAGAGCGTCGTGTAGGGAAAGAGTGT |
| SbfI_6A | GTCCG | ACACTCTTTCCCTACACGACGCTCTTCCGATCTGTCCGTGCA | /5Phos/CGGACAGATCGGAAGAGCGTCGTGTAGGGAAAGAGTGT |
| SbfI_1C | TACCG | ACACTCTTTCCCTACACGACGCTCTTCCGATCTTACCGTGCA | /5Phos/CGGTAAGATCGGAAGAGCGTCGTGTAGGGAAAGAGTGT |
| SbfI_4A | TATAC | ACACTCTTTCCCTACACGACGCTCTTCCGATCTTATACTGCA | /5Phos/GTATAAGATCGGAAGAGCGTCGTGTAGGGAAAGAGTGT |
| SbfI_3H | TCAGT | ACACTCTTTCCCTACACGACGCTCTTCCGATCTTCAGTTGCA | /5Phos/ACTGAAGATCGGAAGAGCGTCGTGTAGGGAAAGAGTGT |

B)

| PCR2 primer name | PCR2 barcode | PCR2 primer sequence |
| --- | --- | --- |
| PCR2(1)* | ATCACG | CAAGCAGAAGACGGCATACGAGATCGTGATGTGACTGGAGTTCAGACGTGTGC |
| PCR2(2)* | CGATGT | CAAGCAGAAGACGGCATACGAGATACATCGGTGACTGGAGTTCAGACGTGTGC |
| PCR2(3) | TTAGGC | CAAGCAGAAGACGGCATACGAGATGCCTAAGTGACTGGAGTTCAGACGTGTGC |
| PCR2(4) | TGACCA | CAAGCAGAAGACGGCATACGAGATTGGTCAGTGACTGGAGTTCAGACGTGTGC |
| PCR2(5) | ACAGTG | CAAGCAGAAGACGGCATACGAGATCACTGTGTGACTGGAGTTCAGACGTGTGC |
| PCR2(6) | GCCAAT | CAAGCAGAAGACGGCATACGAGATATTGGCGTGACTGGAGTTCAGACGTGTGC |
| PCR2(7) | CAGATC | CAAGCAGAAGACGGCATACGAGATGATCTGGTGACTGGAGTTCAGACGTGTGC |
| PCR2(8) | ACTTGA | CAAGCAGAAGACGGCATACGAGATTCAAGTGTGACTGGAGTTCAGACGTGTGC |
| PCR2(9) | GATCAG | CAAGCAGAAGACGGCATACGAGATCTGATCGTGACTGGAGTTCAGACGTGTGC |
| PCR2(10) | TAGCTT | CAAGCAGAAGACGGCATACGAGATAAGCTAGTGACTGGAGTTCAGACGTGTGC |
| PCR2(11) | GGCTAC | CAAGCAGAAGACGGCATACGAGATGTAGCCGTGACTGGAGTTCAGACGTGTGC |
| PCR2(12) | CTTGTA | CAAGCAGAAGACGGCATACGAGATTACAAGGTGACTGGAGTTCAGACGTGTGC |

* These primers used for samples from unrelated projects sequenced on the main chickadee sequencing run, so do not appear in the table below.

C)

| Tissue number | P1 barcode name | PCR2 primer name |  | Tissue number | P1 barcode name | PCR2 primer name |  | Tissue number | P1 barcode name | PCR2 primer name |
| --- | --- | --- | --- | --- | --- | --- | --- | --- | --- | --- |
| 649232 | SbfI_1A | PCR2 (3) |  | 649313 | SbfI_3E | PCR2 (6) |  | 32577 | SbfI_1A | PCR2 (10) |
| 649258 | SbfI_1B | PCR2 (3) |  | 649331 | SbfI_3F | PCR2 (6) |  | 32600 | SbfI_1B | PCR2 (10) |
| 649251 | SbfI_1C | PCR2 (3) |  | 649356 | SbfI_3G | PCR2 (6) |  | 32562 | SbfI_1C | PCR2 (10) |
| 649233 | SbfI_1D | PCR2 (3) |  | 649326 | SbfI_3H | PCR2 (6) |  | 32616 | SbfI_1D | PCR2 (10) |
| 649314 | SbfI_1E | PCR2 (3) |  | 649364 | SbfI_4A | PCR2 (6) |  | 32598 | SbfI_1E | PCR2 (10) |
| 649229 | SbfI_1F | PCR2 (3) |  | 649278 | SbfI_5E | PCR2 (6) |  | 32608 | SbfI_1F | PCR2 (10) |
| 649252 | SbfI_1G | PCR2 (3) |  | 649319 | SbfI_6A | PCR2 (6) |  | 32556 | SbfI_1G | PCR2 (10) |
| 649236 | SbfI_1H | PCR2 (3) |  | 649259 | SbfI_1A | PCR2 (7) |  | 32590 | SbfI_1H | PCR2 (10) |
| 649365 | SbfI_3A | PCR2 (3) |  | 32548 | SbfI_1B | PCR2 (7) |  | 32594 | SbfI_3A | PCR2 (10) |
| 649253 | SbfI_3E | PCR2 (3) |  | 32542 | SbfI_1C | PCR2 (7) |  | 32591 | SbfI_3E | PCR2 (10) |
| 649234 | SbfI_3G | PCR2 (3) |  | 649328 | SbfI_1D | PCR2 (7) |  | 32564 | SbfI_3F | PCR2 (10) |
| 649230 | SbfI_4A | PCR2 (3) |  | 32550 | SbfI_1E | PCR2 (7) |  | 32576 | SbfI_3G | PCR2 (10) |
| 649240 | SbfI_5E | PCR2 (3) |  | 32537 | SbfI_1F | PCR2 (7) |  | 32554 | SbfI_3H | PCR2 (10) |
| 649239 | SbfI_6A | PCR2 (3) |  | 649359 | SbfI_1G | PCR2 (7) |  | 32559 | SbfI_4A | PCR2 (10) |
| 649238 | SbfI_1A | PCR2 (4) |  | 32538 | SbfI_1H | PCR2 (7) |  | 32552 | SbfI_5E | PCR2 (10) |
| 649235 | SbfI_1B | PCR2 (4) |  | 32541 | SbfI_3A | PCR2 (7) |  | 32605 | SbfI_6A | PCR2 (10) |
| 649348 | SbfI_1C | PCR2 (4) |  | 649329 | SbfI_3E | PCR2 (7) |  | 32599 | SbfI_1A | PCR2 (11) |
| 649322 | SbfI_1D | PCR2 (4) |  | 649317 | SbfI_3F | PCR2 (7) |  | 32558 | SbfI_1B | PCR2 (11) |
| 649315 | SbfI_1E | PCR2 (4) |  | 32543 | SbfI_3G | PCR2 (7) |  | 29898 | SbfI_1C | PCR2 (11) |
| 649323 | SbfI_1F | PCR2 (4) |  | 649248 | SbfI_3H | PCR2 (7) |  | 32612 | SbfI_1D | PCR2 (11) |
| 649231 | SbfI_1G | PCR2 (4) |  | 32535 | SbfI_4A | PCR2 (7) |  | 32579 | SbfI_1E | PCR2 (11) |
| 649360 | SbfI_1H | PCR2 (4) |  | 32539 | SbfI_5E | PCR2 (7) |  | 32602 | SbfI_1F | PCR2 (11) |
| 649237 | SbfI_3A | PCR2 (4) |  | 32549 | SbfI_6A | PCR2 (7) |  | 32578 | SbfI_1G | PCR2 (11) |
| 649254 | SbfI_3E | PCR2 (4) |  | 32540 | SbfI_1A | PCR2 (8) |  | 32606 | SbfI_1H | PCR2 (11) |
| 649257^ | SbfI_3F | PCR2 (4) |  | 32621 | SbfI_1B | PCR2 (8) |  | 32593 | SbfI_3A | PCR2 (11) |
| 649344 | SbfI_3G | PCR2 (4) |  | 32582 | SbfI_1C | PCR2 (8) |  | 32592 | SbfI_3E | PCR2 (11) |
| 649325 | SbfI_3H | PCR2 (4) |  | 32597 | SbfI_1D | PCR2 (8) |  | 32566 | SbfI_3F | PCR2 (11) |
| 649332 | SbfI_4A | PCR2 (4) |  | 32545 | SbfI_1E | PCR2 (8) |  | 7421 | SbfI_3G | PCR2 (11) |
| 649321 | SbfI_5E | PCR2 (4) |  | 32622 | SbfI_1F | PCR2 (8) |  | 32624 | SbfI_3H | PCR2 (11) |
| 649255 | SbfI_6A | PCR2 (4) |  | 32613 | SbfI_1G | PCR2 (8) |  | 7420 | SbfI_4A | PCR2 (11) |
| 649320 | SbfI_1A | PCR2 (5) |  | 32546 | SbfI_1H | PCR2 (8) |  | 32563 | SbfI_5E | PCR2 (11) |
| 649247 | SbfI_1B | PCR2 (5) |  | 32561 | SbfI_3A | PCR2 (8) |  | 32609 | SbfI_6A | PCR2 (11) |
| 649256 | SbfI_1C | PCR2 (5) |  | 32611 | SbfI_3E | PCR2 (8) |  | 32585 | SbfI_1A | PCR2 (12) |
| 649330 | SbfI_1D | PCR2 (5) |  | 32536 | SbfI_3F | PCR2 (8) |  | 32589* | SbfI_1B | PCR2 (12) |
| 649363 | SbfI_1E | PCR2 (5) |  | 32580 | SbfI_3G | PCR2 (8) |  | 32610 | SbfI_1B | PCR2 (12) |
| 649357 | SbfI_1F | PCR2 (5) |  | 32617 | SbfI_3H | PCR2 (8) |  | 32573* | SbfI_1C | PCR2 (12) |
| 649318 | SbfI_1G | PCR2 (5) |  | 32547 | SbfI_4A | PCR2 (8) |  | 32607 | SbfI_1C | PCR2 (12) |
| 649327 | SbfI_1H | PCR2 (5) |  | 32626 | SbfI_5E | PCR2 (8) |  | 32560 | SbfI_1D | PCR2 (12) |
| 649355 | SbfI_3A | PCR2 (5) |  | 32625 | SbfI_6A | PCR2 (8) |  | 32571 | SbfI_1E | PCR2 (12) |
| 649358 | SbfI_3E | PCR2 (5) |  | 32623 | SbfI_1A | PCR2 (9) |  | 32569* | SbfI_1F | PCR2 (12) |
| 649324 | SbfI_3F | PCR2 (5) |  | 32555 | SbfI_1B | PCR2 (9) |  | 6281 | SbfI_1F | PCR2 (12) |
| 649346 | SbfI_3G | PCR2 (5) |  | 32588 | SbfI_1C | PCR2 (9) |  | 32583 | SbfI_1G | PCR2 (12) |
| 649352 | SbfI_3H | PCR2 (5) |  | 32551 | SbfI_1D | PCR2 (9) |  | 32586 | SbfI_1H | PCR2 (12) |
| 649241 | SbfI_4A | PCR2 (5) |  | 32595 | SbfI_1E | PCR2 (9) |  | 32587* | SbfI_3A | PCR2 (12) |
| 649316 | SbfI_5E | PCR2 (5) |  | 32618 | SbfI_1F | PCR2 (9) |  | 3474 | SbfI_3A | PCR2 (12) |
| 649353 | SbfI_6A | PCR2 (5) |  | 32601 | SbfI_1G | PCR2 (9) |  | 32603 | SbfI_3E | PCR2 (12) |
| 649249 | SbfI_1A | PCR2 (6) |  | 32557 | SbfI_1H | PCR2 (9) |  | 32567* | SbfI_3G | PCR2 (12) |
| 649354 | SbfI_1B | PCR2 (6) |  | 32581 | SbfI_3A | PCR2 (9) |  | 32565 | SbfI_3G | PCR2 (12) |
| 649334 | SbfI_1C | PCR2 (6) |  | 32619 | SbfI_3E | PCR2 (9) |  | 32574 | SbfI_3H | PCR2 (12) |
| 649361 | SbfI_1D | PCR2 (6) |  | 32614 | SbfI_3F | PCR2 (9) |  | 32584* | SbfI_4A | PCR2 (12) |
| 649362 | SbfI_1E | PCR2 (6) |  | 32572 | SbfI_3G | PCR2 (9) |  | 32553 | SbfI_4A | PCR2 (12) |
| 649246 | SbfI_1F | PCR2 (6) |  | 32615 | SbfI_3H | PCR2 (9) |  | 32534* | SbfI_5E | PCR2 (12) |
| 649342 | SbfI_1G | PCR2 (6) |  | 32604 | SbfI_4A | PCR2 (9) |  | 32568 | SbfI_5E | PCR2 (12) |
| 649279 | SbfI_1H | PCR2 (6) |  | 32620 | SbfI_5E | PCR2 (9) |  | 32570* | SbfI_6A | PCR2 (12) |
| 649333 | SbfI_3A | PCR2 (6) |  | 32596 | SbfI_6A | PCR2 (9) |  | 32575 | SbfI_6A | PCR2 (12) |

Samples denoted by * were sequenced separately on the test run, so some duplicate P1/PCR2 barcode combinations are present. ^ denotes “649257”, the sample excluded from downstream analyses due to low sequencing depth.

# Table S3

Sampling sites in Missouri and Pennsylvania used for inferring climatic trends from PRISM data, as presented in **Fig. 3**. [[back to *Contents*](#_top)]

| **Site** | **Latitude** | **Longitude** |
| --- | --- | --- |
| Rockville | 38.07 | -94.08 |
| Appleton City | 38.19 | -94.03 |
| Tuscarora State Forest | 40.80 | -76.03 |
| Hawk Mountain | 40.65 | -76.00 |
| Nolde Forest | 40.28 | -75.96 |
| Great Marsh | 40.14 | -75.74 |
| Villanova University campus | 40.04 | -75.34 |

# Table S4

Sample-specific RADseq assembly information. The first two columns map to [**Table S1**](#_Table_S1.), the remainder of the columns are taken directly from the associated ipyrad *_ref_stats.txt file, sorted on decreasing read count (reads_raw). Columns used for the calculations in the results text are reads_raw, clusters_total, and loci_in_assembly. The first eight samples in the table correspond to the initial test set of 8 samples sequenced at higher coverage. The last sample in the table (Catalog number: 99788; Tissue number: 649257) is the historical sample excluded due to low coverage. A *tab delimited version of this table is available for download at: <https://github.com/laninsky/chickadees/blob/master/data/Table_S4.txt> [[back to *Contents*](#_top)]

| Catalog_number | Tissue_number | Sample | state | reads_raw | reads_passed_filter | refseq_mapped_reads | refseq_unmapped_reads | clusters_total | clusters_hidepth | hetero_est | error_est | reads_consens | loci_in_assembly |
| --- | --- | --- | --- | --- | --- | --- | --- | --- | --- | --- | --- | --- | --- |
| 132100 | 32587 | 132100_32587 | 7 | 3332843 | 3316518 | 3111068 | 205450 | 43697 | 16370 | 0.006856 | 0.003378 | 15390 | 10239 |
| 132084 | 32573 | 132084_32573 | 7 | 3182468 | 3166738 | 2966882 | 199856 | 38058 | 15834 | 0.006404 | 0.003552 | 14960 | 10289 |
| 132109 | 32569 | 132109_32569 | 7 | 2979852 | 2965821 | 2756892 | 208929 | 31322 | 15333 | 0.006423 | 0.003336 | 14582 | 10244 |
| 132102 | 32567 | 132102_32567 | 7 | 2830768 | 2817414 | 2620968 | 196446 | 34521 | 15357 | 0.007022 | 0.00357 | 14601 | 10247 |
| 132114 | 32534 | 132114_32534 | 7 | 2828667 | 2813115 | 2622649 | 190466 | 28183 | 15610 | 0.006605 | 0.003578 | 14855 | 10325 |
| 132097 | 32589 | 132097_32589 | 7 | 2375262 | 2363011 | 2205933 | 157078 | 33590 | 14971 | 0.006672 | 0.003621 | 14223 | 10127 |
| 132108 | 32570 | 132108_32570 | 7 | 2366950 | 2353464 | 2186331 | 167133 | 43273 | 15496 | 0.007412 | 0.00338 | 14575 | 10119 |
| 132099 | 32584 | 132099_32584 | 7 | 2188989 | 2175013 | 2031855 | 143158 | 35879 | 14927 | 0.006757 | 0.003349 | 14202 | 10095 |
| 132043 | 32565 | 132043_32565 | 7 | 1146352 | 1141828 | 1083507 | 58321 | 20708 | 13812 | 0.007407 | 0.001198 | 13241 | 9959 |
| 132086 | 32575 | 132086_32575 | 7 | 1064097 | 1059243 | 1000756 | 58487 | 24980 | 14149 | 0.007494 | 0.00123 | 13465 | 9986 |
| 132082 | 32577 | 132082_32577 | 7 | 985054 | 976205 | 901506 | 74699 | 27704 | 14325 | 0.006134 | 0.002382 | 13780 | 10409 |
| 132091 | 32585 | 132091_32585 | 7 | 982890 | 977914 | 920554 | 57360 | 22923 | 14478 | 0.007801 | 0.00124 | 13833 | 10015 |
| 132080 | 32594 | 132080_32594 | 7 | 962895 | 953725 | 886099 | 67626 | 36393 | 14633 | 0.006169 | 0.002398 | 14051 | 10299 |
| 132090 | 32586 | 132090_32586 | 7 | 960031 | 955762 | 905618 | 50144 | 19687 | 13817 | 0.00746 | 0.001247 | 13245 | 9894 |
| 132095 | 32571 | 132095_32571 | 7 | 940560 | 935592 | 892056 | 43536 | 21416 | 13387 | 0.007486 | 0.00126 | 12856 | 9819 |
| 132081 | 32576 | 132081_32576 | 7 | 937417 | 931263 | 858248 | 73015 | 28867 | 14293 | 0.005935 | 0.002372 | 13770 | 10305 |
| 132089 | 32560 | 132089_32560 | 7 | 924505 | 920601 | 863700 | 56901 | 20465 | 13459 | 0.00781 | 0.001335 | 12897 | 9802 |
| 132072 | 32603 | 132072_32603 | 7 | 916841 | 911659 | 859713 | 51946 | 21460 | 13877 | 0.007718 | 0.001275 | 13323 | 9916 |
| 90612 | 3474 | 3474_ | 7 | 893929 | 885907 | 837730 | 48177 | 49302 | 15162 | 0.007855 | 0.001166 | 13813 | 9928 |
| 132115 | 32543 | 132115_32543 | 7 | 885743 | 880397 | 806568 | 73829 | 23021 | 13503 | 0.006257 | 0.002717 | 13080 | 9972 |
| 132098 | 32590 | 132098_32590 | 7 | 881900 | 874863 | 805839 | 69024 | 28302 | 14403 | 0.006568 | 0.002367 | 13840 | 10353 |
| 132073 | 32593 | 132073_32593 | 7 | 880203 | 872439 | 804983 | 67456 | 31576 | 14064 | 0.006118 | 0.00274 | 13520 | 10115 |
| 132041 | 32562 | 132041_32562 | 7 | 874776 | 868525 | 799485 | 69040 | 33512 | 14447 | 0.006184 | 0.002439 | 13883 | 10285 |
| 92270 | 7421 | 7421_ | 7 | 870759 | 864312 | 790030 | 74282 | 27400 | 13691 | 0.006299 | 0.00269 | 13237 | 10644 |
| 132092 | 32553 | 132092_32553 | 7 | 869054 | 865308 | 761112 | 104196 | 21739 | 14861 | 0.007494 | 0.001252 | 12854 | 9698 |
| 132107 | 32580 | 132107_32580 | 7 | 868304 | 863848 | 808377 | 55471 | 25345 | 14578 | 0.006551 | 0.002004 | 13977 | 10315 |
| 132101 | 32588 | 132101_32588 | 7 | 863305 | 856371 | 760034 | 96337 | 28520 | 12321 | 0.006485 | 0.003601 | 11896 | 9004 |
| 132124 | 32552 | 132124_32552 | 7 | 853025 | 846735 | 779103 | 67632 | 26822 | 14292 | 0.006397 | 0.002573 | 13762 | 10355 |
| 132093 | 32568 | 132093_32568 | 7 | 852448 | 848722 | 808280 | 40442 | 20442 | 13215 | 0.007436 | 0.001233 | 12731 | 9709 |
| 131638 | 29898 | 9898_ | 7 | 844626 | 838898 | 769794 | 69104 | 26095 | 13890 | 0.005898 | 0.002771 | 13404 | 10385 |
| 99790 | 649259 | 649259_ | 7 | 837663 | 831748 | 758217 | 73531 | 22383 | 12993 | 0.005861 | 0.002695 | 12614 | 9699 |
| 132066 | 32599 | 132066_32599 | 7 | 833622 | 824869 | 750089 | 74780 | 28214 | 13785 | 0.006251 | 0.00263 | 13275 | 10141 |
| 99754 | 649249 | 649249_ | 7 | 830496 | 824235 | 764542 | 59693 | 26650 | 14438 | 0.005862 | 0.002027 | 13858 | 10245 |
| 132074 | 32591 | 132074_32591 | 7 | 829845 | 820003 | 761510 | 58493 | 28029 | 14060 | 0.005981 | 0.00238 | 13552 | 10250 |
| 132117 | 32583 | 132117_32583 | 7 | 822175 | 817063 | 770852 | 46211 | 20233 | 13614 | 0.008191 | 0.001311 | 13005 | 9766 |
| 132106 | 32541 | 132106_32541 | 7 | 819427 | 813839 | 739527 | 74312 | 23365 | 13338 | 0.006149 | 0.002727 | 12910 | 9833 |
| 132077 | 32607 | 132077_32607 | 7 | 815701 | 811847 | 769296 | 42551 | 22154 | 14005 | 0.007791 | 0.001294 | 13429 | 9786 |
| 99743 | 649356 | 649356_ | 7 | 808115 | 803962 | 750981 | 52981 | 23870 | 14470 | 0.006368 | 0.001985 | 13897 | 10267 |
| 132045 | 32605 | 132045_32605 | 7 | 797436 | 790257 | 727612 | 62645 | 35566 | 14300 | 0.006085 | 0.002392 | 13722 | 10295 |
| 132057 | 32623 | 132057_32623 | 7 | 797349 | 789453 | 703071 | 86382 | 23182 | 12254 | 0.006411 | 0.003412 | 11853 | 9189 |
| 132069 | 32610 | 132069_32610 | 7 | 788080 | 784695 | 741703 | 42992 | 19893 | 13185 | 0.007533 | 0.001202 | 12655 | 9653 |
| 132070 | 32598 | 132070_32598 | 7 | 782672 | 775344 | 688811 | 86533 | 36547 | 14570 | 0.006599 | 0.002554 | 13488 | 10171 |
| 132105 | 32542 | 132105_32542 | 7 | 759060 | 754200 | 686888 | 67312 | 20685 | 13358 | 0.006617 | 0.002838 | 12947 | 9860 |
| 95776 | 6281 | 6281_ | 7 | 758006 | 754334 | 713357 | 40977 | 19560 | 13137 | 0.007705 | 0.001281 | 12619 | 9924 |
| 132076 | 32608 | 132076_32608 | 7 | 749031 | 743386 | 684311 | 59075 | 27304 | 14252 | 0.006408 | 0.002557 | 13743 | 10393 |
| 132129 | 32540 | 132129_32540 | 7 | 746092 | 740728 | 689241 | 51487 | 21389 | 14140 | 0.006359 | 0.002069 | 13615 | 10161 |
| 132061 | 32612 | 132061_32612 | 7 | 735626 | 729068 | 673064 | 56004 | 30798 | 13495 | 0.006346 | 0.002888 | 13007 | 10050 |
| 99773 | 649348 | 649348_ | 7 | 731476 | 726481 | 657820 | 68661 | 22535 | 13791 | 0.006153 | 0.002711 | 13359 | 10118 |
| 132096 | 32574 | 132096_32574 | 7 | 728415 | 724772 | 685493 | 39279 | 22543 | 13798 | 0.008324 | 0.001278 | 13241 | 9666 |
| 132075 | 32592 | 132075_32592 | 7 | 724865 | 715646 | 648225 | 67421 | 33402 | 13630 | 0.006537 | 0.002691 | 13121 | 9941 |
| 132088 | 32559 | 132088_32559 | 7 | 720703 | 713138 | 656782 | 56356 | 27049 | 13882 | 0.006113 | 0.002481 | 13389 | 10216 |
| 132132 | 32539 | 132132_32539 | 7 | 718773 | 713801 | 656810 | 56991 | 21760 | 13134 | 0.006333 | 0.002947 | 12747 | 9814 |
| 99807 | 649320 | 649320_ | 7 | 713476 | 707421 | 650600 | 56821 | 27934 | 14320 | 0.005988 | 0.002395 | 13626 | 10209 |
| 132122 | 32538 | 132122_32538 | 7 | 711568 | 706450 | 652324 | 54126 | 19234 | 13090 | 0.006204 | 0.002788 | 12722 | 9744 |
| 99767 | 649346 | 649346_ | 7 | 708056 | 697702 | 642920 | 54782 | 38534 | 14251 | 0.006476 | 0.002353 | 13611 | 10125 |
| 99717 | 649334 | 649334_ | 7 | 706472 | 702268 | 658523 | 43745 | 27271 | 14228 | 0.005799 | 0.002035 | 13710 | 10175 |
| 99746 | 649360 | 649360_ | 7 | 703281 | 698514 | 643288 | 55226 | 19904 | 13586 | 0.005913 | 0.002618 | 13203 | 10175 |
| 99791 | 649251 | 649251_ | 7 | 702006 | 697605 | 651839 | 45766 | 22812 | 13805 | 0.00674 | 0.002041 | 13328 | 10035 |
| 132126 | 32547 | 132126_32547 | 7 | 699511 | 694147 | 646560 | 47587 | 24201 | 14052 | 0.00648 | 0.002162 | 13578 | 10180 |
| 132068 | 32609 | 132068_32609 | 7 | 693127 | 686702 | 622880 | 63822 | 30414 | 13788 | 0.006405 | 0.002713 | 13255 | 10028 |
| 99796 | 649234 | 649234_ | 7 | 690520 | 684108 | 637839 | 46269 | 33417 | 13674 | 0.00667 | 0.002016 | 13131 | 9968 |
| 132049 | 32602 | 132049_32602 | 7 | 687303 | 682160 | 615236 | 66924 | 26402 | 13287 | 0.006429 | 0.002917 | 12830 | 9868 |
| 132104 | 32579 | 132104_32579 | 7 | 681286 | 674611 | 610037 | 64574 | 32131 | 13516 | 0.00643 | 0.002789 | 13007 | 9949 |
| 99716 | 649333 | 649333_ | 7 | 676401 | 671419 | 624818 | 46601 | 27300 | 14071 | 0.005991 | 0.002091 | 13551 | 10069 |
| 99786 | 649255 | 649255_ | 7 | 672756 | 666037 | 613990 | 52047 | 24989 | 13654 | 0.006015 | 0.002705 | 13263 | 10087 |
| 132123 | 32551 | 132123_32551 | 7 | 672241 | 667113 | 549616 | 117497 | 23729 | 11532 | 0.006021 | 0.003861 | 10916 | 8419 |
| 132119 | 32554 | 132119_32554 | 7 | 671375 | 663600 | 610127 | 53473 | 27385 | 13721 | 0.006302 | 0.002572 | 13254 | 10154 |
| 132047 | 32563 | 132047_32563 | 7 | 668776 | 662378 | 601218 | 61160 | 25843 | 13391 | 0.00612 | 0.002663 | 12944 | 10015 |
| 132059 | 32616 | 132059_32616 | 7 | 668333 | 664004 | 605033 | 58971 | 23869 | 13860 | 0.006031 | 0.002472 | 13272 | 10169 |
| 99719 | 649359 | 649359_ | 7 | 662774 | 655045 | 601018 | 54027 | 22978 | 12814 | 0.005849 | 0.002811 | 12433 | 9579 |
| 99787 | 649256 | 649256_ | 7 | 657929 | 653470 | 603197 | 50273 | 27091 | 14440 | 0.006466 | 0.002368 | 13864 | 10161 |
| 132111 | 32557 | 132111_32557 | 7 | 656437 | 650960 | 578600 | 72360 | 21058 | 10867 | 0.006119 | 0.003606 | 10509 | 8079 |
| 132118 | 32582 | 132118_32582 | 7 | 654930 | 650656 | 607080 | 43576 | 26042 | 14294 | 0.006489 | 0.002138 | 13790 | 10197 |
| 132087 | 32556 | 132087_32556 | 7 | 654400 | 645787 | 598786 | 47001 | 23076 | 13863 | 0.006187 | 0.002489 | 13409 | 10213 |
| 99713 | 649327 | 649327_ | 7 | 650649 | 646087 | 598966 | 47121 | 24562 | 13848 | 0.006167 | 0.002402 | 13394 | 10095 |
| 99714 | 649328 | 649328_ | 7 | 647665 | 643134 | 591908 | 51226 | 25810 | 12805 | 0.005757 | 0.00287 | 12398 | 9632 |
| 99775 | 649248 | 649248_ | 7 | 646200 | 641021 | 582207 | 58814 | 26856 | 12737 | 0.006126 | 0.002876 | 12365 | 9543 |
| 132053 | 32624 | 132053_32624 | 7 | 644927 | 638382 | 580319 | 58063 | 23749 | 13231 | 0.006158 | 0.002785 | 12815 | 9935 |
| 99751 | 649365 | 649365_ | 7 | 644631 | 639152 | 597541 | 41611 | 24013 | 13732 | 0.006254 | 0.002046 | 13204 | 9891 |
| 99806 | 649319 | 649319_ | 7 | 641113 | 636562 | 591400 | 45162 | 25997 | 14028 | 0.006 | 0.001955 | 13447 | 10076 |
| 99765 | 649344 | 649344_ | 7 | 637835 | 629759 | 568292 | 61467 | 40353 | 13474 | 0.006582 | 0.002696 | 12953 | 9939 |
| 99755 | 649279 | 649279_ | 7 | 637744 | 633833 | 586187 | 47646 | 22073 | 13909 | 0.006263 | 0.002023 | 13398 | 10127 |
| 99862 | 649238 | 649238_ | 7 | 637298 | 629317 | 579390 | 49927 | 30716 | 13436 | 0.005798 | 0.00257 | 13010 | 10045 |
| 99808 | 649321 | 649321_ | 7 | 636862 | 631906 | 562023 | 69883 | 22944 | 13549 | 0.00647 | 0.002777 | 13095 | 10060 |
| 99813 | 649254 | 649254_ | 7 | 633968 | 627798 | 566353 | 61445 | 21928 | 13632 | 0.006219 | 0.002746 | 13187 | 10078 |
| 132062 | 32611 | 132062_32611 | 7 | 633898 | 627374 | 583444 | 43930 | 23825 | 13966 | 0.0059 | 0.002128 | 13429 | 10101 |
| 132067 | 32600 | 132067_32600 | 7 | 632622 | 627413 | 576922 | 50491 | 23858 | 13577 | 0.006307 | 0.002455 | 13094 | 10071 |
| 99861 | 649237 | 649237_ | 7 | 632027 | 624636 | 564841 | 59795 | 36646 | 13426 | 0.006395 | 0.002716 | 12955 | 9876 |
| 99774 | 649246 | 649246_ | 7 | 631096 | 627083 | 583342 | 43741 | 24871 | 13897 | 0.006149 | 0.002069 | 13397 | 10068 |
| 132046 | 32564 | 132046_32564 | 7 | 629199 | 616777 | 562344 | 54433 | 30358 | 13831 | 0.00615 | 0.002431 | 13299 | 10095 |
| 132116 | 32537 | 132116_32537 | 7 | 627249 | 623185 | 567253 | 55932 | 21919 | 12862 | 0.006307 | 0.002904 | 12507 | 9628 |
| 99795 | 649241 | 649241_ | 7 | 626166 | 620668 | 576015 | 44653 | 26120 | 14026 | 0.005864 | 0.002489 | 13533 | 10199 |
| 99760 | 649278 | 649278_ | 7 | 621293 | 617075 | 573562 | 43513 | 22540 | 13912 | 0.005998 | 0.002023 | 13393 | 10129 |
| 99742 | 649355 | 649355_ | 7 | 617880 | 612373 | 567392 | 44981 | 26672 | 13883 | 0.005992 | 0.002558 | 13404 | 10059 |
| 99704 | 649329 | 649329_ | 7 | 616295 | 607456 | 551795 | 55661 | 34657 | 12683 | 0.005866 | 0.002771 | 12220 | 9407 |
| 99801 | 649323 | 649323_ | 7 | 616014 | 611591 | 558062 | 53529 | 21407 | 13533 | 0.006416 | 0.002853 | 13125 | 10074 |
| 132044 | 32606 | 132044_32606 | 7 | 615544 | 610854 | 554428 | 56426 | 24057 | 13129 | 0.006307 | 0.002648 | 12707 | 9826 |
| 132121 | 32546 | 132121_32546 | 7 | 615269 | 610710 | 567928 | 42782 | 21814 | 13871 | 0.006249 | 0.002149 | 13385 | 10137 |
| 132085 | 32566 | 132085_32566 | 7 | 614593 | 605304 | 550512 | 54792 | 30555 | 13436 | 0.006354 | 0.00269 | 12928 | 9943 |
| 99718 | 649353 | 649353_ | 7 | 612939 | 607367 | 566128 | 41239 | 27580 | 14128 | 0.005916 | 0.002438 | 13615 | 10121 |
| 99811 | 649316 | 649316_ | 7 | 612835 | 607981 | 558986 | 48995 | 25302 | 13957 | 0.00623 | 0.002612 | 13492 | 10155 |
| 99789 | 649258 | 649258_ | 7 | 610833 | 606291 | 564895 | 41396 | 21041 | 13217 | 0.006134 | 0.002125 | 12742 | 9776 |
| 132110 | 32558 | 132110_32558 | 7 | 610796 | 605239 | 554818 | 50421 | 27009 | 13050 | 0.006261 | 0.002767 | 12633 | 9789 |
| 99750 | 649364 | 649364_ | 7 | 609840 | 605847 | 568779 | 37068 | 22056 | 13884 | 0.00574 | 0.001991 | 13422 | 10146 |
| 92269 | 7420 | 7420_ | 7 | 608789 | 602311 | 552334 | 49977 | 27347 | 13525 | 0.006108 | 0.002855 | 13083 | 10595 |
| 132125 | 32548 | 132125_32548 | 7 | 604921 | 600590 | 549636 | 50954 | 20479 | 12632 | 0.006258 | 0.002841 | 12280 | 9571 |
| 99858 | 649236 | 649236_ | 7 | 603270 | 598741 | 560020 | 38721 | 21154 | 13682 | 0.006638 | 0.001968 | 13200 | 9971 |
| 132127 | 32549 | 132127_32549 | 7 | 600301 | 595809 | 540355 | 55454 | 23904 | 12704 | 0.006273 | 0.002831 | 12332 | 9433 |
| 132050 | 32620 | 132050_32620 | 7 | 599716 | 593284 | 518276 | 75008 | 21823 | 10567 | 0.006616 | 0.003807 | 10165 | 7824 |
| 99745 | 649358 | 649358_ | 7 | 597568 | 589915 | 546836 | 43079 | 27908 | 14214 | 0.006465 | 0.002609 | 13646 | 10237 |
| 132120 | 32545 | 132120_32545 | 7 | 595547 | 590331 | 543495 | 46836 | 29379 | 13952 | 0.006516 | 0.002195 | 13415 | 10107 |
| 99794 | 649240 | 649240_ | 7 | 593445 | 588658 | 545599 | 43059 | 23644 | 13562 | 0.006341 | 0.002043 | 13051 | 9925 |
| 99797 | 649239 | 649239_ | 7 | 589621 | 584539 | 547229 | 37310 | 22854 | 13032 | 0.006112 | 0.002047 | 12613 | 9694 |
| 132042 | 32561 | 132042_32561 | 7 | 589172 | 586050 | 542815 | 43235 | 25900 | 14172 | 0.006298 | 0.002142 | 13611 | 10053 |
| 132064 | 32613 | 132064_32613 | 7 | 587399 | 579916 | 543441 | 36475 | 22685 | 13851 | 0.006153 | 0.002087 | 13344 | 10029 |
| 132130 | 32535 | 132130_32535 | 7 | 582014 | 577697 | 520911 | 56786 | 20584 | 12738 | 0.006282 | 0.002877 | 12372 | 9558 |
| 99703 | 649325 | 649325_ | 7 | 578757 | 573301 | 522671 | 50630 | 22769 | 13607 | 0.005955 | 0.002885 | 13168 | 10127 |
| 132103 | 32578 | 132103_32578 | 7 | 578261 | 569566 | 522956 | 46610 | 25964 | 13344 | 0.006494 | 0.002838 | 12919 | 9913 |
| 132112 | 32581 | 132112_32581 | 7 | 576910 | 569929 | 498505 | 71424 | 24155 | 9580 | 0.006121 | 0.003968 | 9313 | 6977 |
| 99715 | 649332 | 649332_ | 7 | 576650 | 571948 | 526871 | 45077 | 21101 | 13287 | 0.0057 | 0.002777 | 12902 | 10047 |
| 99798 | 649232 | 649232_ | 7 | 574020 | 566877 | 528638 | 38239 | 30546 | 13766 | 0.006613 | 0.001968 | 13224 | 9953 |
| 132058 | 32617 | 132058_32617 | 7 | 567884 | 563155 | 526836 | 36319 | 22485 | 13808 | 0.005834 | 0.002178 | 13351 | 10125 |
| 132051 | 32626 | 132051_32626 | 7 | 566080 | 560892 | 520697 | 40195 | 25002 | 13903 | 0.006221 | 0.00224 | 13372 | 10122 |
| 132054 | 32618 | 132054_32618 | 7 | 563808 | 557659 | 487822 | 69837 | 23236 | 9937 | 0.006612 | 0.004137 | 9645 | 7421 |
| 99812 | 649253 | 649253_ | 7 | 562340 | 553724 | 513978 | 39746 | 24779 | 13968 | 0.006617 | 0.002115 | 13388 | 9899 |
| 99764 | 649342 | 649342_ | 7 | 562244 | 556808 | 518231 | 38577 | 22220 | 13550 | 0.006215 | 0.002113 | 13111 | 9978 |
| 132065 | 32601 | 132065_32601 | 7 | 561885 | 548775 | 487228 | 61547 | 19804 | 10070 | 0.006237 | 0.004063 | 9817 | 7541 |
| 132083 | 32572 | 132083_32572 | 7 | 561552 | 555702 | 488052 | 67650 | 22789 | 9921 | 0.006953 | 0.003929 | 9602 | 7301 |
| 132052 | 32625 | 132052_32625 | 7 | 557941 | 552826 | 514472 | 38354 | 26002 | 13752 | 0.006182 | 0.002041 | 13239 | 10014 |
| 99792 | 649230 | 649230_ | 7 | 557402 | 552101 | 516315 | 35786 | 23426 | 13288 | 0.006571 | 0.002118 | 12829 | 9841 |
| 132078 | 32595 | 132078_32595 | 7 | 553829 | 548372 | 494137 | 54235 | 22551 | 9875 | 0.006387 | 0.004136 | 9579 | 7373 |
| 132131 | 32597 | 132131_32597 | 7 | 549871 | 545633 | 510288 | 35345 | 25030 | 13750 | 0.006061 | 0.002177 | 13292 | 10060 |
| 99706 | 649331 | 649331_ | 7 | 548436 | 541816 | 508070 | 33746 | 25570 | 13619 | 0.005879 | 0.002025 | 13146 | 9982 |
| 99744 | 649357 | 649357_ | 7 | 548136 | 544750 | 501496 | 43254 | 24075 | 13558 | 0.006024 | 0.002701 | 13145 | 10069 |
| 99800 | 649315 | 649315_ | 7 | 545408 | 540594 | 490322 | 50272 | 23603 | 13214 | 0.006273 | 0.002831 | 12782 | 9893 |
| 132128 | 32550 | 132128_32550 | 7 | 545326 | 540191 | 486185 | 54006 | 26138 | 12400 | 0.006147 | 0.002982 | 12017 | 9389 |
| 99705 | 649330 | 649330_ | 7 | 545022 | 541154 | 502949 | 38205 | 25378 | 13951 | 0.0061 | 0.002664 | 13430 | 10180 |
| 99741 | 649354 | 649354_ | 7 | 540297 | 536463 | 505433 | 31030 | 23526 | 13573 | 0.005812 | 0.002074 | 13154 | 10006 |
| 99747 | 649361 | 649361_ | 7 | 540107 | 536586 | 502063 | 34523 | 24287 | 13783 | 0.006117 | 0.002236 | 13282 | 10065 |
| 99752 | 649252 | 649252_ | 7 | 537451 | 530244 | 498398 | 31846 | 22294 | 13353 | 0.006081 | 0.002126 | 12898 | 9842 |
| 99804 | 649317 | 649317_ | 7 | 536906 | 532026 | 478563 | 53463 | 25476 | 12497 | 0.006538 | 0.0029 | 12075 | 9344 |
| 99851 | 649235 | 649235_ | 7 | 532723 | 528297 | 486574 | 41723 | 21188 | 12889 | 0.006041 | 0.002738 | 12538 | 9805 |
| 99805 | 649318 | 649318_ | 7 | 529001 | 521346 | 484698 | 36648 | 22661 | 13767 | 0.006379 | 0.002605 | 13324 | 10093 |
| 99802 | 649313 | 649313_ | 7 | 526951 | 521595 | 485289 | 36306 | 25001 | 14071 | 0.006291 | 0.002135 | 13504 | 10054 |
| 132048 | 32619 | 132048_32619 | 7 | 520292 | 511395 | 446434 | 64961 | 20026 | 9594 | 0.006406 | 0.003999 | 9320 | 7146 |
| 132055 | 32621 | 132055_32621 | 7 | 519049 | 515323 | 475965 | 39358 | 21249 | 13688 | 0.006524 | 0.002178 | 13155 | 9988 |
| 99712 | 649326 | 649326_ | 7 | 516855 | 512657 | 478199 | 34458 | 23998 | 13770 | 0.006022 | 0.002152 | 13263 | 9993 |
| 99854 | 649229 | 649229_ | 7 | 511541 | 506987 | 468128 | 38859 | 28357 | 12833 | 0.006255 | 0.002174 | 12406 | 9622 |
| 132079 | 32596 | 132079_32596 | 7 | 511329 | 505445 | 445410 | 60035 | 22465 | 9804 | 0.00613 | 0.003989 | 9436 | 7210 |
| 99803 | 649314 | 649314_ | 7 | 509476 | 505461 | 468918 | 36543 | 22547 | 13337 | 0.006495 | 0.002084 | 12843 | 9774 |
| 99810 | 649324 | 649324_ | 7 | 507113 | 500917 | 458034 | 42883 | 24989 | 13611 | 0.006545 | 0.00267 | 13166 | 10057 |
| 132056 | 32622 | 132056_32622 | 7 | 506782 | 503939 | 465647 | 38292 | 22884 | 13485 | 0.006382 | 0.0023 | 13015 | 9997 |
| 99748 | 649362 | 649362_ | 7 | 506101 | 502359 | 469969 | 32390 | 25515 | 13664 | 0.006412 | 0.002158 | 13178 | 9972 |
| 99793 | 649231 | 649231_ | 7 | 505895 | 498402 | 451818 | 46584 | 26834 | 13182 | 0.006377 | 0.002832 | 12783 | 9889 |
| 132113 | 32536 | 132113_32536 | 7 | 502713 | 496353 | 457941 | 38412 | 24494 | 13349 | 0.00623 | 0.002161 | 12919 | 9964 |
| 99720 | 649352 | 649352_ | 7 | 497223 | 493018 | 456556 | 36462 | 25045 | 13625 | 0.006158 | 0.002662 | 13139 | 10064 |
| 132063 | 32614 | 132063_32614 | 7 | 490722 | 482256 | 437715 | 44541 | 23758 | 10602 | 0.005735 | 0.0038 | 10187 | 7872 |
| 99772 | 649247 | 649247_ | 7 | 490612 | 486653 | 448631 | 38022 | 25101 | 13400 | 0.006225 | 0.002495 | 12993 | 9942 |
| 99749 | 649363 | 649363_ | 7 | 484399 | 480184 | 442134 | 38050 | 24789 | 13556 | 0.006418 | 0.00267 | 13104 | 10014 |
| 132094 | 32555 | 132094_32555 | 7 | 465128 | 459671 | 411194 | 48477 | 24243 | 9738 | 0.006538 | 0.00399 | 9370 | 7226 |
| 132071 | 32604 | 132071_32604 | 7 | 462003 | 456199 | 402441 | 53758 | 20362 | 9635 | 0.006348 | 0.004141 | 9328 | 7152 |
| 99809 | 649322 | 649322_ | 7 | 457994 | 454704 | 411347 | 43357 | 20528 | 13336 | 0.006785 | 0.003038 | 12853 | 9790 |
| 132060 | 32615 | 132060_32615 | 7 | 369723 | 363494 | 328003 | 35491 | 24430 | 9330 | 0.006188 | 0.004212 | 8942 | 6985 |
| 99859 | 649233 | 649233_ | 7 | 280350 | 277389 | 255466 | 21923 | 21689 | 11848 | 0.006837 | 0.002287 | 11318 | 8709 |
| 99788 | 649257 | 649257_ | 7 | 8855 | 5840 | 1911 | 3929 | 1543 | 10 | 0.084753 | 0.010657 | 4 | 0 |

# Table S5

Results from the BGC analysis of patterns of introgression by locus, genomic location of these loci, and potential functional significance. Code for generating these tables is given in at https://github.com/laninsky/chickadees

**(A)** Breakdown of outlier loci categories. For a detailed description of these categories, please see [**Fig. S5**](#_Fig._S7). Total sig. α loci: 63 (0.93%); total sig. β loci: 133 (1.97%); and total sig. loci 191 (2.83%)

**(B)** Genomic location of all significant positive β loci. Genes bolded and italicized are those found to be within 25,000 bp of multiple SNPs. SNPs in red are found in groups of three or more consecutive loci (potentially indicative of inversions/regions of reduced recombination).

[[back to *Contents*](#_top)]

**(A)**

|  |  | More black-capped | More Carolina |  |  |
| --- | --- | --- | --- | --- | --- |
|  |  | +α | -α | NS α | TOTAL |
| Less introgression | +β | 0 (0%) | 0 (0%) | 66 (0.98%) | 66 (0.98%) |
| More introgression | -β | 5 (0.07%) | 0 (0%) | 62 (0.92%) | 67 (0.99%) |
|  | NS β | 41 (0.61%) | 17 (0.25%) | 6557 (97.17%) | 6615 (98.03%) |
|  | TOTAL | 46 (0.68%) | 17 (0.25%) | 6685 (99.07%) | 6748 (100%) |

**(B)**

| **Chromosome** | **kbp_pos** | **scaffold** | **Gene_1** | **Gene_2** | **Gene_3** | **Gene_4** |
| --- | --- | --- | --- | --- | --- | --- |
| **1** | 83535.186 | CM022157.1 | Fam124a |  |  |  |
| **1A** | 67451.986 | JAAMOC010000547.1 | Rasgrf2_1 |  |  |  |
| **1A** | 68099.678 | JAAMOC010000547.1 |  |  |  |  |
| **1A** | 68185.802 | JAAMOC010000547.1 |  |  |  |  |
| **1A** | 68667.748 | JAAMOC010000547.1 |  |  |  |  |
| **2** | 17572.181 | JAAMOC010000550.1 |  |  |  |  |
| **2** | 117623.689 | JAAMOC010000550.1 | Tgs1 |  |  |  |
| **2** | 122667.037 | JAAMOC010000550.1 | Dsg2 | Dsg1a |  |  |
| **2** | 130025.35 | JAAMOC010000550.1 | Mtcl1 |  |  |  |
| **3** | 3503.135 | JAAMOC010000568.1 |  |  |  |  |
| **3** | 7989.542 | JAAMOC010000565.1 | Rps6kc1 | Angel2 |  |  |
| **3** | 60471.91 | JAAMOC010000565.1 |  |  |  |  |
| **4** | 4321.227 | JAAMOC010000569.1 |  |  |  |  |
| **4** | 11788.267 | JAAMOC010000569.1 |  |  |  |  |
| **5** | 37826.625 | CM022158.1 | Kcnk10 |  |  |  |
| **7** | 20052.469 | JAAMOC010000580.1 | Map3k20 |  |  |  |
| **8** | 12679.478 | JAAMOC010000582.1 | Slc25a24 |  |  |  |
| **8** | 16457.074 | JAAMOC010000582.1 | Dnm3 | Mettl13 | Itpa | Vamp4 |
| **11** | 9778.017 | CM022161.1 |  |  |  |  |
| **11** | 15213.194 | CM022161.1 |  |  |  |  |
| **12** | 8103.289 | CM022162.1 |  |  |  |  |
| **14** | 8418.564 | CM022164.1 | Clec19a |  |  |  |
| **CHR_UNK** | 0.245 | JAAMOC010000356.1 | Znf574_1 | Znf574_0 |  |  |
| **CHR_UNK** | 6.408 | JAAMOC010000413.1 |  |  |  |  |
| **Z** | 1126.264 | CM022174.1 |  |  |  |  |
| **Z** | 1636.163 | CM022174.1 |  |  |  |  |
| **Z** | 2559.538 | CM022174.1 | Sncaip |  |  |  |
| **Z** | 3047.882 | CM022174.1 |  |  |  |  |
| **Z** | 4626.93 | CM022174.1 | Trim36 |  |  |  |
| **Z** | 5616.433 | CM022174.1 |  |  |  |  |
| **Z** | 6923.167 | CM022174.1 |  |  |  |  |
| **Z** | 9026.581 | CM022174.1 | Aopep |  |  |  |
| **Z** | 12088.147 | CM022174.1 | Fxn | Pip5k1b |  |  |
| **Z** | 12382.243 | CM022174.1 | Mllt3 |  |  |  |
| **Z** | 12533.625 | CM022174.1 |  |  |  |  |
| **Z** | 14244.53 | CM022174.1 |  |  |  |  |
| **Z** | 14658.884 | CM022174.1 | Cer1 |  |  |  |
| **Z** | 15627.54 | CM022174.1 |  |  |  |  |
| **Z** | 20062.247 | CM022174.1 | Dmrt1 |  |  |  |
| **Z** | 26645.92 | CM022174.1 | Kif2a | Dimt1 | Ipo11 |  |
| **Z** | 26730.719 | CM022174.1 |  |  |  |  |
| **Z** | 29671.922 | CM022174.1 |  |  |  |  |
| **Z** | 30058.601 | CM022174.1 |  |  |  |  |
| **Z** | 32320.285 | CM022174.1 | Ccl28 | Tmem267 |  |  |
| **Z** | 32751.256 | CM022174.1 |  |  |  |  |
| **Z** | 33062.411 | CM022174.1 | Plcxd3 |  |  |  |
| **Z** | 37177.087 | CM022174.1 | ***Rusc2*** |  |  |  |
| **Z** | 37206.266 | CM022174.1 | ***Rusc2*** |  |  |  |
| **Z** | 42669.716 | CM022174.1 | Syt4 |  |  |  |
| **Z** | 45088.124 | CM022174.1 | Ctif |  |  |  |
| **Z** | 45702.619 | CM022174.1 | Myo5b_2 | Myo5b_4 |  |  |
| **Z** | 46828.62 | CM022174.1 | Rnf38 |  |  |  |
| **Z** | 48976.018 | CM022174.1 |  |  |  |  |
| **Z** | 52607.846 | CM022174.1 |  |  |  |  |
| **Z** | 52690.929 | CM022174.1 |  |  |  |  |
| **Z** | 53258.33 | CM022174.1 | Ptar1 |  |  |  |
| **Z** | 54530.532 | CM022174.1 | Tmc1 |  |  |  |
| **Z** | 60414.667 | CM022174.1 |  |  |  |  |
| **Z** | 62192.702 | CM022174.1 | Fbxl17 |  |  |  |
| **Z** | 64331.34 | CM022174.1 |  |  |  |  |
| **Z** | 68426.987 | CM022174.1 | Lingo2 |  |  |  |
| **Z** | 69757.251 | CM022174.1 | Aptx | Ervk6_1 | Dnaja1_0 | Smu1 |
| **Z** | 69812.869 | CM022174.1 | B4galt1 |  |  |  |
| **Z** | 70801.835 | CM022174.1 | Pcsk1 |  |  |  |
| **Z** | 71650.395 | CM022174.1 | Rad23b |  |  |  |
| **Z** | 72917.551 | CM022174.1 |  |  |  |  |

# References

Eaton, D. A. R., Spriggs, E. L., Park, B., & Donoghue, M. J. (2017). Misconceptions on missing data in RAD-seq phylogenetics with a deep-scale example from flowering plants. *Systematic Biology*, *66*(3), 399–412.

Lee, K. M., Kivelä, S. M., Ivanov, V., Hausmann, A., Kaila, L., Wahlberg, N., & Mutanen, M. (2018). Information dropout patterns in restriction site associated DNA phylogenomics and a comparison with multilocus Sanger data in a species-rich moth genus. *Systematic Biology*, *67*(6), 925–939.

Pante, E., Abdelkrim, J., Viricel, A., Gey, D., France, S. C., Boisselier, M. C., & Samadi, S. (2015). Use of RAD sequencing for delimiting species. *Heredity*, *114*(5), 450–459.

Taylor, S. A., White, T. A., Hochachka, W. M., Ferretti, V., Curry, R. L., & Lovette, I. (2014). Climate-mediated movement of an avian hybrid zone. *Current Biology*, *24*(6), 671–676.

[[back to *Contents*](#_top)]
